# The Amazon rain forest plant *Uncaria tomentosa* (cat’s claw) and its specific proanthocyanidin constituents are potent inhibitors and reducers of both brain plaques and tangles

**DOI:** 10.1038/s41598-019-38645-0

**Published:** 2019-02-06

**Authors:** Alan D. Snow, Gerardo M. Castillo, Beth P. Nguyen, Paula Y. Choi, Joel A. Cummings, Judy Cam, Qubai Hu, Thomas Lake, Weihong Pan, Abba J. Kastin, Daniel A. Kirschner, Steven G. Wood, Edward Rockenstein, Eliezer Masliah, Stephen Lorimer, Rudolph E. Tanzi, Lesley Larsen

**Affiliations:** 1 Cognitive Clarity Inc., Edmonds, WA USA; 2 ProteoTech Inc., Kirkland, WA USA; 3 Blood-Brain Barrier Laboratory, Pennington Biomedical Research Center at Louisiana State University, Baton Rouge, Louisiana USA; 4 Department of Biology, Boston College, Chestnut Hill, MA USA; 5 Department of Chemistry and Biochemistry, Brigham Young University, Provo, UT USA; 6 Departments of Neurosciences and Pathology, University of California- San Diego, La Jolla, CA USA; 7 Department of Chemistry, University of Otago, Dunedin, New Zealand; 8 Genetics and Aging Research Unit, Department of Neurology, Massachusetts General Hospital and Harvard Medical School, Charlestown, MA USA; 9 Present Address: PharmaIN Corp., Bothell, WA USA; 10 Present Address: Healthcare Legacy Consulting LLC, Dallas, TX USA; 11 Preclinical GPS, Washington University, St. Louis, MO USA; 12 Present Address: VicLink Ltd., Wellington, New Zealand; 13 Present Address: Biopotentials Sleep Center, Baton Rouge, LA USA; 14 Present Address: Division of Neurosciences, National Institute on Aging, Bethesda, MD USA

## Abstract

Brain aging and Alzheimer’s disease both demonstrate the accumulation of beta-amyloid protein containing “plaques” and tau protein containing “tangles” that contribute to accelerated memory loss and cognitive decline. In the present investigation we identified a specific plant extract and its constituents as a potential alternative natural solution for preventing and reducing both brain “plaques and tangles”. PTI-00703 cat’s claw (*Uncaria tomentosa* from a specific Peruvian source), a specific and natural plant extract from the Amazon rain forest, was identified as a potent inhibitor and reducer of both beta-amyloid fibrils (the main component of “plaques”) and tau protein paired helical filaments/fibrils (the main component of “tangles”). PTI-00703 cat’s claw demonstrated both the ability to prevent formation/aggregation and disaggregate preformed Aβ fibrils (1–42 and 1–40) and tau protein tangles/filaments. The disaggregation/dissolution of Aβ fibrils occurred nearly instantly when PTI-00703 cat’s claw and Aβ fibrils were mixed together as shown by a variety of methods including Thioflavin T fluorometry, Congo red staining, Thioflavin S fluorescence and electron microscopy. Sophisticated structural elucidation studies identified the major fractions and specific constituents within PTI-00703 cat’s claw responsible for both the observed “plaque” and “tangle” inhibitory and reducing activity. Specific proanthocyanidins (i.e. epicatechin dimers and variants thereof) are newly identified polyphenolic components within *Uncaria tomentosa* that possess both “plaque and tangle” reducing and inhibitory activity. One major identified specific polyphenol within PTI-00703 cat’s claw was epicatechin-4β-8-epicatechin (i.e. an epicatechin dimer known as proanthocyanidin B2) that markedly reduced brain plaque load and improved short-term memory in younger and older APP “plaque-producing” (TASD-41) transgenic mice (bearing London and Swedish mutations). Proanthocyanidin B2 was also a potent inhibitor of brain inflammation as shown by reduction in astrocytosis and gliosis in TASD-41 transgenic mice. Blood-brain-barrier studies in Sprague-Dawley rats and CD-1 mice indicated that the major components of PTI-00703 cat’s claw crossed the blood-brain-barrier and entered the brain parenchyma within 2 minutes of being in the blood. The discovery of a natural plant extract from the Amazon rain forest plant (i.e. *Uncaria tomentosa* or cat’s claw) as both a potent “plaque and tangle” inhibitor and disaggregator is postulated to represent a potential breakthrough for the natural treatment of both normal brain aging and Alzheimer’s disease.

## Introduction

Brain aging and Alzheimer’s disease are both known to be characterized by two major hallmarks, the accumulation of beta-amyloid (Aβ) “plaques” and tau-protein containing neurofibrillary “tangles”^1–3^. The accumulation of Aβ “plaques” in healthy people have been found in the brains of individuals as early as 20 years old and increases progressively as one ages^4^. The build-up of tau protein in brain containing neurofibrillary tangles is also believed to accumulate as one ages as well^5,6^. Normal brain aging in healthy individuals therefore involves the accumulation of both “plaques and tangles” and is postulated to be the real reason we lose memory and cognition as we age^1–3^. Thus, mild memory loss is a phenomenon that seems to occur as part of the normal brain aging process. When the accumulation of brain “plaques and tangles” begins to be excessive, memory loss and cognitive decline worsen and appear initially clinically as mild cognitive impairment (MCI). Further accumulation of brain “plaques and tangles” associated with increased brain inflammation and concurrent neuronal loss can then eventually lead to the diagnosis of Alzheimer’s disease [(based on memory testing, the ruling out of other diseases, and more recently using brain imaging techniques to access “plaque” (i.e. beta-amyloid protein) and “tangle” (i.e. tau protein) load in live patients^7–10^. In Alzheimer’s disease, besides the accumulation of thousands, to hundreds of thousands of “plaques and tangles” in specific brain areas including hippocampus and cortex, the marked brain inflammation is believed to contribute to the exuberating neuronal death and disruption of synapses^11,12^. Thus, the trilogy of “Plaques, Tangles and Inflammation” (referred to as “PTI”) is postulated to lead to a marked potential and rapid decline in memory and cognition in the aging population.

There continues to be a concentrated effort by pharmaceutical companies to produce an FDA-approved drug to stop and reverse brain “plaque and tangle” load in an effort to halt cognitive decline and improve memory loss. Such efforts were first initiated from epic innovative investigations that demonstrated that antibodies to Aβ reduced brain plaque load concurrent with cognitive and memory improvement^13^. Initial studies utilized beta-amyloid precursor protein (APP) transgenic animals, genetically engineered to accumulate Aβ amyloid plaques in brain as these animals aged. Both double transgenic (i.e. London and Swedish mutations, and beta-amyloid precursor protein and presenilin-1) and single-transgenic (i.e. Tg2576) mice recaptured the excessive brain plaque load correlating with memory loss observed in humans^14,15^. Reducing brain Aβ plaque load in transgenic mice with a variety of different approaches^16–22^ led to improved memory restoration in these animals as demonstrated by improvements in Morris water maze testing (the gold standard for testing of hippocampus-dependent memory) and probe trials. These tests (along with testing in “tangle’ transgenic mice and/or in testing in “plaque and tangle” double and triple transgenic animals)^22^ over the years led to the development of several potential drugs that initially showed promise in human clinical trials^23–28^. One such promising drug was the human monoclonal antibody called “aducanumab” (BIIB37) that entered the brain and reduced soluble and insoluble Aβ deposits in a dose-dependent manner^29^. Reduction of brain plaque load as shown in human clinical trials with this antibody led to a slowing of clinical cognitive decline in Alzheimer’s patients measured by clinical dementia rating –sum of boxes, and mini-mental state examination scores^29^. This drug is undergoing extensive human clinical trials in large populations of patients and holds promise as a potential effective drug for the treatment of Alzheimer’s disease. Another promising drug is BAN2401, developed by Eisai and Biogen that recently reported a statistically significant slowing in clinical decline and reduction of beta-amyloid protein in brain following 18 months of treatment^30^. This anti-beta-amyloid protein protofibril antibody looks promising as well. The reduction of brain Aβ plaque load accompanied by a slowing in cognitive decline provides compelling evidence and support for the amyloid hypothesis^31,32^ that indicates that Aβ amyloid fibril deposits and plaque accumulation leads to memory loss and cognitive decline, and that by reducing brain Aβ amyloid load in brain leads to improvements in memory.

In the present investigation we sought to determine if a potential natural product (plant or otherwise) can inhibit and reduce both brain Aβ “plaques” and tau “tangles”. The identification of such a natural product might contribute instant beneficial effects for people around the world to help prevent and/or reduce their brain “plaque and tangle” load as they age, and while they are waiting for effective pharmaceutical drugs to hit the market.

In our studies we used “plaque” (beta-amyloid protein) and “tangle” (tau protein) screening technologies to discover and identify a specific natural Amazon rain forest plant known as PTI-00703 cat’s claw (i.e. *Uncaria tomentosa* from a specific Peruvian source) that possessed both potent Aβ “plaque” and tau protein “tangle” inhibitory and reducing activity. Sophisticated structural elucidation studies identified the major fractions and specific small molecule polyphenolic compounds within “PTI-00703 cat’s claw primarily responsible for the observed “plaque and tangle” inhibitory and reducing activity. Specific dimers of epicatechin and variants thereof, known as proanthocyanidins, were newly identified components within *Uncaria tomentosa* (cat’s claw) that were potent natural plant inhibitors and disaggregators of both “plaques and tangles”. More specifically, an epicatechin-dimer, with the structure of epicatechin-4β-8-epicatechin (also known as proanthocyanidin B2) is a major component of *Uncaria tomentosa* that has natural preventative and therapeutic “plaque and tangle” dissolving and inhibitory activity. Other newly identified polyphenols within PTI-00703 cat’s claw that demonstrated “plaque-reducing” activity included proanthocyanidin B4 (i.e. catechin-4α → 8-epicatechin), proanthocyanidin C1 (i.e. epicatechin-4β → 8-epicatechin-4β → 8-epicatechin), an epicatechin trimer (i.e. epicatechin-4β → 8-epicatechin-4β → 8-epicatechin), epiafzelechin-4β → 8-epicatechin and an epicatechin tetramer (i.e. epicatechin-4β → 8-epicatechin-4β → 8-epicatechin-4β → 8-epicatechin). The discovery of a specific natural plant extract derived from the Amazon rain forest plant and the identification of a major proanthocyanidin component (i.e. proanthocyanidin B2) as a potent inhibitor and reducer of both “plaques and tangles” is postulated to represent an important breakthrough for the natural and effective treatment of both normal brain aging and Alzheimer’s disease.

## Results

### Discovery and identification of *Uncaria tomentosa* (cat’s claw) as a potent natural plant extract inhibitor/disrupter of beta-amyloid protein “plaques”

**Figure 1:**
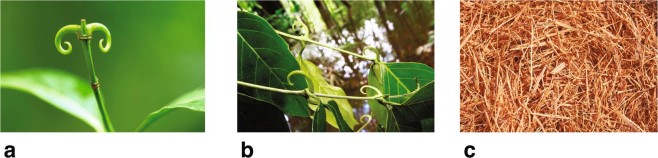
PTI-00703 cat’s claw: a specific plant extract obtained from the amazon rain forest woody vine, *Uncaria tomentosa* (i.e. cat’s claw). (**a**,**b**) *Uncaria tomentosa* (cat’s claw) with its distinctive curved claw-like thorns which project from the base of its leaves. (**c**) Cat’s claw bark is used for extraction purposes and bark bundles are sold in the Peruvian market place. Cognitive Clarity Inc. has copyright license for digital and print use for the images of (**a**) from Getty Images, Chicago, IL, USA; (**b**) from Amazon-Images MBSI/Alamy Stock Photos; and (**c**) from imageBROKER/Alamy Stock Photo.

*Uncaria tomentosa*, also known as “Uňa de Gato” (in Spanish) or cat’s claw (in English) is a woody vine that grows slowly in the Amazon rain forest. The vine is referred to as “cat’s claw” because of its distinctive claw-like thorns which project from the base of its leaves (Fig. 1a,b). The vine can take 20 years to reach maturity and can grow over 100 feet in length as it attaches and wraps itself around the native trees. The cat’s claw bark is harvested for extraction purposes (Fig. 1c) and sold in the Peruvian marketplace as bark bundles. It is found abundantly in the foothills in the Amazon rain forest at elevations of 2,000 to 8,000 feet. There are about 34 species of *Uncaria*, with *Uncaria tomentosa* being the most common species^33,34^.

In the following set of studies, assay-guided fractionation and sophisticated structure elucidation techniques identified specific polyphenolic and proanthocyanidin ingredients within PTI-00703 cat’s claw (i.e. *Uncaria tomentosa* from a specific Peruvian source) that possess a natural ability to reduce and inhibit both beta-amyloid protein “plaques” and tau protein “tangles”, the two hallmarks of brain aging, and the pathological hallmarks of Alzheimer’s disease.

### PTI-00703 cat’s claw is a potent inhibitor of beta-amyloid protein (Aβ) 1–40 fibril formation

**Figure 2:**
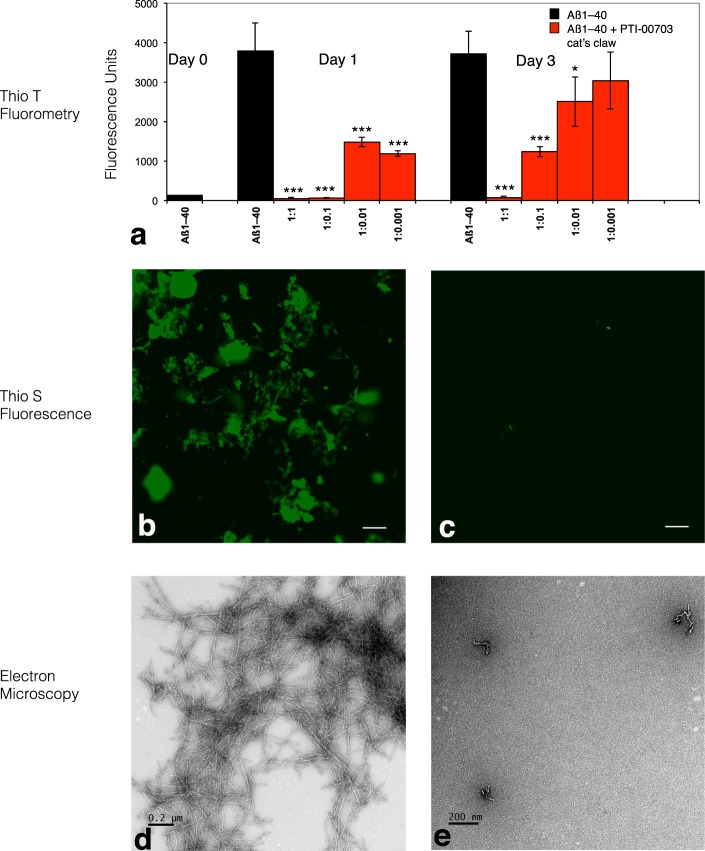
PTI-00703 cat’s claw is a potent inhibitor of Aβ 1–40 fibril formation. (**a**) PTI-00703 cat’s claw caused a dose-dependent inhibition of Aβ 1–40 fibril formation as assessed by Thioflavin T fluorometry. **p < 0.05, ***p < 0.001, by Student’s t-test, Bars represent mean +/− SEM. n = 5. (**b**) Aβ1–40 at 1 day shows positive Thioflavin S fluorescence (indicative of amyloid fibrils) under fluorescent light. Scale bar = 25 µm. (**c**) PTI-00703 cat’s claw inhibited Aβ fibril formation as shown by a marked reduction in Thioflavin S fluorescence (Aβ: PTI-00703 cat’s claw 1:1 wt/wt). Scale bar = 25 µm. (**d**) Electron microscopy at 1 day demonstrated abundant Aβ 1–40 amyloid fibrils with a characteristic fibril diameter of 10-20 nm. Scale bar = 0.2 µm. (**e**) Aβ1–40 in the presence of PTI-00703 cat’s claw demonstrated only formation of amorphous non-fibrillar material (Aβ: PTI-00703 cat’s claw 1:1 wt/wt). Scale bar = 200 nm.

In one study, Aβ 1–40 was incubated in the absence or presence of PTI-00703 cat’s claw for 3 days with aliquots analyzed at 0, 1, and 3 days. Thioflavin T fluorometry demonstrated that Aβ 1–40 fibril formation occurred within 1 day by an increase in fluorescence units from nearly 0 (at day 0) to 3500 fluorescence units within 1 day (Fig. 2a). Thioflavin S fluorescence of Aβ 1–40 fibrils at 1 day demonstrated a positive green fluorescence as viewed under fluorescent light indicative of fibril formation (Fig. 2b).This was confirmed by a positive red/green birefringence following Congo red staining as viewed under polarized light (not shown). Electron microscopy demonstrated abundant Aβ 1–40 fibrils by 1 day, with a fibril diameter of 10–20 nm (Fig. 2d). On the other hand, PTI-00703 cat’s claw significantly inhibited Aβ 1–40 fibril formation as demonstrated by a dose-dependent reduction in Thioflavin T fluorometry at 1 and 3 days (Fig. 2a), and a marked decrease in Thioflavin S fluorescence (Fig. 2c) and Congo red staining (not shown). Electron microscopy additionally demonstrated that in the presence of PTI-00703 cat’s claw, the majority of the Aβ 1–40 was inhibited from forming fibrils and only formed amorphous non-fibrillar Aβ (Fig. 2d). This study demonstrated that PTI-00703 cat’s claw markedly inhibited Aβ 1–40 fibril formation.

### PTI-00703 cat’s claw also disrupts/disaggregates pre-formed Aβ 1–42 fibrils nearly instantly

**Figure 3:**
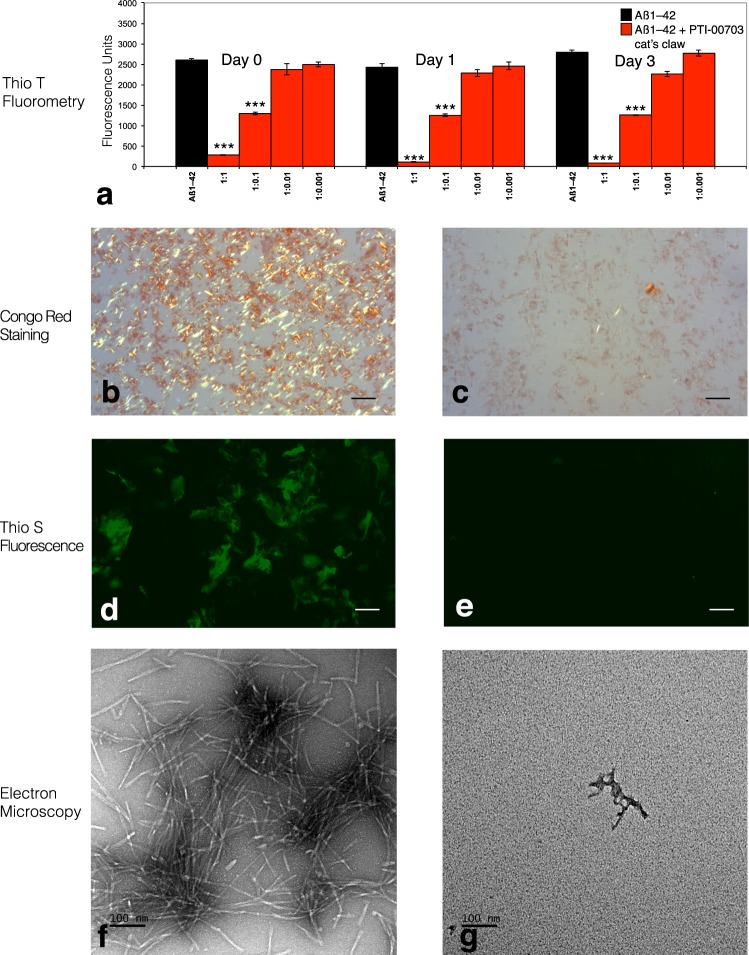
PTI-00703 cat’s claw was also a potent disaggregator/disrupter of Aβ 1–42 fibrils almost instantly. (**a**) PTI-00703 cats claw caused a dose-dependent disruption/disaggregation of Aβ 1–42 fibrils as assessed by Thioflavin T fluorometry. A dose dependent disaggregation/disruption of Aβ 1–42 fibrils was observed nearly immediately (upon mixing). **p < 0.05, ***p < 0.001 by Student’s t-test. Bars represent mean +/− SEM. n = 5. (**b**) Congo red staining of Aβ 1–42 fibrils by day 0 demonstrated a robust red/green birefringence under polarized light indicative of amyloid fibrils. Scale bar = 25 µm. (**c**) Aβ1–42 in the presence of PTI-00703 cat’s claw near completely diminished Congo red staining indicative of a disruption/disaggregation of Aβ 1–42 fibrils (Aβ: PTI-00703 cat’s claw 1:1 wt/wt). Scale bar = 25 µm. (**d**) Aβ 1–42 by day 0 also showed positive Thioflavin S fluorescence under fluorescent light. Scale bar = 25 µm. (**e**) PTI-00703 cat’s claw disaggregated/disrupted Aβ 1–42 fibrils as shown by a marked reduction in Thioflavin S fluorescence (Aβ: PTI-00703 cat’s claw 1:1 wt/wt). Scale bar = 25 µm. (**f**) Electron microscopy at day 0 demonstrated abundant Aβ 1–42 amyloid fibrils with a characteristic fibril diameter of 10-20 nm. Scale bar = 100 nm (**g**) Aβ 1–42 fibrils in the presence of PTI-00703 cat’s claw demonstrated primarily the formation of amorphous non-fibrillar material, nearly immediately upon mixing (Aβ: PTI-00703 cat’s claw 1:1 wt/wt). Scale bar = 100 nm.

In another study, the dose-dependent effects of PTI-00703 cat’s claw on disruption/disaggregation of pre-formed Aβ 1–42 fibrils was determined. Aβ 1–42 was first incubated at 37 °C to observe instant amyloid fibril by Thioflavin T fluorometry at day 0 (Fig. 3a, day 0), positive Congo red staining [(i.e. red-green birefringence as viewed under polarized light)(Fig. 3b) and positive Thioflavin S fluorescence (Fig. 3d). Aβ 1–42 also demonstrated amyloid fibrils at day 0, as shown by negative stain electron microscopy (Fig. 3f). In the presence of PTI-00703 cat’s claw, Aβ 1–42 fibrils were near instantly (within a few minutes of mixing) disaggregated and/or dissolved as shown by a marked decrease in Thioflavin T fluorometry (Fig. 3a, Day 0, 1 and 3), Congo red staining (Fig. 3c) and Thioflavin S fluorescence (Fig. 3e). Electron microscopy revealed that PTI-00703 cat’s claw disaggregated and reduced pre-formed Aβ 1–42 fibrils into mostly amorphous non-fibrillar material (Fig. 3g). This study demonstrated that PTI-00703 cat’s claw was also a potent disaggregator/dissolver of pre-formed Aβ 1–42 fibrils and appeared to do so within minutes of the interaction with Aβ 1–42 fibrils.

### PTI-00703 cat’s claw is also a potent inhibitor/disrupter of “tangles” as demonstrated by *in vitro* assays

**Figure 4:**
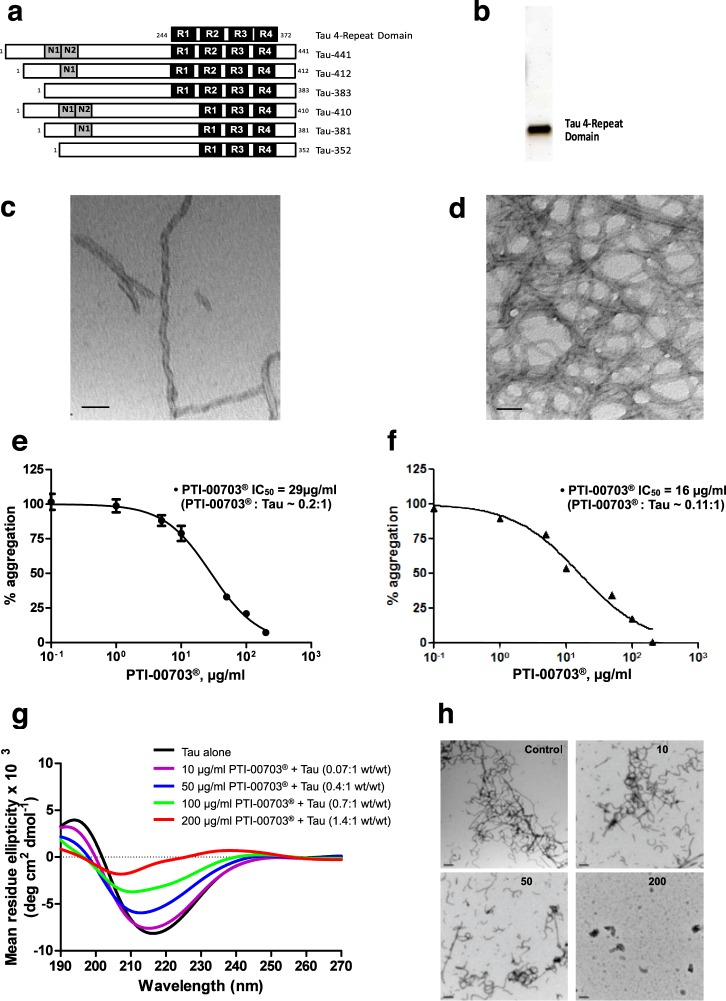
PTI-00703 cat’s claw also inhibited tau protein tangles and filament formation, and reduced preformed tau fibrils. (**a**) Tau protein isoforms and a purified tau 4-repeat domain. Diagram of six tau isoforms aligned with a “new” tau 4-repeat domain that was generated (top of image). (**b**) SDS-PAGE and silver staining showed purified tau 4-repeat domain protein at ~15 kDa (250 ng of protein loaded). (**c**,**d**) Electron microscopy showed paired helical filaments (i.e. tangles) generated from *in vitro* aggregated tau 4-repeat domain (10 µM) in the presence of heparin. Scale bars = 50 nm. (**e**) Inhibition of tau protein fibril formation by PTI-00703 cat’s claw using a Thioflavin S binding assay. n = 4. (**f**) PTI-00703 cat’s claw also disrupted/disaggregated pre-formed tau protein filaments and fibrils using a Thioflavin S binding assay. n = 4 (**g**) PTI-00703 cat’s claw inhibited tau protein fibril formation in a dose-dependent manner as determined by CD spectroscopy. Tau protein alone (black bar) showed a characteristic beta-sheet CD spectra with a minima at a wavelength of 218 nm. Increasing concentrations of PTI-00703 cat’s claw demonstrated a dose-dependent reduction of the tau protein beta-sheet secondary folding as shown by a dose-dependent flattening of the 218 nm minima. n = 3. (**h**) PTI-00703 cat’s claw inhibited tau protein fibril formation in a dose-dependent manner as shown by EM. Increasing concentrations of PTI-00703 cat’s claw (i.e. 10 µg/ml, 50 µg/ml and 200 µg/ml) demonstrated reduction and disaggregation of tau protein filaments and fibrils into primarily amorphous non-fibrillar material. Scale bars = 200 nm.

The human tau 4-repeat domain (generated as recombinant protein in human *E. coli*) that overlaps with amino acids Q244-E372 of tau-441 was expressed in *E. coli* (Fig. 4a,b). It formed paired helical filaments in the presence of heparin identical to brain paired helical filaments in “tangles” as shown by electron microscopy (Fig. 4c,d). The tau 4-repeat domain and full-length tau protein was used to determine if PTI-00703 cat’s claw was also an inhibitor/reducer of tau protein paired helical filaments. In one assay, a Thioflavin S binding assay determined the effects of PTI-00703 cat’s claw on inhibition of tau protein filament/fibril formation. As shown in Fig. 4e, PTI-00703 cat’s claw inhibited tau protein filament/fibril formation with an IC_50_ of 29 µg/ml with a PTI-00703 cat’s claw: tau protein ratio of ~0.2:1. This study demonstrated that PTI-00703 cat’s claw can inhibit tau protein from forming paired helical filaments/tau fibrils.

In another study, PTI-00703 cat’s claw also disrupted/reduced pre-formed tau protein filaments/fibrils as determined using a Thioflavin S binding assay. The IC_50_ of PTI-00703 cat’s claw in this study was 16 µg/ml with a PTI-00703 cat’s claw: tau protein ratio of ~0.11:1 (Fig. 4f). Circular dichroism (CD) spectroscopy further demonstrated that in 24 hours tau protein in the presence of heparin formed a β-sheet secondary folding confirmation (indicative of tau protein filaments/fibrils) with a minima at 218 nm (Fig. 4g, tau alone). Incubation of tau protein + heparin with increasing concentrations of PTI-00703 cat’s claw demonstrated a dose-dependent reduction of tau protein β-sheet formation observed by a flattening of the curve at 218 nm shown by CD spectroscopy (Fig. 4g). This demonstrated that PTI-00703 cat’s claw causes tau protein tangle disruption by a reduction of the β-sheet secondary folding of tau protein tangles.

Electron microscopy (Fig. 4h) confirmed the data observed by other methods as described above. Abundant tau filaments/fibrils were observed by EM within 3-days of incubation at 37 °C (Fig. 4h, control). In the presence of increasing concentrations of PTI-00703 cat’s claw, less and less tau protein filaments/fibrils were formed within a 3-day co-incubation period (Fig. 4h, 50 µg/ml). At the highest concentration tested, PTI-00703 cat’s claw completely abolished the formation of tau protein filaments and fibrils (Fig. 4h, 200 µg/ml). This study demonstrated that PTI-00703 cat’s claw was also effective as an inhibitor and a disrupter of tau tangles (i.e. paired helical filaments) as well. Thus, PTI-00703 cat’s claw was identified as a natural inhibitor and reducer of both Aβ fibrils and tau protein filaments/fibrils.

### Initial isolation and testing of the active ingredients within PTI-00703 cat’s claw responsible for Aβ fibril inhibitory and reducing activity

**Figure 5:**
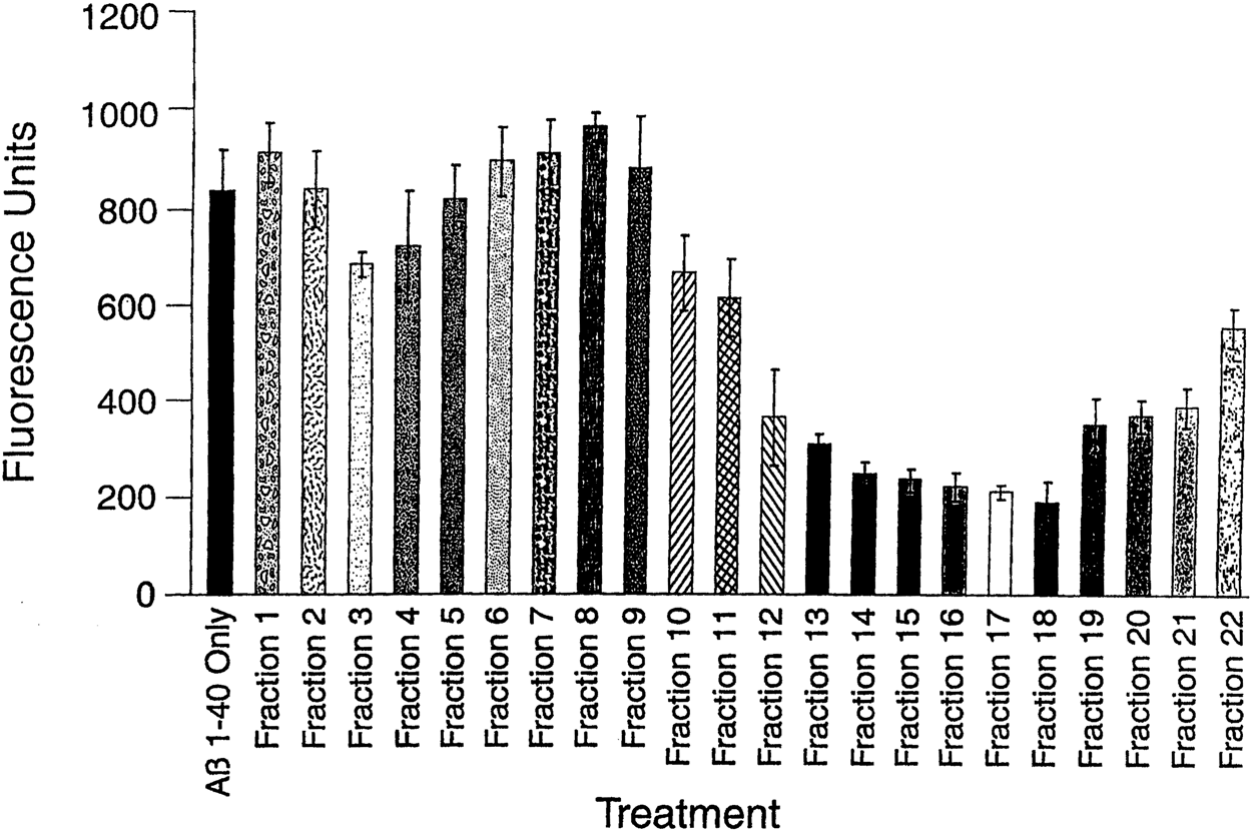
A Thioflavin T fluorometry assay identified water-soluble fractions of cat’s claw (*Uncaria tomentosa*) that possessed Aβ fibril reducing/disaggregation activity. Fractions 13–18 (52–72 minutes) contain components that reduce/dissolve pre-formed Aβ 1–40 fibrils by 60–75%. Bars represent mean +/− SEM. n = 4.

Assay-guided affinity fractionation and high-pressure liquid chromatography (HPLC) separated and purified the major Aβ fibril inhibitory and reducing active components present in PTI-00703 cat’s claw. Aliquots from fractions 1–22 (i.e. 4 to 84 minutes) were incubated with pre-formed Aβ 1–40 (for 3 days) (Fig. 5) or Aβ 1–42 fibrils (instantly formed), for 2 hours (at a wt/wt ratio of 1:1) and tested for their ability to reduce/disassemble pre-formed Aβ fibrils using a Thioflavin T fluorometry assay as previously described^35^. As shown in Fig. 5, fibrillar Aβ 1–40 alone demonstrated a fluorescence of 836 +/− 61 fluorescent units. Fractions 13–18 (i.e. 52–72 minutes) demonstrated the greatest ability (from 60–75%) to reduce/disassemble pre-formed Aβ 1–40 fibrils, as indicated by a marked lowering of fluorescence units (Fig. 5). Similar results were obtained with pre-formed Aβ 1–42 fibrils (not shown). This study suggested that the most active Aβ amyloid fibril inhibitory components within PTI-00703 cat’s claw (*Uncaria* tomentosa) were located within fractions 13–18.

### Purification of the major Aβ amyloid inhibitory components in the water-soluble fraction of *Uncaria tomentosa* (PTI-777 isolation protocol)

**Figure 6:**
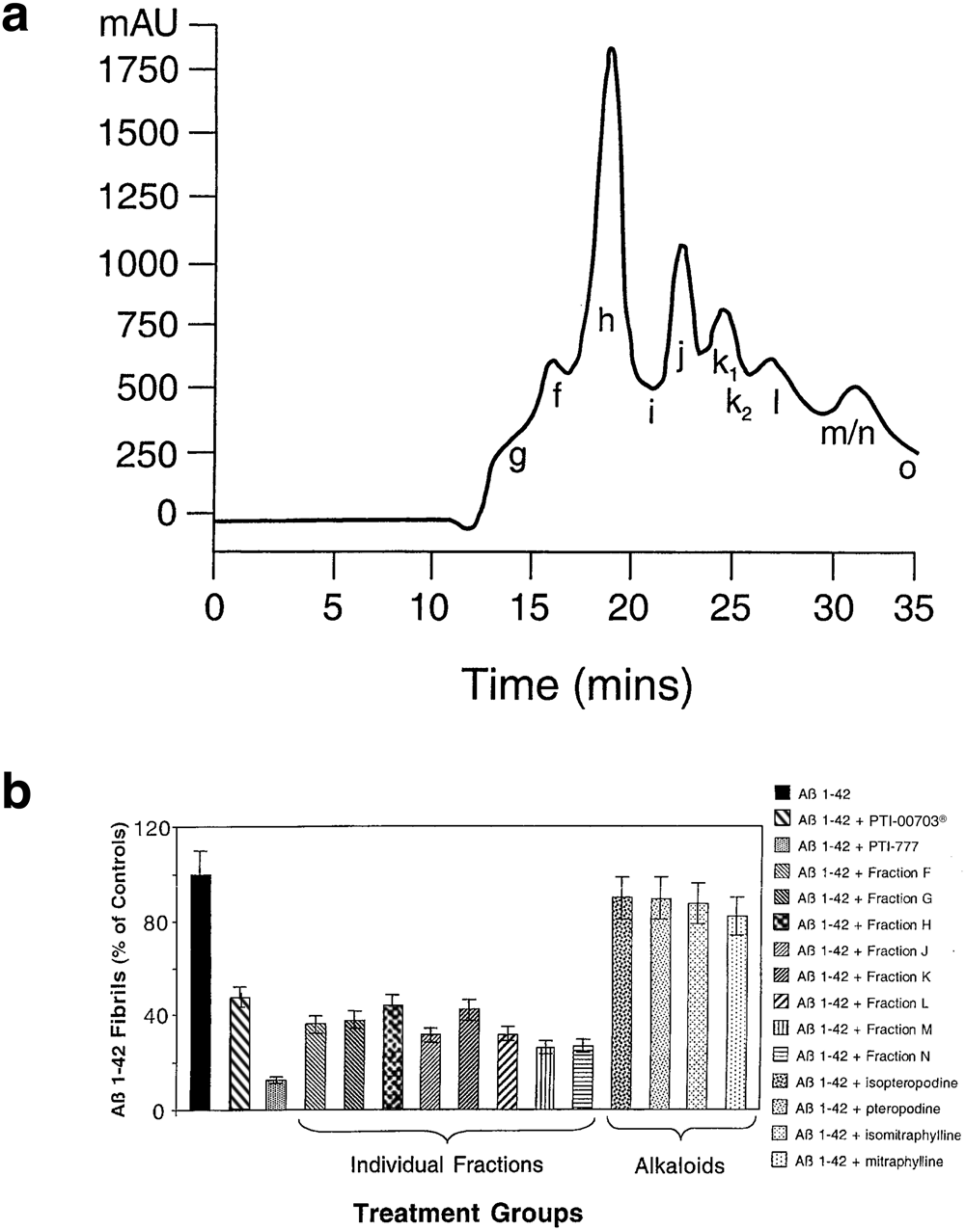
PTI-777 profile (major Aβ inhibitory ingredients in PTI-00703 cat’s claw). (**a**) Preparative HPLC Profile of PTI-777 that demonstrated the major water-soluble Aβ inhibitory components isolated from *Uncaria tomentosa* (cat’s claw), noted (from left to right) as Fractions g, f, h, i, j, k_1_, k_2_, l, m/n and o. (**b**) Identification of each fraction using a variety of techniques including PTI-00703 cat’s claw, PTI-777, and individual fractions of PTI-777 were assessed by Thioflavin T fluorometry. PTI-00703 cat’s claw, PTI-777, and individual fractions f-n were all significant effective disrupters/reducers of Aβ 1–42 fibrils. On the other hand, the cat’s claw alkaloids isopteropodine, pteropodine, isomatraphyline and mitraphylline had no significant effects on disaggregation/reduction of pre-formed Aβ 1–42 fibrils. Bars represent mean +/− SEM. n = 5.

For the isolation of PTI-777 the methodology outlined in Table 1 was used. Under these conditions, PTI-777 separated into 11–13 major components (Fig. 6a), as revealed by uv/vis detection (diode array). These fractions were collected and designated as follows: fraction g (13–14 minutes), fraction f (15–16 minutes), fraction h (17–20 minutes), fraction i (21 minutes), fraction j (22–23 minutes), fraction k1 (24 minutes), fraction k2 (25 minutes), fraction l (26–27 minutes), fraction m (27–28 minutes), and fraction n (28–29 minutes) (Fig. 6a).

**Table 1:** PTI-777 isolation protocol. Step by step isolation protocol to isolate and identify the Aβ amyloid fibril inhibiting/reducing ingredients in PTI-00703 cat’s claw (*Uncaria tomentosa*) as shown in Fig. 6a.

PTI-777 Isolation Protocol (i.e. Beta-amyloid fibril inhibiting ingredients in *Uncaria tomentosa*)	
Step 1	1 kilogram of *Uncaria tomentosa* bark powder + 4000 ml methanol (mix)
Step 2	Centrifuge for 30 minutes at 2,500 × g
Step 3	Collect supernatant – repeat centrifugation and supernatant collection steps – 4 times
Step 4	Evaporate to dryness or until volume is 500 ml at 50 °C
Step 5	Wash with 300 ml of petroleum ether and discard ether layer (repeat 4 times)
Step 6	Evaporate to dryness at 50 °C (100 grams or ~10% of starting materials). Extract with 150 ml of distilled water, followed by centrifugation for 30 minutes at 2,500 × g – repeat 5 times.
Step 7	Lyophilize water extract (yield ~50 grams = ~5% of starting material)
Step 8	Dissolve 50 grams lyophilized water extract in 500 ml distilled water and apply 50–100 ml at a time on a 400 ml LH-20 equilibrated with distilled water
Step 9	Elute with 1100 ml of distilled water and discard
Step 10	Elute with 1100 ml of methanol and collect fractions (these fractions contain mostly f, g, m and n fractions) (yield ~6 grams = ~0.6% of starting material)
Step 11	Elute with 1100 ml of methanol and collect fractions (these fractions contain mostly h, j, k1, k2, l and other more hydrophobic fractions (yield ~6 grams = ~0.6% of starting material; these fractions are the most active against Aβ fibrils)
Step 12	Clean up fractions obtained from step 10 and 11 as follows: Dissolve in distilled water (80 mg/ml) and apply 5 ml at a time to a 10gram disposable C18 SPE column equilibrated in solvent A and washed with 3 volumes of solvent A and discard the eluate. Elute the clean fraction with 3 volumes of solvent A containing 12.5% solvent B. Lyophilize the corresponding fractions (~5 grams each obtained from steps 10 and 11). Where, solvent A = 95% water/5% acetonitrile/0.1% TFA, and solvent B = 955 acetonitrile/5% water/0.1% TFA.
Step 13	Fractionate fractions from step 12 (which consist of two separate fractions) on HPLC using 90 ml C18 reverse-phase HPLC column to isolate individual components.

Conditions**:** 50 mg/ml (in solvent A) injections; 40 times; 5 mls/min flow rate; collect 5 ml fraction every 1 minute. Gradient = 10% B 0–20 minutes, 10–100% B 20–30 minutes, 100–10% B 30–31 minutes; Run time = 35 minutes. Solvent A = 95% water/5% acetonitrile/0.1% TFA. Solvent B = 95% acetonitrile/5% water/0.1% TFA.

Retention Times: Fraction g (13–14 minutes); fraction f (15–16 minutes); fraction h (17–20 minutes); fraction i (21 minutes); fraction j (22–23 minutes); fraction k1 (24 minutes); fraction k2 (25 minutes); fraction l (26–27 minutes); fraction m (27–28 minutes); fraction n (28–29 minutes); fraction o (34–35 minutes).

### *In vitro* testing of individual fractions within PTI-777 for Aβ amyloid fibril inhibitory/reducing activity

The bioactivities of PTI-777 and its isolated individual fractions (i.e. fractions f through o) were evaluated in a number of different *in vitro* assays. Testing included the use of Thioflavin T fluorometry, Congo red staining assays, and negative stain EM. In most experiments, individual isolated fractions of PTI-777 were directly compared to (PTI-00703 cat’s claw, PTI-777 and the major oxindole alkaloids isolated from *Uncaria tomentosa* and thought to contain important bioactivity as described in 2 US Patents^36,37^ (Fig. 6b).

In one set of studies, Thioflavin T fluorometry compared the ability of PTI-00703 cat’s claw, PTI-777 (major components in cat’s claw), PTI-777 individual fractions (including fractions f, g, h, j, k, l, m and n), and alkaloids isolated from *Uncaria tomentosa* (including isopteropodine, pteropodine, isomitraphylline and mitraphylline), to disrupt/reduce pre-formed Aβ 1–42 fibrils. The results of 5 different Thioflavin T fluorometry experiments indicated that PTI-00703 cat’s claw caused a significant (p < 0.001) 53 +/− 2.5% disruption/reduction of pre-formed Aβ 1–42 fibrils (Fig. 6b). On the other hand, individual PTI-777 fractions including fraction f (64.0 +/− 1.7% inhibition), fraction g (62.3 +/− 8.5% inhibition), fraction h (which consisted of both h1 and h2; 56.3 +/− 2.1%), fraction j (68.7 +/− 2.0%), fraction k (which consisted of both k1 and k2; 58.0 +/− 4.6% inhibition), fraction l (68.3 +/− 2.3% inhibition), fraction m (64.0 +/− 1.5% inhibition), and fraction n (63.0 +/− 1.0% inhibition) were all similarly quite effective in causing a significant disruption/reduction of pre-formed Aβ 1–42 fibrils. Surprisingly, the oxindole alkaloids (isopteropodine, pteropodine, isomitraphylline, and mitraphylline) had no significant effects in disruption/disaggregation of pre-formed Aβ 1–42 fibrils (Fig. 6b).This study also showed that PTI-777 (i.e. mixture of fractions f through o) was a significantly more effective disrupter of Aβ of 1–42 fibrils (i.e. 87.3 +/− 3.0%) than any of the individual fractions tested. These studies indicated that PTI-777 and its individual fractions (fractions f, g, h, j, k, l, m, and n) were effective Aβ 1–42 fibril disaggregators and reducers. In addition, it was evident that the combination of fractions as observed with PTI-777 (a mixture of ~11–13 major components) were even more active than any of the individual PTI-777 fractions alone, suggesting a possible synergistic effect between different PTI-777 components. Lastly, the fact that isolated alkaloids from *Uncaria tomentosa* were basically ineffective in the disruption of Aβ 1–42 fibrils, suggested that oxindole alkaloids were not responsible for the Aβ amyloid inhibitory effects exerted by PTI-777, the individual PTI-777 fractions tested above, or PTI-00703 cat’s claw.

**Figure 7:**
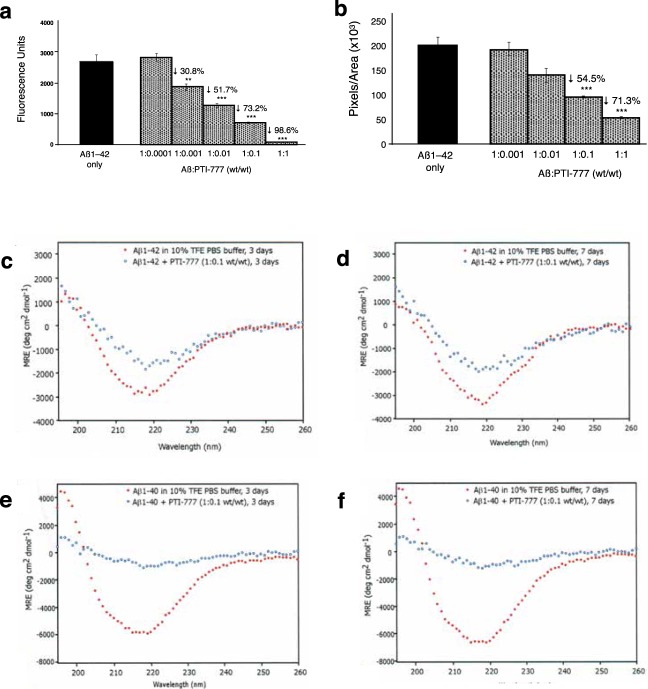
PTI-777 disrupts Aβ 1–42 fibrils. (**a**) Dose-dependent disruption/disaggregation of Aβ 1–42 fibrils by PTI-777 as assessed by Thioflavin T fluorometry. PTI-777 significantly (p < 0.001) disrupted/disaggregated Aβ 1–42 fibrils in a dose-dependent manner. **p < 0.01, ***p < 0.001, by Student’s t-test. Bars represent mean +/− SEM. n = 4. (**b**) Dose-dependent inhibition of Congo red binding to Aβ 1–42 fibrils by PTI-777. ***p < 0.001, by Student’s t-test. Bars represent mean +/− SEM. n = 4. (**c–f**) PTI-777 caused a marked disruption/disaggregation of β-sheet secondary structure for Aβ 1–42 fibrils, and prevented Aβ 1–40 fibril formation, as determined by CD spectroscopy. (**c**) PTI-777 at 3 days caused a decrease in β-sheet of Aβ 1–42 fibrils at a PTI-777:Aβ 1–42 (wt/wt) ratio of 0.1:1, as shown by a decrease in the in the ~218 nm minima curve. (**d**) By 7 days of co-incubation, PTI-777 caused an even greater disruption of the β-sheet secondary structure in Aβ 1–42 fibrils. (**e**) PTI-777 at 3 days completely prevented Aβ 1–40 fibril formation as demonstrated by a complete flattening of the characteristic β-sheet minima observed in Aβ 1–40 fibrils alone. (**f**) A similar inhibition of Aβ 1–40 β-sheet formation was observed with PTI-777 at 7 days.

In another study, Thioflavin T fluorometry determined the dose-dependent effectiveness of PTI-777 (i.e. Aβ amyloid inhibitory components present in PTI-00703 cat’s claw) in disrupting/reducing pre-formed Aβ 1–42 fibrils. For example, PTI-777 at an Aβ:PTI-777 wt/wt ratio of 1:0.1 significantly (p < 0.001) reduced Aβ 1–42 fibrils by 73.2%; whereas at an Aβ:PTI-777 wt/wt/ratio of 1:1 significantly (p < 0.001) reduced Aβ 1–42 fibrils by 98.6% (Fig. 7a).

A Congo red binding assay demonstrated similar results. As shown in Fig. 7b, PTI-777 effectively reduced pre-formed Aβ 1–42 fibrils in a dose-dependent manner. For example, PTI-777 significantly (p < 0.001) reduced Aβ 1–42 fibrils by 54.5% at a PTI-777:Aβ 1–42 wt/wt ratio of 1:0.1:, and by 71.3% (p < 0.001) at a PTI-777: Aβ wt/wt ratio of 1:1. A reduction of Congo red staining on glass slides also demonstrated a marked reduction in the red/green birefringence (not shown) indicative that PTI-777 significantly disrupts pre-formed Aβ -42 fibrils.

Circular dichroism (CD) spectroscopy confirmed that PTI-777 (i.e. Aβ amyloid inhibitory components present in PTI-00703 cat’s claw) not only inhibited Aβ 1–40 fibril formation, but also disrupted/reduced the β-sheet secondary folding of Aβ 1–42 fibrils. In one study, 50 µM of pre-formed Aβ 1–42 fibrils was incubated with PTI-777 at a PTI-777: Aβ 1–42 wt/wt ratio of 0.1:1. As shown in Fig. 7c, Aβ 1–42 (red closed circles) at 3 and 7 days demonstrated a characteristic pattern of extensive β-sheet secondary structure as shown by the curve and the minima at ~218 nm. In the presence of PTI-777 (blue closed circles) at both 3 days (Fig. 7c) and at 7 days (Fig. 7d) there was a marked disruption/reduction of pre-formed Aβ 1–42 fibrils. At 7 days, there was approximately 85–90% disruption/reduction of β-pleated sheet secondary folding by PTI-777, as shown by a flattening/smoothing of the curve especially at ~218 nm wavelength (Fig. 7d).

In another study, 50 µM of Aβ 1–40 was incubated at 37 °C for 1 week in phosphate-buffered saline (pH 7.4) either alone or in the presence of PTI-777 at a PTI-777: Aβ 1–40 wt/wt ratio of 0.1:1. PTI-777 caused a complete reduction of the β-sheet secondary folding curve (i.e. minima at 218 nm) within 3 days of co-incubation with Aβ 1–40 (Fig. 7e, blue closed circles). A similar inhibition of Aβ 1–40 fibril formation with PTI-777 was observed following 7 days of incubation (Fig. 7f, blue closed circles).

### Marked reduction of brain amyloid plaque load by PTI-777 (major Aβ amyloid inhibitory ingredients of PTI-00703 cat’s claw) in a transgenic mouse model of Alzheimer’s disease

**Figure 8:**
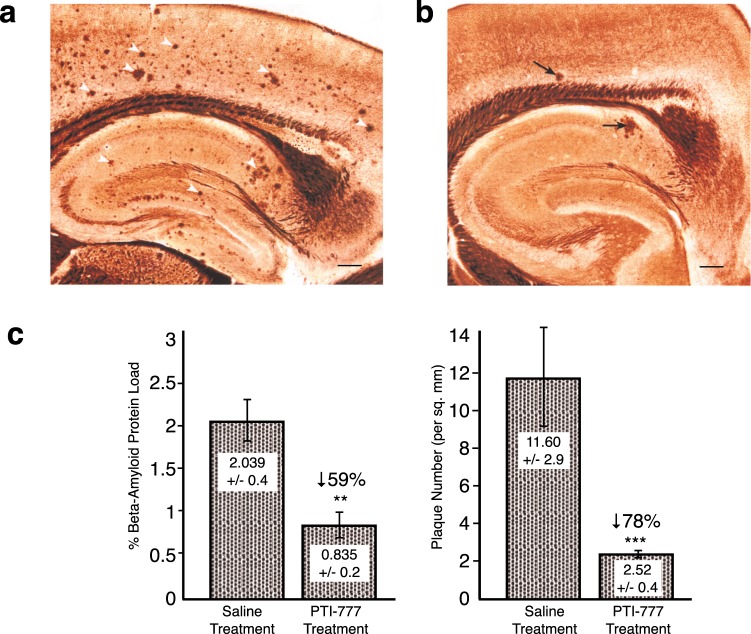
PTI-777 markedly reduced amyloid plaques in brains of 8-month old TASD-41 APP transgenic mice following direct infusion into hippocampus. (**a**) Bielchowsky silver stain showing abundant amyloid plaques in hippocampus and cortex of TASD-41 APP transgenic mouse infused with saline for 14 days. Scale bar = 100 µm. (**b**) PTI-777 treatment (100 µl at 8 mg/ml; 14-days infusion) demonstrated a marked reduction in amyloid plaques (arrows) in 8-month old TASD-41 APP transgenic mice. Scale bar = 100 µm. (**c, d**) Marked reduction in % beta-amyloid protein load and plaque number (per sq. mm.) following PTI-777 infusion. **p < 0.01; ***p < 0.001 by Student’s t-test. Bars represent mean +/− SEM. n = 4.

In the next set of studies, we determined whether PTI-777 may have direct effects on brain amyloid plaque load when directly infused into the cortex of 8-month TASD-41 APP double transgenic mice (i.e. London and Swedish mutations under Thy-1 promoter)^38^. As shown in Fig. 8a, representative Bielchowsky silver-stained sections of hippocampus and cortex from 8-month old plaque-producing transgenic mice are shown. Numerous amyloid plaques (white arrowheads) were observed. Following a 14-day treatment with PTI-777, there was a marked reduction in the number of amyloid plaques in hippocampus and cortex with only a few plaques remaining (Fig. 8b, arrows). Quantitation of % Aβ amyloid load and plaque number (per square mm) indicated that PTI-777 infusion significantly (p < 0.01) reduced % Aβ load by 59%, and plaque number (p < 0.001) by 78%, following only after 14-days of treatment with PTI-777 (Fig. 8c). The infusion site was not seen in these photomicrographs indicating that PTI-777 treatment was effective across the brain, even relatively far away from the infusion site.

### PTI-777 crosses the blood-brain-barrier and enters the brain parenchyma within 2 minutes of being in the blood

**Figure 9:**
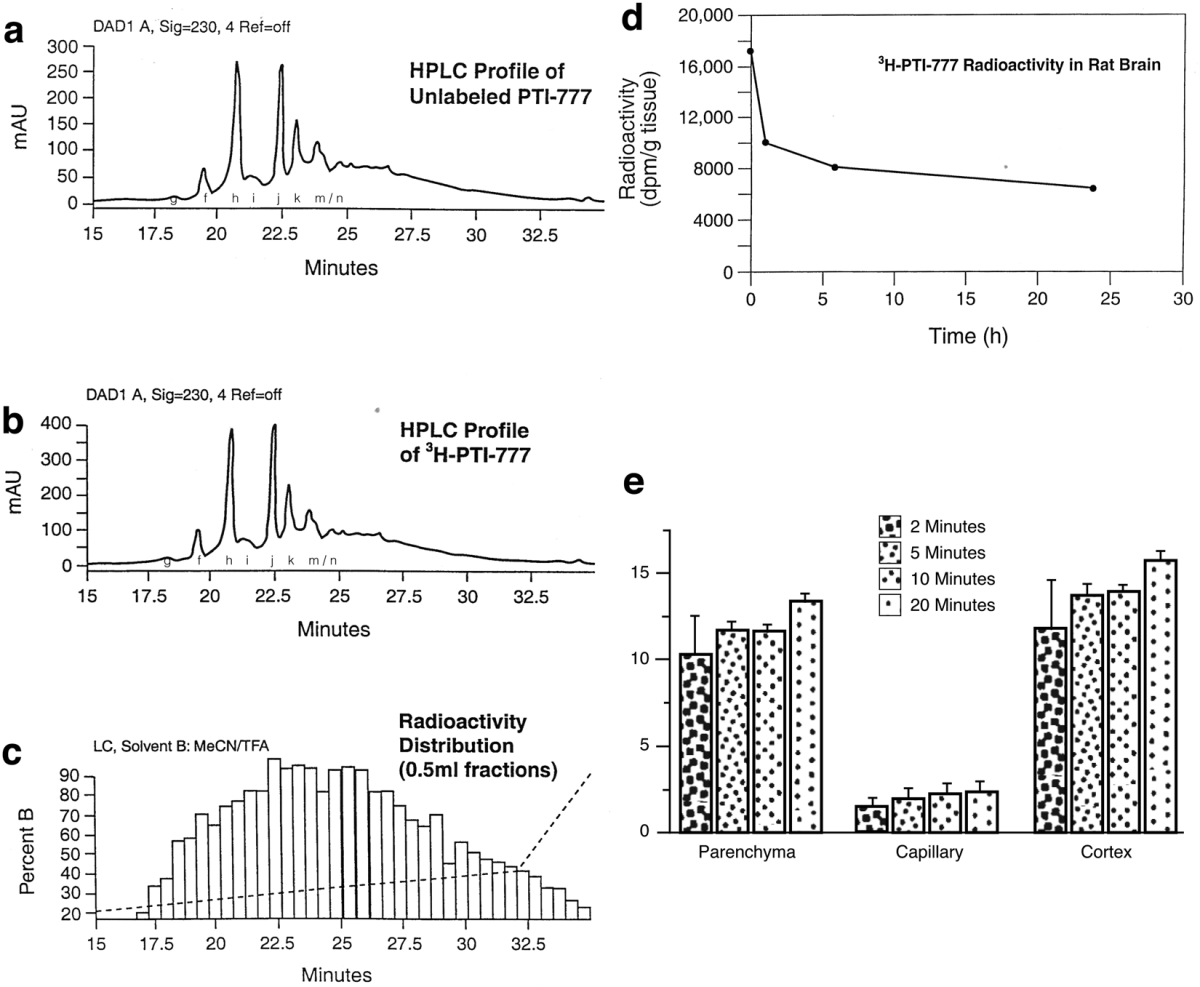
^3^H-PTI-777 crossed the blood-brain-barrier and entered the brain parenchyma within 2 minutes following peripheral administration. 0.9 mCi of ^3^H-PTI-777 with specific activity of 1.25 mCi/mg (at a concentration of 1 mCi/ml) was prepared and used for animal studies. (**a**) Figure demonstrates the HPLC profile of unlabeled PTI-777 which is nearly identical to the HPLC profile of (**b**) ^3^H-PTI-777. Fractions g, f, h, i, j, k and m/n are shown. (**c**) The distribution of radioactivity measured in 0.5 ml fractions that was eluted from the HPLC column and collected beginning at 16.5 minutes. This graph demonstrates that the HPLC peaks of PTI-777 also coincide with the greatest radioactivity. (**d**) Penetration of ^3^H-PTI-777 into rat brain, with radioactivity present in brain tissue in 2 minutes and lasted over a 24-hour period. n = 14 (7 females and 7 male Sprague-Dawley rats) (**e**) Penetration of ^3^H-PTI-777 into mouse brain tissue (CD-1 mice) within 2 minutes after being in the blood. n = 16. Bars represent mean +/− SEM.

The HPLC profile of unlabeled PTI-777 is shown in Fig. 9a, which was nearly identical to the HPLC profile of ^3^H-PTI-777 (Fig. 9b), indicating that the radiolabeling with tritium did little to alter the structure of PTI-777. The distribution of radioactivity measured in 0.5 ml fractions that were eluted from the HPLC column and collected beginning at 16.5 minutes is shown in Fig. 9c. This latter figure demonstrated that the HPLC peaks of PTI-777 also had the greatest radioactivity. In order to determine the potential ability of ^3^H-PTI-777 to cross the blood-brain-barrier and enter into brain tissue, male and female Sprague-Dawley rats were administered with a single i.v. injection of ^3^H-PTI-777. Two animals per group were then sacrificed at 2, 5, 10, 20 minutes, 1, 6 and 24 hours following administration, and the amount of ^3^H-PTI-777 in brain tissue was determined. As shown in Fig. 9d, ~18,000 dpm/g tissue of ^3^H-PTI-777 in brain was found within 5 minutes following intravenous administration. By 1 hour, this had decreased to ~10,100 dpm/g. Even at 24 hours, the brain tissue still contained about ~7,000 dpm/gram of ^3^H-PTI-777, indicating ~41% of the initial dose found to enter the brain parenchyma was retained in the brain tissue even over a 24-hour period.

In another study, groups of CD-1 mice (n = 4 per group) were injected intravenously with ^3^H-PTI-777 (25,000 cpm/µl) and ^99m^TC-albumin (200 µl of each). Following capillary depletion methods, a high brain/serum ratio of radiolabeled ^3^H-PTI-777 was present in brain parenchyma and cortex, with minimal amounts in the capillary fraction (Fig. 9e). Within 2 minutes of administration, ^3^H-PTI-777 (following capillary depletion methods) was found in brain parenchyma and cortex, and was maintained in brain tissue following 5, 10 and 20 minutes of administration (Fig. 9e). This data demonstrated that PTI-777 can cross the blood-brain-barrier and enter the brain parenchyma, even within 2 minutes of being in the blood.

### Marked reduction of brain plaque load by PTI-777 within 30 days of peripheral administration

**Figure 10:**
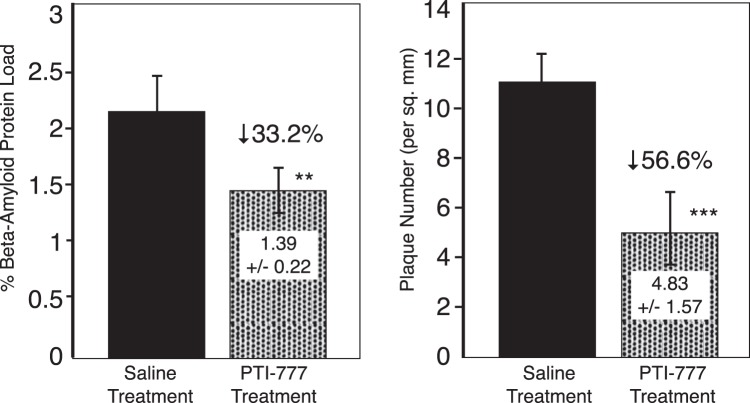
Marked reduction of brain plaque load in TASD-41 APP transgenic mice following 30-days of intraperitoneal injections of PTI-777. 6 month old TASD-41 APP transgenic mice were injected daily with saline or PTI-777. Within 30 days, PTI-777 reduced % Aβ plaque load by 33.2% (p < 0.05) and plaque number (per sq. mm) by 55.6% (p < 0.01). **p < 0.05; ***p < 0.001, by Student’s t-test. Bars represent mean +/− SEM. n = 8 per group.

PTI-777 (25 mg/kg) or saline was injected intraperitoneally daily for 30 days into 6-month old TASD-41 transgenic mice. Following the 30-day treatment, the results indicated that PTI-777 treatment caused a significant (p < 0.01) 33.2% reduction in % Aβ amyloid load, and a significant (p < 0.001) 56.6% reduction in amyloid plaque number (per square mm) (Fig. 10). These studies demonstrate the ability of the major components of *Uncaria tomentosa* (cat’s claw) to enter the brain and markedly reduce brain plaque load within 30 days following peripheral administration.

### Identification of the Major Components within PTI-00703 cat’s claw and PTI-777 responsible for the observed Aβ amyloid inhibitory activity

The preparative HPLC work on PTI-777 described here resulted in 11–13 major water-soluble active fractions, each of which contained primarily one major compound. Composition and structural studies demanded that the major individual compounds within each fraction to be purified to homogeneity in sufficient quantity for NMR and other spectroscopy studies (about 30–50 mg for each compound). Although each of the fractions of PTI-777 showed significant activity in the *in vitro* assays, fractions f, j, h1, h2, k1 and k2 were initially singled out for initial final purification based on their starting purity as determined by analytical HPLC, and the amount of material available. These fractions were passed through the preparative HPLC column until they were deemed pure.

#### PTI-777 fraction f identified as chlorogenic acid

**Figure 11:**
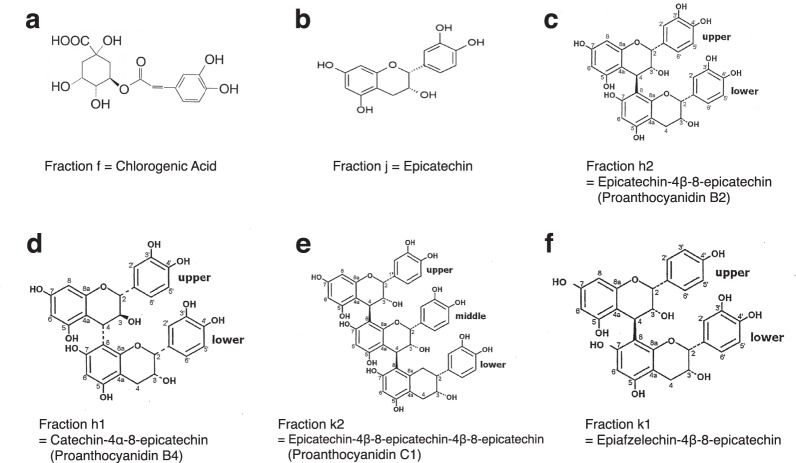
Purification and identity of the major components in PTI-777. (**a**) Structure of fraction f as chlorogenic acid (**b**) Structure of fraction j as epicatechin. (**c**) Structure of fraction h2 as epicatechin-4β-8-epicatechin (known as proanthocyanidin B2). (**d**) Structure of fraction h1 as catechin-4α-8-epicatechin (known as proanthocyanidin B4). (**e**) Structure of fraction k2 as epicatechin-4β-8-epicatechin-4β-8-epicatechin (known as proanthocyanidin C1). (**f**) Structure of fraction k1 as epiafzelechin-4β-8-epicatechin. See Supplement Results and Figures for structural elucidation and identification studies.

A variety of methods were used to isolate and identify the major compound within PTI-777 fraction f. HPLC, mass spectroscopy, NMR spectroscopy (^1^H-NMR and ^13^C-NMR), correlation spectroscopy (COSY) and ultraviolet (UV) spectroscopy were all used. As shown in Supplementary Material Fig. S1a,S1b, PTI-777 fraction f compound was identified as chlorogenic acid (C_16_H_18_O_9_; MW 354.31) with a structure as shown in Fig. 11a.

#### PTI-777 fraction j identified as epicatechin

Fraction j was the second material to be purified in a quantity sufficient for structural elucidation work. Following the isolation and purification of PTI-777-compound j, mass spectroscopy, NMR spectroscopy, correlation spectroscopy (COSY), and UV spectroscopy were all used on the fraction j compound and its pentaacetate derivative. As shown in Supplementary Material Fig. S2a–S2w, PTI-777 fraction j compound was identified as epicatechin (C_15_H_14_O_6_; MW 290.27), with a structure as shown in Fig. 11b.

#### PTI-777 fraction h2 identified as epicatechin-4β → 8-epicatechin (a specific epicatechin dimer known as proanthocyanidin B2)

Fraction h2 was the third material to be purified in a quantity sufficient for structural elucidation work. Following the isolation and purification of PTI-777-compound h2, HPLC, -ve ion electrospray mass spectroscopy, NMR spectroscopy (^1^H NMR and ^13^C NMR), correlation spectroscopy (COSY) and ultraviolet (UV) spectroscopy were all used. The main component of fraction h from the PTI-777 extract was also a major component in *Uncaria tomentosa* (cat’s claw) responsible for the Aβ (plaque) and tau protein (tangle) inhibitory and disaggregation activity (see Fig. 6a). As shown in Supplementary Material Fig. S3a–S3m, PTI-777 fraction h2 compound was identified as epicatechin-4β-8-epicatechin, also known as proanthocyanidin B2 (C_30_H_26_O_12_; MW 577), with a structure as shown in Fig. 11c.

#### PTI-777 fraction h1 identified as catechin-4α → 8-epicatechin (proanthocyanidin B4)

Fraction h1 was the fourth material to be purified in a quantity sufficient for structural elucidation work. Following the isolation and purification of PTI-777-compound h1, HPLC, -ve ion electrospray mass spectroscopy. NMR spectroscopy (^1^H-NMR and ^13^C-NMR), correlation spectroscopy (COSY), constant time inverse detection gradient accordion rescaled (CIGAR) spectroscopy, and ultraviolet (UV) spectroscopy were all used. As shown in Supplementary Material Fig. S4a–S4j, PTI-777 fraction h1 compound was identified as catechin-4α-8-epicatechin (Fig. 11d), also known as proanthocyanidin B4 (C_30_H_26_O_12_; MW 577).

#### PTI-777 fraction k2 identified as epicatechin-4β → 8-epicatechin-4β → 8-epicatechin or proanthocyanidin C1

Fraction k2 was the fifth material to be purified in a quantity sufficient for structural elucidation work. Following the isolation and purification of PTI-777-compound k2, HPLC, -ve ion electrospray mass spectroscopy. NMR spectroscopy (^1^H NMR and ^13^C NMR), correlation spectroscopy (COSY), constant time inverse detection gradient accordion rescaled (CIGAR) spectroscopy, and ultraviolet (UV) spectroscopy were all used. As shown in Supplementary Material Fig. S5a–S5j, PTI-777 fraction k2 compound was identified as epicatechin-4β → 8-epicatechin-4β → 8-epicatechin (Fig. 11e), also known as proanthocyanidin C1 (C_45_H_38_O_18_; MW 866).

#### PTI-777 fraction k1 identified as epiafzelechin-4β → 8-epicatechin

Fraction k1 was the sixth material to be purified in a quantity sufficient for structural elucidation work. Following the isolation and purification of PTI-777-compound k1, HPLC, -ve ion electrospray mass spectroscopy, NMR spectroscopy (^1^H NMR and ^13^C NMR), correlation spectroscopy (COSY), constant time inverse detection gradient accordion rescaled (CIGAR) spectroscopy, and ultraviolet (UV) spectroscopy were used. As shown in Supplementary Material Fig. S6a–S6j, PTI-777 fraction k1 compound was identified as epiafzelechin-4β → 8-epicatechin (Fig. 11f) (C_30_H_26_O_11_; MW 562).

#### PTI-777 fraction l identified as epicatechin-4β → 8-epicatechin-4β → 8-epicatechin-4β → 8- epicatechin (an epicatechin tetramer)

Fraction l was the seventh material to be purified in a quantity sufficient for structural elucidation work. Following the isolation and purification of PTI-777-compound l, HPLC, -ve ion electrospray mass spectroscopy, NMR spectroscopy (^1^H NMR and ^13^C NMR), correlation spectroscopy (COSY), constant time inverse detection gradient accordion rescaled (CIGAR) spectroscopy, and ultraviolet (UV) spectroscopy were used. As shown in Supplementary Material Fig. S7a, PTI-777 fraction l compound was identified epicatechin-4β → 8-epicatechin-4β → 8-epicatechin-4β → 8-epicatechin (an epicatechin tetramer) (C_80_H_50_O_24_; MW 1153).

### PTI-00703 cat’s claw proanthocyanidins are potent disaggregators/reducers of Aβ 1–42 fibrils

**Figure 12:**
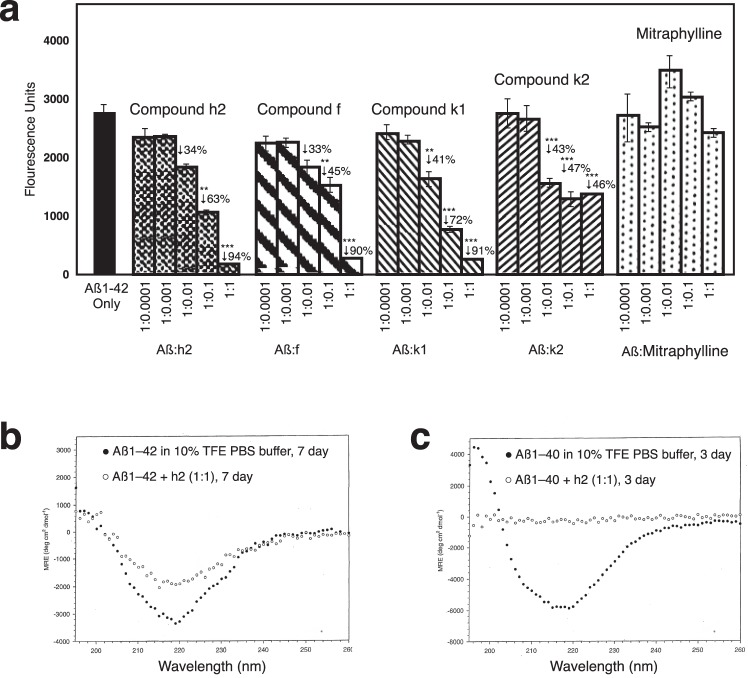
PTI-00703 cat’s claw proanthocyanidins are potent disrupters of Aβ 1–42 fibrils. (**a**) Disruption/disassembly of Aβ 1–42 fibrils by specific PTI-00703 cat’s claw proanthocyanidins. Compound h2 (epicatechin-4β → 8-epicatechin; proanthocyanidin B2) caused a dose-dependent disruption/disaggregation of pre-formed Aβ 1–42 fibrils. Similarly, compound f (chlorogenic acid), compound k1 (epicatechin trimer; proanthocyanidin C1) and compound k2 (epifzeledin-4β-8-epicatechin) all caused a marked disruption/reduction of preformed Aβ 1–42 fibrils when used at a 1:1 wt/wt ratio. The major cat’s claw alkaloid mitraphylline was found to be totally ineffective. **p < 0.01, ***p < 0.001, by Student’s t-test. Bars represent mean +/− SEM. n = 5. (**b**,**c**) Disruption/disaggregation of pre-formed Aβ 1–42 and 1–40 fibrils by epicatechin-4β-8-epicatechin (i.e. proanthocyanidin B2; compound h2) as assessed by CD spectroscopy.

In the next study, Thioflavin T fluorometry determined the effects of compounds h2, f, k1, k2 and mitraphylline (major alkaloid in cat’s claw) on disassembly/disaggregation of pre-formed Aβ 1–42 fibrils. In this study, 25 µM of pre-fibrillized Aβ 1–42 was incubated at 37 °C for 1 week alone or in the presence of compound h2, f, k1, k2 and mitraphylline at Aβ: test compound weight ratios of 1:1; 1:0.1; 1:0.01; 1:0.001 and 1:0.0001. The results of day 7 incubations are presented in Fig. 12a, but similar results were observed as early as 3 days (not shown). As shown in Fig. 12a, whereas the cat’s claw alkaloid mitraphylline caused no significant disruption of Aβ 1–42 fibrils at all concentrations tested, compound h2 (epicatechin-4β → 8-epicatechin; proanthocyanidin B2) caused a dose-dependent disruption/reduction of pre-formed Aβ 1–42 fibrils, with a significant (p < 0.001) 94% reduction when used at an Aβ:h2 wt/wt ratio of 1:1, and a significant (p < 0.01) 63% reduction when used at a Aβ:h2 wt/wt ratio of 1:0.1 (~1:1 molar ratio). Similarly, compound f (chlorogenic acid), compound k2 (epicatechin trimer; proanthocyanidin C1) and compound k1 (epiafzelechin -4β → 8-epicatechin) all caused a marked disruption/reduction of preformed Aβ 1–42 fibrils when used at a 1:1 wt/wt ratio (Fig. 12a). The most effective compound of these tested for disassembly/disaggregation of Aβ 1–42 fibrils was compound h2 (epicatechin-4β → 8-epicatechin; proanthocyanidin B2).

### PTI-00703 cat’s claw proanthocyanidin B2 (epicatechin-4β-8-epicatechin) is a potent disaggregator/reducer of pre-formed Aβ 1–42 and Aβ 1–40 fibrils as shown by circular dichroism spectroscopy

Circular dichroism (CD) spectroscopy determined the effects of test compounds to disrupt/reduce pre-formed amyloid fibrils. In one study, CD spectroscopy determined the effects of pure compound h2 (i.e. epicatechin-4β-8-epicatechin) on disruption of β-pleated sheet secondary structure of pre-formed Aβ 1–40 or Aβ 1–42 fibrils. As shown in Fig. 12b, Aβ 1–42 alone in 10% TFE buffer showed the typical CD spectra of an amyloid protein with significant β-structure, as demonstrated by the sharp minima observed at 218 nm. However, in the presence of the h2 compound (i.e. epicatechin-4β-8-epicatechin; proanthocyanidin B2) at a 1:1 molar ratio, a marked disruption of β-sheet structure in Aβ 1–42 fibrils was evident as shown by the flattening out of the minima observed at 218 nm (compared to Aβ 1–42 alone) (Fig. 12b). This was observed at both 3 days (not shown) and 7 days (Fig. 12b) following co-incubation of Aβ 1–42 fibrils with compound h2. This study clearly demonstrated that compound h2 (epicatechin-4β-8-epicatechin; proanthocyanidin B2) had the ability to disrupt/disassemble the beta-pleated sheet secondary structure characteristic of Aβ 1–42 fibrils.

As shown in Fig. 12c, Aβ 1–40 alone in 10% TFE PBS buffer also showed the typical CD spectra of an amyloid protein with significant β-sheet structure, as demonstrated by the sharp minima at 218 nm. However, in the presence of compound h2 (at a 1:1 molar ratio), a nearly complete disruption/disassembly of β-sheet structure in Aβ 1–40 fibrils was evident (with a significant increase in random coil or α-helix) as shown by the complete flattening out of the minima observed at 218 nm (Fig. 12c). This was observed at both 3 days (Fig. 12c) and 7 days (not shown) following co-incubation of Aβ 1–40 fibrils with compound h2. This study clearly demonstrated that proanthocyanidin B2 (i.e. compound h2) disrupted/disassembled the beta-pleated sheet secondary structure of Aβ 1–40 fibrils as well.

### Proanthocyanidin B2 (epicatechin-4β-8-epicatechin; compound h2) reduces brain plaque load and improves memory in a “plaque-producing” transgenic mouse model

**Figure 13:**
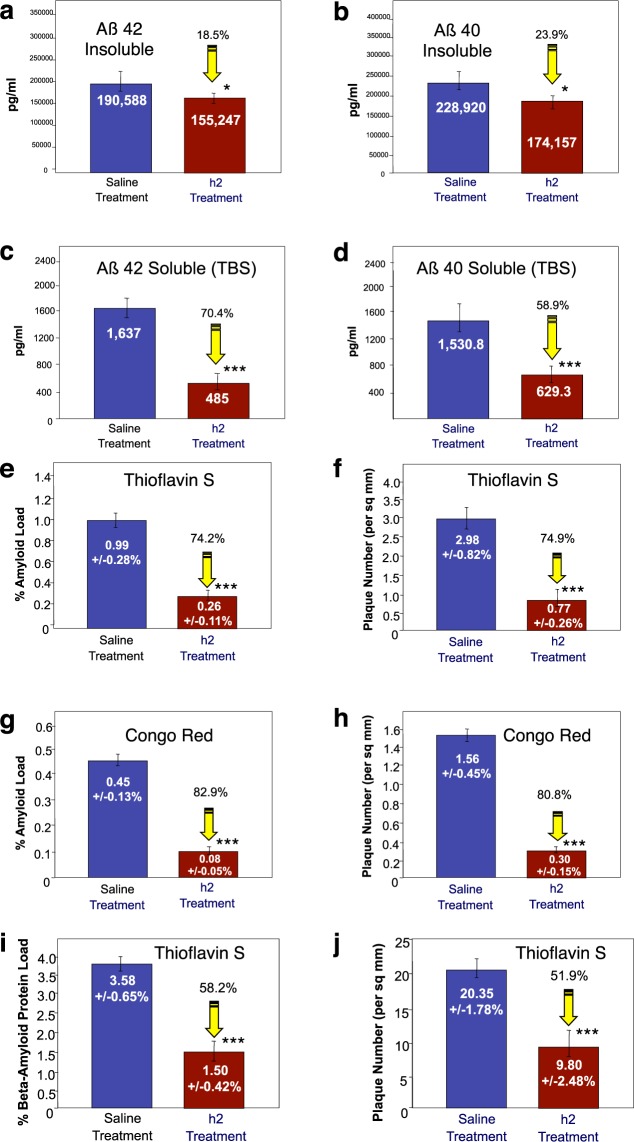
Reduction of brain plaque load in younger (**a–h**) and older (**i–j**) TASD-41 APP transgenic mice by proanthocyanidin B2 (compound h2). (**a–h**) Reduction of brain Aβ 1–42 and Aβ 1–40 load by proanthocyanidin B2 (i.e. compound h2) in younger (4 months old at start; 7 months old at sacrifice) APP transgenic mice. n = 10 per group. (**a**) Proanthocyanidin B2 caused a significant (p < 0.05) 18.5% reduction of insoluble Aβ 1–42, and (**c**) a significant (p < 0.05) 70.4% reduction of soluble Aβ 1–42, as determined by Aβ ELISAs. (**b**) Compound h2 also caused a significant 23.9% reduction of insoluble Aβ 1–40, and (**d**) a significant 58.9% reduction in soluble Aβ 1–40 in brains of TASD-41 transgenic mice. n = 4 per group. (**e–h**) Reduction of brain amyloid load and plaque number by proanthocyanidin B2 in younger (4 months old at start; 7 months old at sacrifice) TASD-41 transgenic mice. n = 10 per group. (**e**) Image analysis of Thioflavin-S stained sections from cortex revealed that proanthocyanidin B2 (h2 treatment) caused a significant 74.2% reduction in Thioflavin S amyloid load, and (**f**) a significant 74.9% reduction in plaque number (per sq. mm). Similarly, Congo red staining revealed a significant 82.9% in % amyloid load (**g**), and an 80.8% reduction in plaque number (**h**). Thioflavin S-stained sections from cortex of older TASD-41 APP transgenic mice (6 months old at start; 9 month old at sacrifice) revealed that proanthocyanidin B2 caused a significant 58.2% reduction in Thioflavin S amyloid load (**i**), and a significant 51.9% reduction in plaque number (**j**). *p < 0.05, **p < 0.01, ***p < 0.001, by Student’s t-test. Bars represent mean +/− SEM.

As shown in Fig. 13, brain (i.e. cortex) levels of Aβ 42 and Aβ 40 soluble and insoluble levels in younger 4-month old TASD-41 APP transgenic mice were significantly (p < 0.01) reduced following administration of proanthocyanidin B2 (compound h2; epicatechin-4β-8-epicatechin) at 50 mg/kg/day following 90-days of treatment (i.e. 7-months old at sacrifice) as determined using ELISAs for both Aβ 1–42 and 1–40. Proanthocyanidin B2 treatment significantly (p < 0.05) reduced brain Aβ 42 and Aβ 40 insoluble levels by 18.5% (Fig. 13a), and 23.9% (Fig. 13b), respectively. Proanthocyanidin B2 treatment also significantly (p < 0.01) reduced Aβ 42 and Aβ 40 soluble levels by 70.4% (Fig. 13c), and 58.9% (Fig. 13d), respectively, as well.

As shown in Fig. 13e, quantitative image analysis of Thioflavin-S stained sections from cortex of younger TASD-41 APP transgenic mice (i.e. 4 months of age to start; 7-months old at sacrifice) revealed that proanthocyanidin B2 treatment caused a significant (p < 0.001) 74.2% reduction in Thioflavin-S amyloid load (Fig. 13e), and a significant (p < 0.001) 74.9% reduction in plaque number (Fig. 13f) compared to saline-treated APP mice. Quantitative image analysis of Congo red stained sections demonstrated that proanthocyanidin B2 also caused a significant (p < 0.001) 82.9% reduction in Congo red stained amyloid load (Fig. 13g) and a significant (p < 0.001) 80.8% reduction in plaque number (Fig. 13h) compared to saline-treated APP mice.

A similar reduction in brain amyloid load and plaque number was also found after 90 days of peripheral treatment with proanthocyanidin B2 in older (9 months at sacrifice) TASD-41 APP transgenic mice. In this latter study, proanthocyanidin B2-treated animals displayed a significant (p < 0.001) 58.2% reduction in beta-amyloid protein load (as assessed using an Aβ 6E10 antibody) (Fig. 13i) and a significant (p < 0.001) 51.9% reduction in plaque number (Fig. 13j) compared to saline-treated mice.

**Figure 14:**
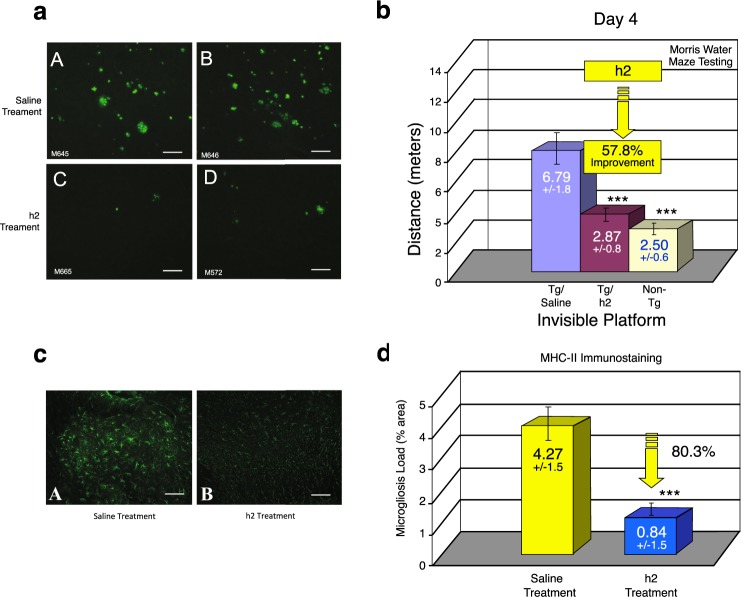
Improvement of memory and reduction of inflammation (astrocytosis and microgliosis) by proanthocyanidin B2. (**a**) Example of reduction of Thioflavin-S stained plaques in cortex of 2 saline-treated TASD-41 transgenic mice (Fig. a, upper panels) compared to two proanthocyanidin B2 (i.e. h2-treated TASD-41 transgenic mice (Fig. a, lower panels) Scale bar = 50 µm. (**b**) Morris water maze testing demonstrated that proanthocyanidin B2 treatment (Tg/h2) markedly and significantly (p < 0.001) improved short-term memory by 57.8% compared to saline-treated animals (Tg/Saline) as shown on day 4 of the invisible platform. A similar reduction was observed at day 5 of the invisible platform following procyanidin B2 treatment. p < 0.001 by student’s t-test and ANOVA. n = 12 per group. (**c**) Proanthocyanidin B2 was a potent reducer of astrocytosis in TASD-41 transgenic animals as demonstrated by a significant (p < 0.001) 69% reduction in astrocytosis (i.e. GFAP immunostained brain sections). Scale bar = 50 µm. (**d**) MHC-II immunostaining and image analysis also revealed that proanthocyanidin B2 treatment (significantly p < 0.001) reduced MHC-II (i.e. microgliosis) immunostaining by 80.3%. ***p < 0.001, by Student’s t-test. Bars represent mean +/− SEM.

Examples of 2 TASD-41 APP transgenic mice treated with saline for 90 days and stained for amyloid plaques in cortex using Thioflavin S is shown in Fig. 14a (a, b, saline treatment). Proanthocyanidin B2 treatment (h2 treated) caused a marked reduction of brain amyloid load/plaque number in cortex as shown in 2 different TASD-41 APP mice using Thioflavin S fluorescence (Fig. 14a, h2 treatment as shown in c & d).

Following 90 days of daily i.p. injections (50 mg/kg/day) with saline (Tg/saline) or proanthocyanidin h2 (Tg/h2), TASD-41 APP transgenic mice, and non-transgenic littermate controls (Non-Tg) were tested in a Morris water maze to determine effects on hippocampus-dependent memory (i.e. spatial acquisition). Proanthocyanidin h2 treatment caused marked improvements in hippocampus-dependent memory (by 57.8% on day 4 of the invisible platform; Fig. 14b) and by 57.3% on day 5 of the invisible platform (not shown). This was detected by both path length (distance in meters) (Fig. 14b) and in latency (m/sec)(not shown). No change in swimming speed (m/sec) between all groups were therefore found. Proanthocyanidin B2-treated TASD-41 APP mice had remarkable improvements in short-term memory approaching those levels observed in non-transgenic animals (Non-Tg) (Fig. 14b). On the last day of training, all groups found the visible platform demonstrating that all animals had no motor abnormalities. This study confirmed that the major component of PTI-00703 cat’s claw, proanthocyanidin B2, was a potent reducer of brain amyloid “plaques” that coincided with marked improvements in short-term memory.

### The major component of PTI-00703 cat’s claw (proanthocyanidin B2) is a potent reducer of both brain astrocytosis and microgliosis

Both GFAP immunostaining (for astrocytes) and MHC-II immunostaining (for microglia) from saline-treated versus proanthocyanidin B2-treated APP transgenic mice were quantified using an image analysis program. As shown in Fig. 14c, proanthocyanidin B2 treatment (h2 treatment) caused a significant reduction in astrocytosis as shown by a marked decrease in GFAP-immunostained brain sections. Quantitative image analysis indicated that compound h2 caused a significant (p < 0.001) 69.0% reduction in astrocytosis. As shown in Fig. 14d, proanthocyanidin B2 treatment also caused a significant (p < 0.001) 80.3% reduction in microgliosis as indicated by a marked reduction in MHC-II immunostaining in brain sections. These studies further verified the potent anti-inflammatory activity of proanthocyanidin B2 by markedly reducing both brain astrocytosis and microgliosis in TASD-41 APP transgenic mice. Cat’s claw has previously been shown to be a potent anti-inflammatory agent^39,40^.

## Discussion

The present investigation has led to the discovery that *Uncaria tomentosa* (and specifically PTI-00703 cat’s claw), an Amazon rain forest plant, is a natural and potent inhibitor and reducer of both brain “plaques and “tangles”. PTI-00703 cat’s claw and its ~11–13 major polyphenolic components (referred to as PTI-777) not only prevented Aβ 1–40 amyloid fibril and tau-protein paired helical filament/fibril formation, but also disrupted/disassembled and reduced pre-formed Aβ 1–42 fibrils (as found in brain “plaques”) and tau-protein paired helical filaments/fibrils (as found in brain “tangles”), nearly instantly. One major identified polyphenolic constituent of PTI-00703 cat’s claw was epicatechin-4β-8-epicatechin a specific epicatechin-dimer (known as proanthocyanidin B2) that potently reduced brain “plaque load” in TASD-41 APP transgenic mice. The reduction of brain “plaques” in APP transgenic mice by proanthocyanidin B2 correlated with marked improvements in short-term memory as demonstrated by Morris water maze testing.

PTI-00703 cat’s claw is a source of cat’s claw (i.e. *Uncaria tomentosa*) that comes from a specific commercial Peruvian manufacturer, where the extract is isolated from *Uncaria tomentosa* (i.e. cat’s claw) in a specific manner (described in methods section). It is important to note that there are approximately 34 species of cat’s claw^33,34^ and that each species may contain a set of different constituents within that are dependent on, a) the area the cat’s claw is grown, b) the season the cat’s claw is harvested, c) the extraction process to obtain the cat’s claw powder, d) whether the bark is obtained from the woody vine or root, and d) the specific species of cat’s claw (~34 known species of *Uncaria*)^33,34^.

Our studies elaborate on our initial investigation that first identified PTI-00703 cat’s claw as a potential inhibitor/disaggregator of Aβ fibril formation using x-ray diffraction methods. In this study, x-ray diffraction techniques established the inhibitory effects of PTI-00703 cat’s claw, in comparison to several different small molecules on structural inhibition/disassembly of pre-formed Aβ fibrils^41^. Of all the compounds tested, PTI-00703 cat’s claw was by far the most effective inhibitor/disaggregator of pre-formed Aβ fibrils as shown by x-ray diffraction analysis^41^. PTI-00703 cat’s claw abolished the H-bonding reflection indicative of beta-sheet secondary structure present in Aβ fibrils^41^. This mechanism is further confirmed in the present study by PTI-00703 cat’s claw’s ability to rapidly reduce Aβ fibrils into mostly amorphous non-fibrillar material and causes a marked decrease in the secondary folding of both Aβ fibrils and tau protein tangles as determined by circular dichroism spectroscopy.

PTI-00703 cat’s claw, PTI-777 or proanthocyanidin B2 (compound h2), resulted in Aβ 1–42 or Aβ 1–40 fibrils forming soluble, mostly amorphous material that is believed to be non-toxic to cells. *In vivo* studies following injection of PTI-777 into brain, or peripheral administration of proanthocyanidin B2 (i.e. compound h2), demonstrated no neuronal loss or apparent cellular toxicity in brain following up to 6 months of treatment. In addition, proanthocyanidin B2 treatment caused marked memory improvements in TASD-41 APP transgenic mice, which would not occur if PTI-777 or the proanthocyanidin B2 component was toxic to neurons or other cells in the brain. This is similar to the lack of neuronal toxicity observed with the polyphenol, epigallocatechin gallate (ECGC) whereby incubation with Aβ 1–42 fibrils results in off-pathway, highly stable oligomers that are non-toxic to cells^42^. The current investigation also suggests that it is the specific small molecule polyphenolic and proanthocyanidins compounds present in *Uncaria tomentosa* (and not the alkaloid compounds) that are responsible for the observed Aβ 1–40 and 1–42 inhibitory and reducing effects.

### What is *Uncaria tomentosa* (cat’s claw)?

Most commercial preparations of cat’s claw consist of the plant species *Uncaria tomentosa*, although there are 34 other known species of cat’s claw other than *Uncaria tomentosa*^33,34^. Cat’s claw is found in nature in two different chemotypes producing different alkaloidal constituents. Pentacyclic oxindoles are found in the roots of one type, whereas the tetracyclic oxindoles are present in the second type^43^. Uncarine C and Uncarine E are two stereoisomers of the pentacyclic oxindoles^44^. Other alkaloids of the tetracyclic oxindoles found in cat’s claw include mitraphylline, rhynchophylline and isorhychophylline^45^ that we tested and were found not to have any inhibitory or disaggregation effects on Aβ fibrils.

Besides the presence of alkaloids, *Uncaria tomentosa* has been found to contain other phytochemicals including quinic acid^46,47^, quinovic acid glycosides^48^, other low molecular weight polyphenols^49^, ursolic acid, oleanolic acid^50^, beta-sitosterol, stigmasterol, campesterol^51^, the three polyhydroxylated triterpenes^52^. The C-8-(S) isomer of deoxyoganic acid (7-deoxyoganic acid), together with beta-sitosteryl glucoside, five known stereoisomeric pentacyclic oxindole alkaloids, and the tetracyclic oxindole isorhyncophylline, have also been isolated from the inner bark of *Uncaria tomentosa*^53,54^. In addition, rotundifoline and isorotundifoline^55^, quinovic acid 1–7, flavonoids and coumarins^51,56^ also have been isolated and identified from *Uncaria tomentosa*.

### Effects of PTI-00703 cat’s claw on “tangles”

Our studies identified the inhibitory effects of PTI-00703 cat’s claw and its small molecule natural polyphenolic and proanthocyanidin components on tau-protein containing “tangles”. Using the human tau 4-repeat domain, we demonstrated by electron microscopy that we could produce paired helical and straight filaments *in vitro* following induction with heparin^35,57–61^. In the present investigation, PTI-00703 cat’s claw was identified as a potent inhibitor/reducer of tau protein containing paired helical filaments and fibrils, in addition to its inhibitory effects on Aβ “plaques”. This agrees with grape-seed extract polyphenols (primarily catechin and epicatechin) that reduced paired helical filaments isolated from Alzheimer’s disease brain^62^ and reduced tau pathology in JNPL3 transgenic mice after 6 months of oral administration^63^. Further studies are needed to determine the effects of PTI-00703 cat’s claw and its proanthocyanidin constituents on reduction of tau protein “tangles” *in vivo* (i.e. in tau transgenic mice). However, this is the first study to demonstrate that a form of cat’s claw (i.e. PTI-00703 cat’s claw) has the ability to inhibit and disrupt both “Aβ plaques” and “tau protein tangles” *in vitro*, as well as *in vivo* (in plaque-producing TASD-41 APP transgenic mice), which are important findings.

### Proanthocyanidin B2 (epicatechin-4β-8-epicatechin) is a major component of PTI-00703 cat’s claw responsible for potent Aβ fibril inhibitory and dissolving activity

A newly identified component present in PTI-00703 cat’s claw responsible for the potent Aβ fibril disaggregation/dissolving activity is epicatechin-4β-8-epicatechin, a specific epicatechin dimer known as proanthocyanidin B2. Thioflavin T fluorometry, CD spectroscopy and EM all demonstrated the marked ability of proanthocyanidin B2 to inhibit the formation (i.e. Aβ 1–40) of Aβ fibrils, as well as disrupt/disassemble and reduce pre-formed Aβ 1–42 fibrils. CD spectroscopy also revealed a marked reduction in the β-sheet secondary folding of Aβ fibrils. Similar results were observed by electron microscopy whereby the disaggregation of Aβ 1–42 fibrils into mostly amorphous non-fibrillar material were visualized. We believe this is the first study identifying proanthocyanidin B2 as a major component of *Uncaria tomentosa* (cat’s claw) that is a potent reducer/dissolver of Aβ fibrils. Other proanthocyanidins identified in the current investigation within cat’s claw that also demonstrated good Aβ inhibitory activity included proanthocyanidin B4 and proanthyocyanidin C1. The major alkaloids found within *Uncaria tomentosa* (cat’s claw) including isopteropodine, pteropodine, isomitraphylline and mitraphylline in a number of different experiments had no significant effects on disruption/disassembly of pre-formed Aβ fibrils.

### Major PTI-00703 cat’s claw components cross the blood-brain-barrier and enter the brain parenchyma

Our studies demonstrated that the major Aβ amyloid inhibitory ingredients of PTI-00703 cat’s claw (i.e. PTI-777) can enter the brain parenchyma within 2 minutes of being in the blood. This finding agrees with previous published studies in which epicatechin and its metabolites entered the brain parenchyma following oral administration for 1, 5 and 10 days^64^. In another study, proanthocyanidins and their metabolites entered the brain following oral administration and improved cognitive function^65^. Furthermore, rats fed a blackberry proanthocyanidin diet (*Rubus fruiticosus*) had proanthocyanidins and their metabolites in brain tissue following oral administration^66^. Proanthocyanidins from grapes were also found to enter the brain within minutes following oral administration^67^. All of these studies correlate with our findings that specific polyhydroxylatic aromatic compounds and proanthocyanidins (within cat’s claw) can enter the brain parenchyma within minutes following peripheral administration.

### Proanthocyanidin B2 markedly reduces brain plaque load and improves short-term memory in Alzheimer’s transgenic mice

Proanthocyanidin B2 markedly reduced Aβ plaque load and plaque number in younger (i.e. 7 months old at sacrifice) TASD-41 APP transgenic mice. A similar reduction was observed in older (i.e. 9-months old at sacrifice) TASD-41 APP transgenic animals treated with proanthocyanidin B2 suggesting that this small molecule polyphenolic compound within cats’ claw can reverse much of the Aβ plaque load already present, even in older APP transgenic animals with an enhanced brain plaque load.

### Other polyhydroxylated aromatic compounds are potent inhibitors/disrupters of brain “plaques” and “tangles” associated with brain aging and Alzheimer’s disease

Several different studies have now identified specific polyhydroxylated aromatic containing compounds within natural products to be inhibitors/reducer of brain “plaques” associated with brain aging and Alzheimer’s disease. For example, epicatechin (a major component we identified in cat’s claw, which is also present in green tea) reduced amyloid plaque load in brains of APP/PS1 transgenic mice after oral administration^68^. In another study, grape seed extract containing a mixture of gallic acid, catechin and epicatechin were orally fed a diet that reduced brain amyloid plaques by 49%, and microgliosis by 70%^69^. The polyphenols found in red wine grapes (in Cabernet Sauvignon) also attenuated Aβ neuropathology in the Tg2576 mouse model of Alzheimer’s disease when delivered orally for 7 months^70^. In this latter study, a decrease in brain amyloid plaque load also coincided with improvements in memory. Wang *et al*.^65^ also demonstrated that specific monomeric proanthocyanidins found in grape seed extract (i.e. catechin and epicatechin) given orally to Tg2576 mice significantly lowered Aβ 1–42 levels, as well as improved the cognitive behavioral performance. Studies indicated that small molecule proanthocyanidins do cross the blood-brain-barrier and enter the brain parenchyma. These studies agree with the findings of the present investigation.

Several different studies demonstrate that other polyhydroxylated aromatic compounds can also reduce brain “plaque” and/or “tangle” load in brains of transgenic animals. In early studies, curcumin^71–76^, green tea polyphenols^42,77^ such as epigallocatechin gallate (EGCG)^42,78–80^ and catechins^81^, resveratrol^82–86^ and other enriched polyphenol extracts^87^ had effects in reducing Aβ “plaque” and/or tau protein “tangle” pathology *in vitro* and/or *in vivo*.

### Identification of specific phenolic motifs in proanthocyanidin B2 in PTI-00703 cat’s claw

**Figure 15:**
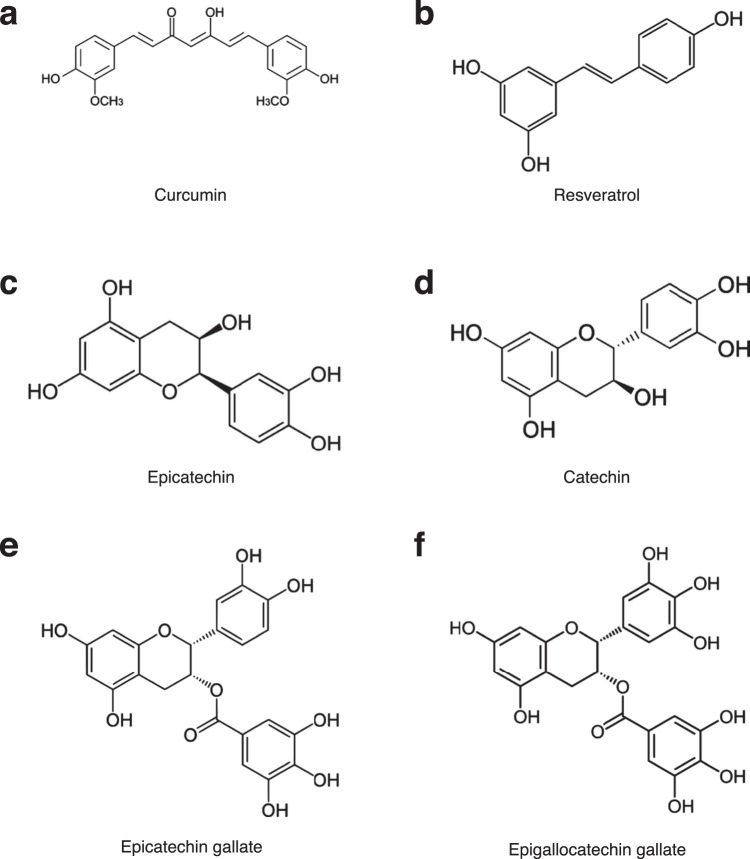
Importance of specific hydroxyl groups on polyphenolic compounds reported as inhibitors of Aβ amyloid fibrillogenesis. (**a**) Curcumin has a hydroxyl (OH) group next to a methoxy group on is aromatic rings. (**b**) Resveratrol has no adjacent hydroxyl groups. (**c**) Epicatechin has adjacent hydroxyl groups on one of its aromatic rings. (**d**) Catechin also has adjacent hydroxyl groups on one of its aromatic rings. (**e**) Epicatechin gallate (ECG) has aromatic rings with two and three adjacent hydroxyl groups on one of its aromatic rings. (**f**) Epigallocatechin gallate (EGCG) as two aromatic rings each containing three adjacent hydroxyl groups.

Comparison of structures of postulated effective molecules previously identified to be inhibitor/disrupters of Aβ-containing “plaques” and/or tau protein containing “tangles” include: 1) curcumin (Fig. 15a); 2) resveratrol (Fig. 15b); and components of tea (*Camellia sinensis*) including, 3) epicatechin (Fig. 15c), 4) catechin (Fig. 15d), 5) epicatechin gallate (ECG) (Fig. 15e), and 6) epigallocatechin gallate (ECGC) (Fig. 15f). Note that the “common motif structure consist of aromatic compounds containing hydroxyl groups. For example, curcumin (Fig. 15a) contains an aromatic ring with a hydroxyl (“OH”) group next to a methoxy group (“OCH_3_”); whereas resveratrol (Fig. 13b) contains aromatic rings with hydroxyl groups that are non-adjacent to each other.

The most effective and potent “plaque” and/or “tangle” inhibitors and disrupters consist of aromatic polyhydroxylated compounds appear to contain “two or more adjacent” hydroxyl groups” (i.e. catechol groups), such as found in epicatechin, catechin, ECG and ECGC (Fig. 13). The present study confirms this concept in that all the compounds we identified as potent “plaque and tangle” inhibitors within PTI-00703 cat’s claw (Fig. 10), all contain polyhydroxylated aromatic compounds with one or more catechol groups. Whereas chlorogenic acid and epicatechin contain one catechol group, the most effective major component identified in PTI-00703 cat’s claw was the specific dimeric epicatechin, proanthocyanidin B2 (Fig. 10C). We believe the position and spacing of the two catechol groups is important for “plaque” and “tangle” inhibitory and dissolving activity. A replacement of the any catechol groups on the aromatic rings with methyl groups leads to nearly complete abolishment of amyloid inhibitory activity (Snow *et al*., unpublished data). We believe that the activity has nothing to do with “anti-oxidant” potential, suggesting no reported correlation of *in vitro* inhibitory IC_50_ values with Aβ fibrils, and anti-oxidative features of various polyphenols tested^88^.

Porat *et al*.^88^ suggested that, the inhibition mechanism of amyloid fibril formation by small polyphenolic compounds require a) specific structural conformation necessary for specific β-sheet interaction and stabilization of the inhibitor-protein complex, and b) aromatic interaction between the phenolic compound in the inhibitor molecule and aromatic residues (like phenylalanine at residues 4; 19 and 20 of Aβ 1–42) in the amyloidogenic sequence that may direct the inhibitor to the amyloidogenic core and facilitate binding interaction, but also interfere with fibril assembly.

We postulate that PTI-00703 cat’s claw is a superior natural product to be effective against both Aβ plaques and tau protein tangles, especially in comparison to other polyphenols that are lacking a catechol group (i.e. two adjacent hydroxyl groups on an aromatic ring), such as resveratrol and curcumin (Fig. 15). It is also noteworthy to point out that *Uncaria tomentosa* (cat’s claw) has a multitude of polyphenolic constituents, each containing aromatic rings with two adjacent hydroxyl groups. This includes chlorogenic acid, epicatechin, proanthocyanidins B2, B4 and C1, and epicatechin tetramers, all identified in the present investigation. Therefore, PTI-00703 cat’s claw is postulated to have a superior inhibitory and reducing activity on both “plaques and tangles” due to most of its major constituents containing aromatic rings that contain dihydroxyl groups.

### *Uncaria tomentosa* (cat’s claw) is also a natural potent anti-inflammatory agent

Previous studies demonstrated that cat’s claw has the natural and innate properties of being a potent anti-inflammatory agent. Cat’s claw extract inhibits the production of tumor necrosis factor-α, an inflammatory messenger that sets the stage for acute and chronic inflammation^89^. It also inhibits the activation of NFK-β^40,89^. In a human trial, cat’s claw offered relief for adults suffering from osteoarthritis, with those receiving a cat’s claw preparation reporting significant reductions in pain associated with physical activity^89^.

In our hands, PTI-00703 cat’s claw can markedly reduce tumor necrosis factor (TNF-α) and interleukin 1, two important inflammatory cytokines (Snow *et al*., unpublished data). In fact, reduction of brain astrocytosis (Fig. 14c) and microgliosis (Fig. 14d) was noted in our animal studies following administration of proanthocyanidin B2 (epicatechin dimer), in addition to its concurrent reduction of brain “plaque” load and improved memory in APP transgenic mice.

### Isolation of PTI-00703 cat’s claw compared to other cat’s claw products in the marketplace today

PTI-00703 cat’s claw represents a 70% ethanol/water extract of *Uncaria tomentosa* bark powder that is filtered (to remove high molecular weight material) and finally concentrated by spray drying. We have tested at least 10 different sources of *Uncaria tomentosa* for specific Aβ 1–42 fibril and “plaque- dissolving” activity from different companies across Europe, Brazil, Peru, and the USA. We have identified one Peruvian source (referred to as “PTI-00703 cat’s claw”) that produces an *Uncaria tomentosa* extract (dry bark powder) to contain the most robust “plaque” dissolving activity compared to other cat’s claw sources tested (Snow *et al*., unpublished data).

### Memory enhancing effects of cat’s claw in human clinical trials

There are now a number of published human clinical trials that suggest the potent memory and cognition enhancing effects of cat’s claw, usually in a mixture with other nutraceutical ingredients. In one study, a dietary supplement containing ~25% cat’s claw bark powder, was tested in a randomized, double-blind placebo-controlled study^90^. The results demonstrated that this product with cat’s claw significantly improved short-term memory (i.e. delayed verbal recall) and executive function in normal individuals aged 18–35 years old, following only 6-weeks of oral treatment^90^.

In another study, a Chinese Herbal Medicine known as “Choto-san” that contains ~20% cat’s claw bark powder (from *Uncaria seninsis*) improved cognitive function and activities of daily living of patients with dementia in a double-blind, randomized, placebo controlled study^91^. Choto-san in a kampo formulation of 11 herbs with *Uncaria seninsis* (note this is a different species of cat’s claw) recognized as the most important. Comparisons of the cat’s claw containing Choto-san was made to another Japanese traditional medicine known as “Gosyajink-gan”. After 8 weeks of oral administration, the mini-mental state examination scores increased significantly in the cat’s claw containing Choto-san group (from 15.5 points to 17.5 points out of 30, 95% confidence interval), but not in the “Gosyajink-gan” or placebo groups.

### Percepta: A new plant-based natural supplement targeting brain “plaques & tangles”

A new product called Percepta is being sold in the USA and is the only brain health supplement on the market today that contains “PTI-00703 cat’s claw”. The Percepta product contains PTI-00703 cat’s claw in combination with a specific oolong tea extract. Only these two major ingredients caused potent “plaque and tangle” inhibitory and dissolution activity in a number of pre-clinical studies (Snow, unpublished studies). The results of numerous pre-clinical studies using Percepta demonstrates that it has potential to be the first nutraceutical developed to specific target and reduce/prevent brain “plaques and tangles”. Further *in vitro* and human clinical trials are postulated to confirm the efficacy of such a promising new nutraceutical. Initial pre-clinical studies suggest it has superior effects to hitting “plaques and tangles” in comparison to other leading brain health/memory supplements sold in the marketplace today (Snow *et al*., unpublished studies). The present investigation has presented a good case for including PTI-00703 cat’s claw as a major ingredient for any brain aging and/or memory supplement due to its inherent and natural ability to target the accumulation of brain “plaques and tangles”.

## Methods

### PTI-00703 cat’s claw

PTI-00703 cat’s claw represents a dried extract of cat’s claw (*Uncaria tomentosa*) extracted by a specific manufacturing company source in Peru. The general extraction process to obtain PTI-00703 cat’s claw required use of 70% ethanol/distilled water at a ratio of 5:1. The grinded cat’s claw bark was put into tanks with dynamic maceration for 3 days before separating the liquid from the herb in an Alfa-Laval decanter. The cat’s claw product was then filtered, and the liquid was concentrated to recover the solvent and the tincture was concentrated to a proper amount of brix to facilitate the drying process. The concentration of the liquid was implemented by a falling film evaporator with the temperature kept under 70 °C and a small amount of vacuum ensured that the temperature was controlled. The tincture was then spray dried by a hot gas. The temperature used an inlet temperature of 180 °C and an outlet temperature of 70 °C. The resulting powder was then homogenized and packaged in 5 kilogram polyethylene double bags, ready for testing.

### Isolation of the active ingredients within PTI-00703 cat’s claw responsible for Aβ fibril inhibitory and reducing activity

Assay-guided affinity fractionation and high-pressure liquid chromatography (HPLC) separated and purified the major Aβ fibril inhibitory and reducing active components present in PTI-00703 cat’s claw. Initial studies utilized water extracts of *Uncaria tomentosa* (cat’s claw) applied to different Affi-Prep 10 gel columns derivitized with Tris-HCl, ammonia or ethanolamine. Methanol, ethanol or acetonitrile effectively eluted the *Uncaria tomentosa* active components from the column. A Tris-HCl derivatized column was most effective in binding the Aβ fibril inhibitory and reducing components of *Uncaria tomentosa* which suggested that the active components may have affinity for both hydroxyl (due to the Tris) and hydrophobic (due to the resin used) groups. In order to scale-up separation of the Aβ fibril inhibitory and reducing components within *Uncaria tomentosa*, a Tris-acrylate column was prepared by rinsing 25 ml of Affi-Prep 10 gel with distilled water and incubating with 100 ml Tris-HCl (1 M, pH 8.0). The resulting material was packed into a 20 ml column (MT20, Biorad), attached to an 1100 series Hewlett Packard HPLC with a diode array detector. The column was equilibrated at a flow rate of 0.5 ml/min with water. A water soluble extract prepared from 400 mg of lyophilized PTI-00703 cat’s claw in 2 ml of distilled water was injected onto the Tris-derivatized Affi-gel 10 column and eluted using the following profile: 0–10 minutes 100% distilled water, 10–100 minutes, 0–100% acetonitrile, and 100–110 minutes, 100% acetonitrile. Fractions were collected every 4 minutes. Aliquots from fractions 1–22 (i.e. 4 mins to 84 mins) were then incubated with fibrillar Aβ 1–40 or Aβ 1–42 for 2 hours at a wt/wt/ratio of 1:1 and tested for their ability to disrupt/disassemble preformed Aβ fibrils using Thioflavin T fluorometry as previously described^35^. Fractions 13–18 (i.e. 52–72 mins) demonstrated the greatest ability (from 60–75%) to disrupt/disassemble pre-formed Aβ 1–40 fibrils, as indicated by a marked lowering of Thioflavin T fluorescence. These fractions were collectively designated as PTI-777 (fractions 13–18). PTI-777 was later efficiently enriched and scaled-up using LH 20 columns for individual fraction/compound identification.

### Isolation of major water-soluble Aβ amyloid inhibitory ingredients within *Uncaria* tomentosa (referred to as PTI-777)

The HPLC profile of PTI-777 indicated at 11 distinct fractions. Preparative HPLC was used to further fractionate PTI-777. For these studies, 50 mg of PTI-777 (prepared as described above) was injected into a Hewlett-Packard 1100 Series HPLC instrument with diode array detector, fitted with a 2.2 cm × 25 cm Vydac 218TP1022 C18 reverse-phase column maintained at 25 °C. The sample was eluted at a flow rate of 5 ml/min as follows: 10% B for 0–20 mins., 10–100% B gradient for 20–30 mins., and 100–10% B gradient from 30–31 mins. where A = 95% water/5% acetonitrile/0.1% TFA, and B = 95% acetonitrile/5% water/0.1% TFA. Under these conditions, PTI-777 separated into 11 major components as revealed by uv/vis detection (diode array). Fractions containing 11 components were collected and designated as follows: fraction g (14–15 mins), fraction f (17–18 mins), fraction h (19–20 mins), fraction i (21 mins), fraction j (22–23 mins), fraction k1 (24 mins), fraction k2 (25 mins), fraction l (26–27 mins), fraction m (29 mins), fraction n (30 mins) and fraction o (34 mins). The activity of each of these fractions were also tested for their ability to disrupt/disassemble pre-formed Aβ 1–40 or Aβ 1–42 fibrils as previously described^35^.

### Scale Up Preparation of PTI-777 and Individual Fractions for Compound Identification

For scale-up preparation of each of the fractions and components of PTI-777 (1 gm 5–10 ml in solvent A) were injected onto a 4.14 cm × 25 cm Varian Dynamax C-18 reverse phase column (336 ml bed volume) fitted to a Varian Prostar 215 solvent delivery system and a Varian model 320 UV-Vis detector. The separation was carried out at ambient temperature, at a flow rate of 50 ml/min and UV detection at 230 nm. The solvent gradient profile was as follows: 0–4 min, 25% B; 4–11 min, 25–30% B; 11–14 min, 30–90% B; 14–17 min, 90% B; and 17–19 min, 90-25% B; where A was distilled water with 0.1% TFA and B was methanol with 0.1% TFA. Based on our work, these modified HPLC conditions also resulted in the separation and purification of the 11 major fractions (g-o, including k1 and k2), previously isolated from PTI-777. The fractions obtained under these procedures were correlated with the original fractions by HPLC under conditions described above.

Final purification of PTI-777 individual components required additional HPLC to separate each of the major compounds within each fraction, from any minor components that me be present. The major components of each fraction (which usually represented 90% of the material) were isolated by the pooling (and drying) of fractions comprised of a single major peak when viewed at 210 nm on HPLC. The resulting pure material was used for *in vitro*/*in vivo* testing and structural elucidation studies as described below.

To assess the purity of individual major PTI-777 components, analytical HPLC with diode array detector (Agilent 1100 series) was used. In addition to observing a single peak by UV chromatogram, the diode array detection allowed is to ensure that the tailing end of each peak has the same spectra (superimposable) as to the middle and the leading edge of the peaks, indicating purity. The collected compounds were then further analyzed by mass spectroscopy and nuclear magnetic resonance (NMR) spectroscopy (see supplemental information).

### Identification of Individual Components within PTI-777

A variety of methods were implemented to identify the major compounds within the major fractions of PTI-777 (i.e. Aβ amyloid inhibiting components within cat’s claw). These latter studies included the use of HPLC, -ve ion electrospray mass spectroscopy (relative intensity of the molecular ion given as a percentage), fourier transfer mass spectroscopy, ultraviolet spectroscopy, ^1^H NMR, ^13^C NMR, electrospray ionization time-of-flight mass spectroscopy (ESI-TOF), electron impact (EI) initiated mass spectroscopy, fast atom bombardment (FAB) mass spectroscopy, homonuclear correlation spectroscopy (COSY), constant time inverse-detection gradient accordion rescaled heteronuclear multiple bond correlation spectroscopy (CIGAR) and heteronuclear correlation spectroscopy (HETCOR).

All solvents were distilled before use and were removed by rotary evaporation at temperatures up to 20 °C. Octadecyl functionalized silica gel (C18) was used for reverse-phase (RP) flash chromatography, and Merck silica gel 60, 200–400 mesh, 40–63 µm, was used for silica gel flash chromatography. Thin layer chromatography was carried out using Merck DC-plastikfolien Kieselgel 60 F_254_, first visualized with a UV lamp, and then by dipping in 5% aqueous ferric chloride solution. Optical rotations were measured on a Perkin-Elmer 241 polarimeter.

The analytical HPLC equipment consisted of a Waters 717 autosampler, 600 pump and controller, and a 2487 UV detector controlled by Omega software. Samples were analyzed by using an RP-18 semi-preparative column (Phenomenex Jupiter 5 µm C18 300 A, 250 × 10 mm) with a guard column (Phenomenex Security Guard cartridge containing a C18 ODS 4 × 3 mm, 5 µm column) fitted at 30 °C. Samples (5 µl) were analyzed using a mobile phase flow rate of 5.0 mL/min, with UV detection at 280 nm. Solvent A = CH_3_CN containing 0.1% TFA. Solvent B = H_2_O containing 0.1% TFA.

Mass, UV, and IR spectra were recorded on an electrospray time-off-flight (ESI-TOF) mass spectrometer, Kratos MS-80, Shimadzu UV 240, and Perkin-Elmer 1600 FTIR instruments, respectively. NMR spectra, at 25 °C, were recorded at 500 or 300 MHz for ^1^H and 125 or 75 MHz for ^13^C on Varian INOVA-500 or VXR-300 spectrometers. The data was analysed using the software provided with each instrument. Chemical shifts are given in ppm on the δ scale referenced to the solvent peak CH_3_OH at 3.30, **C**D_3_OD at 49.3 ppm, CHCl_3_ at 7.25, CDCl_3_ at 77.0; (CH_3_)_2_CO at 2.15 and (CD_3_)_2_CO at 30.5.

Identification of the major components within each fraction of PTI-777 relied on structural assignments of both ^1^H and ^13^C NMR spectra in comparison with published spectra (Aldrich Library of ^1^H NMR and ^13^NMR Spectra) and those reported in the literature^92,93^. Furthermore, comparison of the spectral data of peracetylated derivatives were also compared to those in the literature^93^.

### Thioflavin T fluorometry studies

The effects of PTI-00703 cat’ claw, PTI-777 (amyloid inhibitory components of cat’s claw) and individual cat’s claw components (i.e. fractions and compounds) on inhibiting Aβ 1–40 fibril formation and disrupting/reducing pre-formed Aβ 1–42 fibrils, was determined using a previously described method of Thioflavin T fluorometry^94–97^. Using this sensitive assay, any decreases or increases in fluorescence correlated with a decrease or increase in the amount of amyloid fibrils present^94–97^, allowing one to determine the extent of potential inhibitors and/or enhancers of Aβ amyloid fibril formation.

For these studies, 22 µM of Aβ 1–40 or Aβ 1–42 (rPeptide, Watkinsville, GA, USA) was incubated in 96-well assay plates at 37 °C for 1-week (in triplicate) either alone or in the presence of increasing amounts of PTI-00703 cat’s claw, PTI-777 or individual components (i.e. fractions and compounds). Aliquots were taken and usually analyzed at 0, 1, 3 and 7 days of incubation. Following the incubation period, 240 µl of Aβ 1–40 or Aβ 1–42 +/− increasing concentrations of PTI-00703, PTI-777 or individual components were added to 60 µl of 500 µM Thioflavin T (Sigma Chemical Co., St. Louis, MO) in 50 mM NaPO_4._ Studies indicated that increasing concentrations of Aβ gave a proportional increase in fluorescence in the presence of 500 µM Thioflavin T, ruling out the presence of any disproportionate inner filter effect in these studies. Fluorescence emission at 485 nm was measured on a Molecular Devices instrument model SpectraMAX GeminiXS fluorometer, at an excitation wavelength of 450 nm. For each determination, the fluorometer was calibrated by zeroing in the presence of the Thioflavin T reagent alone. All fluorescent determinations were based on these references and any fluorescence given off by any of the compounds in the presence of the Thioflavin T reagent was always subtracted from all pertinent readings.

### Congo red staining

The presence of Aβ amyloid fibrils was assessed by Congo red staining as viewed under polarized light^98^. Briefly, 15ul aliquots of incubated solutions (i.e. Aβ 1–40, Aβ 1–42 +/− PTI-00703 cat’s claw, PTI-777 and/or individual components) were reacted in solution with 5ul of a 0.5% Congo red aqueous solution for 10 minutes. The samples were then micro-centrifuged for 2 minutes at high speed. Next 10ul of the Congo red supernatant was pipetted off from the sample tubes, and 10 ul of double-distilled water was added. The samples were gently mixed and micro-centrifuged for 2 minutes, and 15 ul of the Congo red supernatant was pipetted from each tube. The remaining material was well mixed and applied to slide wells (PTFE printed slides, EMS sciences). 3 ul of Vector Mount (Vector Labs) was then added to the wells and cover slipped with 5 mm Phototech glass coverslips. The samples were viewed under polarized light and a prominent red/green birefringence was indicative of Aβ amyloid fibrils. A reduction or loss of red/green birefringence staining was indicative of disaggregation/dissolution of Aβ fibrils.

### Thioflavin S Fluorescence

The presence of Aβ amyloid fibrils was also assessed by Thioflavin S fluorescence as viewed under fluorescence light^99^. Briefly, 10–20ul aliquots of incubated solutions (i.e. Aβ 1–40, Aβ 1–42 +/− PTI-00703 cat’s claw, PTI-777 and/or individual components) were placed on PTFE printed slides (EMS Sciences) and allowed to dry at room temperature overnight. Next, 10 ml of Thioflavin S (0.5% Fisher) solution was pipetted onto the dried samples in the wells and reacted for one minute. Then 10 ml of Thioflavin S solution was gently removed from the sample wells. Lastly, 3 ml of Vector Mount (Vector Labs) was added to the wells and cover slipped with 5 mm Phototech glass coverslips. The samples were viewed under fluorescent light and a prominent green fluorescence was indicative of Aβ amyloid fibrils present. A reduction and loss of the green fluorescence was indicative of disaggregation/dissolution of Aβ fibrils.

Thioflavin S fluorescence was also used to detect Aβ amyloid plaques in the brains of APP transgenic mice as previously described^100^. Positive green fluorescent plaques were obvious following staining of brain tissue sections and viewing under fluorescent light.

### Congo red binding

In this assay, the ability of PTI-777 (major Aβ amyloid inhibitory components present in PTI-00703 cat’s claw) to alter Aβ binding to Congo red was quantified. Aβ 1–42 +/− PTI-777 was incubated for 3 days and then vacuumed through a 0.2 µm filter. The amount of Aβ 1–42 retained in the filter (i.e. retentate) was then quantified following staining of the filter with Congo red. After appropriate washing of the filter, any lowering of the Congo red color on the filter in the presence of PTI-777 (compared to the Congo red staining of the amyloid protein in the absence of the test compound) was indicative of PTI-777’s ability to diminish/alter the amount of aggregated and congophilic Aβ.

### Circular dichroism spectroscopy

Circular dichroism (CD) spectroscopy was used to evaluate the effects of extracts and individual compounds on disaggregation/disruption of β-sheet secondary structure of Aβ 1–40 and 1–42 fibrils, and tau protein fibrils (induced by heparin). For these studies, 50 µM of Aβ 1–40, Aβ 1–42, or tau protein (+heparin), was incubated at 37 °C for 1 week in phosphate-buffered saline (pH 7.4) either alone, or in the presence of PTI-00703 cat’s claw, PTI-777 or individual cat’s claw components at increasing doses to Aβ 1–40, Aβ 1–42, or tau protein (+heparin) on a weight-to-weight basis. CD spectra were collected at 25 °C on an AVIV CD Spectrometer 62DS. Measurements were carried out in a 0.5 mm path length quartz cuvette, over the range of 190–260 nm. The instrument was calibrated with an aqueous solution of (+)-10-camphorsulfonic acid. CD spectra consisted of an average of a series of scans made at 0.5 nm intervals.

### Negative Stain Electron Microscopy

For negative stain electron microscopy (EM), 50 µM Aβ 1–40, 1–42 fibrils, or tau protein fibrils (induced by heparin)^35,37,57–61^ was incubated at 37 °C for 7 days in the absence or presence of PTI-00703 cat’s claw, at increasing weight-to-weight ratios. Aliquots were taken at 1, 3, and 7 days for electron microscopy analysis to observe any time-dependent effects on Aβ fibrils and tau protein fibrils. Negatively-stained Aβ amyloid and tau protein fibrils were prepared by floating piloform, carbon-coated grids on peptide solutions. After the grids were blotted and air-dried, the samples were stained with 2% (w/v) uranyl acetate and visualized with a JOEL 1400 Transmission electron microscope and digitally imaged using Gatan digital camera and software.

### Effects of PTI-777 on amyloid plaque load in a transgenic mouse model of Alzheimer’s disease

In these studies, the effects of PTI-777 on reduction of brain amyloid plaque load was evaluated in Alzheimer’s transgenic mice over-expressing human APP-751 cDNA containing the London (V717I) and Swedish (K670M/N671L) mutations under regulatory control of the Thy-1 promoter (i.e. TASD-41 mice)^38^. Two groups (n = 6 per group) of 6–8 month old plaque-producing TASD-41 transgenic mice were anesthetized with pentobarbital (50 mg/kg) and a 27 gauge stainless steel cannula was stereotaxically placed into cortex using bregma as reference point (stereotaxic co-ordinates of AP -1.7; ML 3.0; DV -3.0) and secured to the skull by machine screws and dental acrylic. The cannula was connected via a 5 cm coil tubing to a model 2002 osmotic minipump (Alzet Inc.) placed subcutaneously between the shoulder blades. The transgenic mice were infused for 2 weeks (using Alzet osmotic pumps) with saline or PTI-777. 100 µl of saline or PTI-777 (8 mg/ml) per animals was used for infusion directly into cortex of APP transgenic animals (i.e. ~800 µl of PTI-777 delivered into each animal). Bielchowsky silver staining and Aβ immunostaining were used to demonstrate and quantitate amyloid plaque load.

### Labelling of PTI-777 with ^3^H

In order to determine whether PTI-777 and/or its components have the capacity to cross the blood-brain-barrier and enter the brain, PTI-777 had to be effectively radiolabeled without affecting its structure. Due to its highly electron rich structure due to the presence of the OH groups such as those found in chlorogenic acid (i.e. fraction f) and epicatechin (fraction j), PTI-777 was initially labeled with tritium using proprietary atom bombardment technologies (Moravek Inc., Brea, CA). 0.9 mCi of ^3^H-PTI-777 with specific activity of 1.25 mCi/mg (at a concentration of 1 mCi/ml) was prepared and used for animal studies.

### ^3^H-PTI-777 blood-brain-barrier studies in rats and mice

Groups of male and female Sprague Dawley rats (n = 7 per group), and CD-1 mice (n = 4 per group) determined the ability of ^3^H-PTI-777 to cross the blood-brain-barrier and enter the brain parenchyma. Following capillary depletion methods^101,102^, groups of CD-1 mice (n = 4 per group; 16 total) were injected intravenously with ^3^H-PTI-777 (25,000 cpm/µl) and ^99m^TC-albumin (200 µl of each). The brain/serum ration (in µl/gram) was determined in capillaries, cortex and parenchyma at 2, 5, 10 and 20 minutes following administration as previously described^101,102^.

### Intraperitoneal injections of PTI-777 into brain plaque-producing transgenic mice

In another animal study, the effects of PTI-777 on reduction of brain amyloid plaque load was determined following daily intraperitoneal (i.p.) injections. PTI-777 (25 mg/kg) or saline (n = 12 per group) was injected daily for 30 days into 6 month-old TASD-41 transgenic mice.

### Peripheral Administration of proanthocyanidin B2 (epicatechin-4β-epicatechin) in plaque-producing mouse models of brain aging and Alzheimer’s disease

Transgenic mice expressing human APP-751 cDNA under regulatory control of the Thy-1 promoter were used. Two groups (n = 10 per group) of 4-month old APP transgenic mice were injected daily with proanthocyanidin B2 (fraction h2 isolated as described above) or saline (i.p. injections, 50 mg/kg/day) for 90 days (until they reached 7 months of age). In another study to test the effects of proanthocyanidin B2 (h2 compound) on older APP transgenic mice, two groups (n = 10 per group) of 6-month old APP transgenic mice were injected daily with proanthocyanidin B2 (h2 compound) or saline for 90 days (until the animals reached 9 months of age). Quantitative analysis of brain sections included use of Aβ antibodies (i.e. 6E10), amyloid stains (Thioflavin S, Congo red) and glial markers (MHC-II for microgliosis; GFAP for astrocytosis).

### Preparation of Mouse Brains for Immunostaining

At the appropriate time points, mice were sacrificed by deep anesthesia and exsanguination. The brain was carefully extracted, bisected along the midline, and half the brains were flash frozen with the other half submerged in 20 ml of fresh 4% paraformaldehyde (PFA) in PBS at 4 °C. The next morning, the PFA was replaced with PBS and the brains were stored at 4 °C before cutting on the vibratome (Leica Biosystems). 40 µM sections of mouse brains were cut and sections were floated off the specimen into PBS and picked up and moved to multi-well plates with a paintbrush.

Sections were stained in 24 or 12 well multi-well plates. Non-specific binding was first blocked by incubation in a solution of 5–10% normal goat serum (or appropriate species serum based on secondary) in PBS in 0.1–0.3% TX100 for 1 hour at room temperature with gentle shaking. The next morning sections were washed in PBS 3 times for 5 minutes each time with gentle shaking. Sections were again washed in PBS 3 times for 5 minutes each time with gentle shaking. Sections were carefully mounted onto charged slides (Fisher) using a paintbrush and cover slipped for viewing.

### Antibodies

Antibodies used included 6E10 for Aβ amyloid plaques; Glial fibrillar acidic protein (GFAP) for astrocytes (Dako) and MHC-II (Dako) for microglia.

### Brain Plaque Load Quantitation

The % beta-amyloid protein load and plaque number (per sq mm) was determined by Aβ immunostaining and quantitative analysis as previously described^103^.

### Morris Water Maze Testing

Morris water maze testing determined the effects of proanthocyanidin B2 (h2 compound) on hippocampus-dependent memory (i.e. spatial acquisition) as previously described^38^. For the latter study, following 90 days of treatment with saline or proanthocyanidin B2, groups of APP transgenic mice and non-transgenic littermate controls (n = 12 per group) were tested in a Morris water maze to determine effects of proanthocyanidin B2 on hippocampus-dependent memory. Following 3 days of training (3 trials per days with the visible platform), the invisible platform testing (3 trials per day) were implemented for 5 days, and the time to reach the platform (latency/secs), path length (meters) and swim speed (m/sec) were recorded with an imaging tracking system. On the last day, the animals were shown the platform (i.e. visible platform) to ensure that there were no motor abnormalities in all animals tested.

### Aβ ELISAs

Aβ 1–42 and 1–40 level s were determined using the Life Technologies detection kit following the specified instructions. Protein samples were diluted 1:50 in standard diluent buffer to ensure that the levels of SDS were compatible with the ELISA assay kit. Samples were then compared to a standard curve, and Aβ (1–42 and 1–40) levels were compared to a standard curve and concentrations of each were established as per manufacturing recommendations.

### Animal Studies

All murine studies involving use of stereotaxic apparatus, use of osmotic pumps, peripheral administration of PTI-777 and its components, proanthocyanidin B2 (h2 compound) and behavioral testing were approved by the Institutional Animal Care and Use Committee (IACUC) at the University of California–San Diego (Laboratories of EM) and performed in accordance with approved Animal Care and Use Protocols (ACUPs). All rodent and murine studies involved use of radiolabeled PTI-777 for blood-brain-barrier penetration were approved by the Institutional Animal Care and Use Committee (IAUCAC) at the Pennington Biomedical Research Center at Louisiana State University, Baton Rouge, LO (labs of AK and WP).

### Statistical Analysis

For all fibrillogenesis studies utilizing Thioflavin T fluorometry, comparisons of Aβ or tau protein, in the presence or absence of test compounds were based on paired Student’s t tests with data shown as mean +/− SEM. Significance was reported at the 95% (p < 0.05), 99% (p < 0.01) and 99.999% (p < 0.001) confidence levels.

## Supplementary information

Supplementary Information
