# Supplementary material for: The Amazon rain forest plant Uncaria tomentosa (cat’s claw) and its specific proanthocyanidin constituents are potent inhibitors and reducers of both brain plaques and tangles
Source: Sci Rep. 2019 Feb 6;9:561. doi: 10.1038/s41598-019-38645-0 (PMC6365538; doi:10.1038/s41598-019-38645-0)
Supplement: Supplementary file 1 — Supplementary Information [file 41598_2019_38645_MOESM1_ESM.pdf]

Supplementary (S) Information:

The Amazon rain forest plant *Uncaria tomentosa* (cat's claw) and its proanthocyanidin constituents are potent inhibitors and reducers of brain plaques and tangles

Alan D Snow*[1,2,10], Gerardo M Castillo[2,10], Beth P Nguyen[2], Paula Y Choi[2,10], Joel A Cummings[1,2,10], Judy Cam[2], Qubai Hu[2], Thomas Lake[1, 2, 10], Weihong Pan[3], Abba Kastin[3], Daniel A. Kirchner[4], Steven G Wood[5], Edward Rockenstein[6], Eliezer Masliah[6], Stephen Lorimer[7], Rudolph E Tanzi[8] and Lesley Larsen[7,9,10]

[1]Cognitive Clarity Inc., Edmonds, WA, USA. [2]ProteoTech Inc., Kirkland, WA, USA. [3]Blood-Brain Barrier Laboratory, Pennington Biomedical Research Center at Louisiana State University, Baton Rouge, Louisiana, USA. [4]Department of Biology, Boston College, Chestnut Hill, MA, USA. [5]Department of Chemistry and Biochemistry, Brigham Young University, Provo, UT, USA. [6]Departments of Neurosciences and Pathology, University of California- San Diego, La Jolla, CA, USA. [7]Department of Chemistry, University of Otago, Dunedin, New Zealand. [8]Genetics and Aging Research Unit, Department of Neurology, Massachusetts General Hospital and Harvard Medical School, Charlestown, MA, USA. [9]Deceased. [10]These authors contributed equally to this work.

*Correspondence and requests for materials should be addressed to Dr. Alan D. Snow (email: dralansnow@cognitiveclarityinc.com)

Figure Legends:

Figure S1. PTI-777 fractions f identified as chlorogenic acid.

Figure S2. PTI-777 fraction j identified as epicatechin.

Figure S3. PTI-777 fraction h2 identified as epicatechin-4β-8-epicatechin (proanthocyanidin B2).

Figure S4. PTI-777 fraction h1 identified as catechin-4α-8-epicatechin (proanthocyanidin B4).

Figure S5. PTI-777 fraction k2 identified as epicatechin-4β-8-epicatechin-4β-8-epicatechin (proanthocyanidin B4).

Figure S6. PTI-777 fraction k1 identified as epiafzelechin-4β-8-epicatechin.
compound k1 peracetate.

Figure S7. PTI-777 fraction l identified as epicatechin-4β-8-epicatechin-4β-8-epicatechin-epicatechin-4β-8-epicatechin-4β-8-epicatechin (epicatechin tetramer).

SUPPLEMENTARY METHODS AND RESULTS:

a) PTI-777 fraction f identified as chlorogenic acid

Fraction F was the first material to be purified in quantity sufficient for structural elucidation work. Mass spectroscopy and NMR spectroscopy analysis was initially employed. Several different types of mass spectra ([(chemical ionization (CI), fast atom bombardment (FAB), and electron impact (EI)] were taken of the purified samples.

The ^{1}H-NMR (500 MHz) of fraction f in pyridine (d_5) showed 12 signals (Fig. **S1a**). A broad signal at about 8.4 ppm was attributed to OH groups on the compound. Two strongly coupled signals at 8.0 and 6.8 ppm and three aromatic signals at 7.5, 7.16 and 7.07 ppm were present in the spectrum indicative of hydrogen bound to sp^2 hybridized carbon atoms. There were also signals at 6.2, 4.75, 4.3, 2.9, 2.72 and 2.7 ppm. All of these signals with the exception of ones at 8.4 and 2.72ppm showed integration consistent with one proton. The integration of the 2.72 ppm signal was closer to 2 protons and the OH signal at 8.4ppm was not integrated. The Correlation Spectroscopy (COSY) spectrum (a two dimensional NMR experiment identifying adjacent protons) showed that the protons responsible for the signals at 8.0 and 6.8ppm were adjacent to each other. The large coupling constant of these two signals as well as their chemical shifts were indicative that these protons were attached to a carbon-carbon double bond system with trans geometry coupled to an aromatic ring. The COSY spectrum also revealed that the three aromatic protons were all on the same benzene ring and that the remaining six signals all showed connectivity indicating five contiguous carbon atoms.

The ^{13}C NMR (500 MHz) in pyridine (d_5) showed 16 discrete signal regions (Fig. **S1b).** The two signals at 177 and 167 ppm indicated the presence of two carbonyl C atoms. There were 8 carbons found in the

shift range for sp^2 carbons (signals at 150, 147, 146, 127, 122, 117, 116 and 115 ppm). There were four signals (76, 74, 72 and 71 ppm) indicative of sp^3 carbons bonded to oxygen atoms. Three of these signals were doubled (74, 72 and 71 ppm). The two signals at 39.7 and 39.2 ppm were representative of sp^3 carbon atoms not bonded to oxygen. The six doubled signals were assumed to be due to a mixture of isomers. The Distortionless Enhancement by Polarization Transfer (DEPT) experiment (distinguishes between carbon atoms bonded to 1, 2, 3 or no H atoms) indicated that the aromatic carbon atoms showed signals at 150, 147 and 127 ppm were not bonded to H. This was also the case for the atom responsible for the signal at 76 ppm. The Heteronuclear Correlation Spectroscopy (HETCOR) experiment helped confirm which ^1H signals were associated with individual ^{13}C signals.

The diode array ultraviolet (UV) spectrum of fraction f showed a peak at 33nm indicating a possible presence of an aromatic ring with extended conjugation. Analysis of both the ^1H and ^{13}C NMR data pointed to the presence of a tri-substituted aromatic ring with two phenolic groups and a conjugated ethylene group with trans geometry. The chemical shift of the carbonyl carbon at 177 ppm, suggested that this signal was due to a carboxylic acid group attached to this conjugated system. The COSY data suggested a chain of five continguous carbon atoms, three of which were oxygenated. Attachment of the remaining sp^3 quarternary carbon atom (76 ppm) to both ends of the five-carbon atom chain would form a cyclohexane ring, which would be consistent with the data. The remaining carbonyl carbon atom was assigned as a carboxylic acid group attached to the cyclohexane ring. The conjugated aromatic and the cyclohexane portions of the molecule were connected via an ester linkage, again consistent with the chemical shift data.

The structural features of fraction f were consistent with the compound, chlorogenic acid ($C_{16}H_{18}O_9$, MW 354.31) (Fig. **11a**). The structural assignment of fraction f as chlorogenic acid was confirmed by comparison of both the ^1H and ^{13}C NMR spectra of fraction f with the published spectra of chlorogenic acid [("Aldrich Library of ^{13}C and ^1H FT NMR Spectra" I (2) 1235C)]. The spectra were identical when solvent dependent chemical shift changes were taken into account. The published UV spectrum of chlorogenic acid was also compared with that of fraction f and found identical. The study indicated that the major compound within fraction f was identified as chlorogenic acid (Fig. **11a**).

b) PTI-777 fraction j identified as epicatechin

Mass Spectroscopy: Numerous attempts to obtain a reliable molecule ion peak of the compound using both the fast atom bombardment (FAB+) and chemical ionization (CI) techniques were unsuccessful. A reliable molecule ion peak (M+1) with m/z 291.05 was however, obtained using electrospray techniques using both time-of-flight (Fig. **S2a**) and fourier transform mass spectroscopy (Fig. **S2b**). This mass to charge (m/z) ratio of 290 is consistent with a possible molecular formula of $C_{14}H_{12}O_7$ or $C_{15}H_{14}O_6$. An electron impact (EI) initiated mass spectrum showed large m/z fragments at 123 ($C_7H_7O_2$), 140 ($C_7H_7O_2$), and 153 ($C_9H_8O_3$)(Fig. **S2c**).

Pentaacetate derivative of compound j: Mass spectra taken in both the FAB+ (Fig. **S2d**) and electron impact (EI) modes (Fig. **S2e**) gave molecular ion peaks of 523 and 500 respectively correlating with a sodiated and non-sodiated pentaacetate derivative of a compound with molecular weight 290. High resolution spectra taken in these modes gave observed m/z ratios of 523, 1214 ($C_{25}H_{24}O_{11}Na$, error -0.4ppm/-0.2mmu) and 500.1317 ($C_{25}H_{24}O_{11}$, error -0.2 ppm/-0.1 mmu). This information firmly established the molecular formula of the pentaacetate derivative of PTI-777 compound j as $C_{25}H_{24}O_{11}$ and hence compound j to have the molecular formula corresponding to $C_{15}H_{14}O_6$.

Nuclear magnetic resonance (NMR) spectroscopy

In d_6 acetone, the ^{1}H NMR spectra (Fig. **S2f**) showed a doublet of doublets centered on 2.8 ppm (2H), a sharp multiplet at 4.2 ppm (1H), and apparent singlet at 4.85 ppm (1H), two singlets at 5.9 (1H) and 6.0 ppm (1H), two multiplets at 6.8 ppm (1H), a sharp multiplet at 7.02 ppm (1H) and four hydroxyl signals at 7.83 (2H), 8.03 (1H), 8.2 (1H), and 3.6 ppm (1H). The ^{13}C NMR spectrum (Fig. **S2g**) displayed signals at 20.06, 66.98, 79.49, 95.72, 96.20 ,99.85, 115.35 ppm, Fig. **S2h**) and ^{13}C NMR (Fig. **S2i**) spectra in D_2O with 0.1% trifluoroacetic acid were significantly different from the above reported spectra. The ^{1}H NMR spectrum (Fig. **S2h**) differed most strikingly in the absence of the two singlets at 5.9 and 6.0 ppm. In the ^{13}C NMR spectrum (Fig. **S2i**), the three signals between 90 and 100ppm moved to just above 160 ppm. The sample remained stable in the acidified D_2O when stored over the course of several months at room temperature, but it began to degrade in acetone within 24 hours.

Correlation spectroscopy (COSY)

Correlation spectroscopy (COSY) spectra of the sample in both d_6 acetone (Fig. **S2j**) and acidified D_2O (Fig. **S2k-S2m**) revealing coupling between the 3 signals from 6.8 to 7.1 ppm and the signals at 2.8 and 4.2 ppm.

Pentaacetate derivative of PTI-777 compound j

The expected methyl groups and carbonyl carbon atoms were present in both the proton and carbon spectra of the acetylated derivative of j. The most striking changes in the ^{1}H NMR spectrum ($CDCl_3$) upon acetylation (Fig. **S2n**) were the downfield shifts at about 0.6 ppm of the two singlets at 5.9 and 6.0 ppm and the downfield shift of 1.2 ppm of the apparent singlet at 4.85 ppm (compare to Fig. **S2h**). The ^{13}C spectrum (Fig. **S2o**) was less affected, but the aromatic carbon atoms were, in general, shifted downfield upon acetylation. All 15 carbon signals associated with compound j were readily seen in the spectrum. In addition to the ^{1}H-^{1}H coupling detected in the COSY spectrum of the unacetylated compound (Figs. **S2j, S2k-S2m**), the derivative (Fig. **S2p**) showed a correlation between the apparent singlet at 4.85 ppm and the sharp multiplet at 4.2 ppm of the original compound. The heteronuclear correlation spectroscopy (HETCOR) spectrum (Figs. **S2q-S2s**) of acetylated derivative was consistent with expectation and confirmed and the assignment and the identity of the protonated carbon atoms. Furthermore, these data are consistent with those reported by Huo et al[93].

Ultra Violet (UV) Spectroscopy

The UV spectrum of PTI-777 compound j showed a maximum at 278 nm consistent with an aromatic phenolic compound (Fig. **S2t**).

Structural Assignment

The ^{13}C NMR spectrum showed the presence of three sp^3 type carbon atoms, 9 sp^2 carbon atoms (no carbonyl carbons) and 3 carbon atoms that were either very upfield sp^2 type or very downfield sp^3 type carbon atoms. The ^{1}H NMR spectrum indicated the presence of 4 phenolic hydroxyl groups and one non-aromatic hydroxyl group. The major fragments observed in the EI mass spectrum corresponded to dihydroxylated benzene rings without and with carbon group substitution (m/z of 123, 139 and 152). The proton NMR data (6.8-7.02 ppm) showed evidence for a tri-substituted benzene ring that was hydroxylated. Comparison of ^{1}H NMR spectra data of model compounds with that of compound j, showed the observed splitting pattern and chemical shifts of the signals were consistent with a 1-substituted, 3,4-dihydroxy benzene structure.

The COSY spectra showed ^1H-^1H coupling in a contiguous three-carbon fragment. The relative chemical shifts of two of these carbon atoms indicated that they were directly bonded to oxygen atoms. The large chemical shift change observed for the signal at 4.8 ppm upon acetylated indicated the location of the non-aromatic hydroxyl group and the chemical shift of the third carbon atom implied that it was benzylic. Together, these data were consistent with the following structure, Ar-CH_2-CH(OH)-CH(R)O.

The above fragments accounted for all but 4 hydrogen atoms (two of which are found in phenolic type OH groups) and for all but 6 carbon atoms (3 sp^2 type and 3 intermediates between sp^3 and sp^2 type). The C_6H_4 formula indicated a high degree of unsaturation consistent with a second aromatic ring. The remaining accounted for singlets in the ^1H NMR at 5.9 and 6.0 ppm revealed that this ring was tetra substituted and electron rich. These data indicated that this benzene ring was bonded to three oxygen atoms (two hydroxyl groups and one ether) and once carbon atom. These three structural units (two phenolic rings and the three carbon fragment) when connected together form a flavanol structure identical to the diastereomers, catechin and epicatechin. Comparison of the ^1H and ^{13}C NMR spectra, as well as the infrared (IR) spectra of compound j with the published spectra (Aldrich collection) for catechin and epicatechin (Figs. **S2u, S2v and S2w**) establishing the identity of PTI-777 compound j as epicatechin. The splitting pattern of doublet of doublets center on 2.8 ppm in the ^1H NMR spectrum matches that of the epicatechin reference spectrum. The pattern of the aromatic signals between 6.8 and 7.02ppm is also most similar to that of epicatechin. The IR spectrum of compound j matches closely with that of epicatechin, while the IR spectrum of catechin differs significantly in the fingerprint region.

c) PTI-777 fraction h2 identified as epicatechin-4β-8-epicatechin (proanthocyanidin B2)

Acetylation of h2

A sample of h2 (7 mg) was dissolved in a mixture of acetic anhydride (0.5 ml) and pyridine (0.5 ml). The mixture stood at room temperature for 18 hours, then the solvents were removed in vacuo. Purification by column chromatography over silica gel, eluting with 20% ethyl acetate in dichloromethane gave the h2 peracetate (6 mg) as a colorless gum.

A sample of a fraction rich in peaks h1 and h2, from a second silica gel column of PTI-777 (50 mg) was dissolved in a mixture of acetic anhydride (0.5 ml) and pyridine (0.5 ml). The mixture stood at room temperature for 18 hours, then the solvents were removed in vacuo. Purification by column chromatography over silica gel, eluting with 20% ethyl acetate in dichloromethane gave the h2 peracetate (28 mg) as a colorless gum.

Identification of peak h2 from PTI-777 as epicatechin-4β-8-epicatechin (proanthocyanidin B2)

The main component of peak h, called h2, from the extract PTI-777, was isolated by a series of chromatographic techniques, monitored by HPLC. We initially separated the original extract PTI-777 by column chromatography over silica gel, when 20% methanol in chloroform gave a fraction rich in the two components of peak h on a preparative scale, to give us mostly pure h1(16 mg) and pure h2 (23 mg). A $^-$ve ion electrospray mass spectrum of peak h2 gave a clean 100% ion at 577 Da. This is appropriate for the molecular ion $(M\text{-}H^+)^-$ of a molecular formula of $C_{30}H_{26}O_{12}$, such as a dimer of two epicatechin, or isomeric units. A ^1H NMR spectrum (Fig. **S3a**) of peak h2 showed unusual broadening of the signals, whilst the ^{13}C NMR (Fig. **S3b**) showed sharp and broad signals, consistent with some kind of flavonol dimer. We were surprised to see no signals in the 5.8 – 6.3 ppm region of the ^1H NMR spectrum, or in the

90-99 ppm region of the ^{13}C NMR spectrum, where the characteristic H-6/H-8 and C-6/C-8 signals would appear. Running the NMR spectra in deuteroacetone (Fig. **S3c; S3d**) instead of deuteromethanol, showed the expected signals to be present, indicating that in deuterated protic solvents, an exchange of these H-6 and H-8 protons for deuterons took place.

Acetylation of h2

Since the compound h2 was unstable under the conditions necessary to prove its structure by NMR spectroscopy, we had to make a stable derivative. Acetylation of a sample of pure h2 gave a peracetate (Fig. **S3e**), which was purified by column chromatography over silica gel. A larger sample of this peracetate, identical by NMR and thin layer chromatography, was also obtained by silica gel separation of the two main products from acetylation of a fraction rich in h1 and h2.

One and 2D NMR experiments (Fig. **S3f; S3g**) on the h2 peracetate showed it to be a decaacetate. Two sets of signals were seen in both the ^{1}H and ^{13}C NMR spectra, in a ratio of three to one. These were due to rotational isomers (atropisomers), shown by opposite phase cross peaks in the NOESY spectrum to interconvert in the time frame of the NMR experiment. We solved the structure using the signals of the major atropisomer. The presence of two flavan-3-ol units could be seen from the four ^{13}C signals for the C-2 and C-3 positions in the 60 – 80 region, as well as a signal at 26.65 for the free C-4 position of the lower unit and a signal at 33.99 for the linked C-4 of the upper unit. A CIGAR ^{1}H - ^{13}C correlation experiment (Figs. **S3h; S3i**) showed that the two units were connected from the 4(u) position to the 8(l) position, by the correlations from H-4(u) to C-8(l) and C-8a(l). The stereochemistries at C-2 and C-3 of both upper and lower units was shown to be the same as in epicatechin by the similar chemical shifts of the ^{1}H and ^{13}C signals for the lower unit, as well as the similar low coupling constants between H-2 and H-3 in both units. The stereochemistry of the linkage was shown to be 4β-8 from the NOE interactions (Figs. **S3j; S3k; S3l**), in particular the lack of an interaction between H-2(u) and H-4(u), and the presence of an interaction between H-2(u) and H-6'(l), and between H-3(u) and H-6'(l). The structure of the natural product h2 was therefore assigned to be epicatechin-4β-8-epicatechin (Fig. **S3m**).

Epicatechin-4β-8-epicatechin is also known as procyanidin B2 or proanthocyanidin B2. Our NMR data on PTI-777 h2 matched partial NMR data published on procyanidin B2[104-105] and our data of the PTI-777 h2 peracetate exactly matches the published data on peracetylated procyanidin B2[106]. The optical rotation of +29.0^{o} compared to a literature[5] value of +25° showed the absolute stereochemistry to be the same as found previously.

Peak h2

ESI-TOF -ve-m.s. 577 ($M-H^{+}$, 100%)

^{1}H NMR (CD_3OD): 2.81 (1H, br d), 2.95 (1H, br d), 3.92 (1H, s), 4.30 (1H, br s), 4.65 (1H, br s), 4.98 (br s), 5.07 (1H, br s), 6.70 - 7.20 (6H, m).
^{13}C NMR (CD_3OD): 29.97 (C-4l), 37.50 (C-4u), 67.33, 73.83, 77.45, 80.23, 101.00, 115.71, 116.27, 116.31, 119.77, 132.41, 145.96 and 146.22.

^{1}H NMR $((CD_3)_2CO)$: 2.83 (1H, m, H-4l), 2.96 (1H, m, H-4l), 4.10 (1H, s), 4.41 (1H, br s), 4.83 (1H, s, H-4u), 5.07 (1H, br s), 5.20 (1H, br s), 6.07 (1H, s), 6.09 (1H, s), 6.12 (1H, s), 6.80 - 7.20 (6H, m) and 7.50 - 8.30 (6H, br s, OHs)

^{13}C NMR $((CD_3)_2CO)$: 30.3, 37.6, 67.1, 73.6, 77.6, 79.9, 96.6, 97.1, 97.7, 101.3, 115.5 - 116.2, 119.9, 120.2, 132.5, 133.1, 145.6 – 146.1, 156.4 – 160.0.

UV (MeOH) λ max (log ε) 210 (4.79), 227sh (4.48) and 280 (3.81) nm;

$[\alpha]^{24}_{589nm}$ + 29.0°, $[\alpha]^{24}_{577nm}$ +19.8°, $[\alpha]^{24}_{546nm}$ +5.7°, $[\alpha]^{24}_{435nm}$ –87.4°, $[\alpha]^{24}_{405nm}$ -106.1°(c 0.2, MeOH)

d) Isolation and Identification of the minor component, h1, of peak h from PTI-777 as catechin-4α-8-epicatechin (proanthocyanidin B4)

Experimental Procedures

Aliquots (14 X 70µl) of fractions 20-24 (134 mg in 1ml) (**Fig. S4a**) from a silica gel column were separated by HPLC. The peaks between 14.5 and 16.2 and 16.2 and 19.0 minutes were collected, then freeze dried to give two products, about 80% pure h1, retention time 15.1 minutes (16mg) (**Fig. S4b)** as a white solid; and pure h2 (23 mg) as a white solid.

Acetylation of h1 protocol

A sample of h1 (5 mg) was dissolved in a mixture of acetic anhydride (0.5 ml) and pyridine (0.5 ml). The mixture stood at room temperature for 18 hours, then the solvents were removed in vacuo. Purification by column chromatography over silica gel, eluting with 20% ethyl acetate in dichloromethane gave the h1 peracetate (2 mg) as a colorless gum. A sample of a fraction rich in peaks h1 and h2, from a second silica gel column of PTI-777 (50 mg) was dissolved in a mixture of acetic anhydride (0.5 ml) and pyridine (0.5 ml). The mixture stood at room temperature for 18 hours, then the solvents were removed in vacuo. Purification by column chromatography over silica gel, eluting with 20% ethyl acetate in dichloromethane gave the h2 peracetate (38 mg) followed by the h1 peracetate (15 mg) as a colorless gum.

Results:

The minor component of peak h, called h1, of the extract PTI-777 was isolated by a series of chromatographic techniques, monitoring by HPLC. We initially separated the original extract PTI-777 by column chromatography over silica gel, when 20% methanol in chloroform gave a fraction rich in the two components of peak h (134 mg). An HPLC method was developed to separate the two main components of peak h on a preparative scale, to give us a mostly pure h1 (16 mg) and pure h2 (23 mg).

A -ve ion electrospray mass spectrum of h1 gave a 100% ion at 577 daltons. This is approximate for the molecular ion $(M-H^+)$ of a molecular formula of $C_{30}H_{26}O_{12}$ (molecular weight 578), such as a dimer of two epicatechin, or isomeric units. We had previously isolated and identified epicatechin from the PTI-77 extract (described above).

A ^{1}H NMR spectrum (Fig. **S4c**) and ^{13}C NMR spectrum (Fig. **S4d**) of peak h1 showed two sets of signals, consistent with some kind of flavonol dimer, with major and minor atropisomers present.

Acetylation of a sample of h1 gave a peracetate (Fig. **S4e**), which was purified by column chromatography over silica gel. A larger sample of this peracetate, identical by NMR and thin layer

chromatography, was also obtained by silica gel separation of the two main products from acetylation of a fraction rich in h1 and h2.

One and 2D NMR experiments (Figs. **S4f; S4g**) on the h1 peracetate showed it to be a decaacetate. The structure was solved using the signals of the dominant atropisomer. The presence of two flavan-3-ol units could be seen from the four ^{13}C NMR signals in the 60 – 80 ppm region, as well as a signal at 26.56 for the free C-4 position of the lower unit and a signal at 36.72 ppm for the coupled C-4 of the upper unit (Fig. **S4g**). The positions of the ^{13}C signals and the small couplings of the ^{1}H signals of the lower unit were typical of an epicatechin unit, while the positions of the ^{13}C signals and the much larger couplings of the ^{1}H signals of the upper unit were typical of a coupled catechin[107].

A CIGAR ^{1}H – ^{13}C correlation experiment (Figs. **S4h; S4i**) showed that the two units were connected from the 4(u) position to the 8(l) position, by the correlations from H-4(u) to C-8(l) and C-8a(l).

The structure of the natural product h1 was therefore assigned to be catechin-4α-8-epicatechin also known as procyanidin B4 or proanthocyanidin B4 (Fig. **S4j**). Our NMR data on compound h1 matched partial NMR data published on procyanidin B4[105-106] and our data of acetylated compound h1 (**Fig. S4e**) matched partial NMR data published on peracetylated procyanidin B4[107-108]. The optical rotation of -102° (MeOH) for compound h1 compared to a literature value of -193° (EtOH) showed the absolute stereochemistry to be the same as found previously[108].

ESI-TOF -ve-m.s. 577 ($M-H^{+}$, 100%)

-molecular weight 578

<u>^{1}H NMR </u>($(CD_3)_2CO$)(major isomer, partial data) :2.92 (1H, dd, 2, 16, H-4l), 3.02 (1H, dd, 5, 16, H-4l), 4.34 (1H, bs, H-3l), 4.54 (1H, d, 10, H-4u), 4.64 (1H, dd, 8, 10, H-3u), 4.79 (1H, d, 8, H-2u), 5.09 (1H, s, H-2l), 5.94 (1H, d, 2, H-6u), 5.96 (1H, d, 2, H-8u) and 6.15 (1H, s, H-6l).

<u>^{13}C NMR </u>($(CD_3)_2CO$)(major isomer, partial data):30.30 (determined from HSQC correlation), 38.85, 67.49, 73.89, 80.52, 83.99, 96.76, 97.81, 98.02, 106.66, 108.29 and 116.11.

<u>UV </u>(MeOH) λ max (log ε) 209 (4.71), 225sh (4.44) and 281 (3.65) nm;

$[\alpha]^{24}_{589nm}$ -103.0°, $[\alpha]^{24}_{577nm}$ -118.3°, $[\alpha]^{24}_{546nm}$ -153.1°, $[\alpha]^{24}_{435nm}$ -379.9°, $[\alpha]^{24}_{405nm}$ -469.1°(c 0.2, MeOH)

e) PTI-777 fraction k2 identified as epicatechin-4β→8-epicatechin-4β→8-epicatechin or proanthocyanidin C1

The major component of peak k, called k2, of the PTI-777 extract was also isolated by a series of chromatographic techniques, monitored by HPLC. We initially separated the original PTI-777 extract by column chromatography over silica gel, when 40% methanol in chloroform gave a fraction rich in the major component of peak k. Preparative HPLC on a fraction rich in k2 (Fig. **S5a**) gave a pure sample of peak k2 (Fig. **S5b**). A –ve ion electrospray mass spectroscopy of this showed it to have a molecular ion M^{+} of 866 (Fig. **S5c**). This is appropriate for a molecular formula of $C_{45}H_{38}O_{18}$ (molecular weight = 866), such as a trimer of three epicatechin or catechin units. The initial ^{1}H NMR (Fig. **S5d**) showed there to be

similar broad peaks to that seen in h2, so it was decided to acetylate the compound to definitely identify the structure of k2. A further fraction from the silica gel column that was rich in peak k2 was acetylated as before to enable us to obtain more material for structure elucidation. The peracetate of k2 was purified by column chromatography over silica gel.

One (Fig. **S5e**) and 2D NMR experiments (Fig. **S5f**) were carried out on the k2 peracetate. Two sets of signals were seen in both the ^{1}H and ^{13}C NMR spectra in a ratio of three to one. These were due to rotational isomers (aptropisomers). The structure was determined from signals of the major isomer.

The position of the ^{13}C NMR signals and the small couplings of the ^{1}H NMR signals of the lower unit was typical of epicatechin, and the positions of the ^{13}C signals and the small couplings of the ^{1}H signals of the other two units were typical of coupled epicatechin[107]. The presence of three flavon-3-ol units could be seen from the six ^{13}C signals in the 60-80 ppm region, as well as a signal at 26.39 from the free C-4 position of the lower unit and signals at 34.36 and 35.04 for the coupled C-4's of the other units.

A CIGAR ^{1}H - ^{13}C correlation experiment (Figs. **S5g; S5h**) showed that the two units were connected from the 4 (upper) position to the 8 (lower) positions, by the correlations form H-4(u) to C-8(m) and C-8a(m), and from H-4(m) to C-8(l) and C-8a(l).

K2 peracetate was therefore determined to be the structure shown in Fig. **S5i**. Our NMR data on the K2 peracetate shown in Fig. **S5i** matched partial NMR data published on proanthocyanidin C1[104] but we could not find any ^{13}C NMR data published on structure k2 (Fig. **S5j**). The optical rotation of +60.9^{o} (MeOH) for structure k2 compared to the literature value of +92° (H_2O) showed that it to have the same absolute stereochemistry as that published. K2 was therefore identified as epicatechin-4β→8-epicatechin-4β→8-epicatechin or proanthocyanidin C1 (Fig. **S5j**).

<u>^{13}C NMR</u> ($(CD_3)_2CO$ (partial data on major isomer); 29.3 (determined from HSQC correlation), 37.6, 37.6, 67.10, 72.68, 73,69, 77.48, 77.61, 79.85.

<u>UV</u> (MeOH) λ max (log ε) 211 (4.95), 226sh (4.66) and 281 (3.94) nm;
$[\alpha]^{24}_{589nm}$ + 60.9^{o}, $[\alpha]^{24}_{577nm}$ +53.2^{o}, $[\alpha]^{24}_{546nm}$ +40.0^{o} (c 0.2, MeOH)

<u>Acetylation of k2 protocol</u>
A sample of k2 (5mg) was dissolved in a mixture of acetic anhydride (0.5ml) and pyridine (0.5ml). The mixture was kept at room temperature for 18 hours, then the solvents were removed *in vacuo*. Purification by column chromatography over silica gel, eluting with 20% ethyl acetate in dichloromethane gave the k2 peracetate (2 mg) as a colorless gum. A fraction rich in k2 (34 mg) (Fig. **S5b**) was dissolved in a mixture of acetic anhydride (0.5ml) and pyridine (0.5ml). The mixture was kept at room temperature for 18 hours, then the solvents were removed in vacuo. Purification by column chromatography over silica gel, eluting with 20% ethyl acetate in dichloromethane gave the k2 peracetate (15mg) as a colorless gum.

f) PTI-777 fraction k1 identified as epiafzelechin-4β-8-epicatechin

General Experimental Procedures

A sample of the PTI-777 extract (1 gram) was dissolved in ethanol (2 ml) and then loaded onto a sephadex LH20 (10g) column, prepared in ethanol. Elution of this column with ethanol (100ml), followed by 5% acetone in ethanol (400ml), 20% acetone in ethanol (200 ml), then 50% acetone in methanol (200ml) gave 120 (12 ml) fractions.

Isolation of peak k1:

Fractions 38 to 42 contained compound k1 (22mg) as a pale brown gum. The retention time of this k1 peak was 15.0 minutes as monitored by HPLC. For acetylation of k1 to determine the structure, a sample of k1 (15 mg) was dissolved in a mixture of acetic anhydride (0.5ml) and pyridine (0.5ml). The mixture stood at room temperature for 18 hours, then the solvents were removed *in vacuo* to give the k1 peracetate (16mg) as a colorless gum.

Isolation of k1 and the k1 peracetate:

The minor component of peak k, called k1, of the PTI-777 extract was isolated by column chromatography over sephadex LH20, monitored by HPLC. Elution with 95% ethanol followed by increasing amounts of acetone and water, followed by methanol, gave pure peak k1 in fractions 38 to 42 . The structure of the k1 peracetate is shown in Fig. **S6a**, whereas the structure of k1 is shown in Fig. **S6b**. To arrive at these structures, the following analysis and results were obtained.

A –ve ion spectroscopy mass spectrum of k1 gave a 100% ion at 561 daltons (Fig. **S6c)**. This is appropriate for the molecular ion (M^+-H) of a molecular formula of $C_{30}H_{26}O_{11}$ (molecular weight = 562), such as a mixed dimer of one epicatechin, or isomeric unit and one epiafzelechin, or isomeric unit. The ^{13}C NMR of k1 showed signals consistent with some kind of flavanol dimer (Fig. **S6d**). The ^{1}H NMR spectrum of k1 showed there to be similar broad peaks to that seen in compound h2 (Fig. **S6e**), so it was decided to acetylate the compound to determine the final structure. Acetylation of pure k1 gave a peracetate (structure shown in Fig. **S6a**). The ^{1}H and ^{13}C spectrum of the k1 peracetate are shown in Fig. **S6f** and **Fig. S6g**, respectively. Two sets of signals were seen in both the ^{1}H and ^{13}C NMR spectra, in a ratio of three to one. These were due to rotational isomers (atropisomers). The structure was determined using the signals of the major atropisomer.

The presence of the two flavan-3-ol units could be seen from the four ^{13}C signals for the C-2 and C-3 position in the 60-80 ppm region, as well as a signal at 26.61 ppm for the free C-4 position of the lower unit and a signal at 34.14 for the linked C-4 of the upper unit. A CIGAR ^{1}H-^{13}C correlation experiment (Figs. **S6h to S6j**) showed that the two units were connected from the 4(μ) position to the 8(l) position between H-4(μ) and C-8(l). The sterochemistries at C-2 and C-3 of both upper and lower units was shown to be the same as in epicatechin by the similar chemical shifts of the ^{1}H and ^{13}C signals for the lower unit, as well as the similar low coupling constants between the H-2 and H-3 in both units. The lower flavan-3-ol unit was shown to be epicatechin by CIGAR correlations from H-2(l) to C-2’ and C-6’ signals of the 3’-4’-dioxygenated aromatic ring. The upper flavan-3-ol unit was identified by CIGAR correlations from H-2(μ) to equivalent C-2’/C-6’ signals of a 4’-oxygenated ring. This constitutes an epiafelechin unit. The structure of the natural product k1 was therefore assigned to be epiafzelechin-4β→8-epicatechin. This compound is a known compound[109-110]. Our NMR data on the structure for k1 matched partial NMR data published on epiafzelechin4β→8-epicatechin. The optical rotation of -1.4^o compared to a literature value

of +29° showed uncertain stereochemistry.

Peak k1 data summary:
ESI-TOF -ve-m.s. 561 ($M-H^+$, 100%)

Molecular weight 562

^{1}H NMR (($(CD_3)_2CO$):2.88 (2H, m, H-41), 3.59 (1H, br, s, OH), 3.73 (1H, br, s, OH), 4.11 (1H, s), 4.39 (1H, br, s), 4.84 (1H, s, H-4μ), 5.07 (1H, br,s), 5.26 (1H, s), 6.08 (1H, s), 6.09 (1H, s), 6.12 (1H, s), 6.80 (1H, m), 6.85 (2H, d, J 8Hz), 6.97 (1H, m), 7.19 (1H, br, s), 7.37 (2H, d, J 8hz) and 7.40-8.20 (7H, br s, OHs)

^{13}C NMR (($(CCD_3)_2CO$): 37.69, 67.11, 73.47, 77.70, 79.95, 96.63, 97.20, 97.74, 101.30, 115.62, 116.14, 119.84, 129.89, 132.48, 145.79, 145.98, 156.78, and 158.28.

UV (MeOH) λ max (log ε) 216 (4.89), 227 sh (4.74) and 280 (4.04) nm;
$[\alpha]^{24}_{589nm}$ -1.4°, $[\alpha]^{24}_{577nm}$ -23.1°, $[\alpha]^{24}_{546nm}$ -62.3°, (c 0.1, MeOH)

g) PTI-777 fraction l identified as epicatechin-4β→8-epicatechin-4β→8-epicatechin4β→8-epicatechin4β→8-epicatechin (epicatechin tetramer)

Peak l from the extract PTI-777 was isolated and shown from electrospray mass spectroscopy, degradation studies and by partial NMR studies to be the epicatechin tetramer epicatechin-4β→8-epicatechin-4β→8-epicatechin4β→8-epicatechin (Fig. **S7**). The compound proved to be unstable in solution, and to acetylation conditions, which prevented full characterization in the same manner as that used for the other proanthocyanidins previously isolated from this extract. The epicatechin tetramer has been reported in the literature, and our partial data match that partial data reported.

Results:
Peak l of the extract PTI-777 was isolated by a series of chromatographic techniques monitored by HPLC. We separated the original extract of PTI-777 by column chromatography over silica gel, when 50% methanol in chloroform gave a fraction rich in peak l. Preparative HPLC of this fraction, using method 2 gave a pure sample of peak l. A –ve ion electrospray m.s. of a sample of peak l, showed it to have a pseudo molecular ion [M-H] of m/z 1153. The spectrum also showed a strong ion with m/z 576 due to $[M-2H]^{2-}$. This is appropriate for a molecular formula of $C_{80}H_{50}O_{24}$, such as a tetramer of four epicatechin or isomeric units.

^{1}H and ^{13}C NMR spectra of peak l showed the typical peaks for an epicatechin oligomer, the peaks in the 60 to 90 ppm region are diagnostic of the flavanol units present, peaks at 67 and 80 ppm were seen for the C-2 and C-3 of a terminal epicatechin, then a group of peaks between 77 and 78 ppm for the -2 of linked epicatechins, and a group of peaks between 72 and 74 for the C-3 of linked epicatechins. The spectra were limited due to the small amount of sample, as well as its instability in solution over time, but the ^{13}C NMR spectrum did match (allowing for slight differences due to solvent) that of proanthocyanidin B2 (Fig. **S7**) reported previously[110]. Attempts to acetylate a newly isolate sample of peak l, gave only the peracetate of the epicatechin dimer (PTI-777 fraction h2), which indicated that peak l is not stable to the acetylation conditions.

The small amount of sample available of a four flavanol unit oligomer, with the additional complication of rotational isomers and its instability in solution, means that the structure of peak l couldn't be elucidated by NMR alone. Indeed, the structure of most oligomers of this size and many of the trimer flavanols have been previously proven by degradation studies. The units are cleaved in ethanolic HCl, then the resultant flavanol cations, formed from all of the linked units, are trapped out by reaction with phloroglucinol, to give their 4-phloroglucinol adducts[111]. The terminal unit is left as the free flavanol. Therefore, this reaction can be used to work out which units are present, which is the terminal unit and the ratio of linked units to terminal units.

HPLC analysis of the reaction products of peak l, with phloroglucinol in ethanolic HCL showed three peaks, two equivalents of epicatechin-4-phloroglucinol, one equivalent of epicatechin and one equivalent of the epicatechin dimer, proanthocyanidin B2 (i.e. Fraction PTI-777 peak h2). The epicatechin dimer, proanthocyanidin B2 is the result of incomplete degradation, but can be used in conjunction with other products to prove the structure. This showed that peak l must be comprised of 4 units of epicatechin. The results are very similar to those obtained from phloroglucinol degradation of the proanthocyanidin previously described[111]. Further evidence that peak l is made of epicatechin units comes from the formation of peak l from a disproportionate reaction of the proanthocyanidin dimer, Proanthocyanidin B2, in acidic methanol, which leads to the formation of epicatechin (PTI-777 peak j), the epicatechin trimer C1 (PTI-777 peak k2), as well as peak l, and post-peak l material. Also, it was noted that one of the products from a disproportion reaction of the proanthocyanidin trimer C1 (PTI-777 peak k2), formed when left in methanol, was the proposed tetramer peak l. The formation of peak l from dimer proanthocyanidin B2 and trimer proanthocyanidin C1, combined with the degradation studies indicate that the tetramer must be made up of 4β→8 linkages, giving us the structure of peak l as epicatechin-4β→8-epicatechin-4β→8-epicatechin4β→8-epicatechin4β→8-epicatechin.

Supplementary Information -References:

104. Hemingway R.W., Foo L. Y. & Porter, J., J. Linkage isomerism in trimeric and polymeric 2,3-cis-procyanidins. *J. Chem. Soc. Perkin Trans.* **1**, 1209-1216 (1982).

105. Kashiwada, Y. et al. Tannins and related compounds. XCIII. Occurrence of enantiomeric proanthocyanidins in the Leguminosae plants, cassia fistula L. and C. javanica L. *Chem. Pharm. Bull.* 38, 888-893 (1980).

106. Franck, U., Neszmelyi, A. & Wagner, H. 2D-NMR structural elucidation of a procyanidin from Musanga cecropioides. *ACH Models in Chemistry* **136**, 511-517 (1999).

107. Fletcher, A.C., Porter, L.J., Haslam, E. & Gupta, R.K. Plant proanthocyanidins. Part 3. Conformational and configurational studies of natural procyanidins. *JCS Perkin* **1**, 1628-1637 (1977).

108. Thompson, R.S., Jacques, D., Haslam, E. & Tanner, R.J.N. Plant proanthocyanidins. Part I. Introduction; the isolation, structure and distribution in nature of plant procyanidins. *J. Chem. Soc. Perkin Trans* **1**:1387-1399 (1972).

109. Morimoto, S., Nonaka, G., Chen, R. & Nishioka. Tannins and related compounds. LXI: isolation and structures of novel bi- and triflavanoids from the leaves of Cassia fistula L. *Chem. Pharm. Bull.* **36**, 39-47 (1988).

110. Foo, L.Y & Lu, Y. Isolation and identification of procyanidins in apple pomace. *Food Chemistry* **64**, 511-518 (1999).

111. Koupai-Abyazani, M.R. et al. Determination of the constituent flavonoid units in sainfoin proanthocyanidins by reversed-phase high performance liquid chromatography. *J. Chromatog.* **594**, 117-123 (1992).

Fig. S1a, S1b

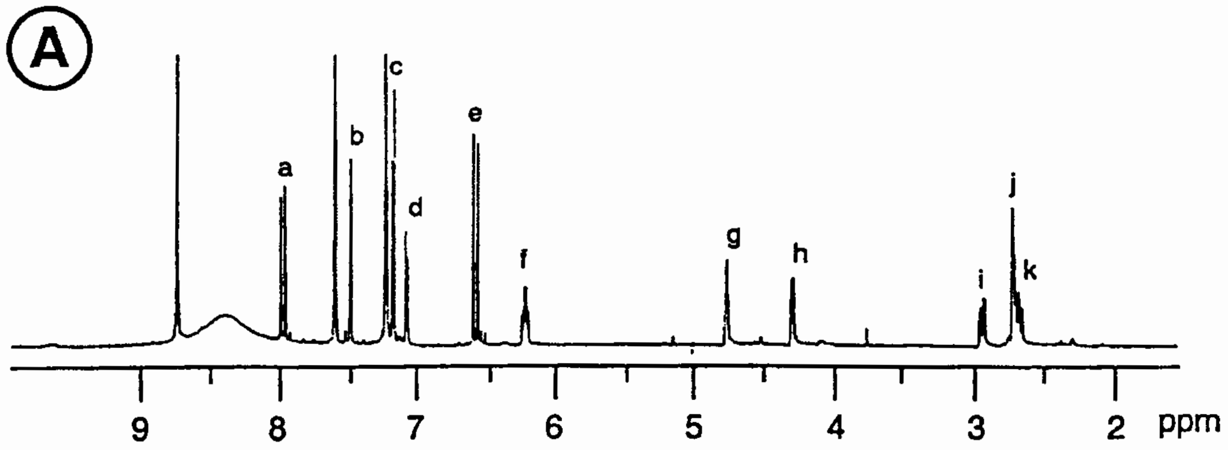

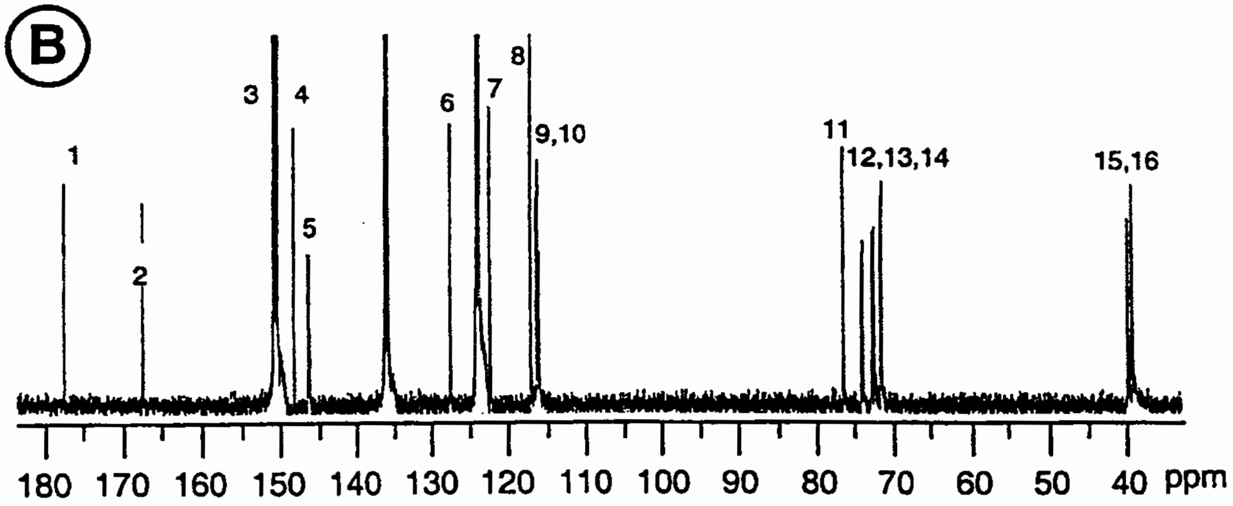

Fig. S1a. ^{1}H NMR (500 MHz) of fraction f in pyridine (d_5).
Fig. S1b. ^{13}C NMR (500 MHz) of purified fraction f in pyridine (d_5).

Fig. S2a

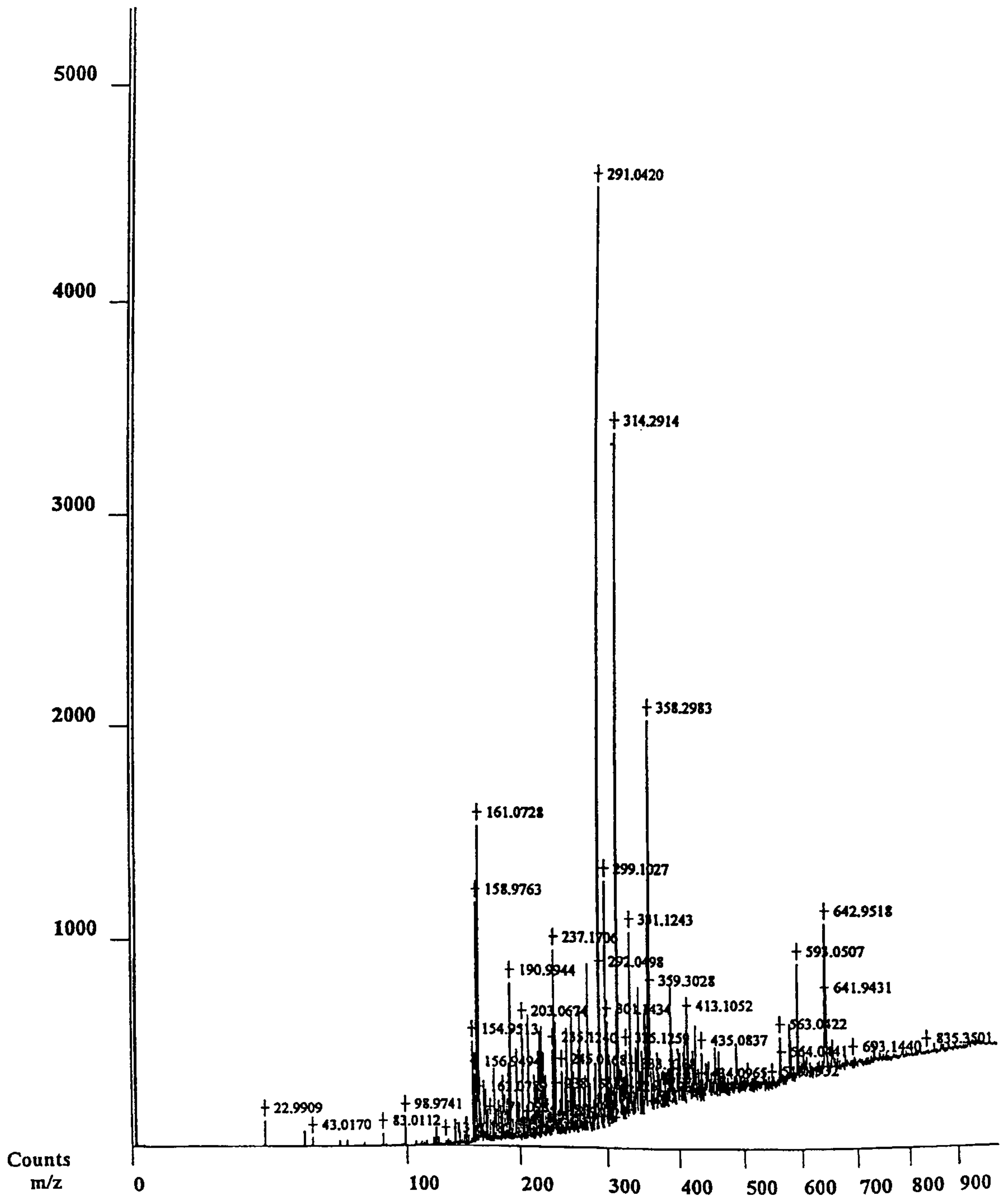

Fig. S2a. Electrospray technique using time of flight mass spectroscopy of purified PTI-777 compound j.

Fig. S2b

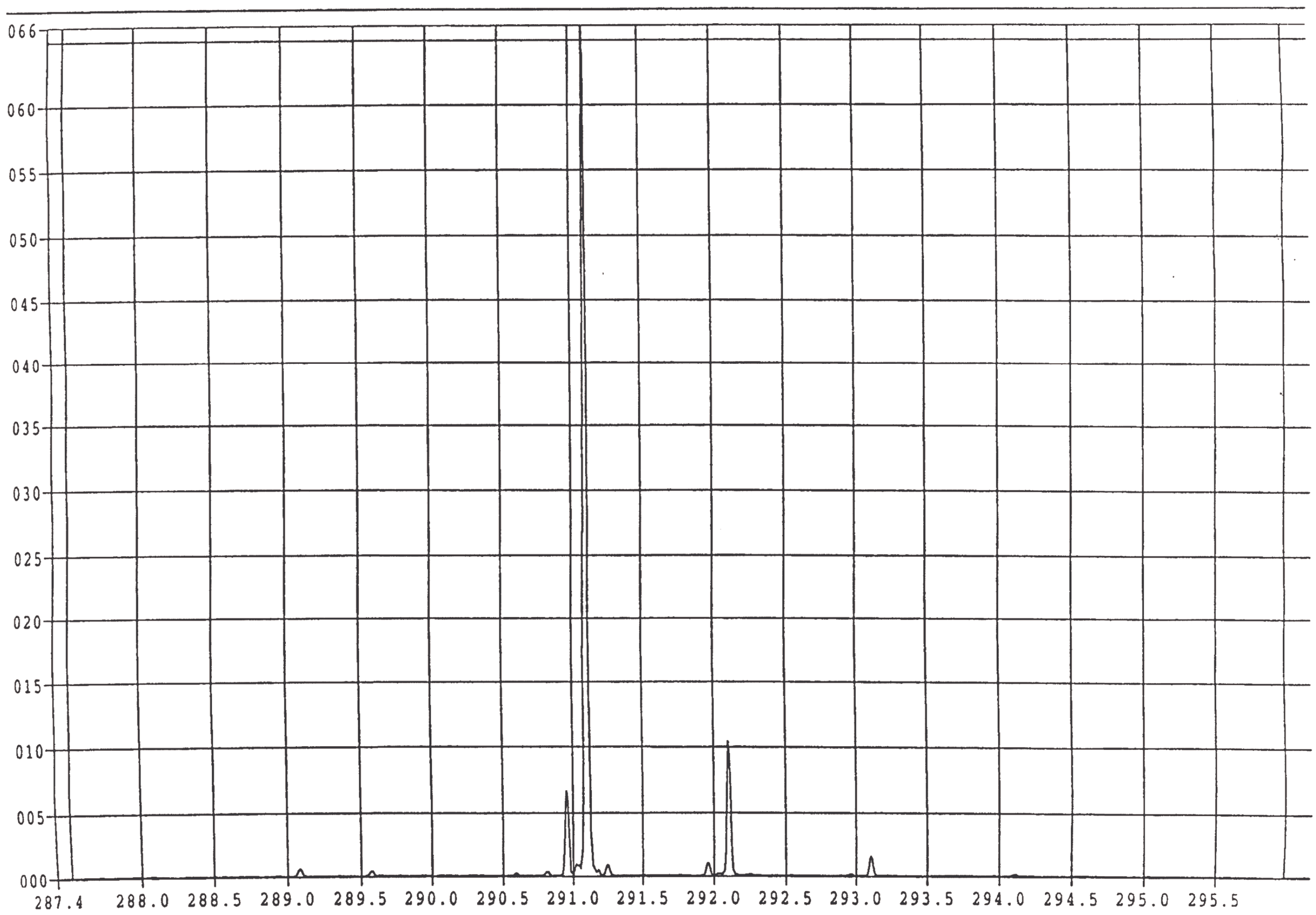

Fig. S2b. Electrospray technique using fourier transform of purified PTI-777-compound j.

Fig. S2c

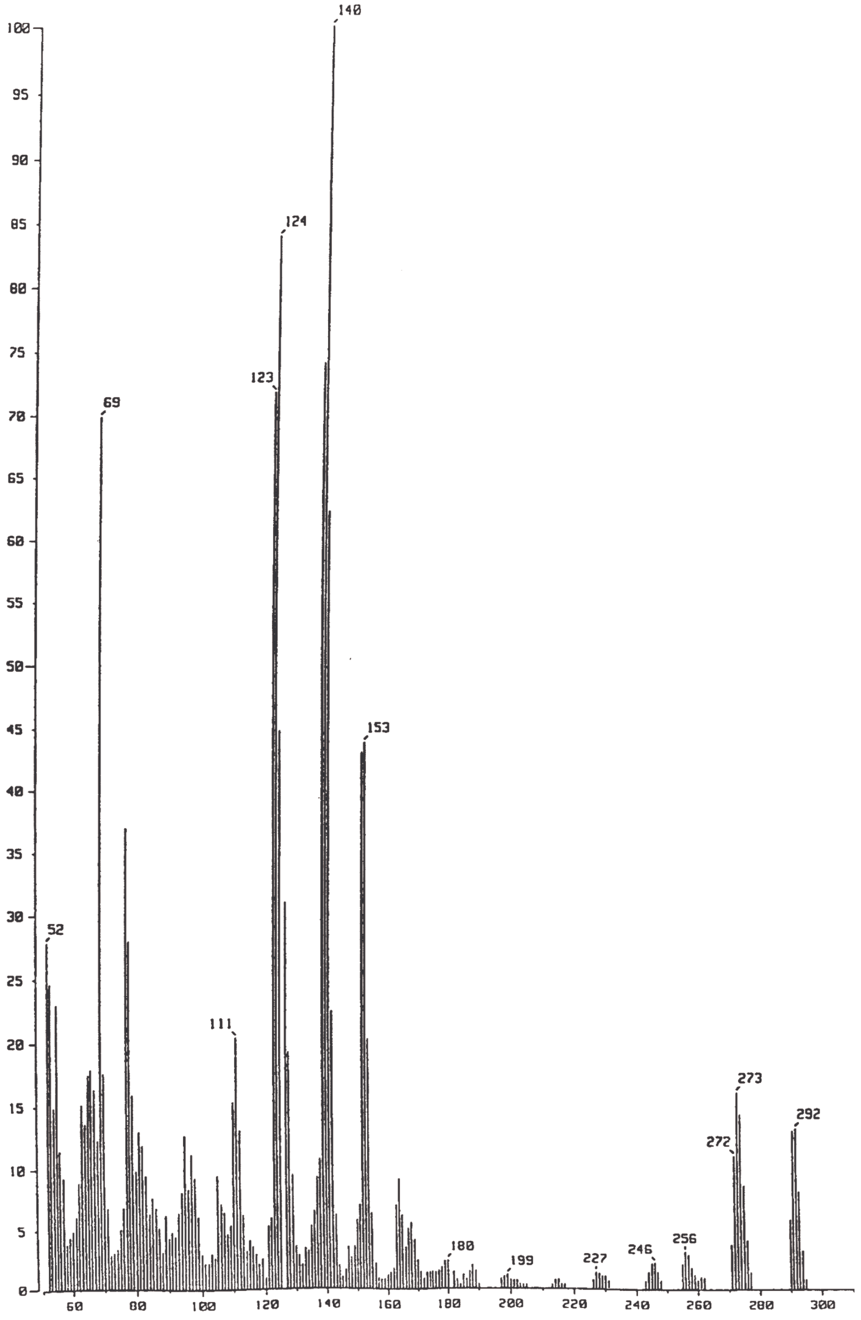

Fig. S2c. Electron impact (EI) initiated mass spectrum of purified PTI-777 compound j.

Fig. S2d

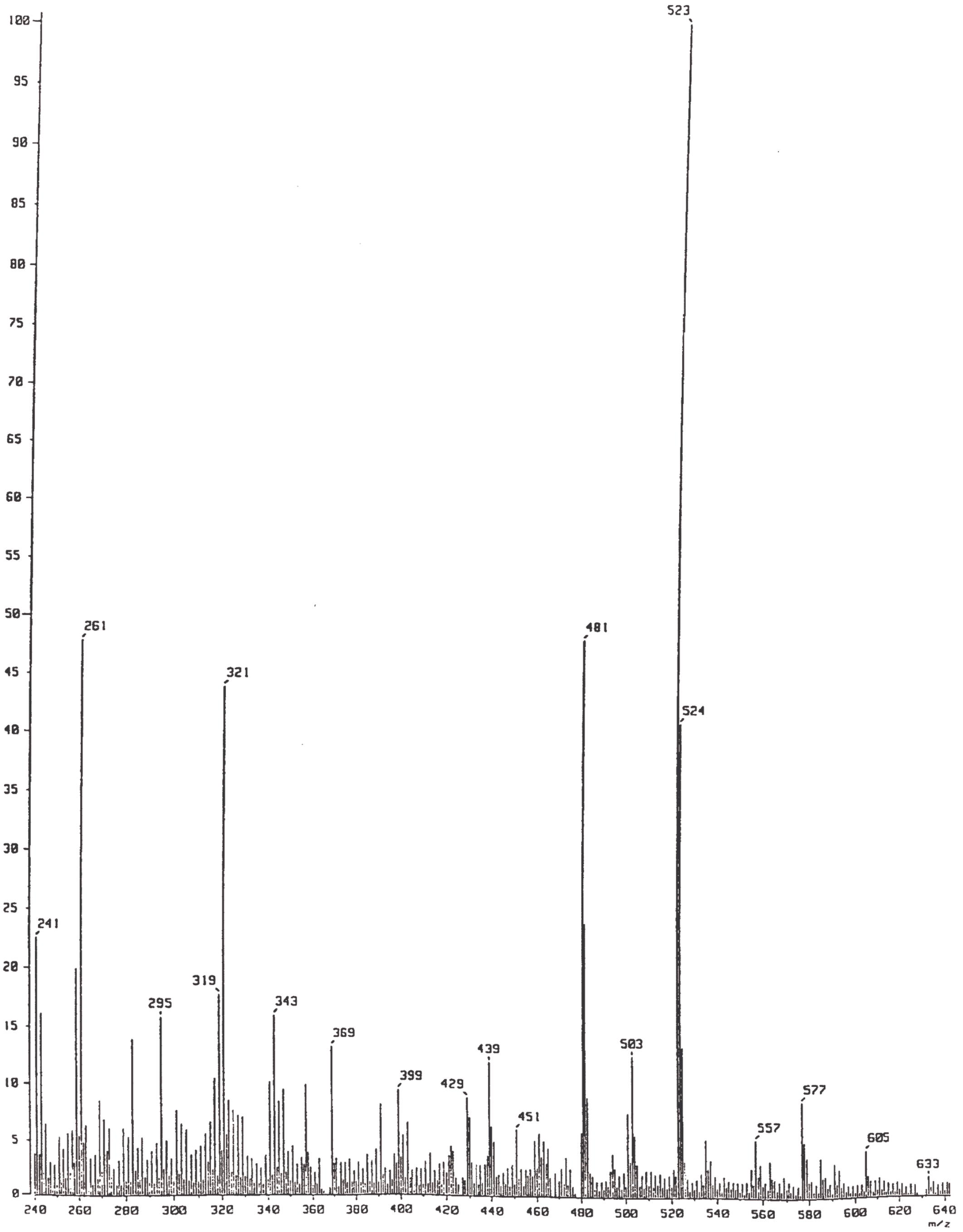

Fig. S2d. Fast atom bombardment (FAB) mass spectroscopy of PTI-777 compound j that had been acetylated.

Fig. S2e

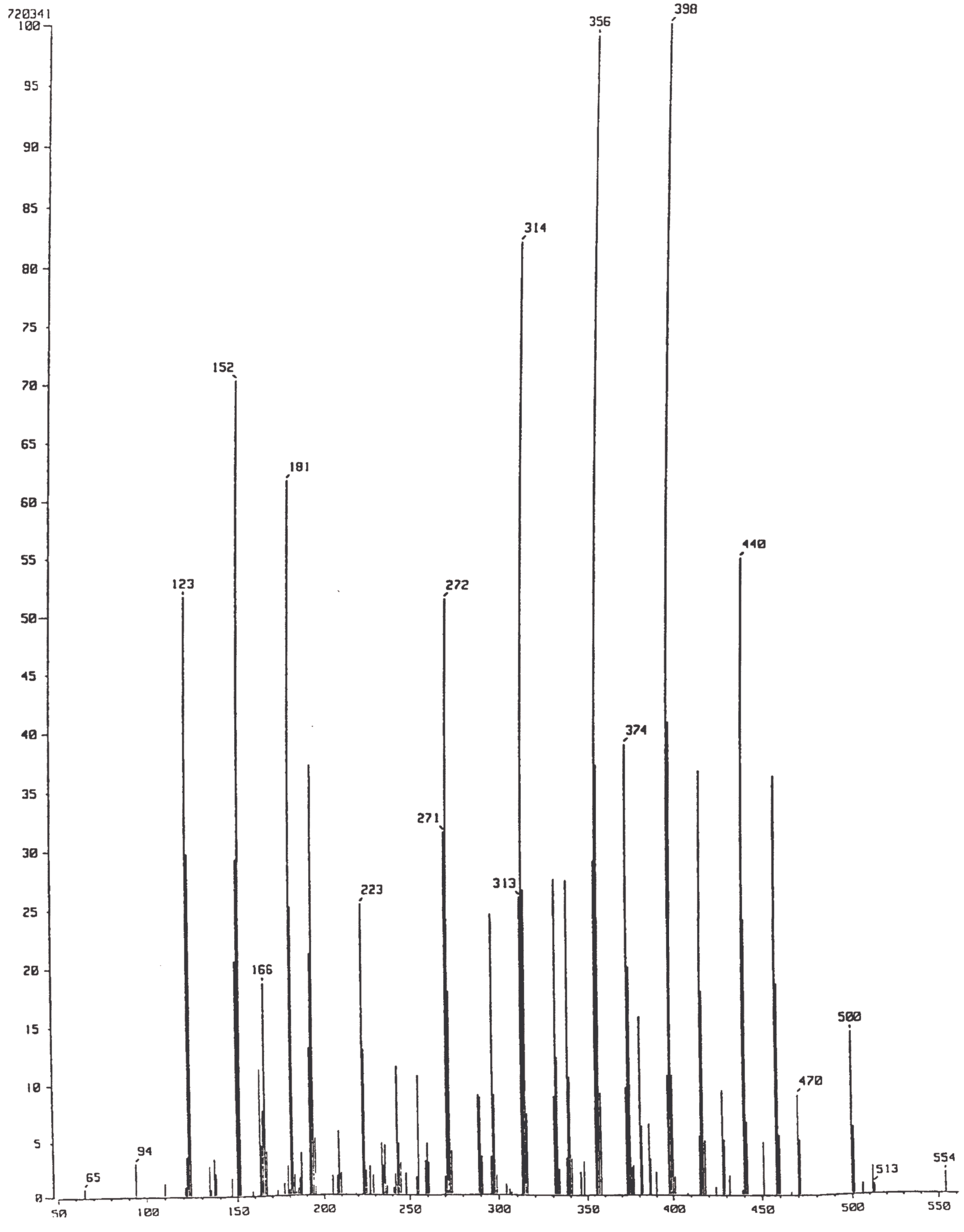

Fig. S2e. Electron impact (EI) mass spectroscopy of purified PTI-777 compound j that had been acetylated.

Fig. S2f

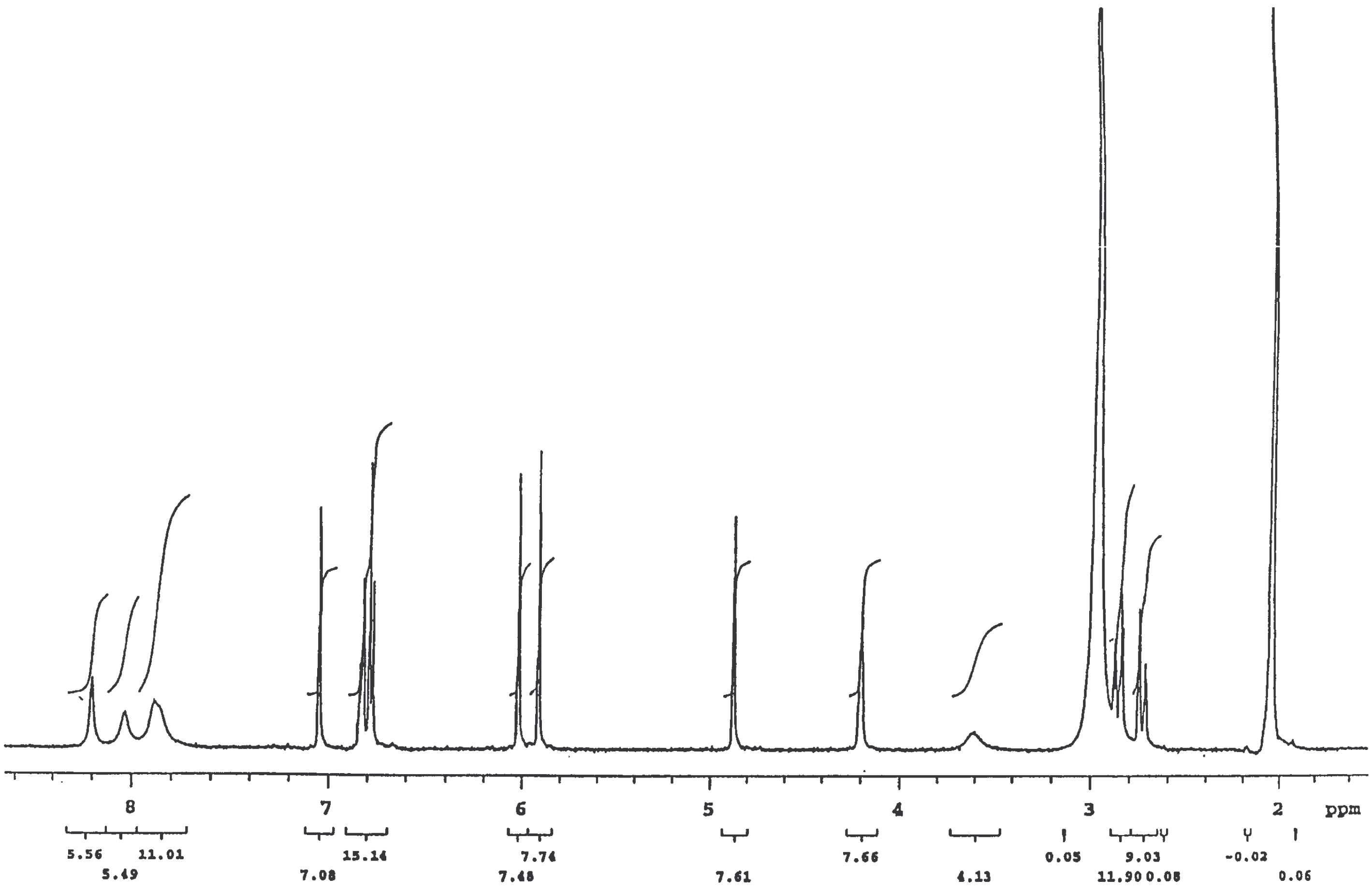

Fig. S2f. ^{1}H NMR spectra of PTI-777 compound j in d_6 acetone.

Fig. S2g

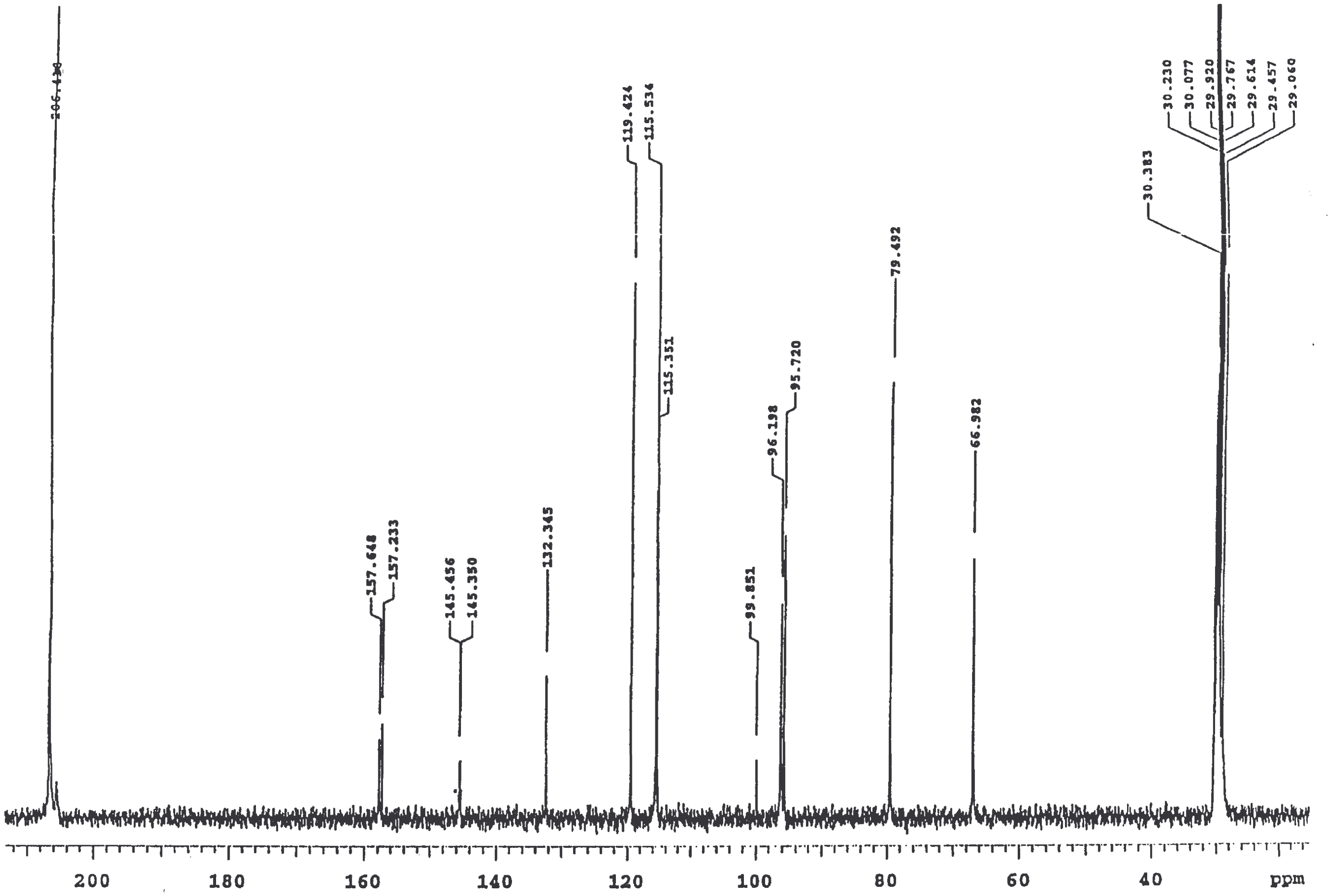

Fig. S2g. ^{13}C NMR spectra of PTI-777-compound j in d_6 acetone.

Fig. S2h

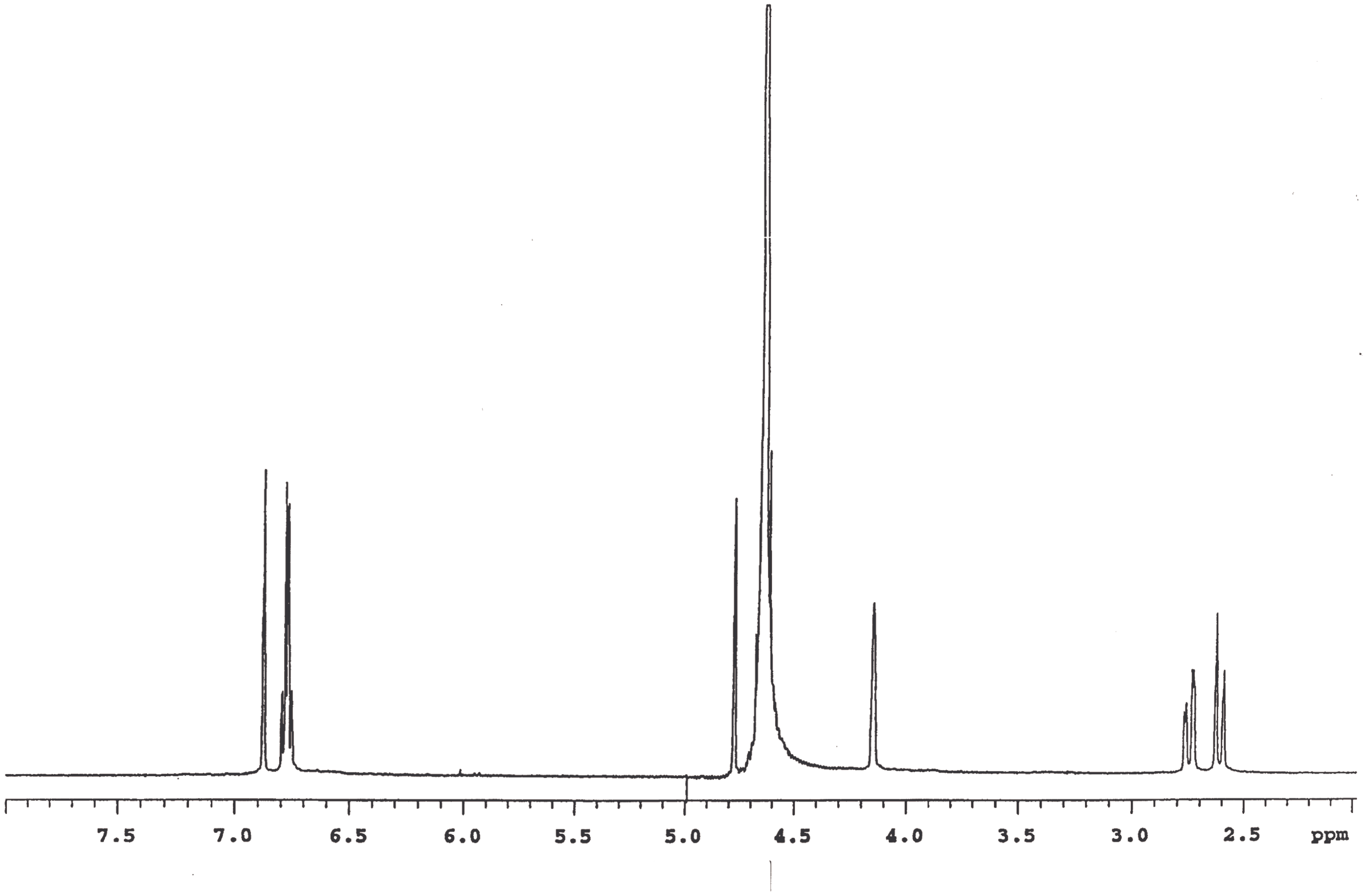

Fig. S2h. ^{1}H NMR spectra of PTI-777-compound j in D_2O with 0.1% trifluoroacetic acid (TFA).

Fig. S2i

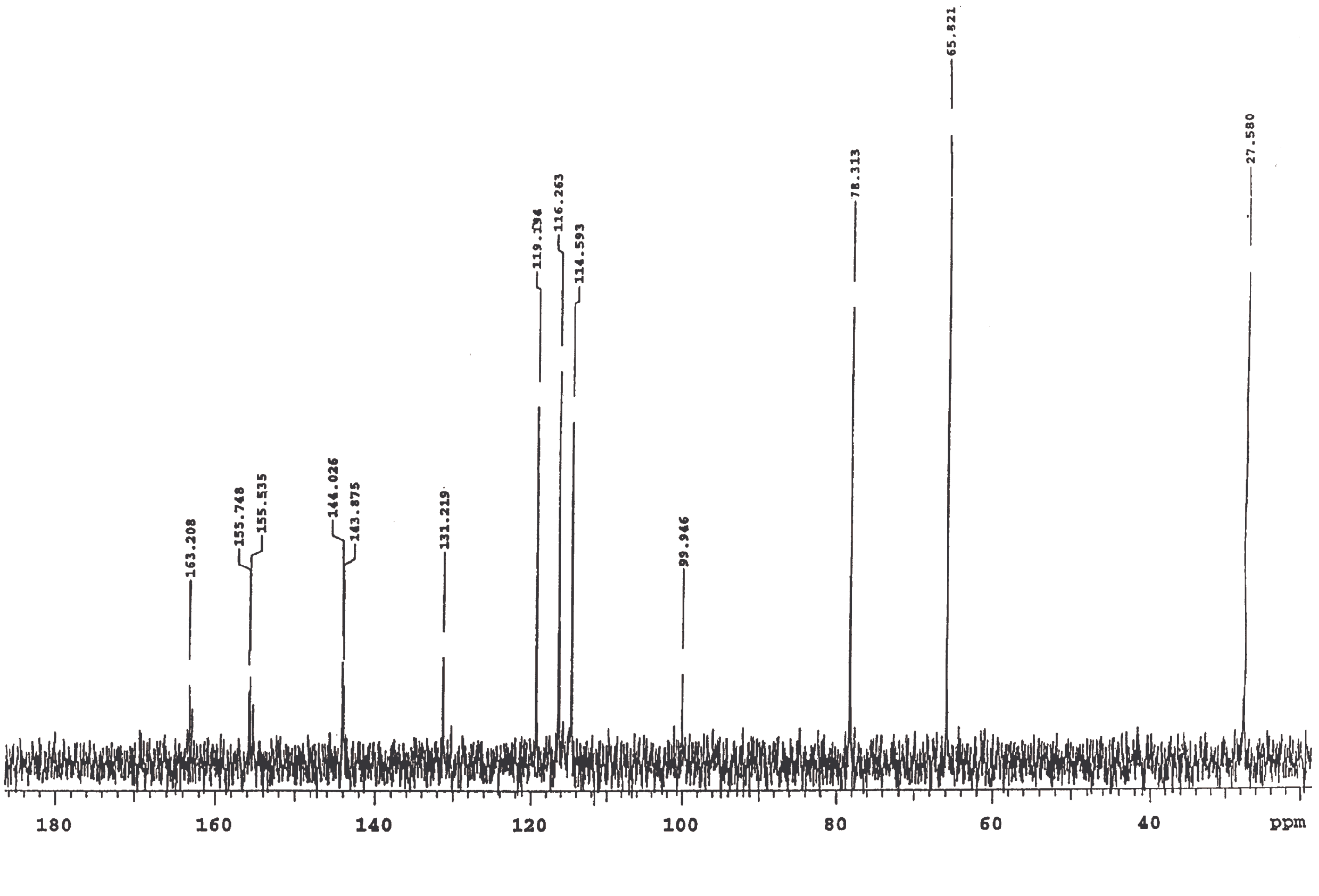

Fig. S2i. ^{13}C NMR spectra of PTI-777-compound j in D_2O with 0.1% trifluoroacetic acid (TFA).

Fig. S2j

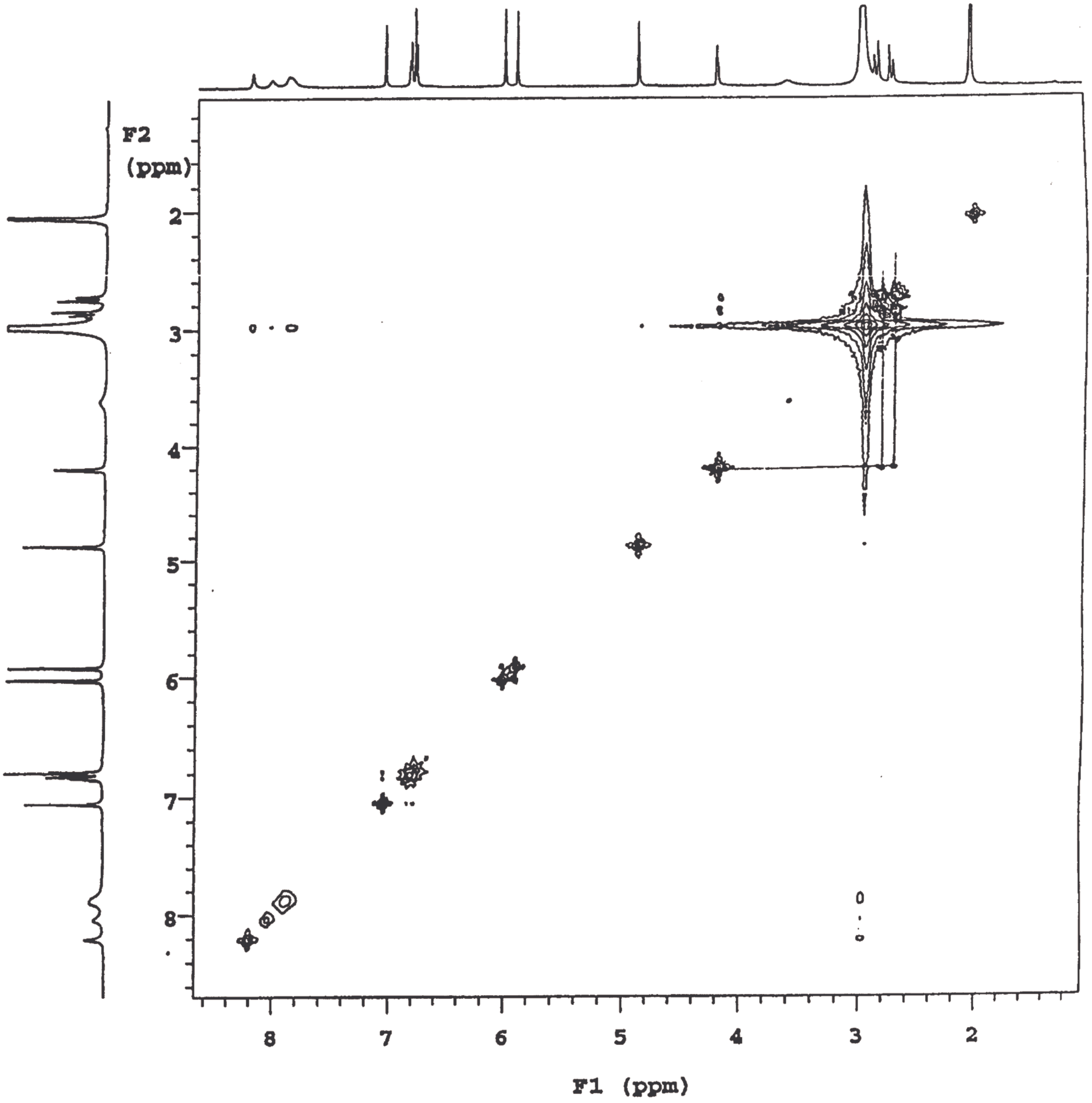

Fig. S2j. Correlation spectroscopy (COSY) of PTI-777-compound j in acetified d_6 acetone.

Fig. S2k

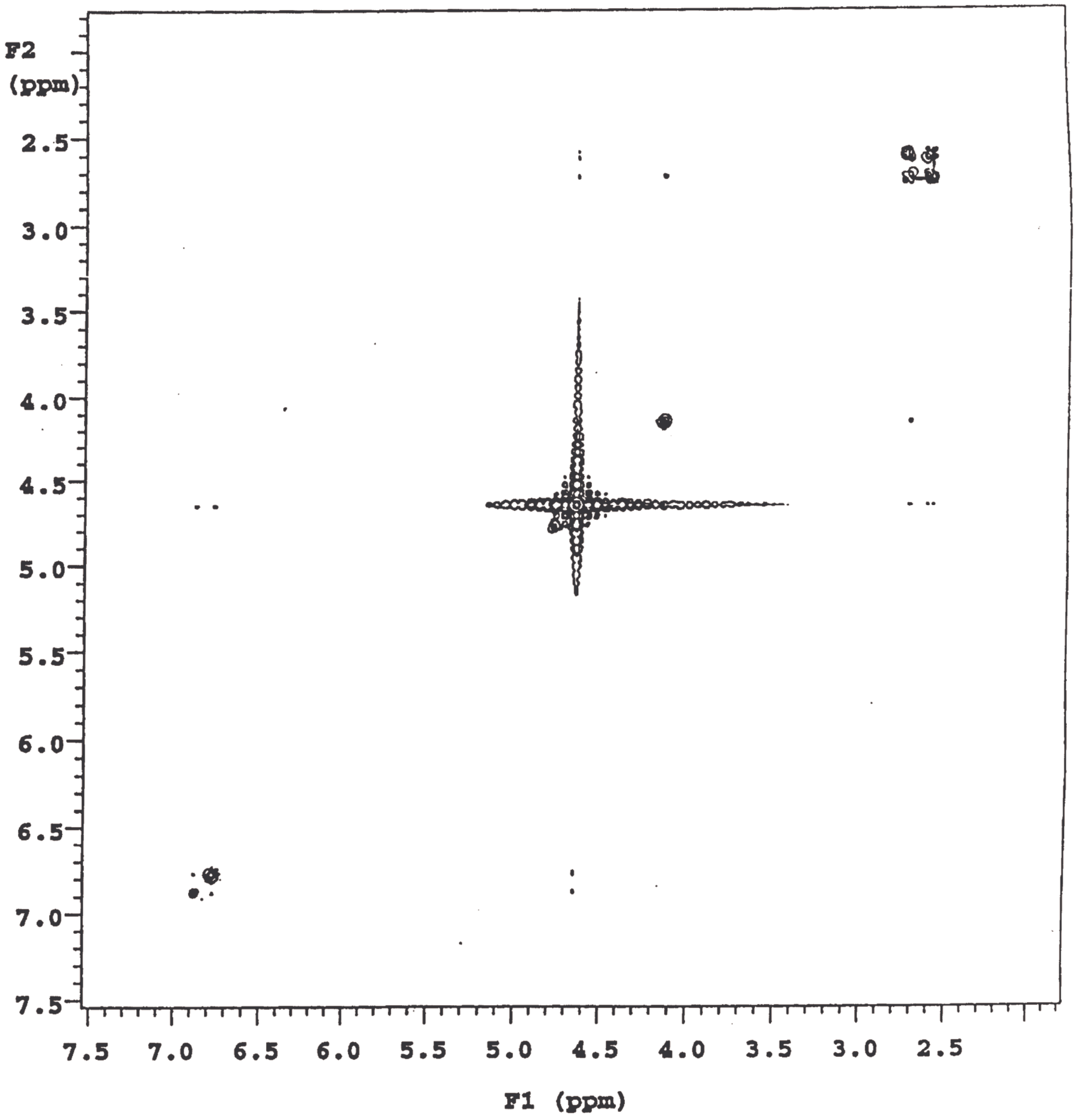

Fig. S2k. COSY of PTI-777-compound j in acetified D_2O.

Fig. S2l

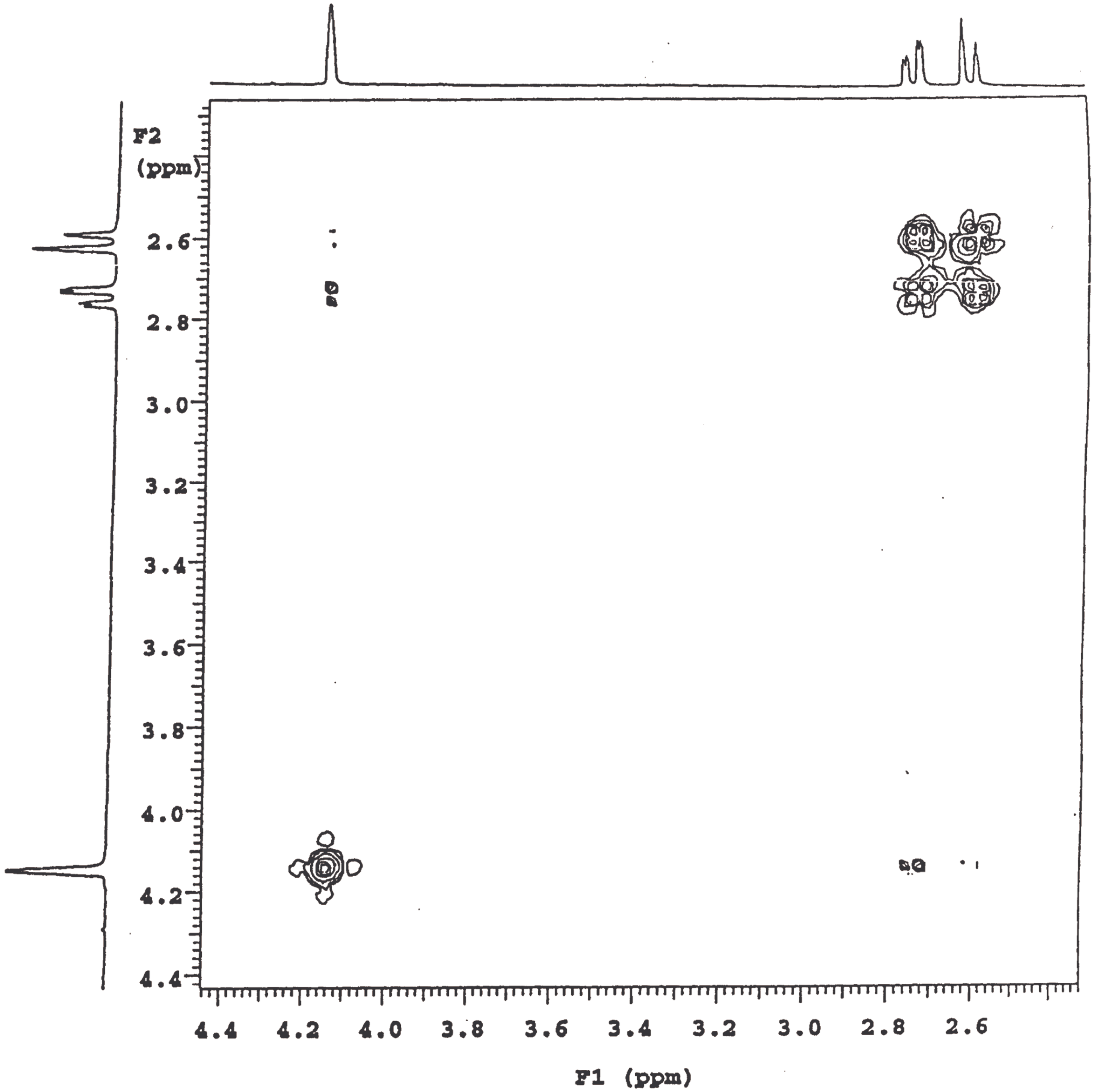

Fig. S2l. Enlargement of the upper right quadrant of the COSY image shown in Fig. S2k.

Fig. S2m

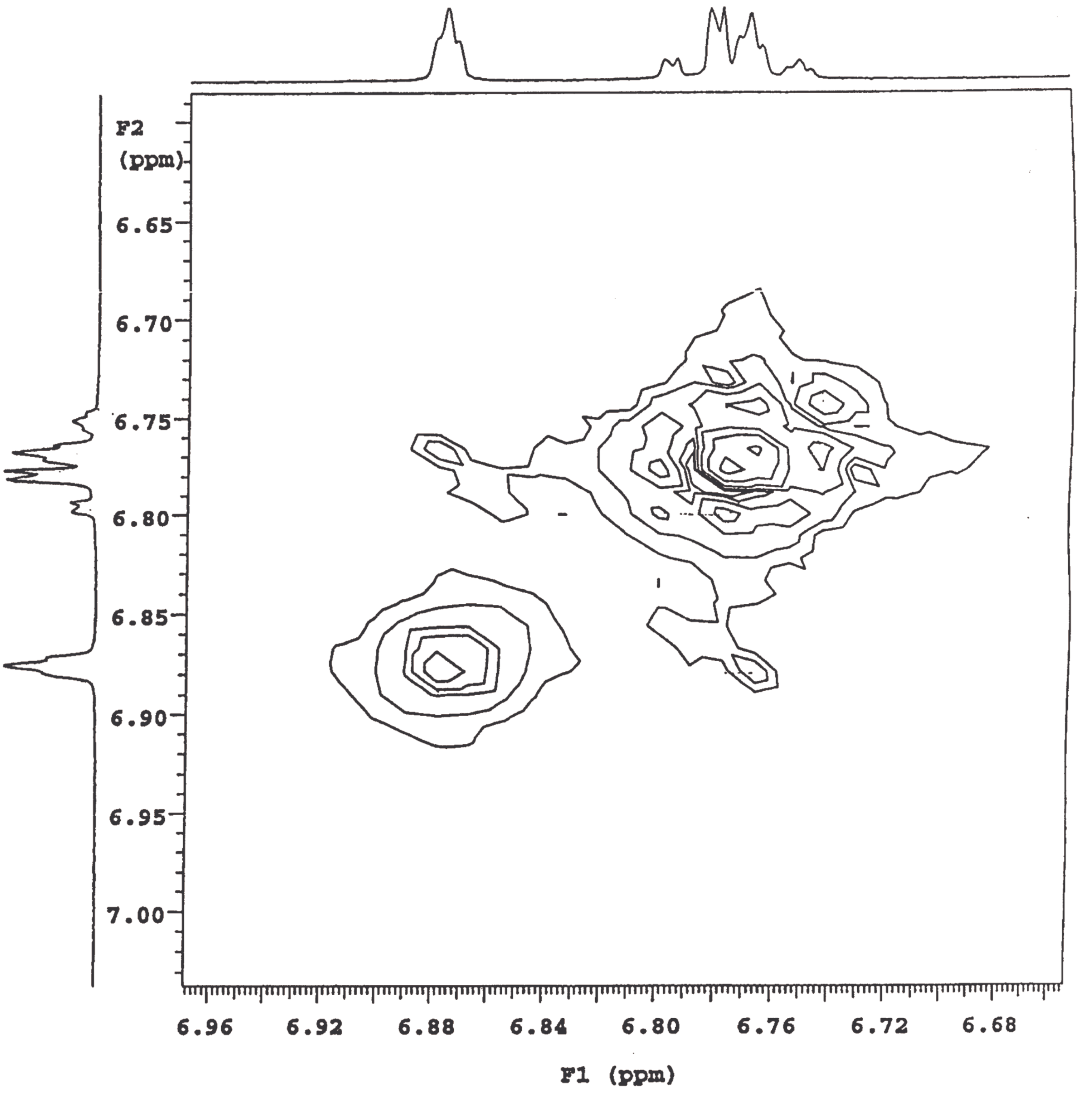

Fig. S2m. Enlargement of the lower left quadrant of the COSY image shown in Fig. S2k.

Fig. S2n

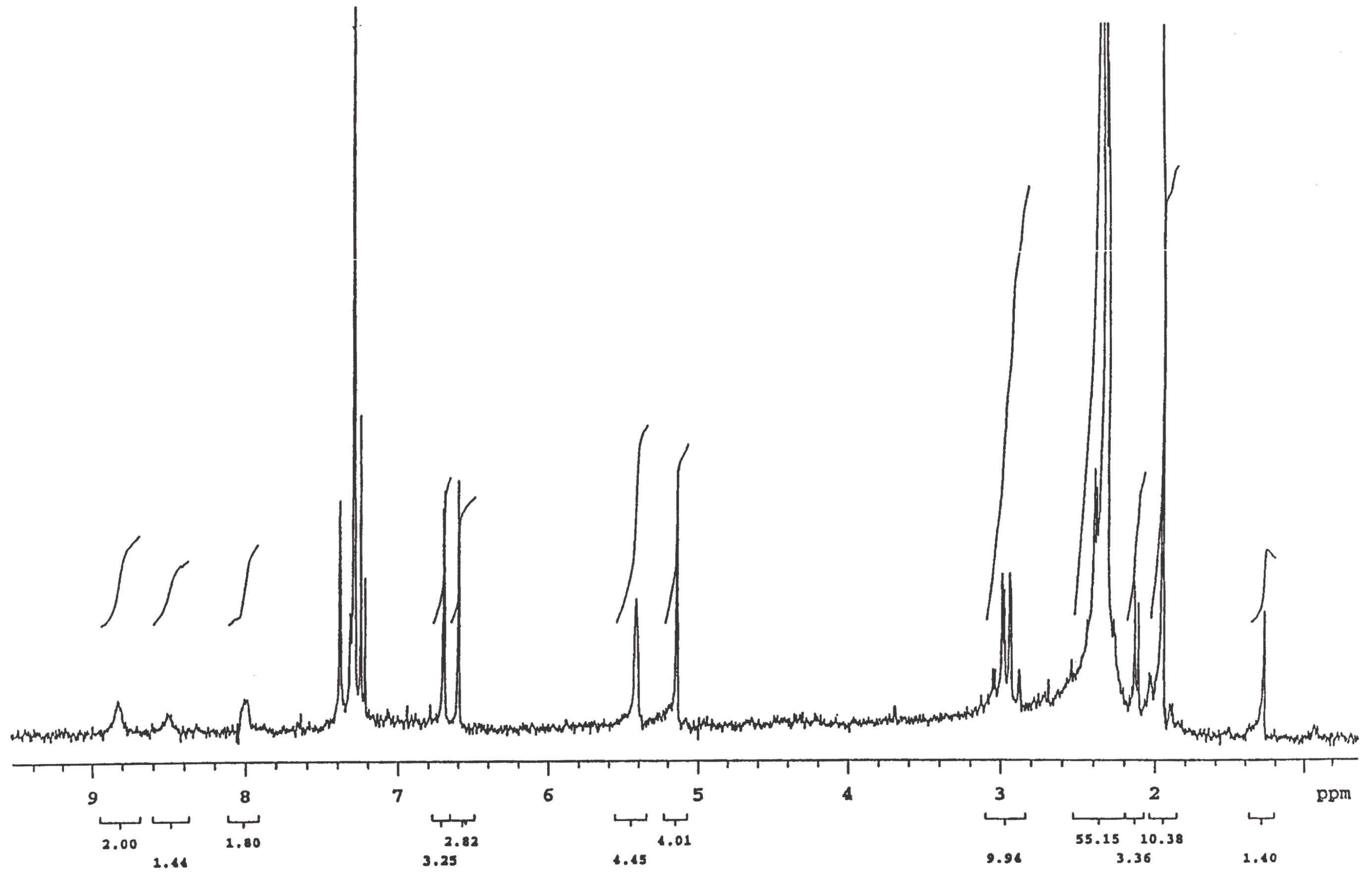

Fig. S2n. ^{1}H NMR spectra of PTI-777-compound j that had been acetylated.

Fig. S2o

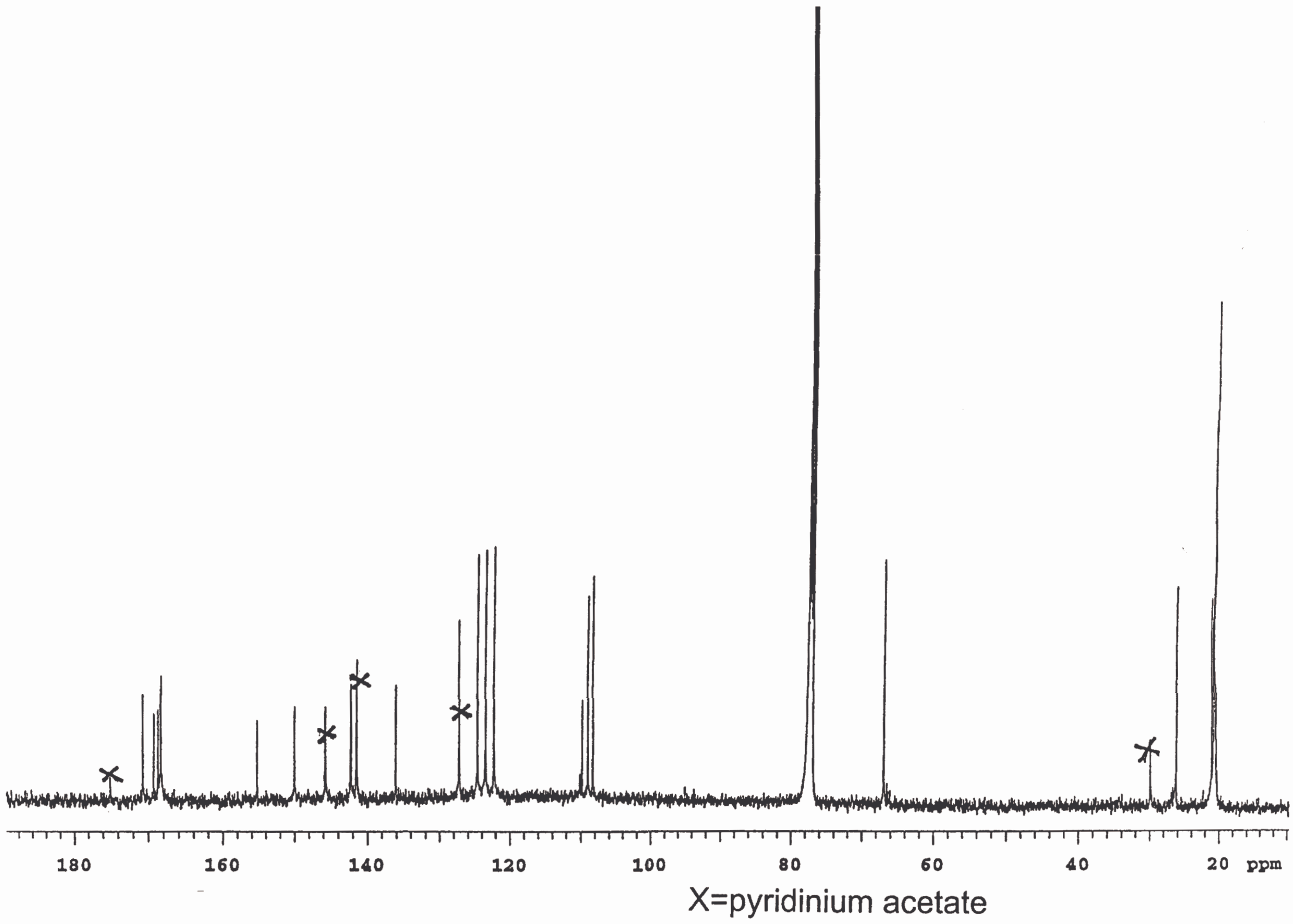

Fig. S2o. ^{13}C NMR spectra of PTI-777-compound j that had been acetylated.

Fig. S2p

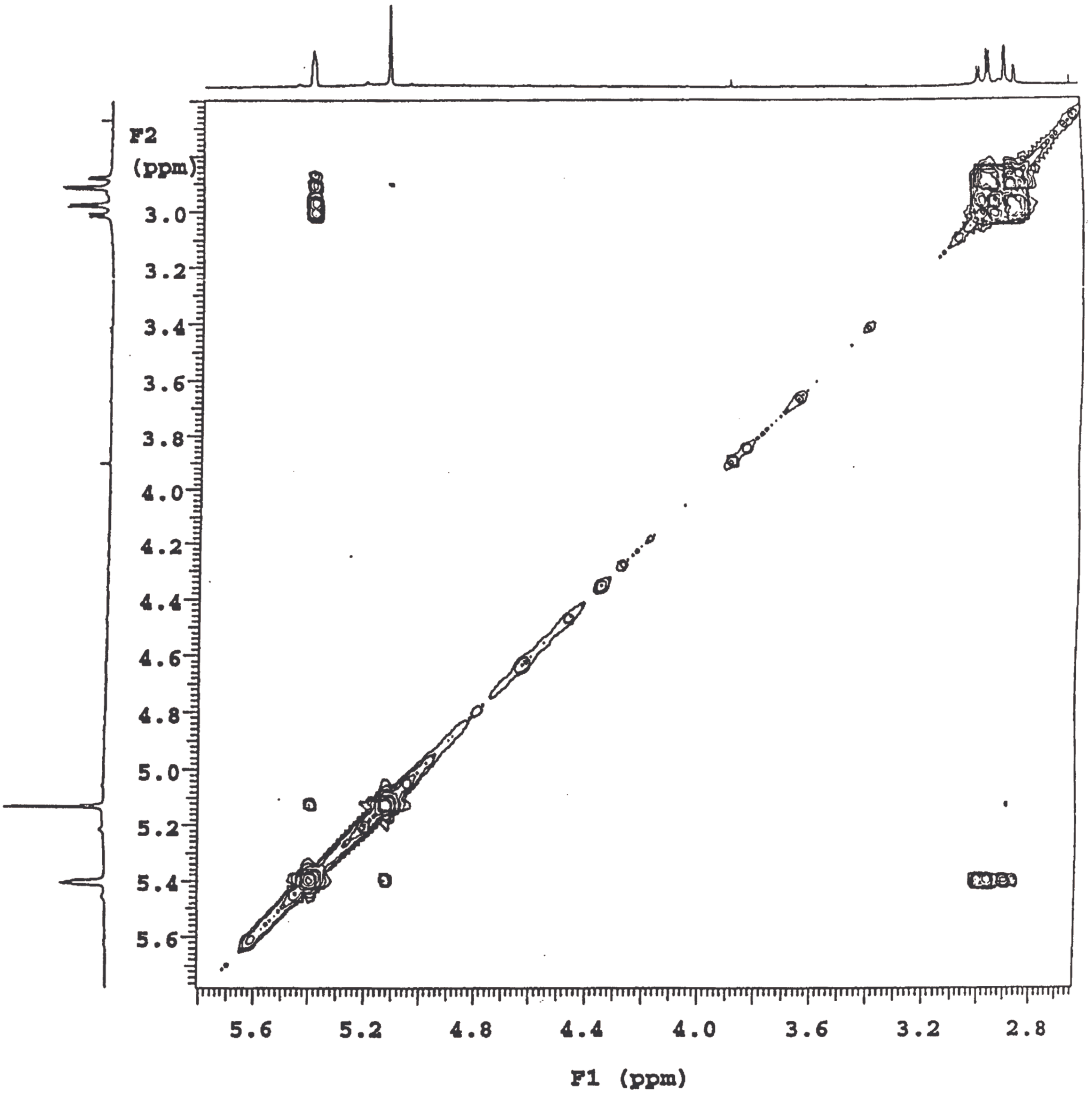

Fig. S2p. COSY of pentaacetate derivative of PTI-777 compound j.

Fig. S2q

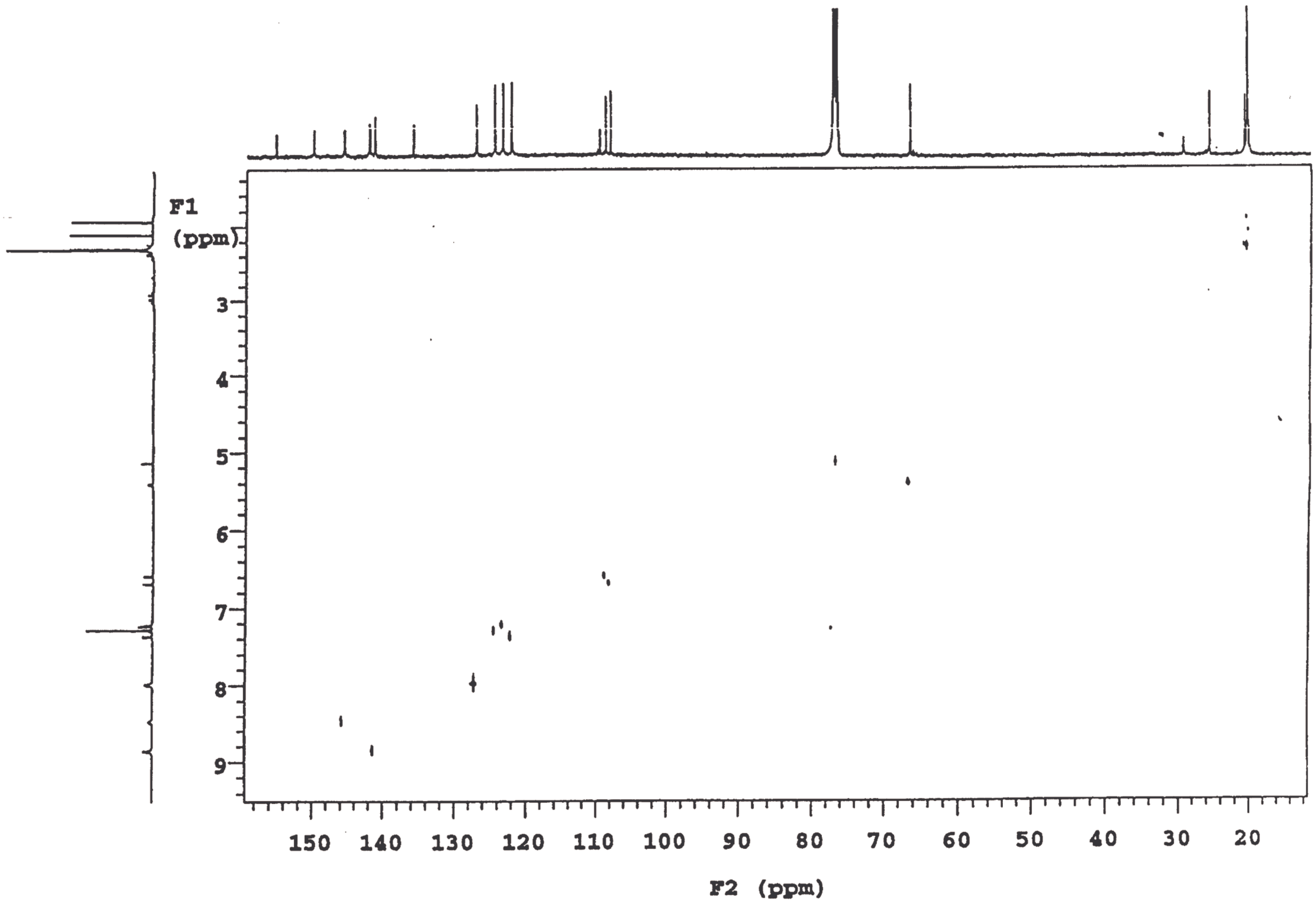

Fig. S2q. Heteronuclear correlation spectroscopy (HETCOR) of the pentaacetate derivative of PTI-777 compound j.

Fig. S2r

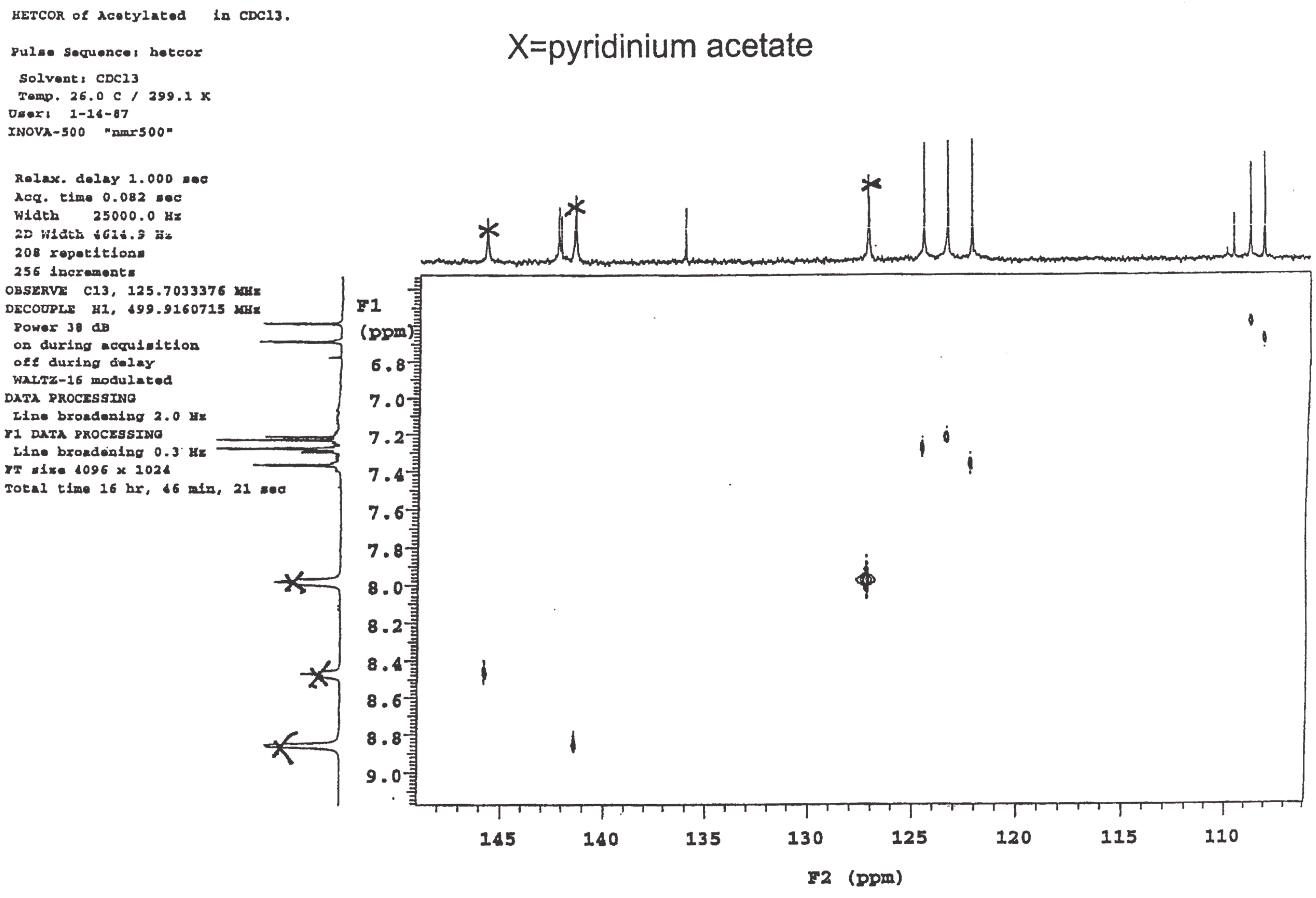

Fig. S2r. Enlargement of the aromatic region of the HETCOR spectra of the pentaacetate derivative of PTI-777 compound j shown in Fig. S2q.

Fig. S2s

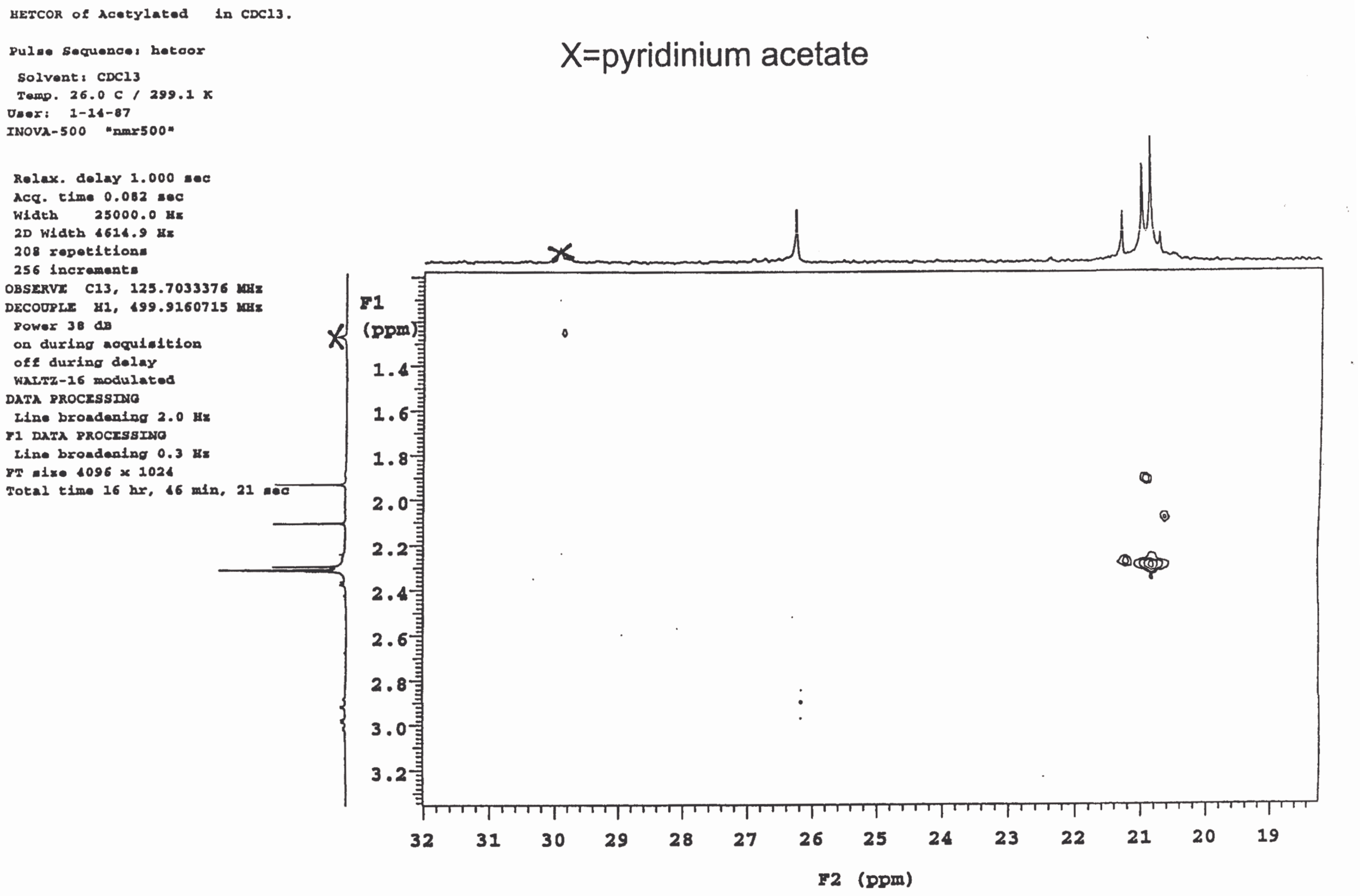

Fig. S2s. Enlargement of the methyl region of the HECTOR spectra of the pentaacetate derivative of PTI-777 compound j shown in Fig. S2q.

Fig. S2t

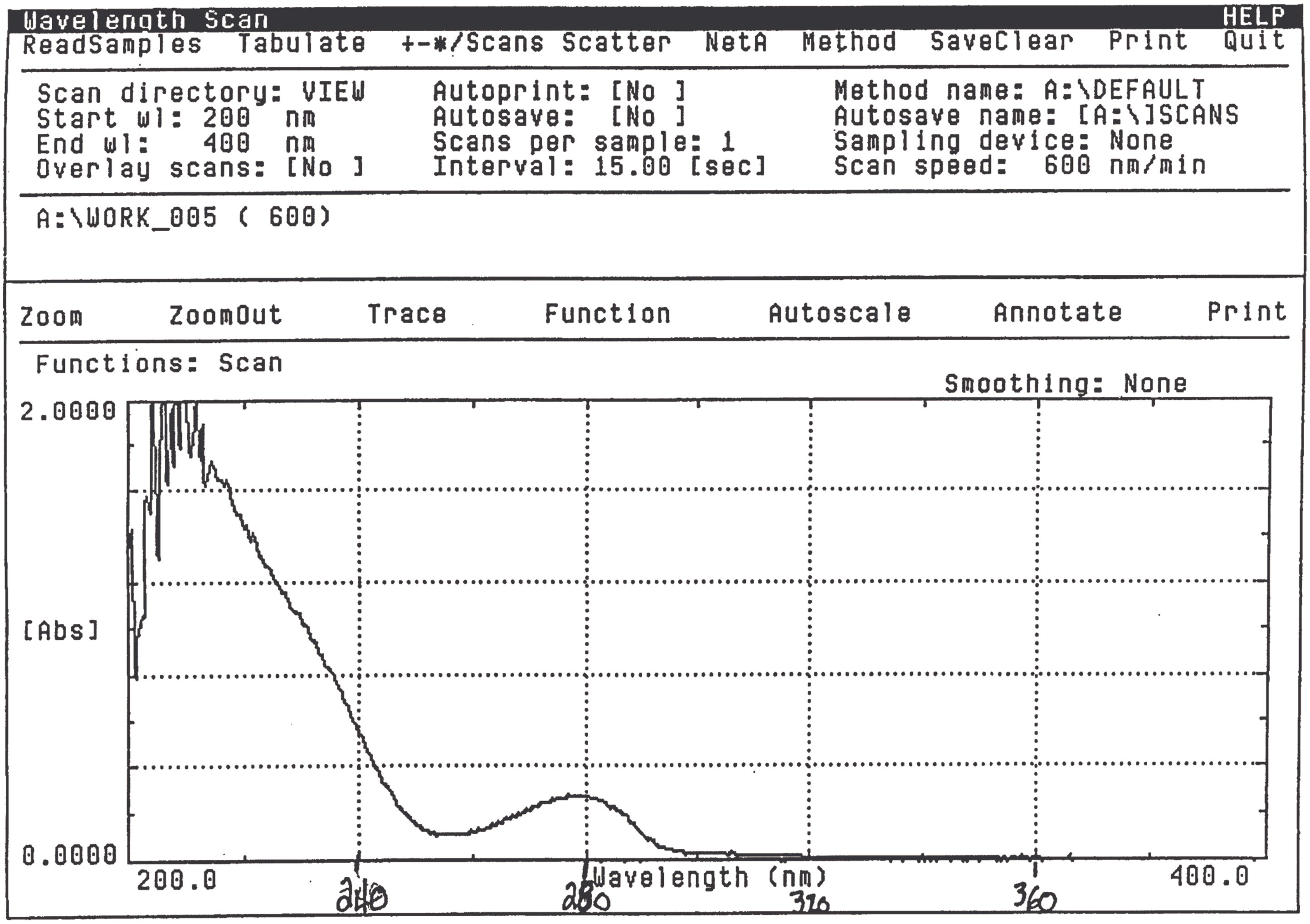

Fig. S2t. Ultraviolet scan of PTI-777 compound j.

Fig. S2u

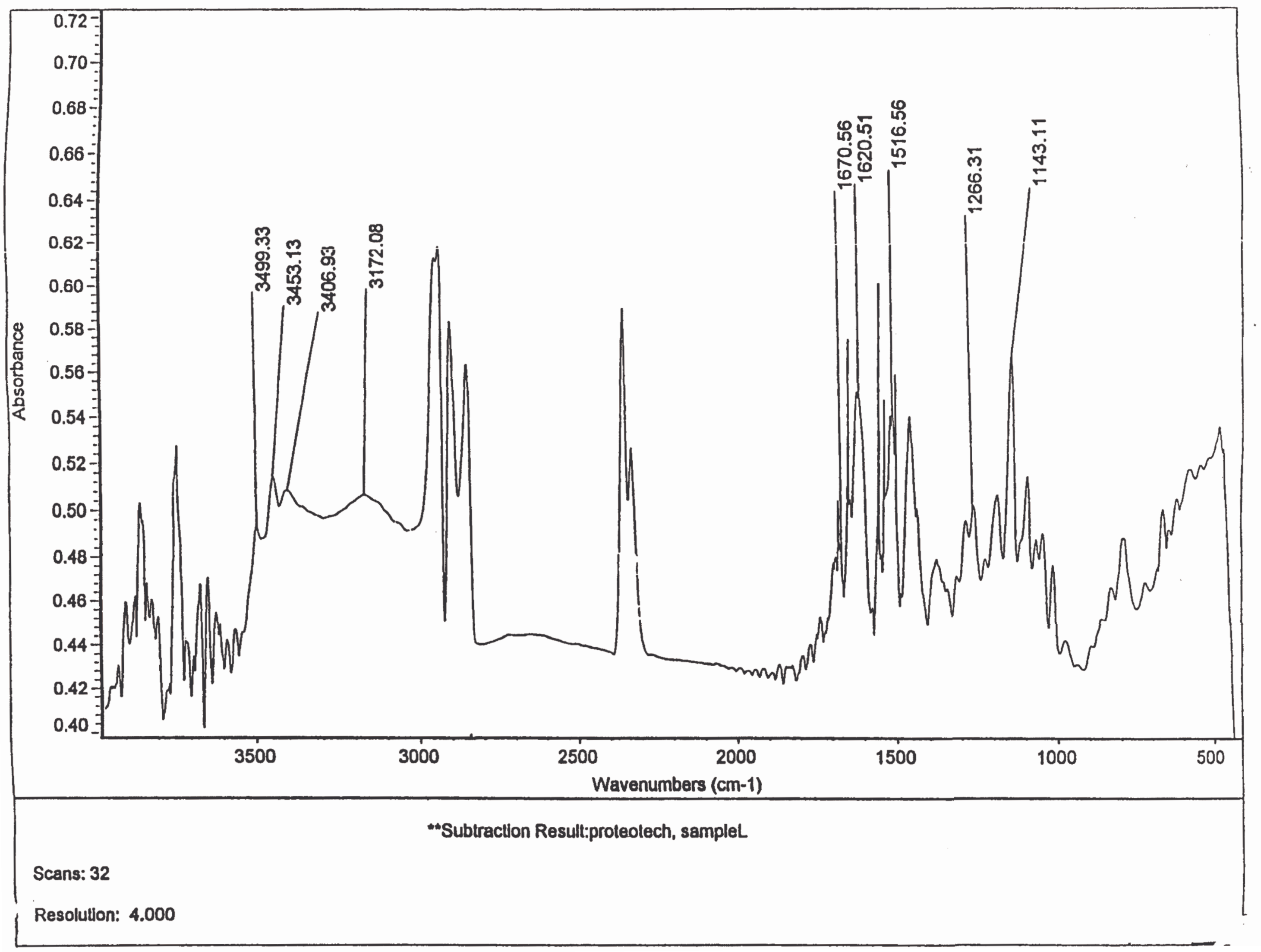

Fig. S2u. Infrared spectra of PTI-777 compound j.

Fig. S2v

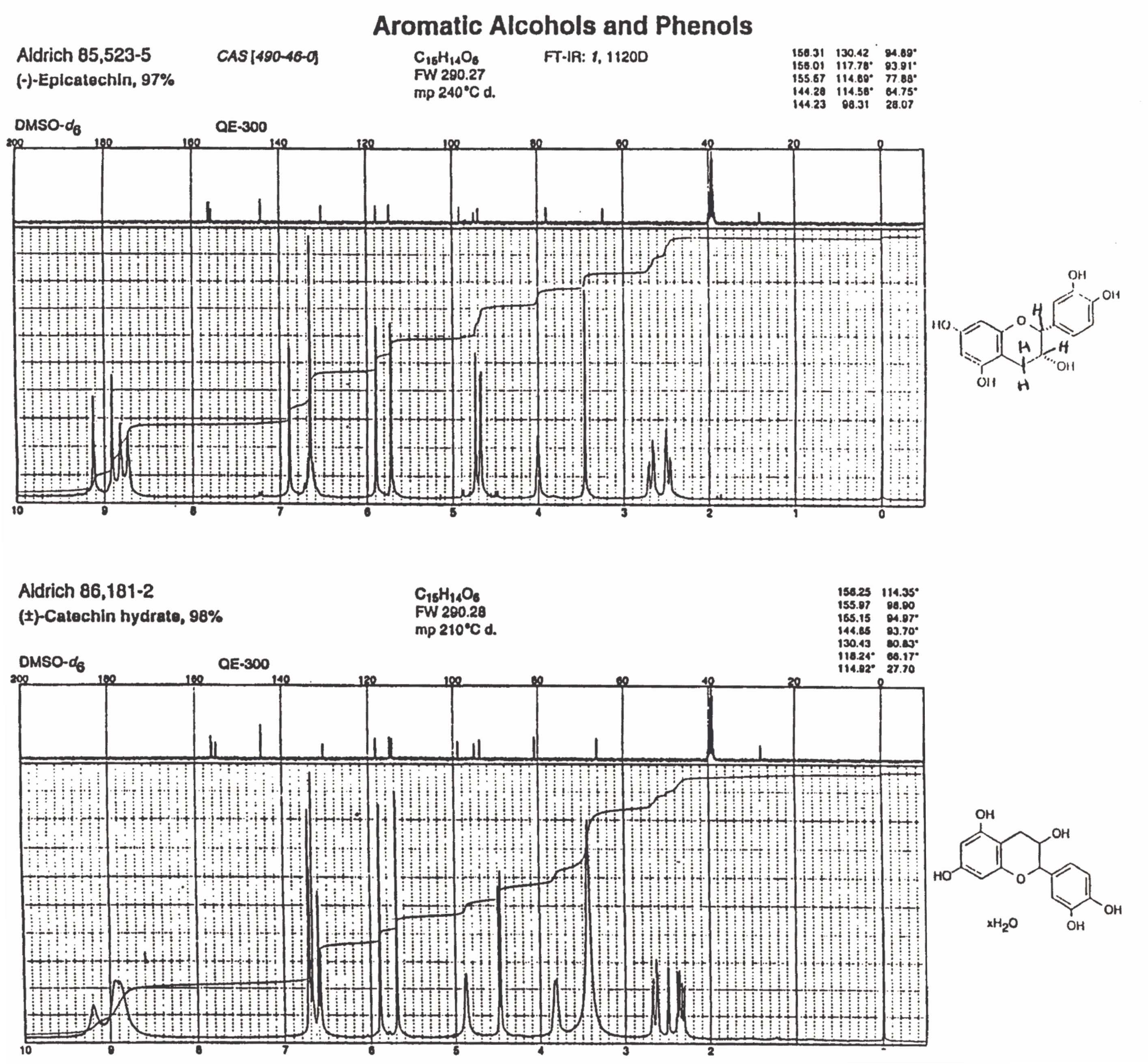

Fig. S2v. Published NMR spectra (Aldrich) of epicatechin and catechin hydrate.

Fig. S2w

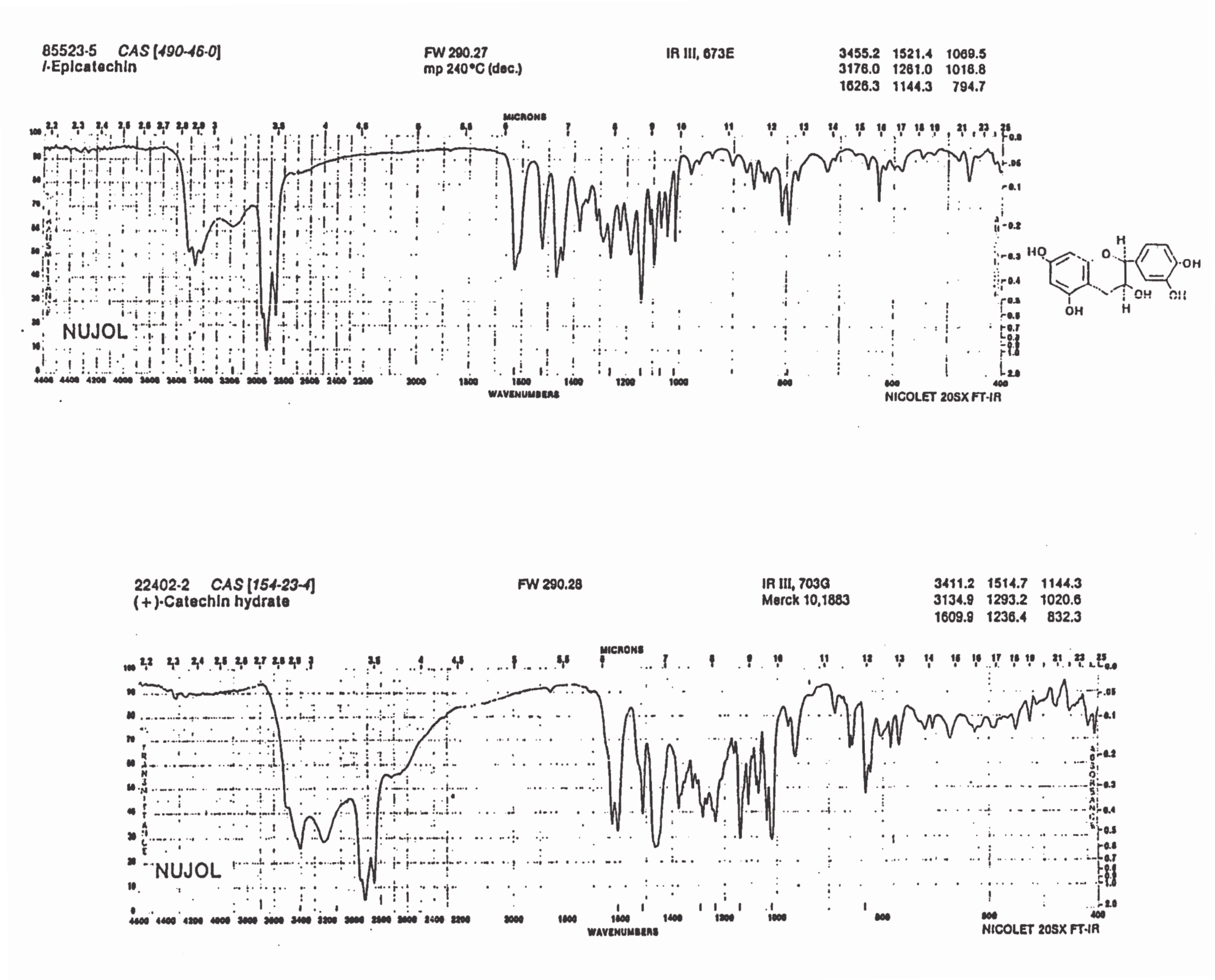

Fig. S2w. Published infrared spectra (Aldrich) of epicatechin and catechin hydrate.

Fig. S3a

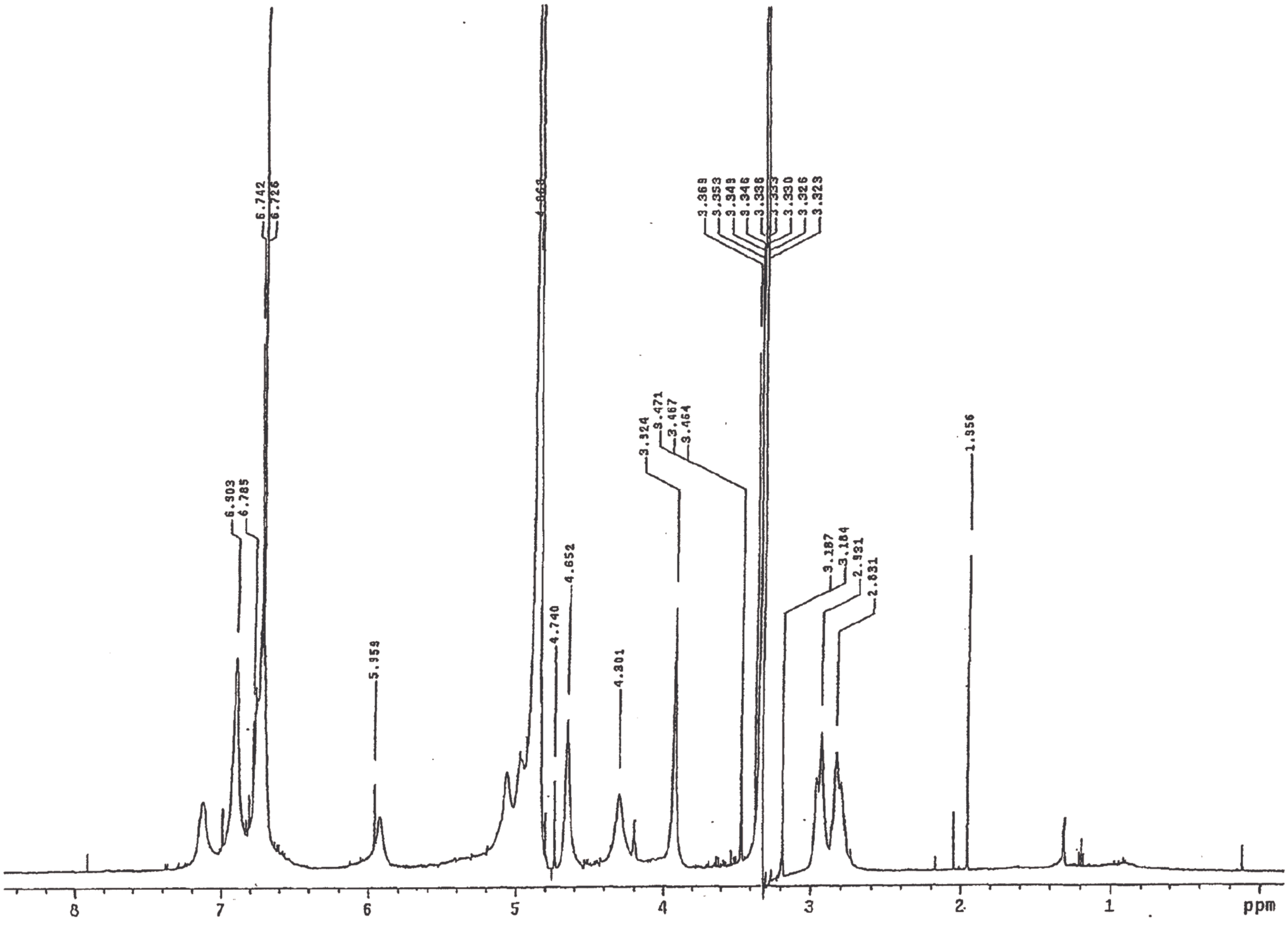

Fig. S3a. ^{1}H NMR spectra of PTI-777 compound h2.

Fig. S3b

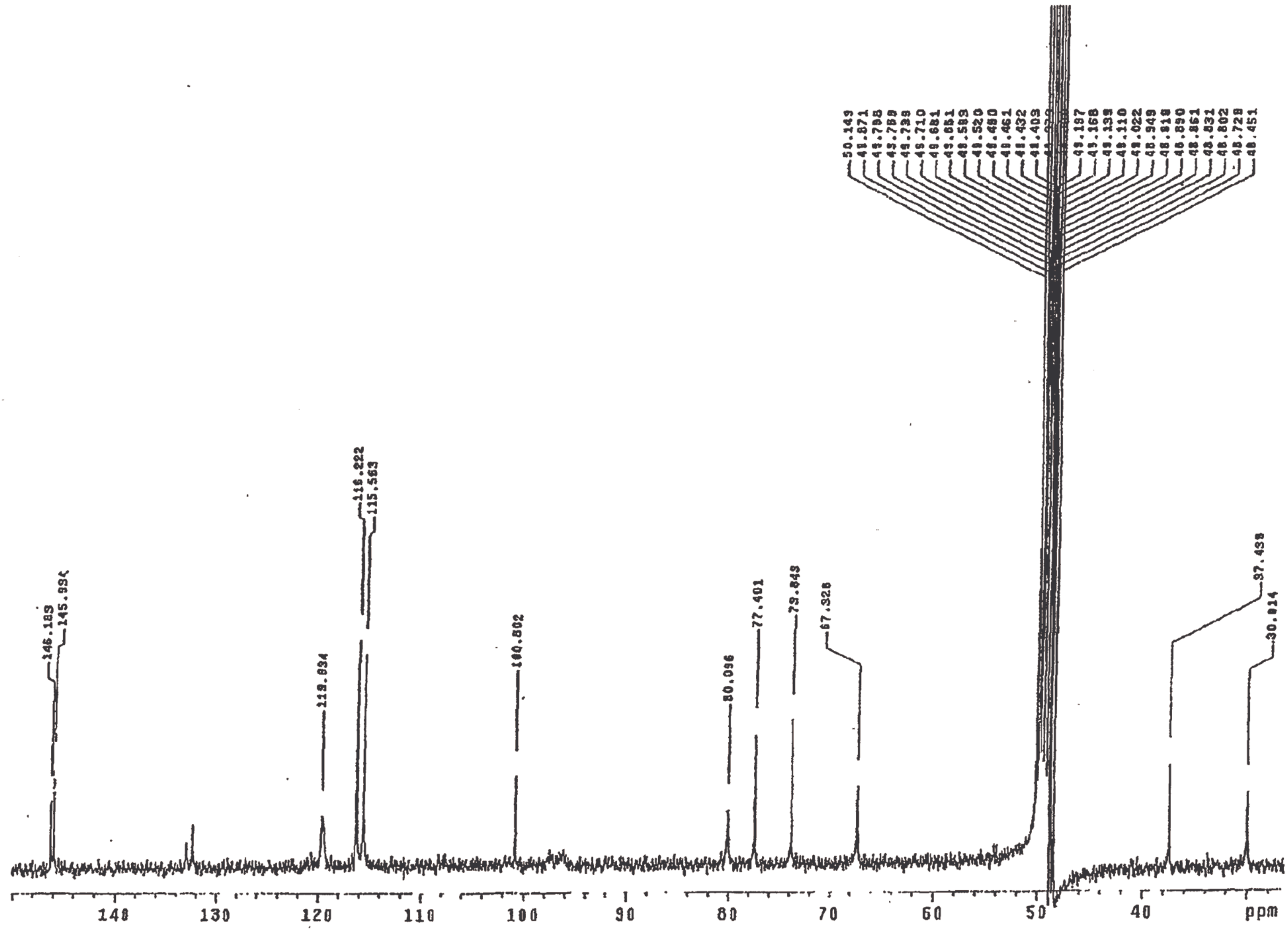

Fig. S3b. ^{13}C NMR spectra of PTI-777compound h2.

Fig. S3c

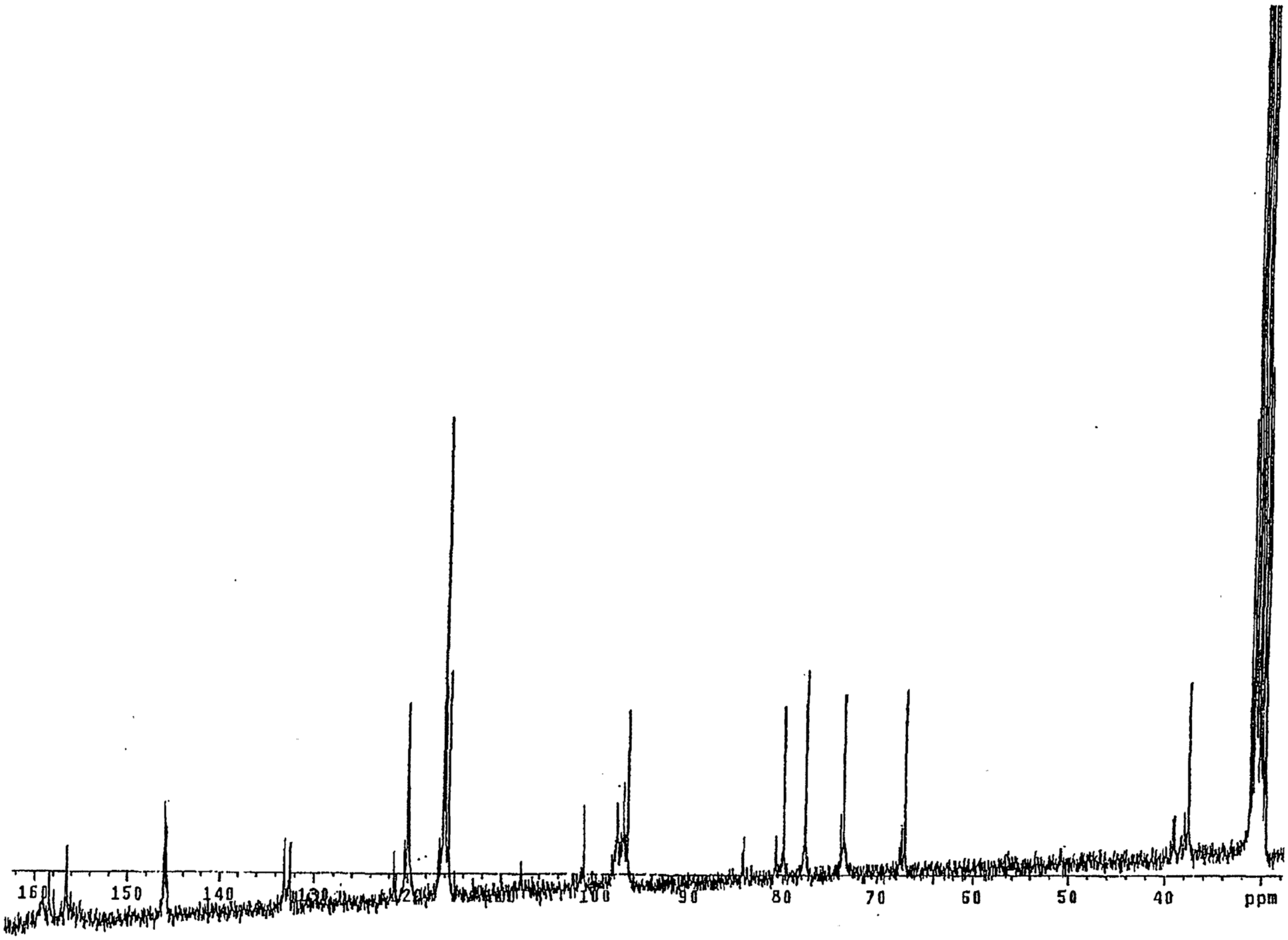

Fig. S3c. ^{13}C NMR spectra of PTI-777-compound h2 in deuteroacetone.

Fig. S3d

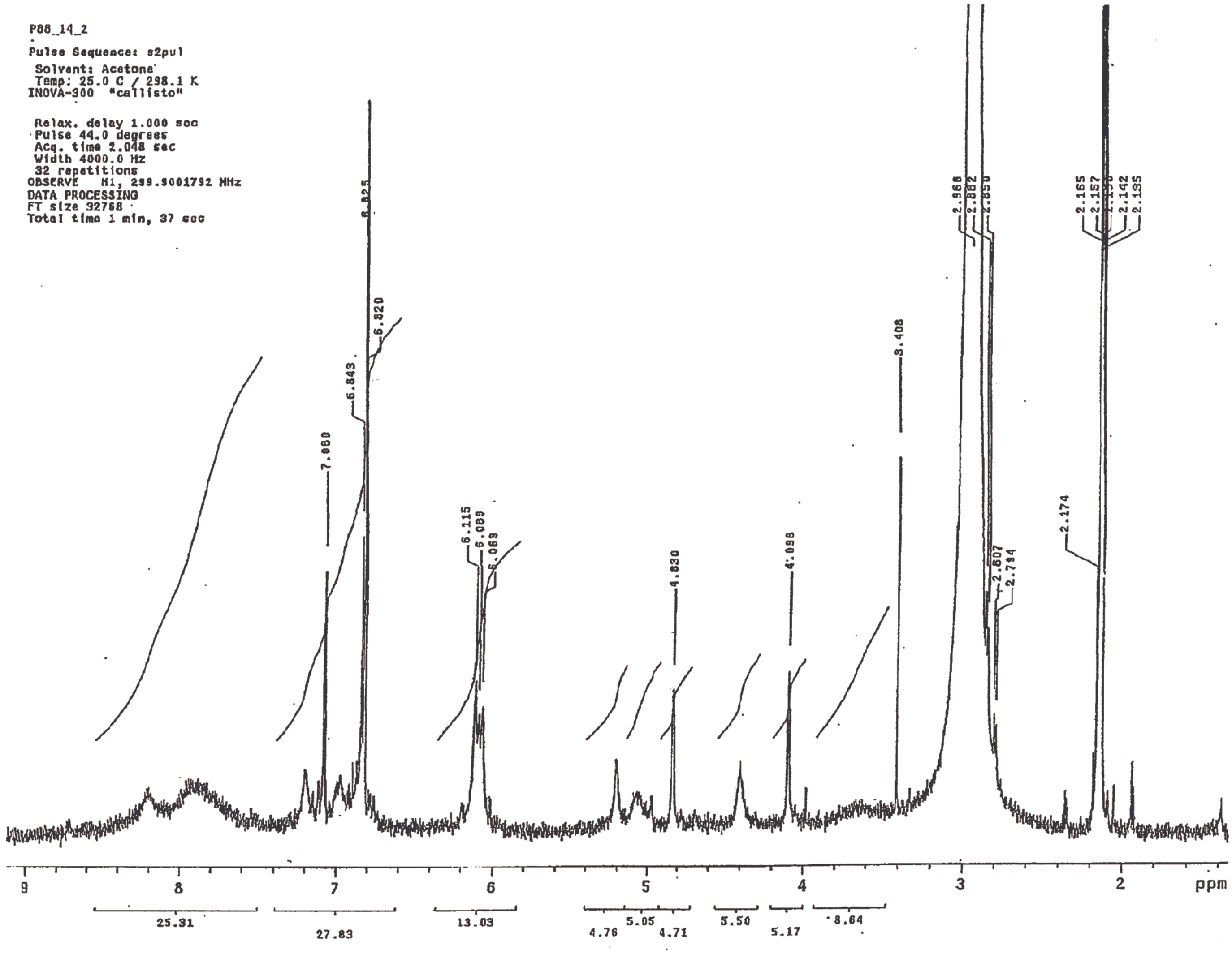

Fig. S3d. ^{1}H NMR spectra of PTI-777-compound h2 in deteroacetone.

Fig. S3e

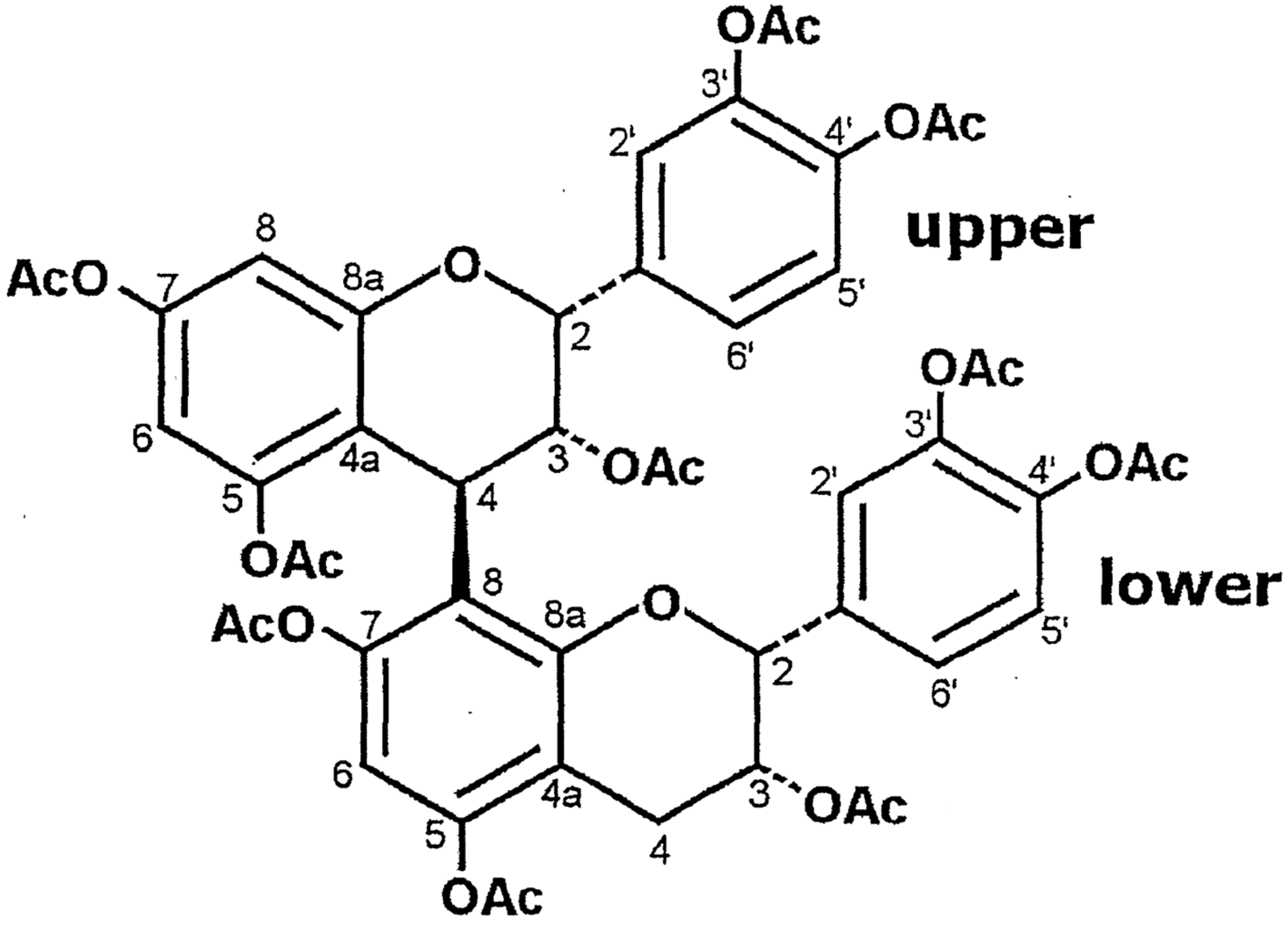

Fig. S3e. Postulated peracetate structure of acetylated PTI-777 compound h2 (acetylated proanthocyanidin B2).

Fig. S3f

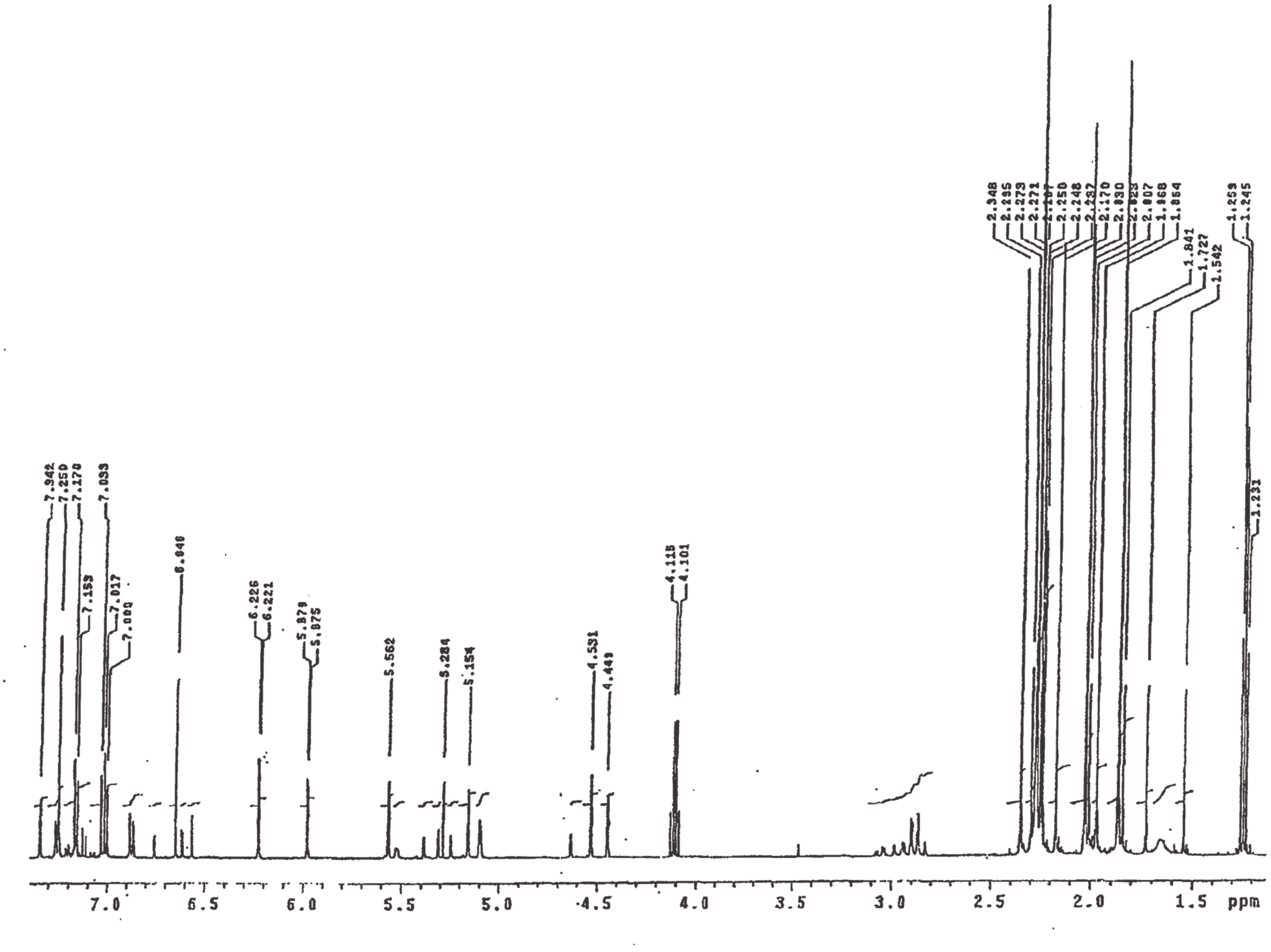

Fig. S3f. ^{1}H NMR spectra of PTI-777 compound h2 peracetate.

Fig. S3g

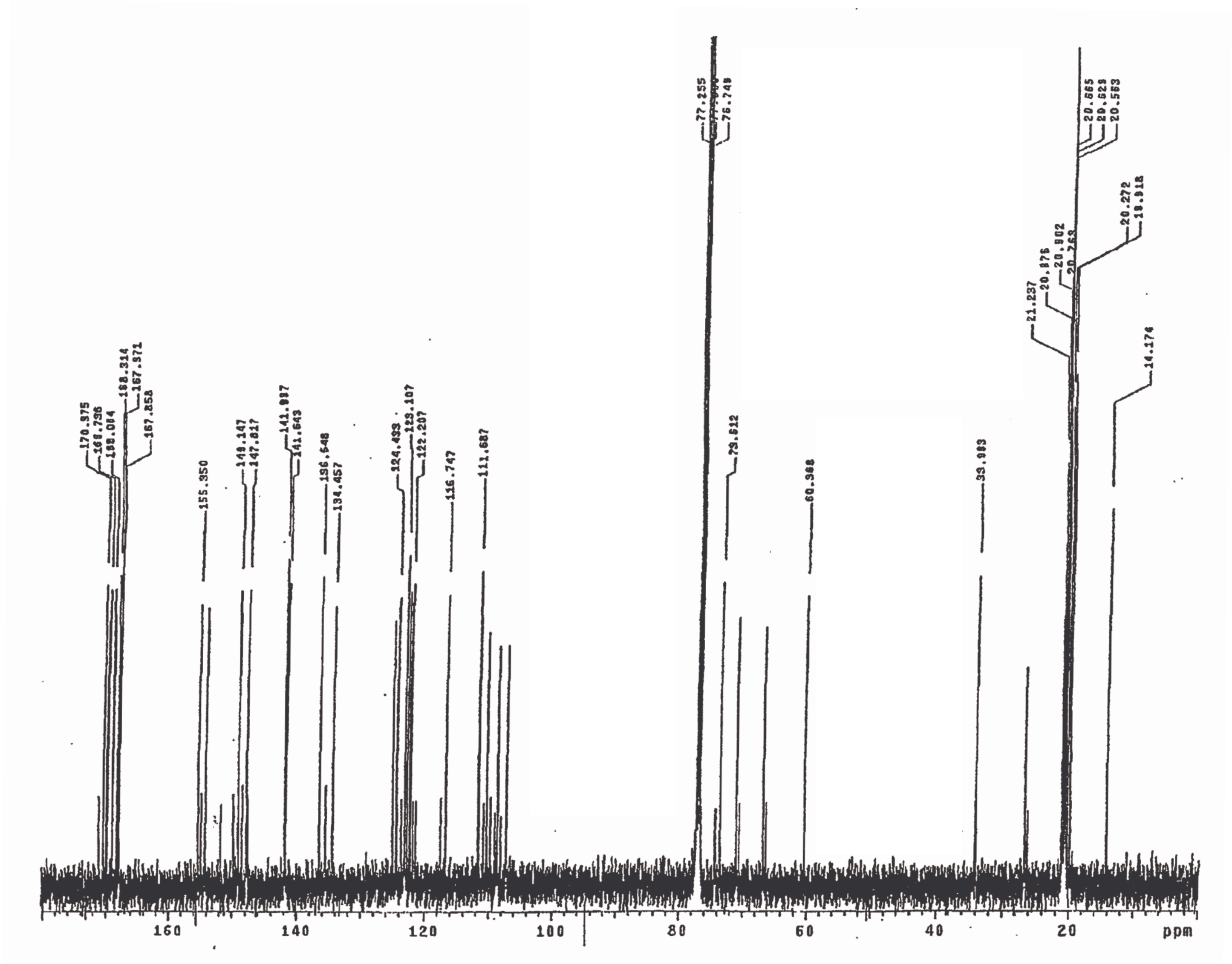

Fig. S3g. ^{13}C NMR spectra of PTI-777-compound h2 peracetate.

Fig. S3h

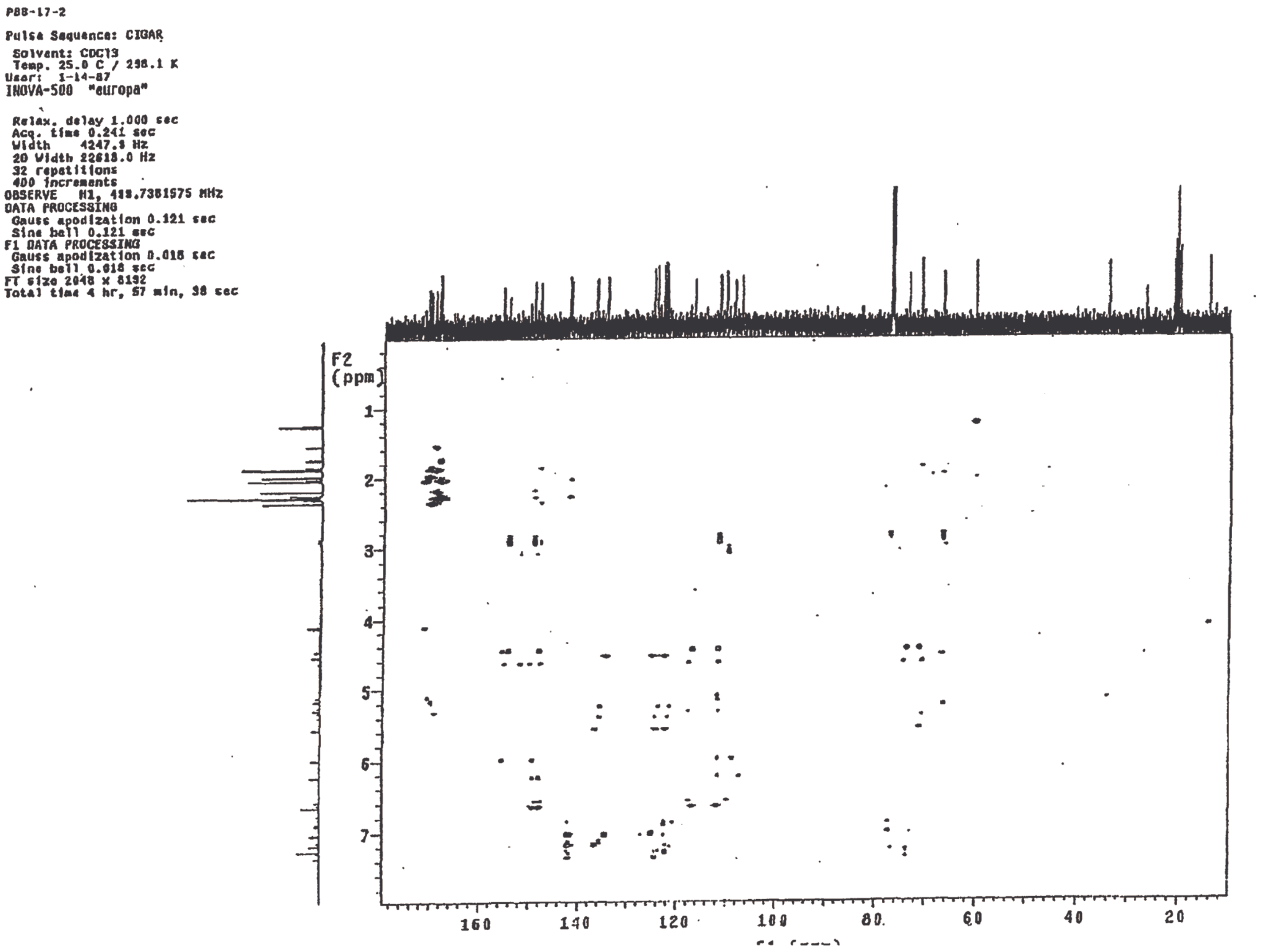

Fig. S3h. A constant time inverse-detection gradient accordion rescaled heteronuclear multiple bond correlation spectroscopy (CIGAR) ^1H-^{13}C correlation spectrum (low resolution) of the h2 peracetate.

Fig. S3i

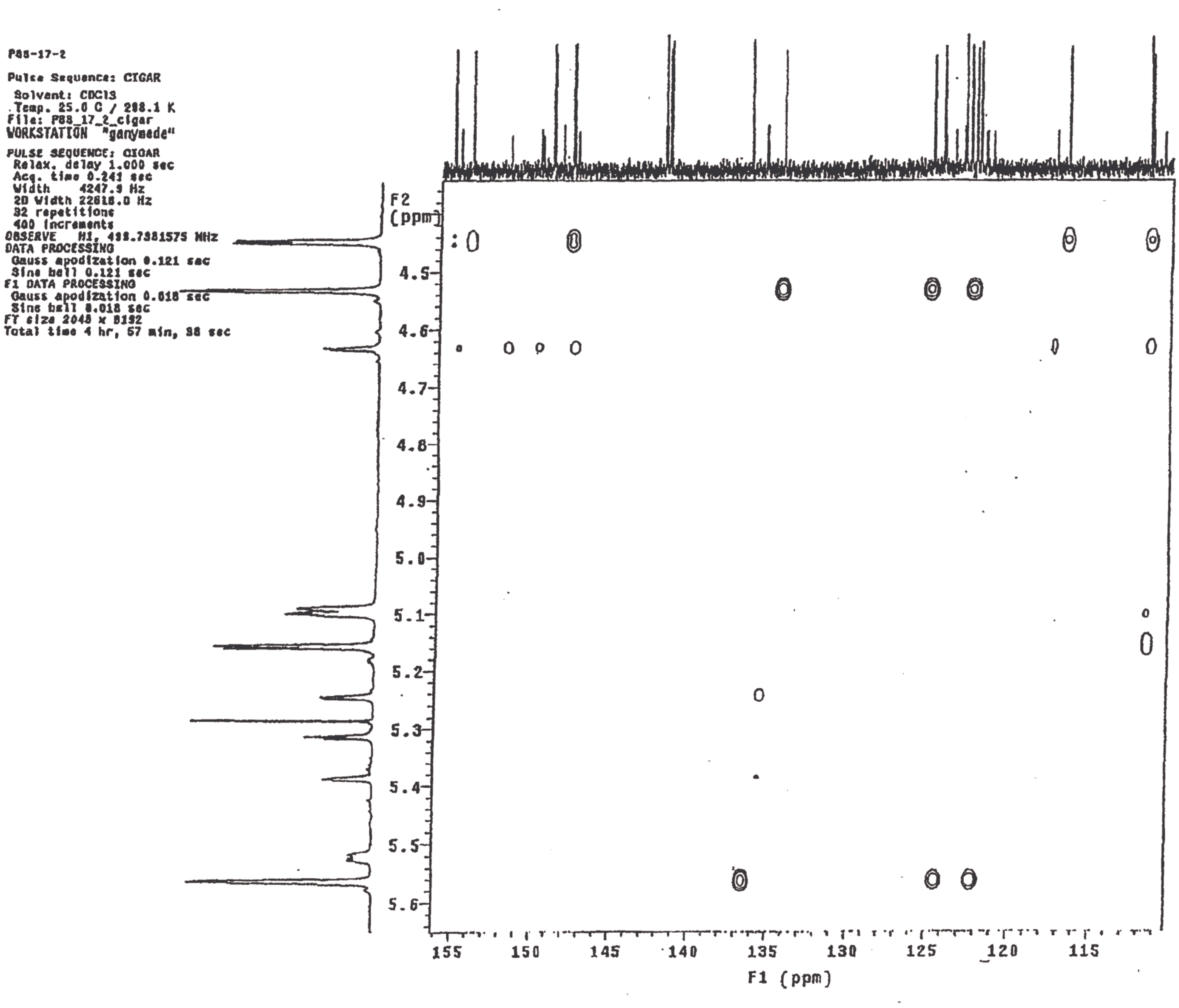

Fig. S3i. High resolution of the constant time inverse-detection gradient accordion rescaled heteronuclear multiple bond correlation spectroscopy (CIGAR) of the h2 peracetate.

Fig. S3j

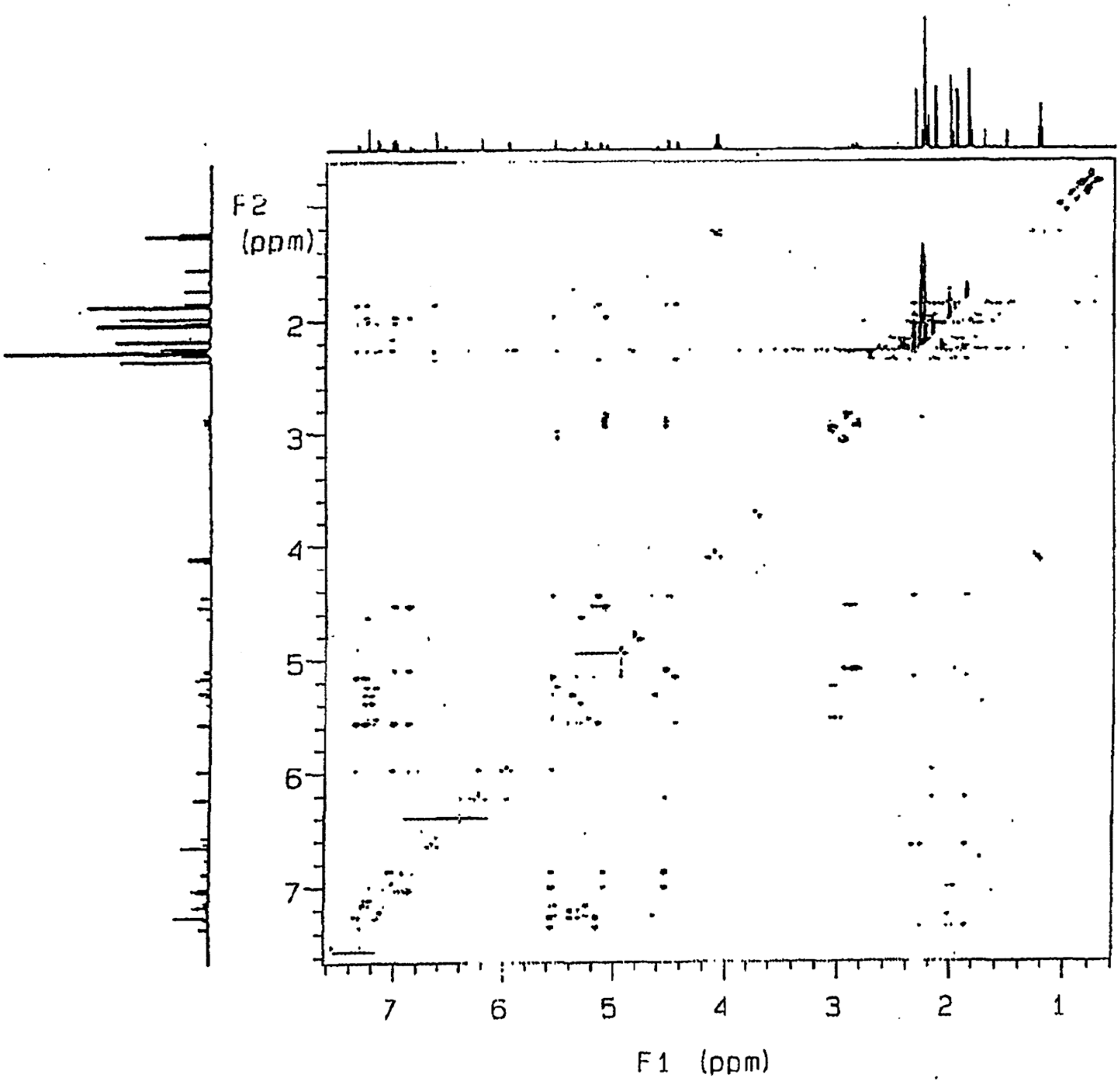

Fig. S3j. Nuclear overhauser effect spectroscopy (NOESY) spectrum of PTI-777-compound h2 peracetate.

Fig. S3k

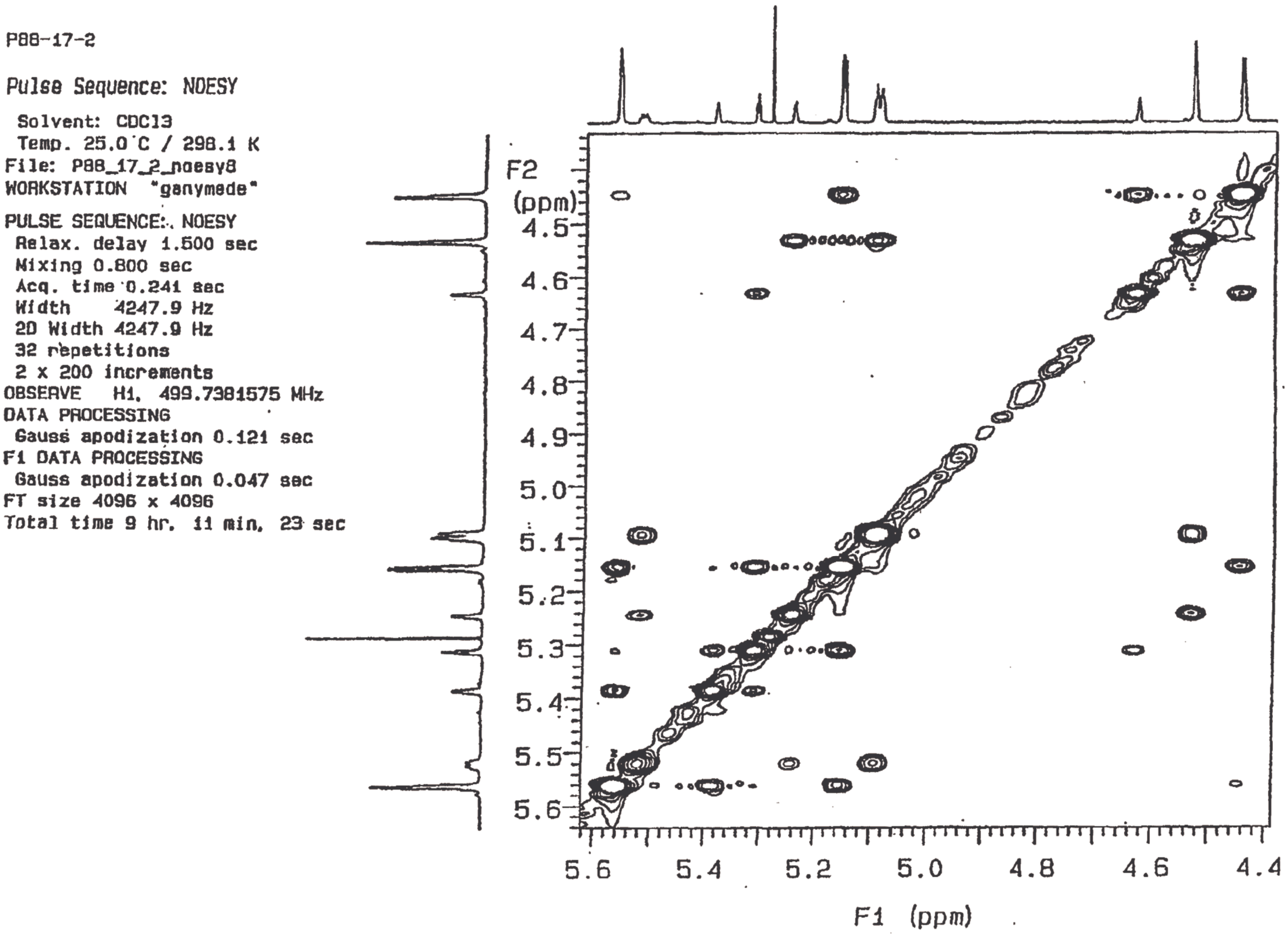

Fig. S3k. Enlargement of quadrant of NOESY spectra of PTI-777 compound h2 peracetate.

Fig. S3I

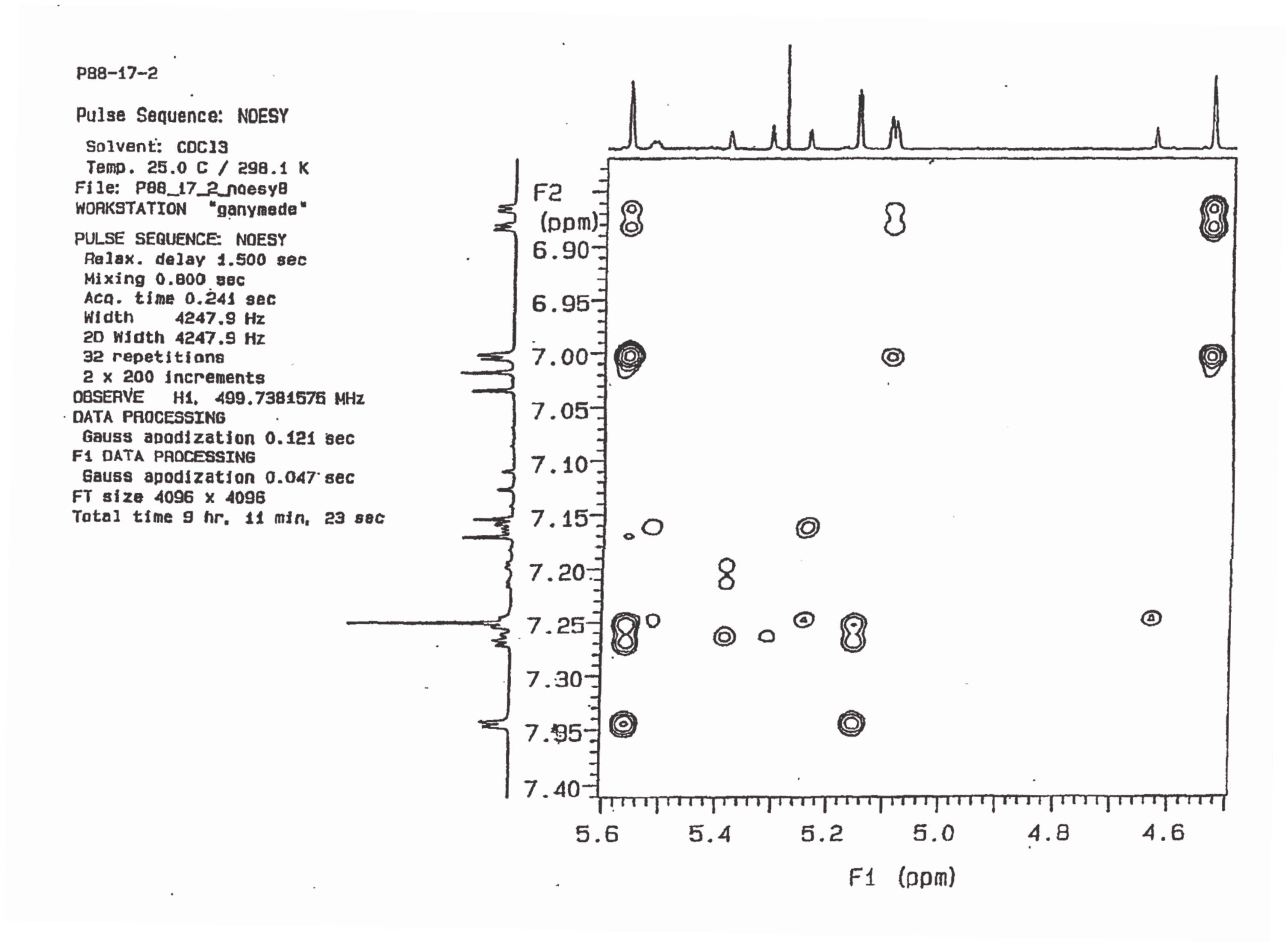

Fig. S3I. Enlargement of quadrant of NOESY spectra of PTI-compound h2 peracetate.

Fig. S3m

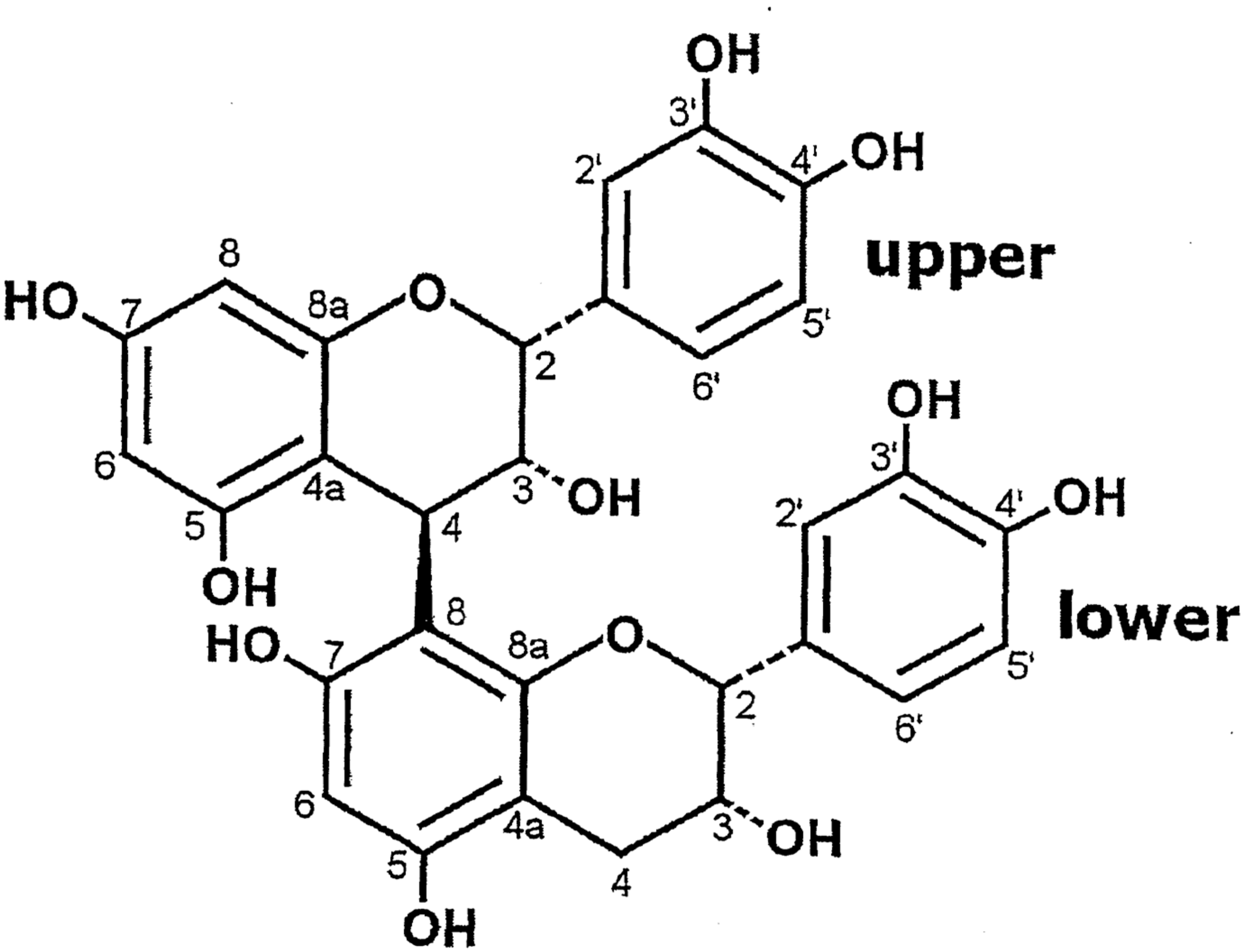

Fig. S3m. Elucidated structure of PTI-777 compound h2 to be epicatechin-4β-8-epicatechin (also known as proanthocyanidin B2).

Fig. S4a

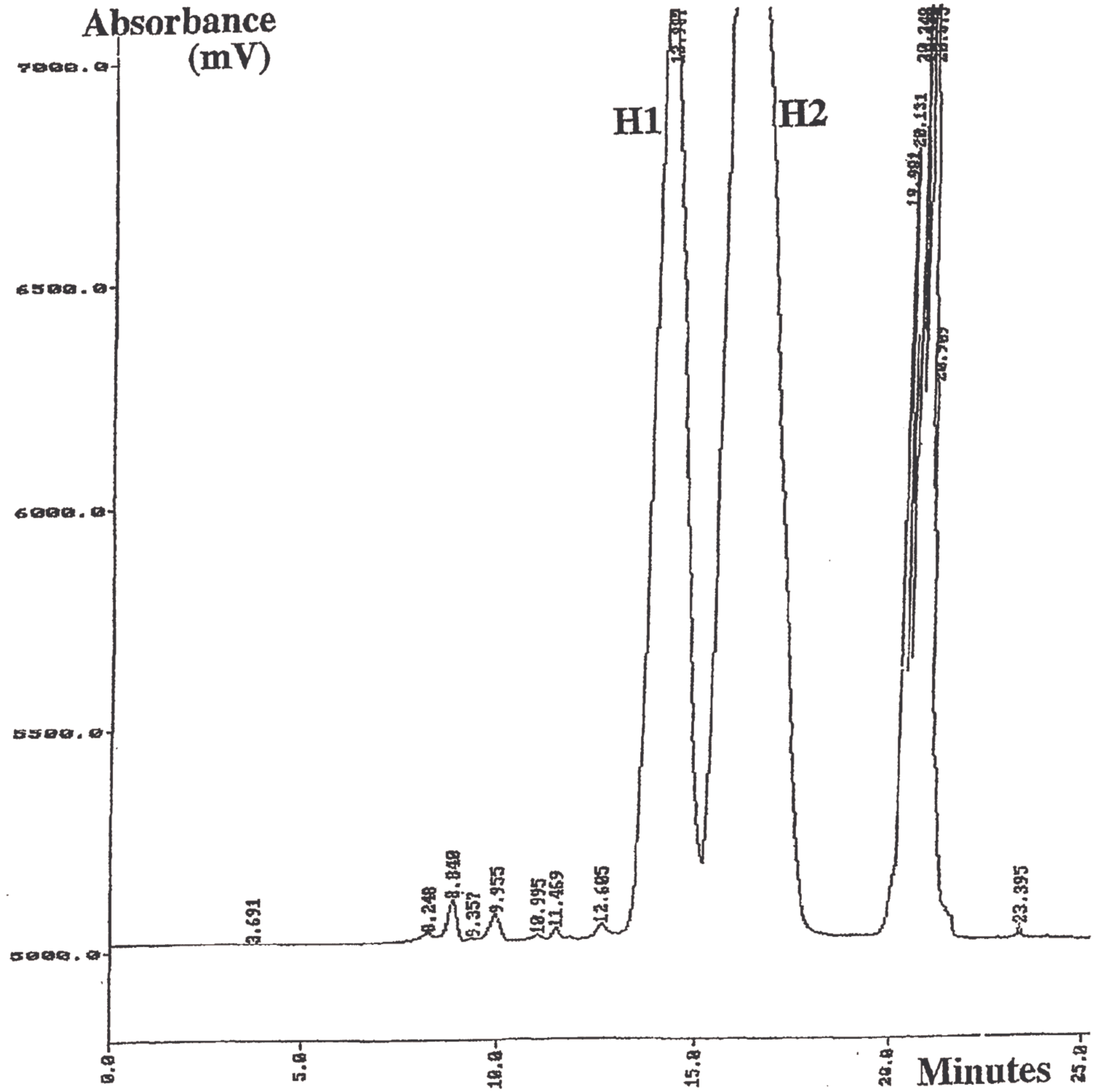

Fig S4a. HPLC profile shown to separate out PTI-777 compound h1 from PTI-777 compound h2.

Fig. S4b

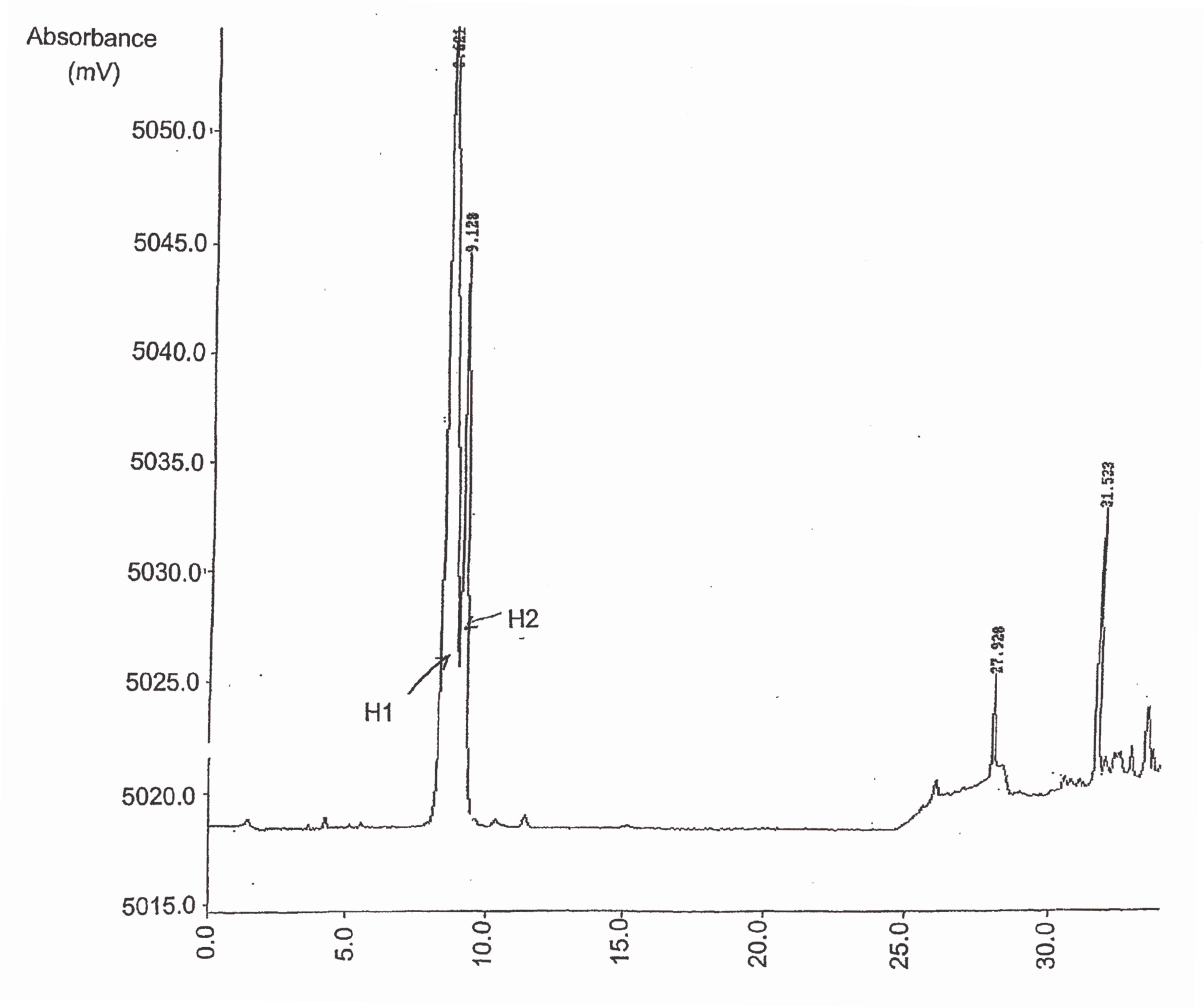

Fig. S4b. HPLC profile demonstrating isolation of PTI-777 compound h1 (16 mg) used for structure elucidation studies after fractionation of PTI-777 using silica gel chromatography, followed by HPLC.

Fig. S4c

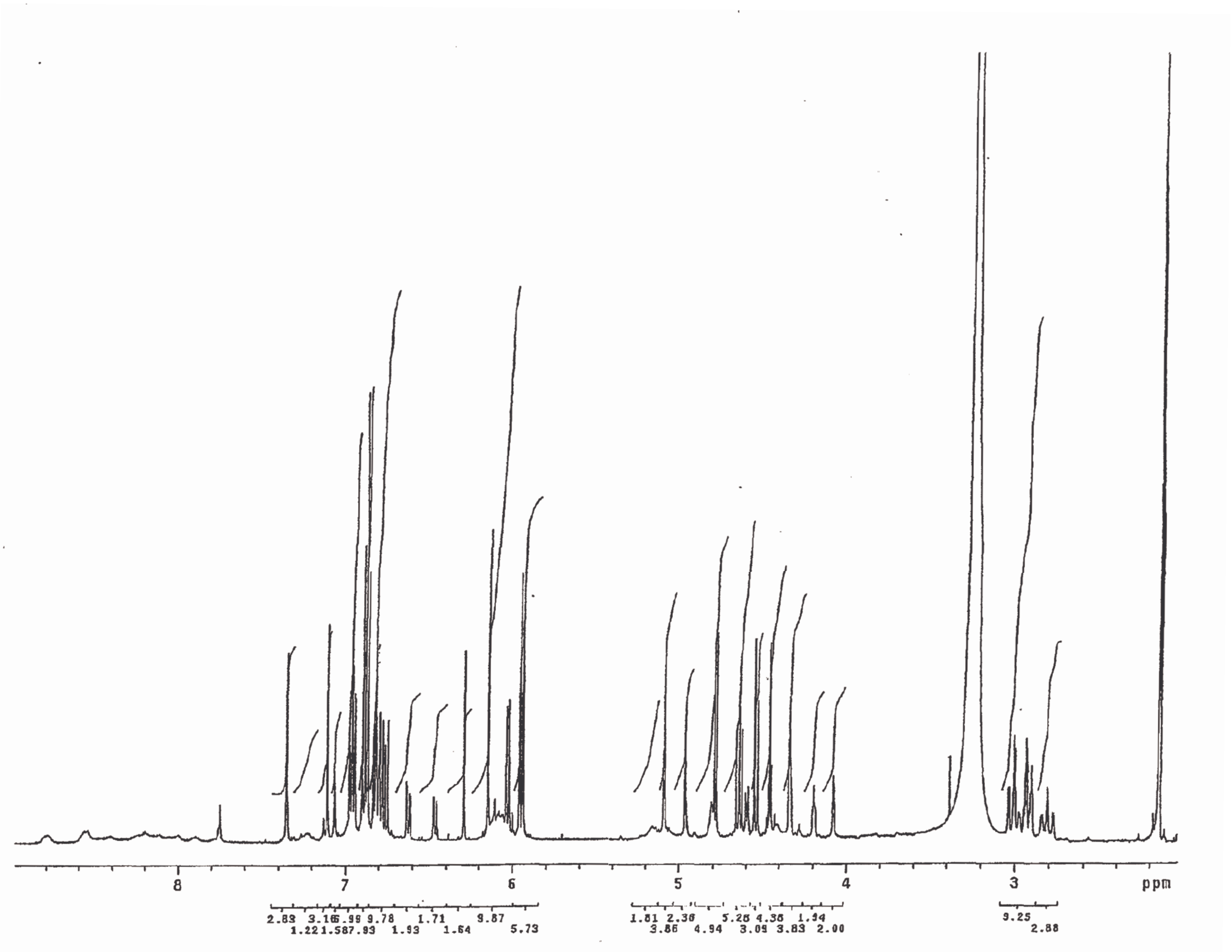

Fig. S4c. ^{1}H NMR spectra of PTI-777-compound h1 in deuteroacetone.

Fig. S4d

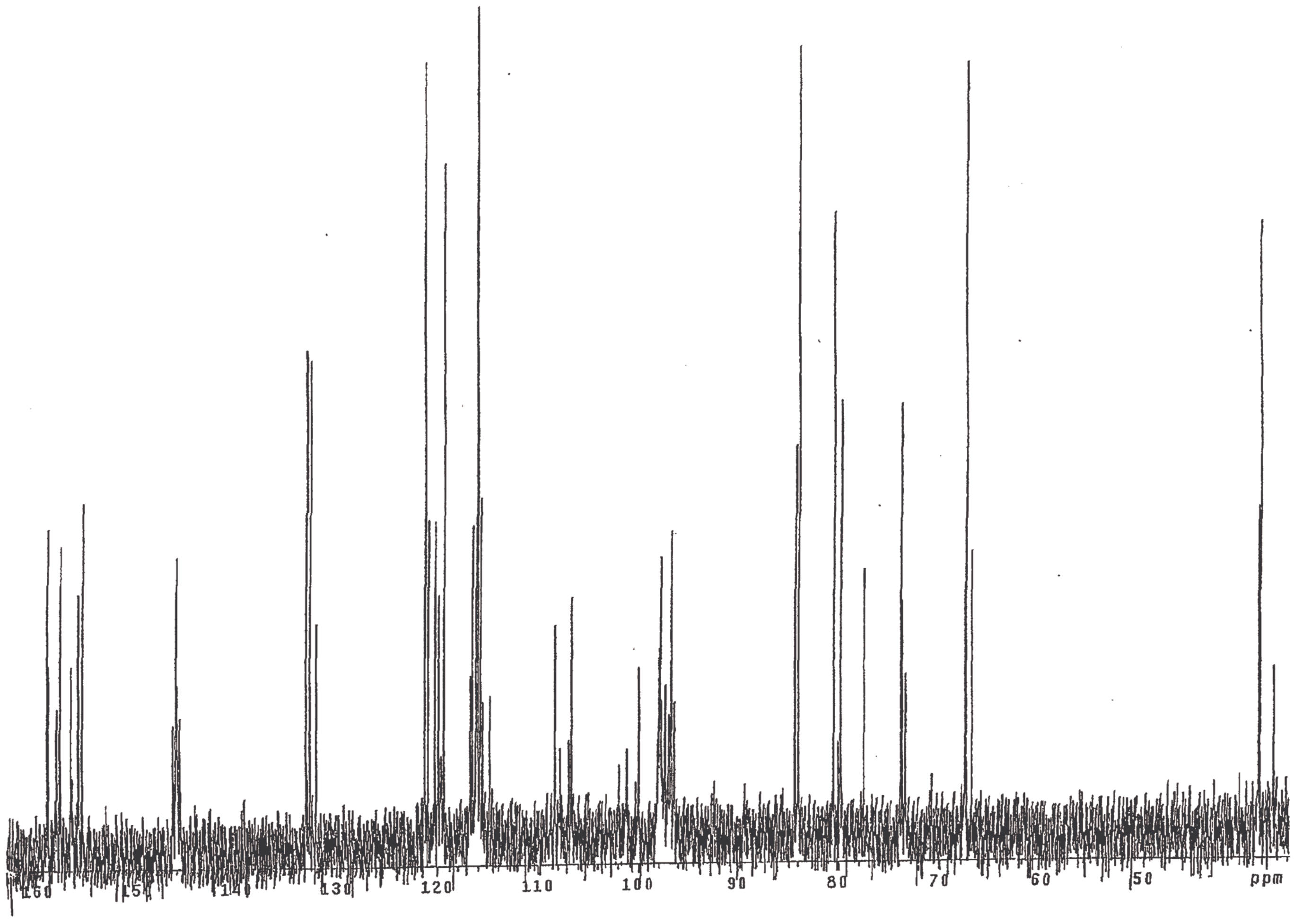

Fig. S4d. ^{13}C NMR spectra of PTI-777-compound h1 in deuteroacetone.

Fig. S4e

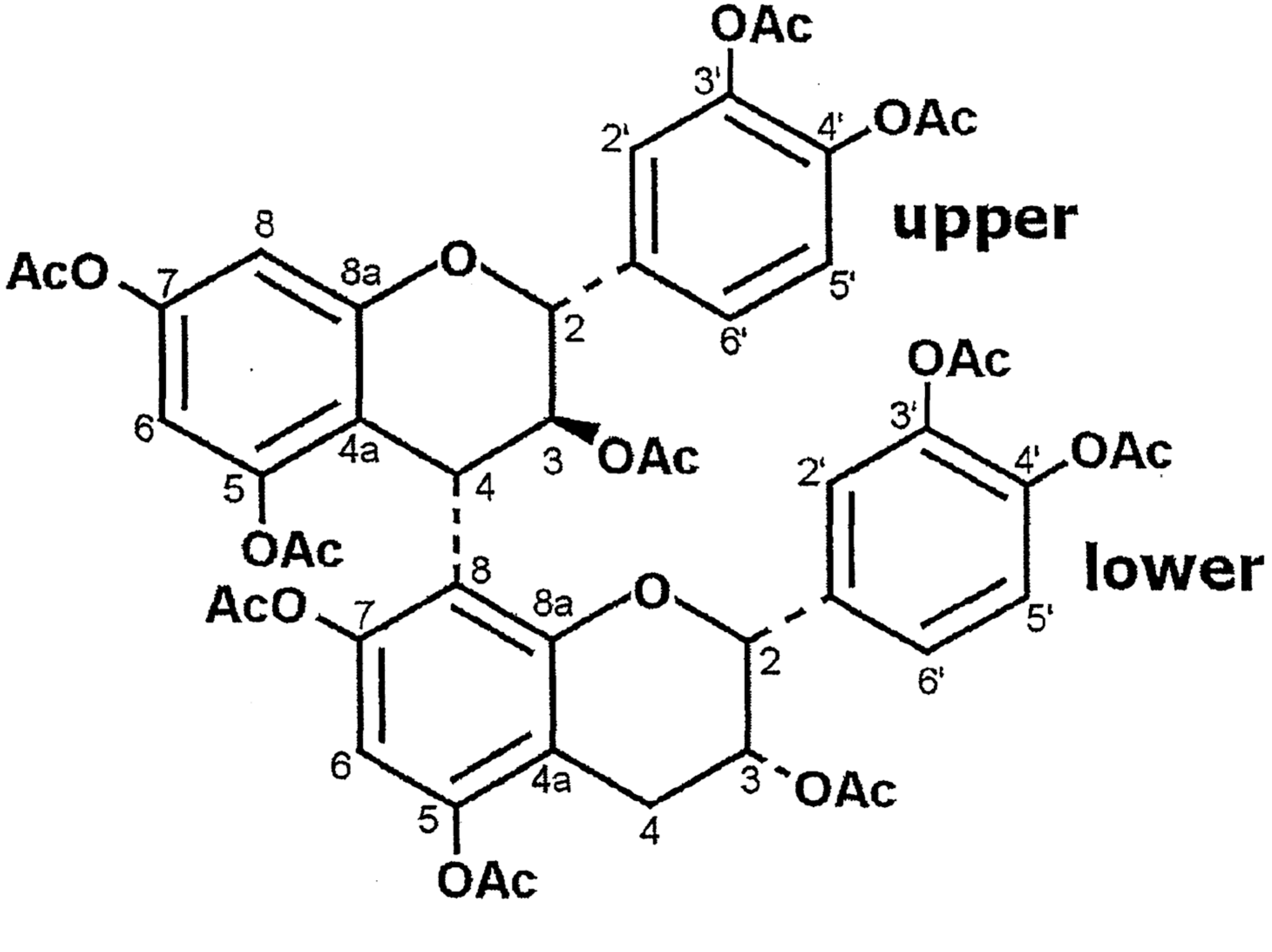

Fig. S4e. Postulated structure of acetylated PTI-777 compound h1.

Fig. S4f

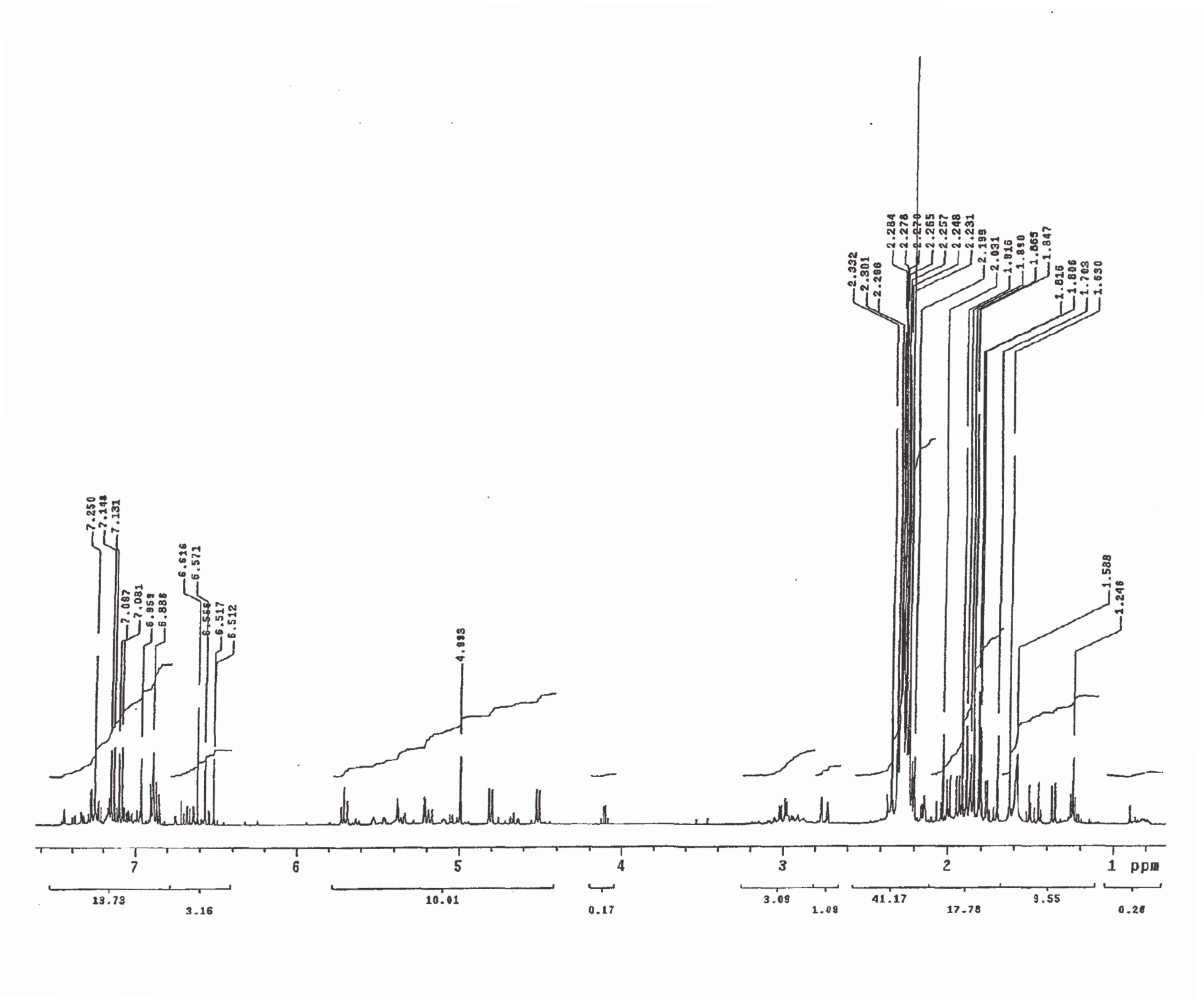

Fig. S4f. ^{1}H NMR spectrum of PTI-777 compound h1 peracetate in $CDCl_3$.

Fig. S4g

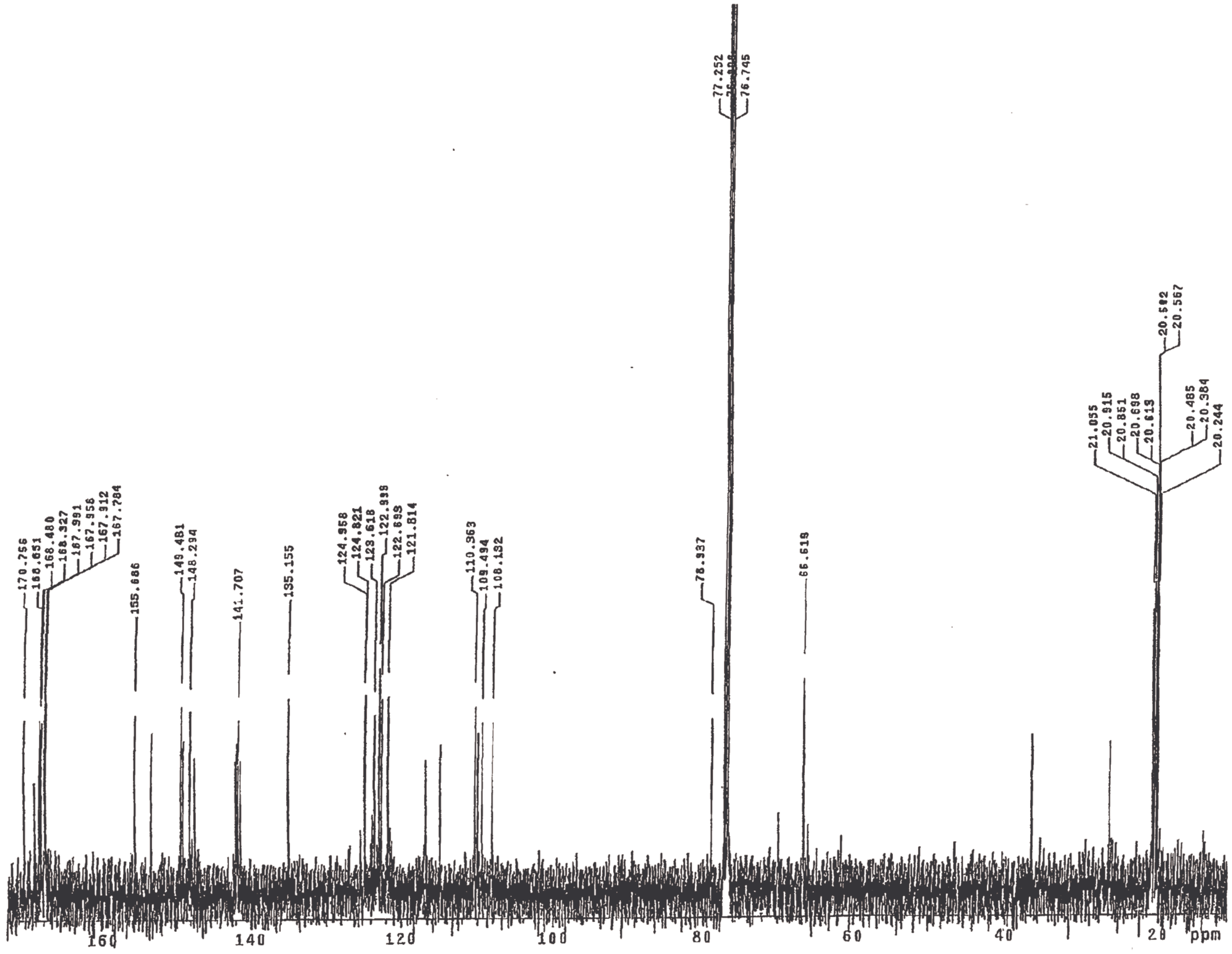

Fig. S4g. ^{13}C NMR spectrum of PTI-777 compound h1 peracetate in $CDCl_3$.

Fig. S4h

P88-17-3

Pulse Sequence: CIGAR
Solvent: CDCl3
Temp. 25.0 C / 298.1 K
User: 1-14-87
INOVA-500 "europa"

Relax. delay 1.000 sec
Acq. time 0.205 sec
Width 4997.5 Hz
2D Width 25133.5 Hz
64 repetitions
256 increments
OBSERVE H1, 499.7381559 MHz
DATA PROCESSING
Gauss apodization 0.102 sec
Sine bell 0.102 sec
F1 DATA PROCESSING
Gauss apodization 0.016 sec
Sine bell 0.087 sec
FT size 2048 x 4096
Total time 6 hr, 9 min, 51 sec

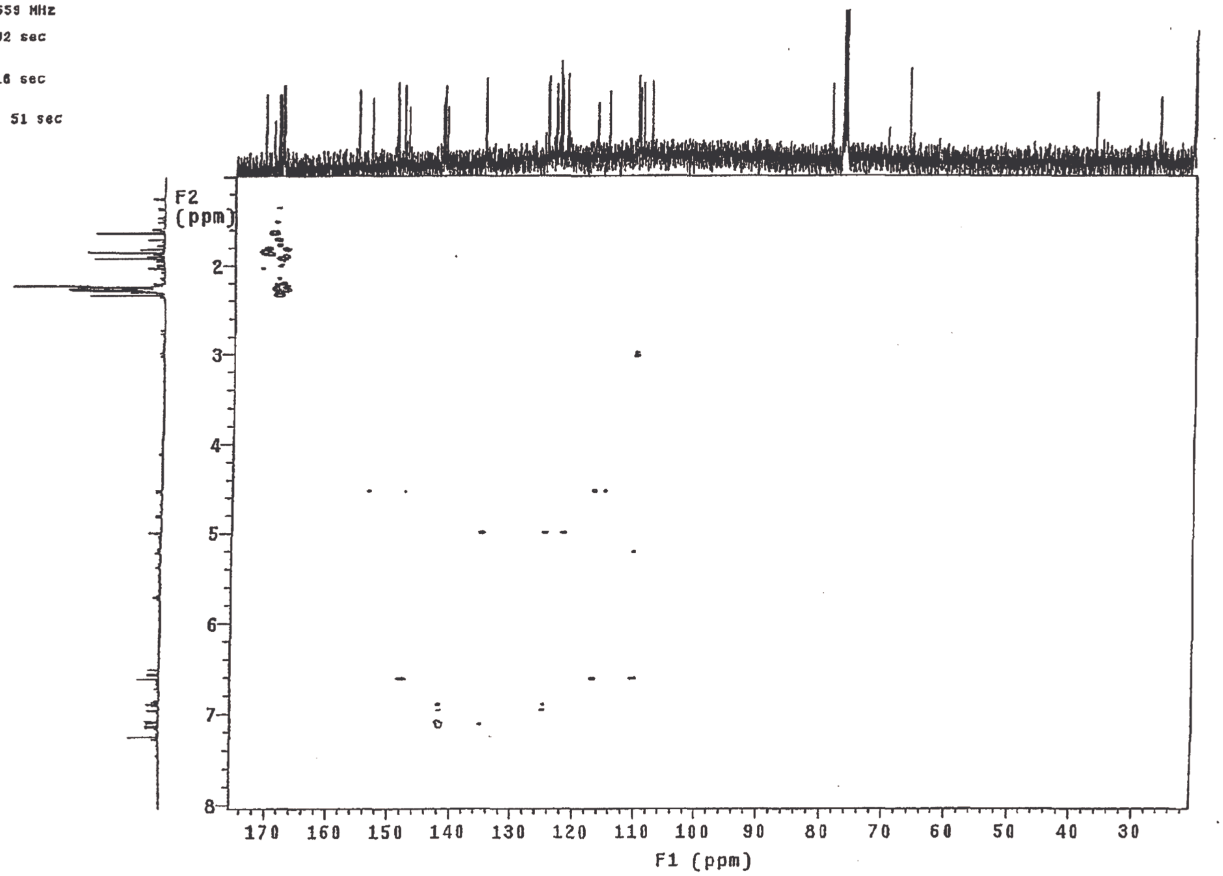

Fig. S4h. CIGAR ^{1}H-^{13}C correlation of PTI-777-compound h1 peracetate (low resolution).

Fig. S4i

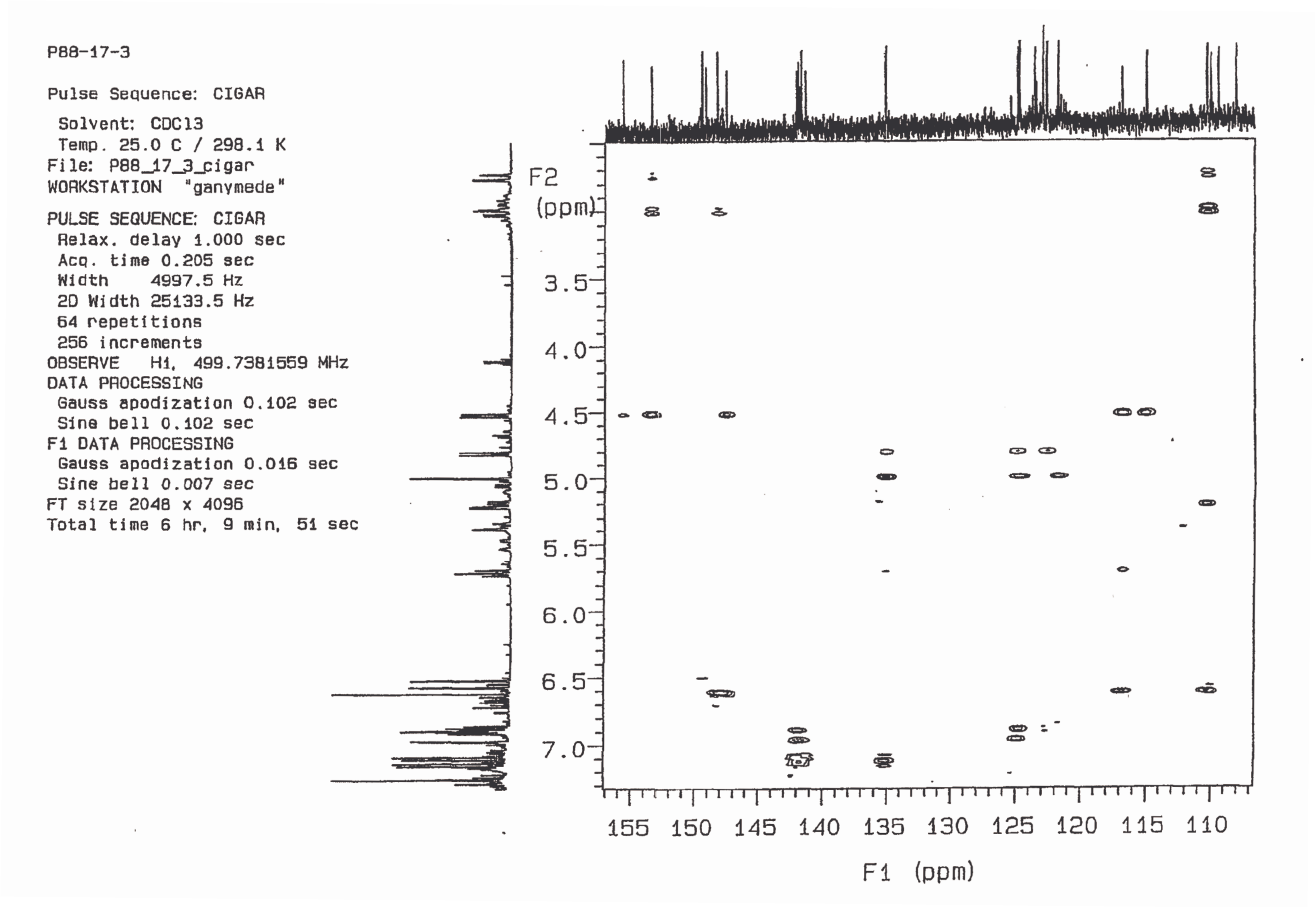

Fig. S4i. CIGAR ^{1}H-^{13}C correlation of PTI-777 compound h1 peracetate.

Fig. S4j

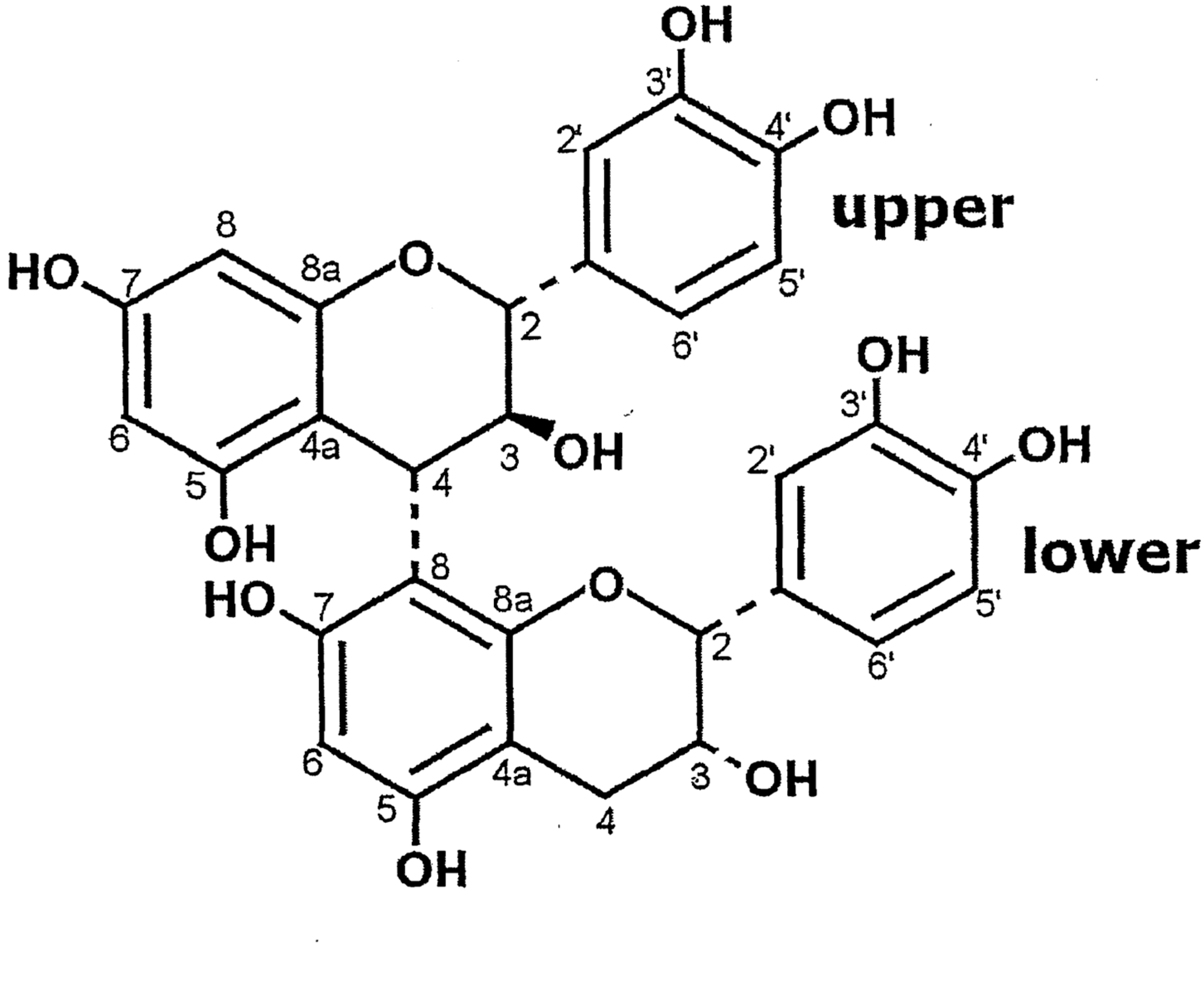

Fig. S4j. Structure of PTI-777 h1 identified as catechin-4α-8-epicatechin (proanthocyanidin B4).

Fig. S5a

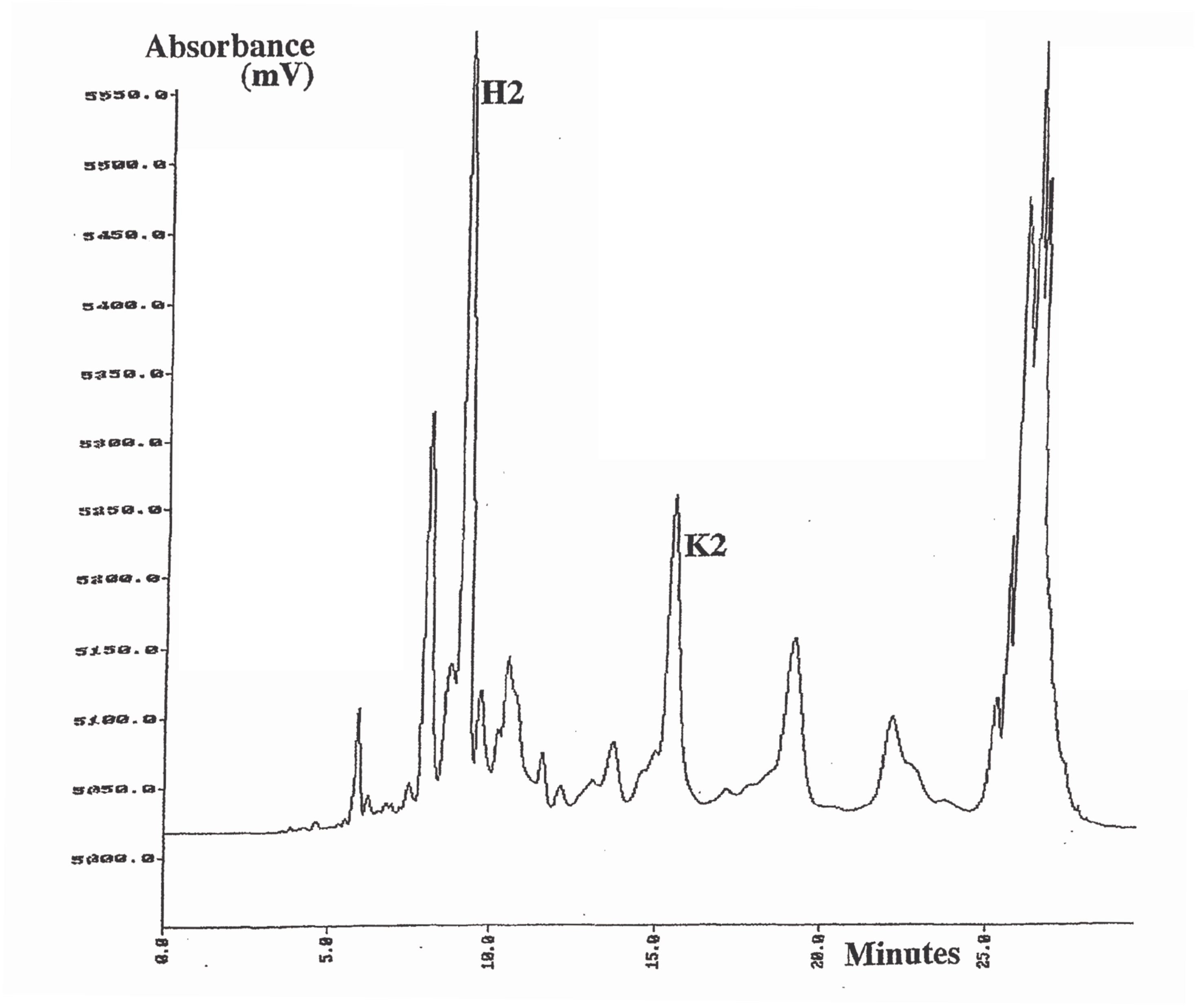

Fig. S5a. Preparative HPLC demonstrates isolation and purification of compound PTI-777 k2.

Fig. S5b

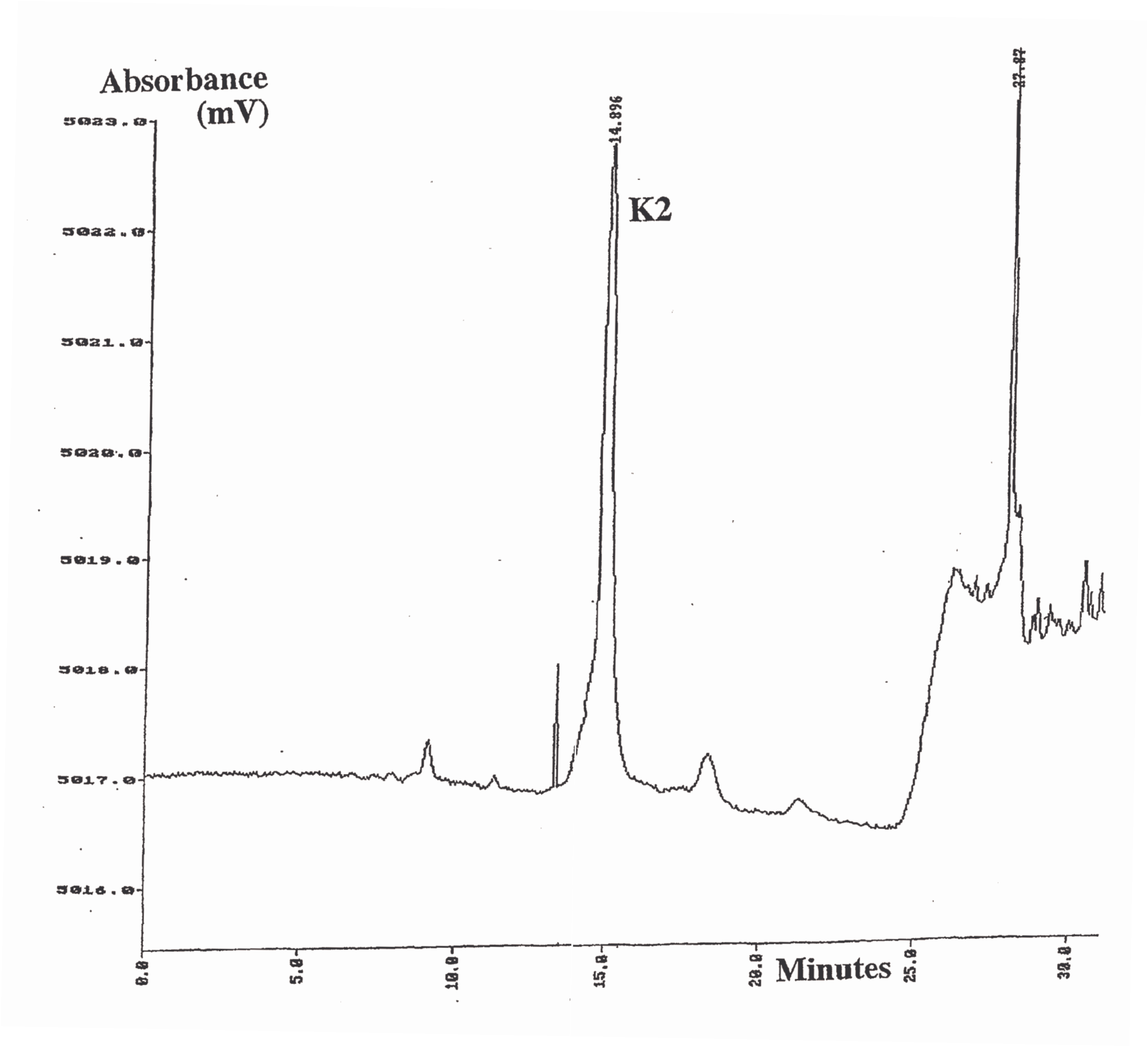

Fig. S5b. HPLC purification of PTI-777 compound k2.

Fig. S5c

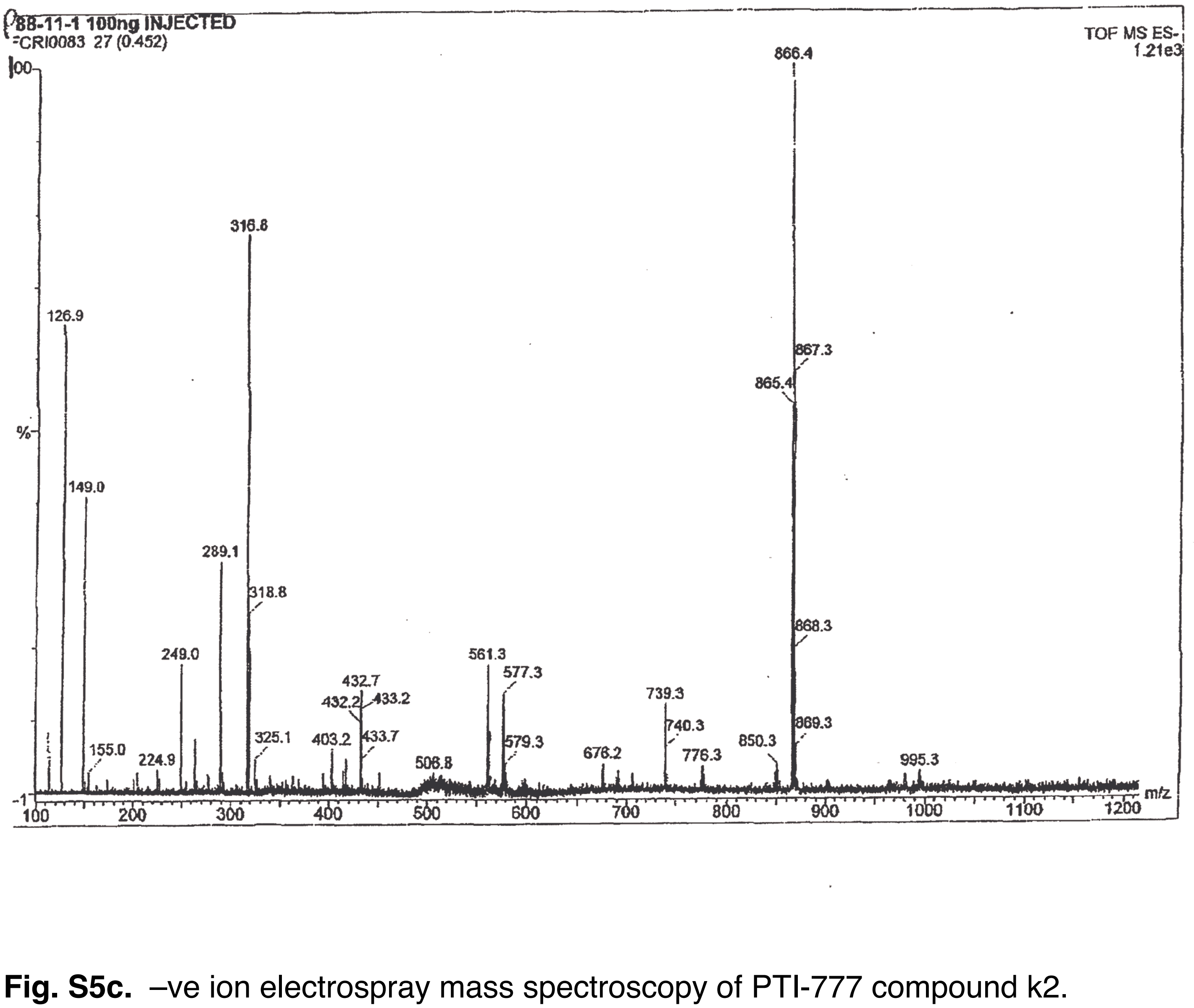

Fig. S5c. –ve ion electrospray mass spectroscopy of PTI-777 compound k2.

Fig. S5d

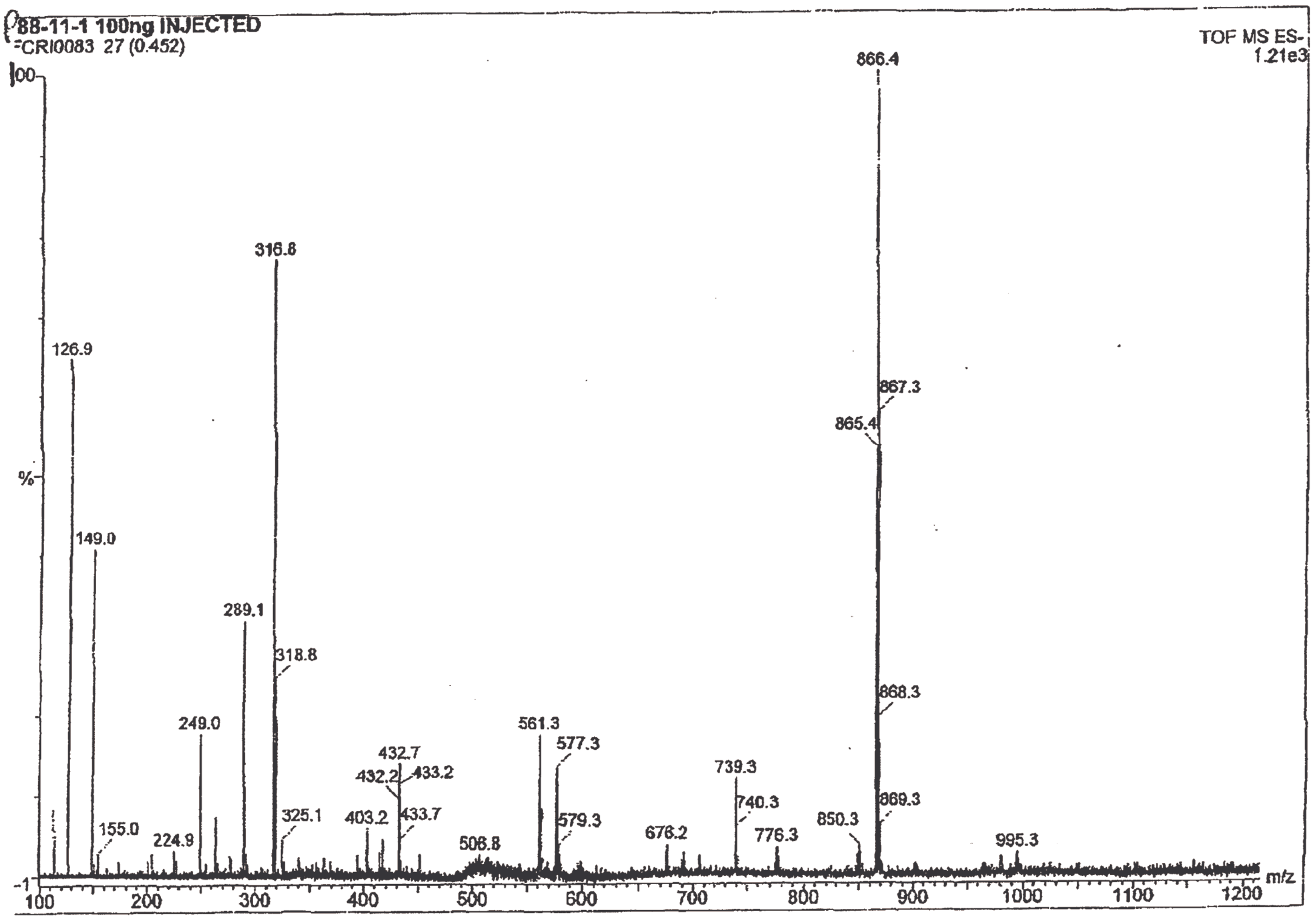

Fig. S5d. ^{1}H NMR spectra of PTI-777 compound k2.

Fig. S5e

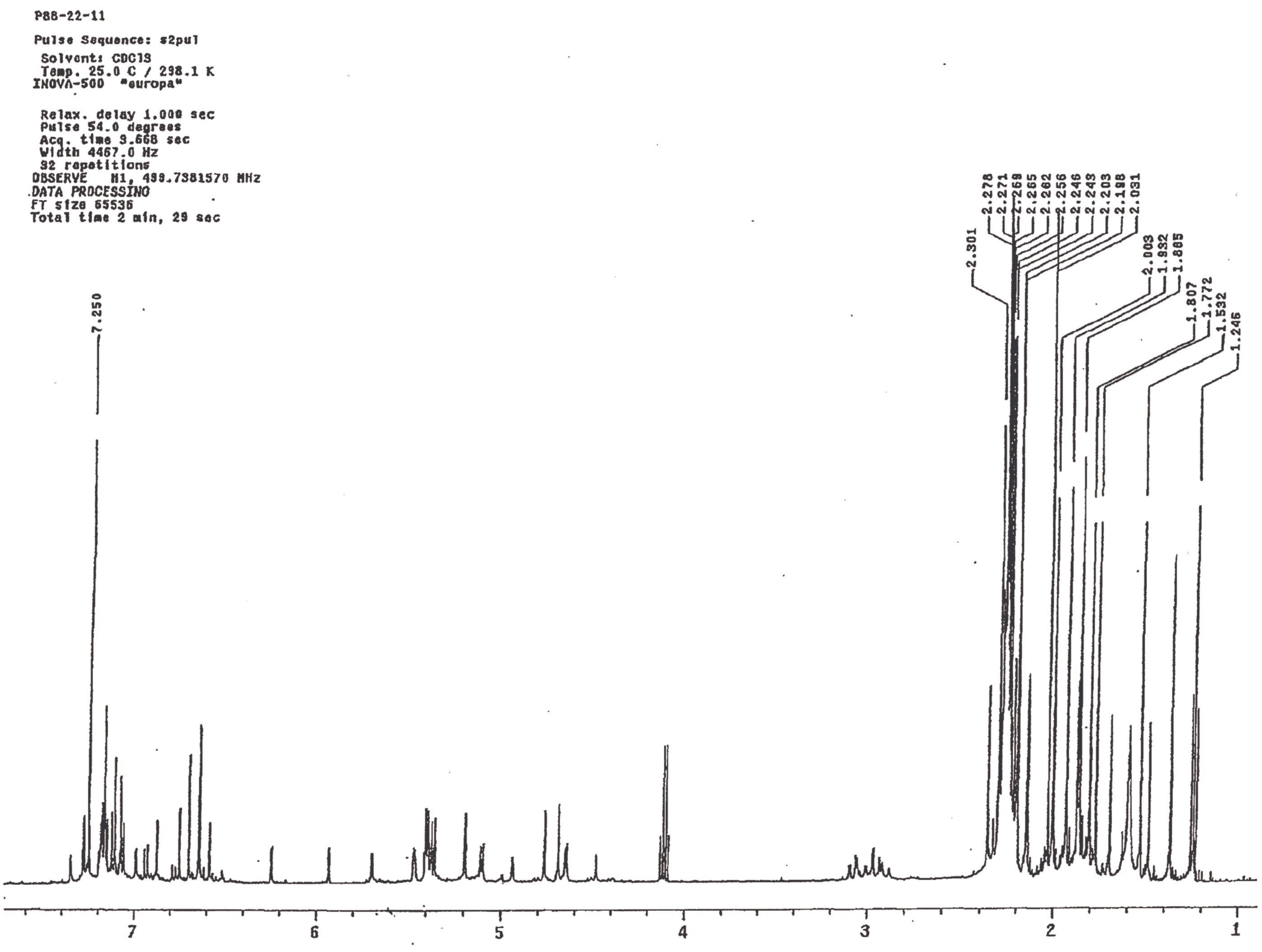

Fig. S5e. ^{1}H NMR spectra of PTI-777 compound k2 peracetate.

Fig. S5f

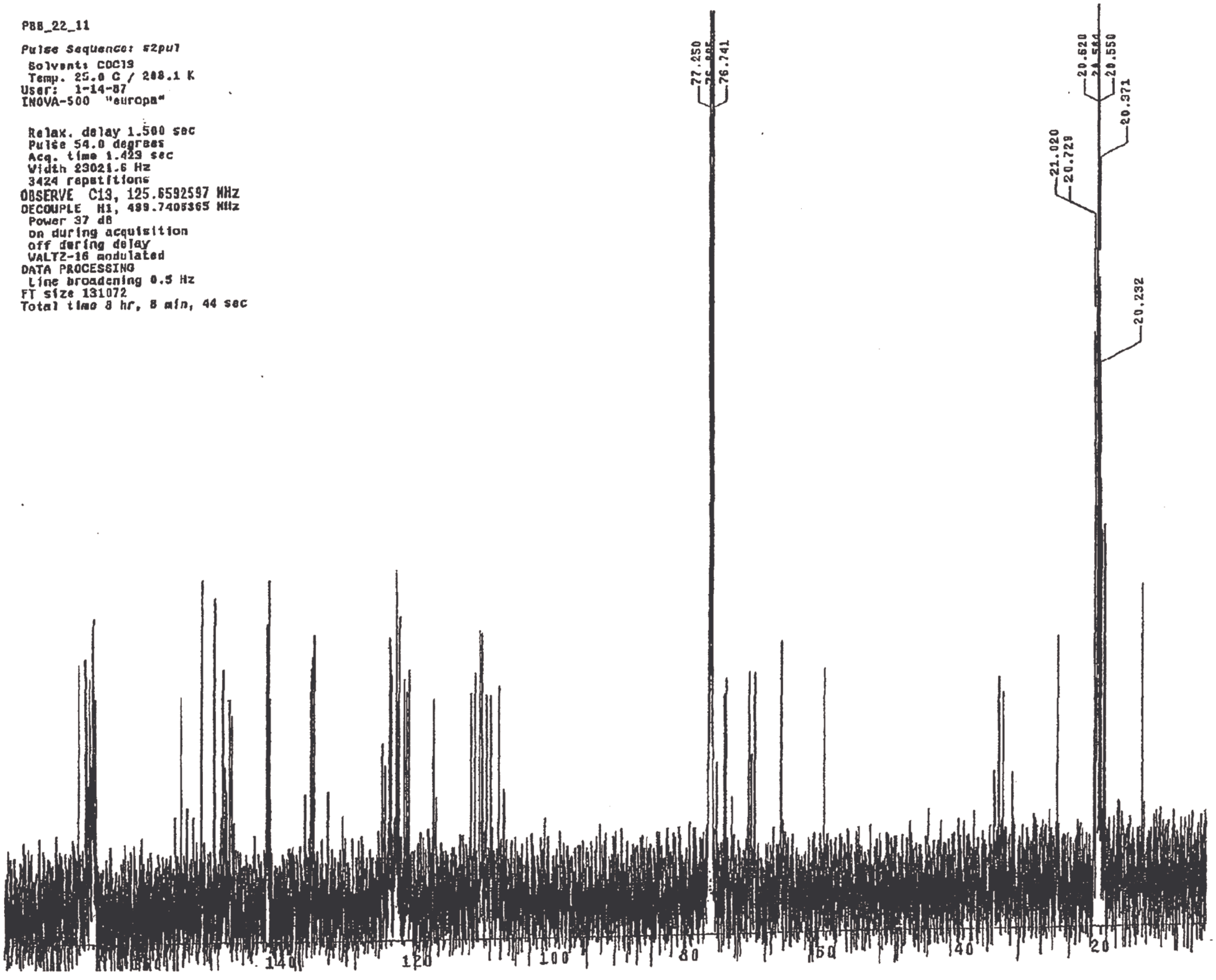

Fig. S5f. ^{13}C NMR spectra of PTI-777 compound k2 peracetate.

Fig. S5g

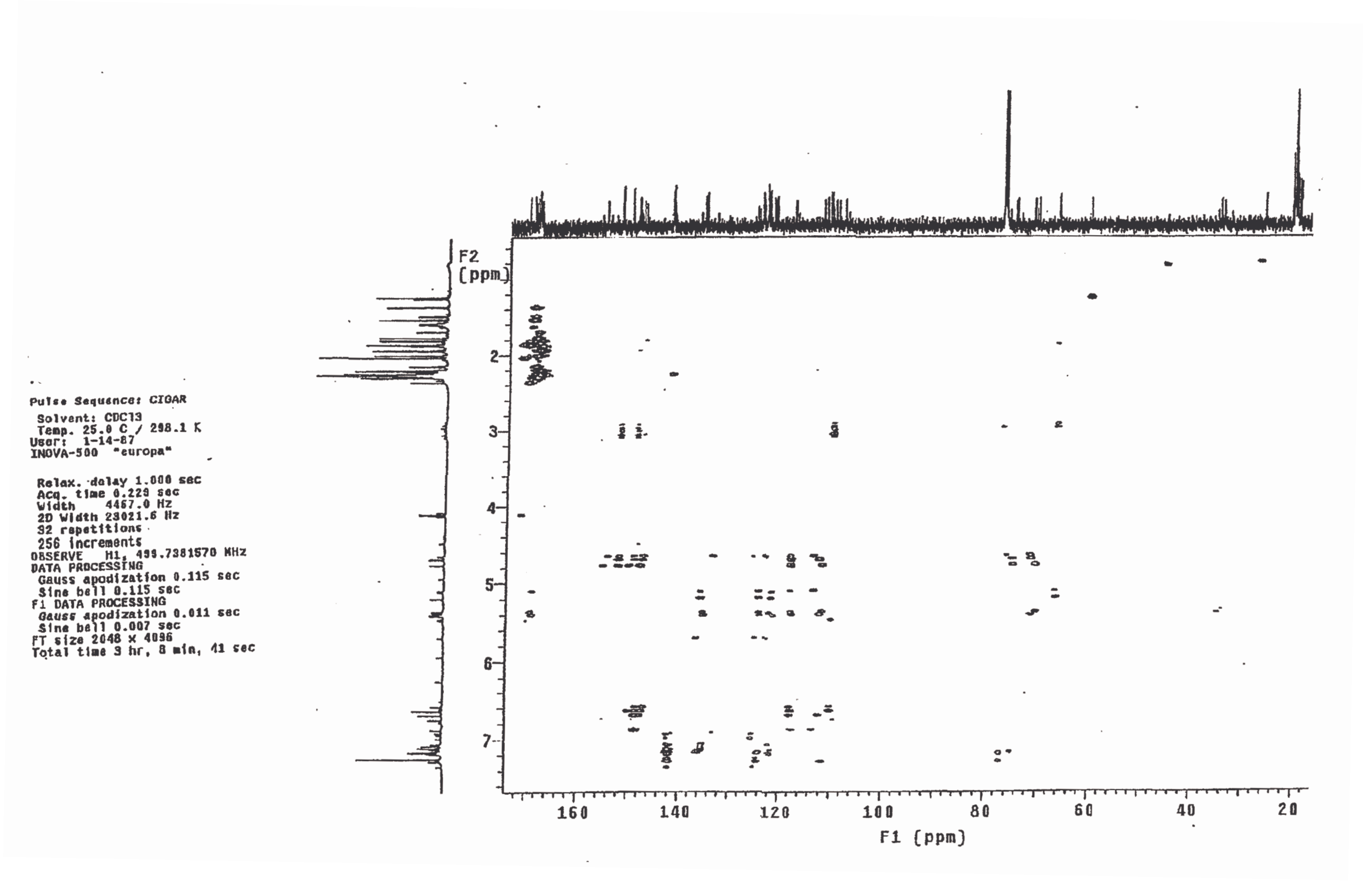

Fig. S5g. CIGAR ^1H-^{13}C correlation spectrum of PTI-777 compound k2 peracetate (low resolution).

Fig. S5h

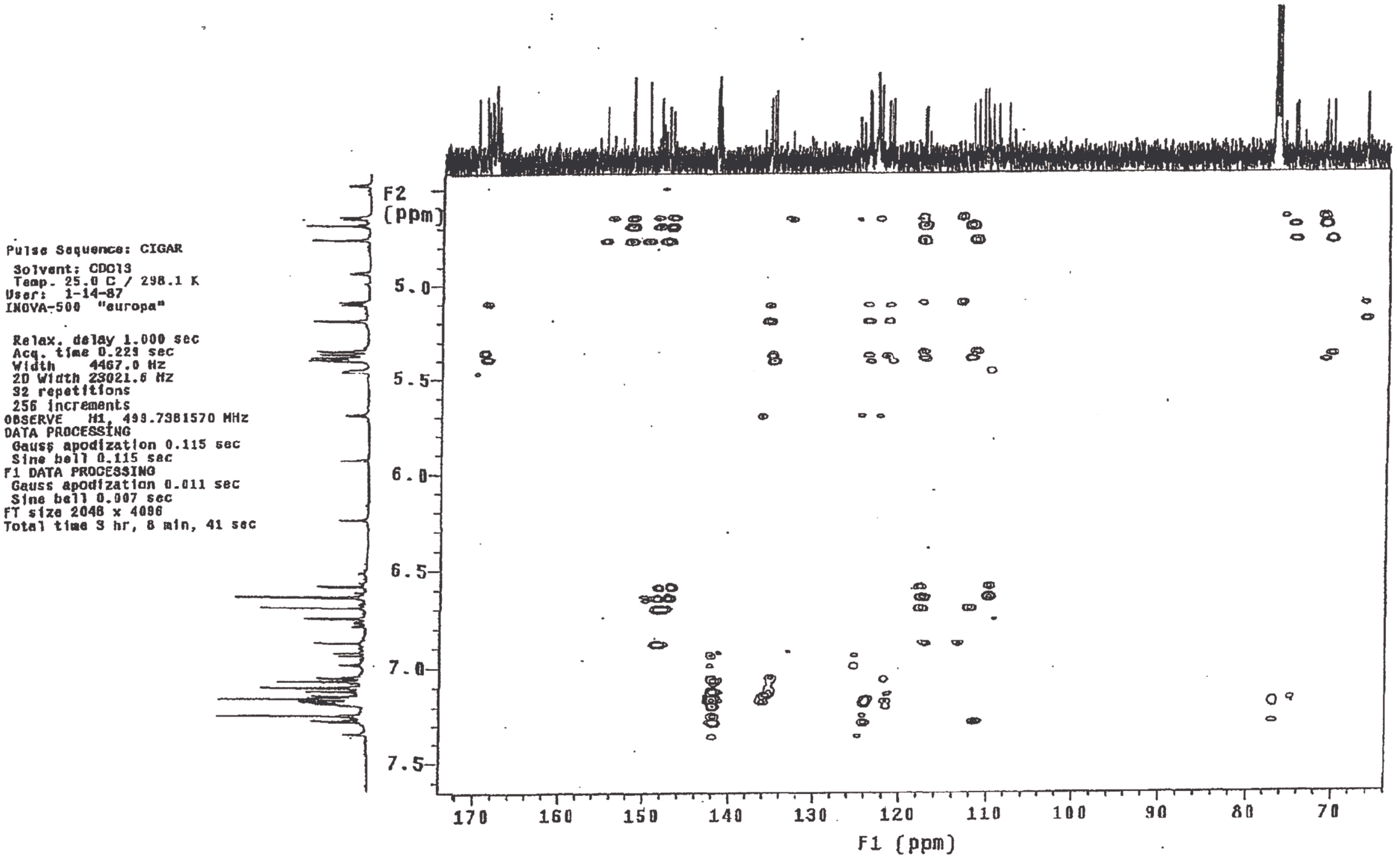

Fig. S5h. High resolution of CIGAR ^{1}H-^{13}C correlation spectrum of PTI-777 compound k2 peracetate.

Fig. S5i

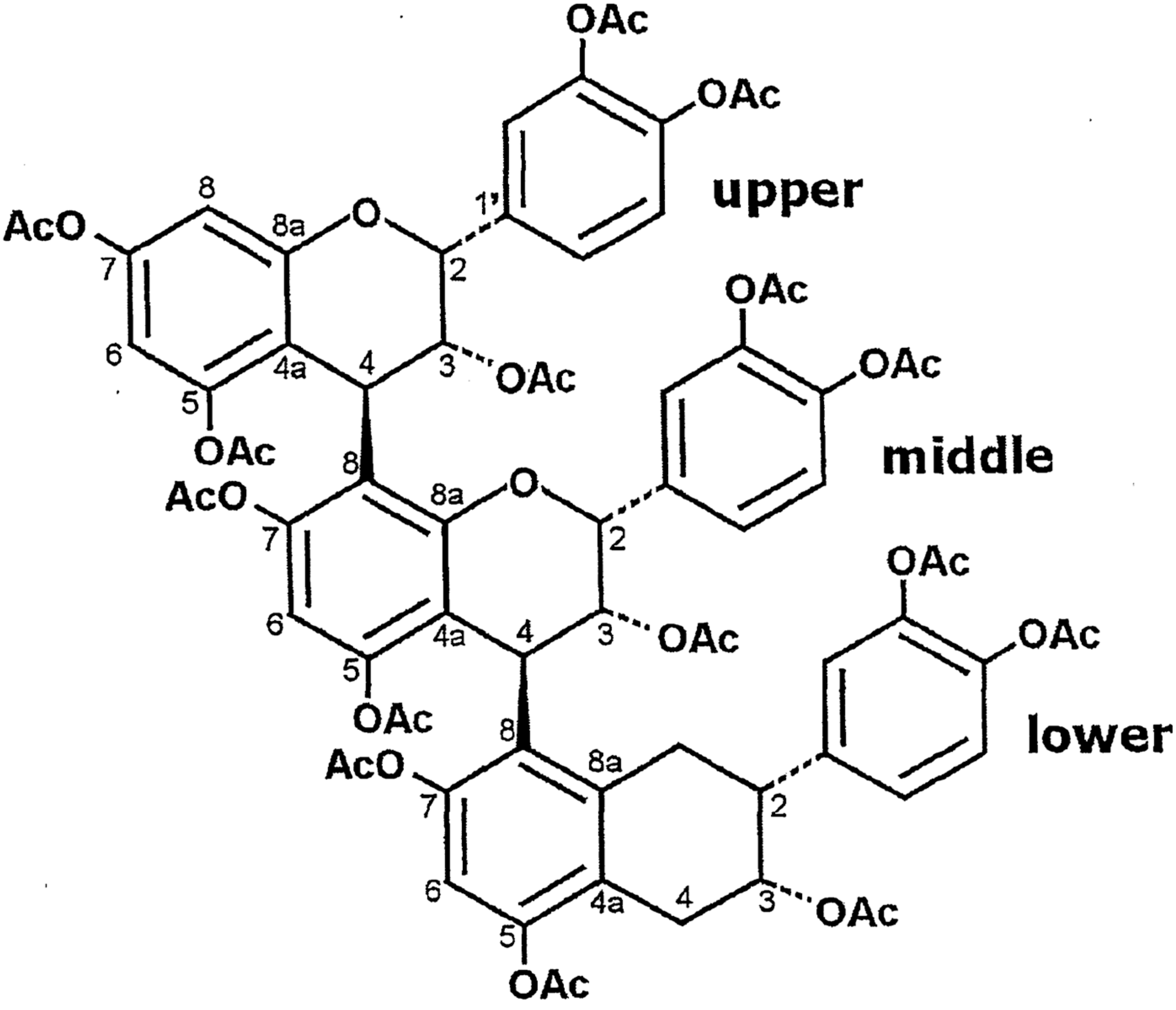

Fig. S5i. Peracetate structure of PTI-777 compound k2.

Fig. S5j

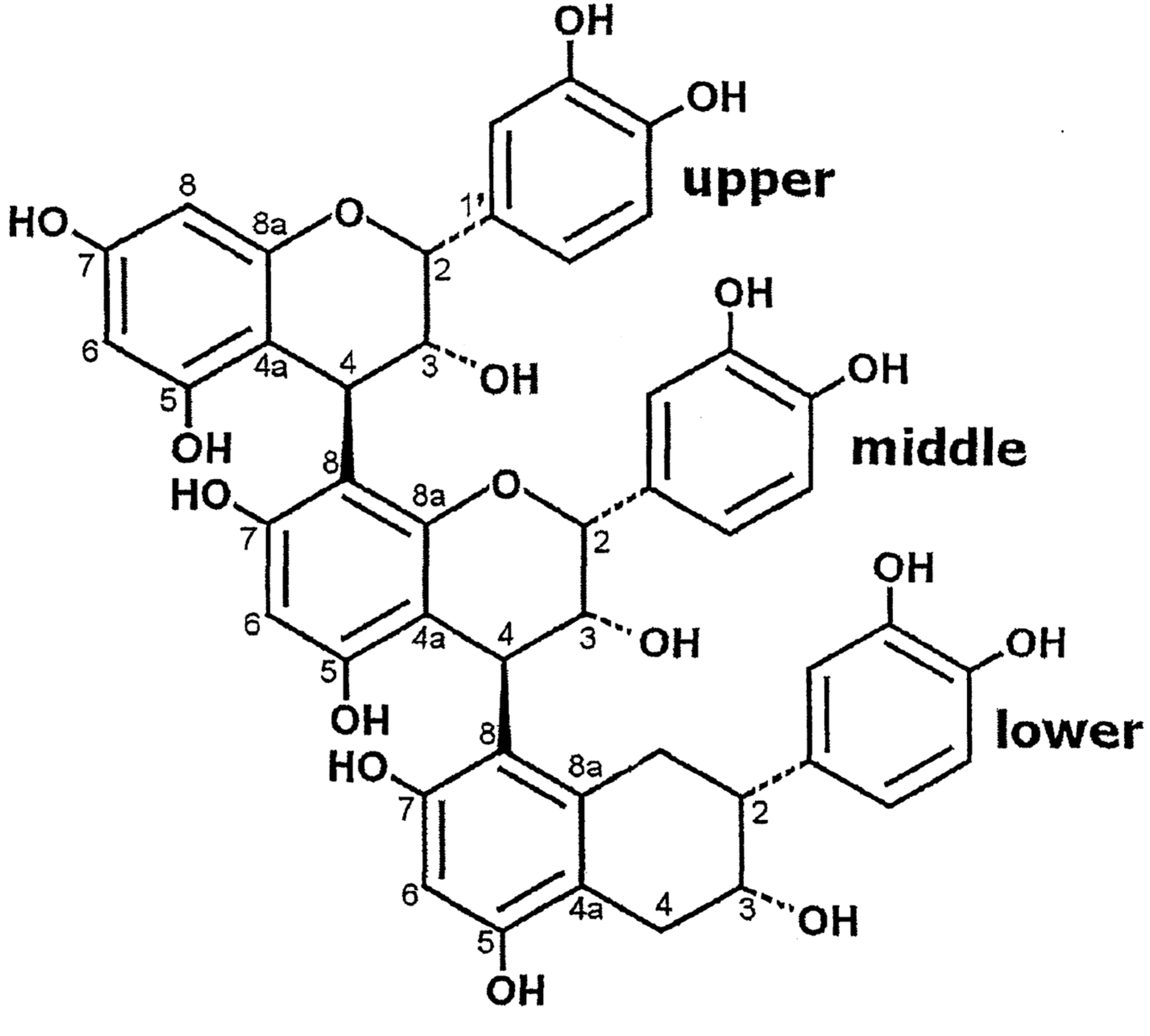

Fig. S5j. Structure of PTI-777 compound k2 determined to be epicatechin-4β-8-epicatechin-4β-8-epicatechin (proanthocyanidin B4).

Fig. S6a

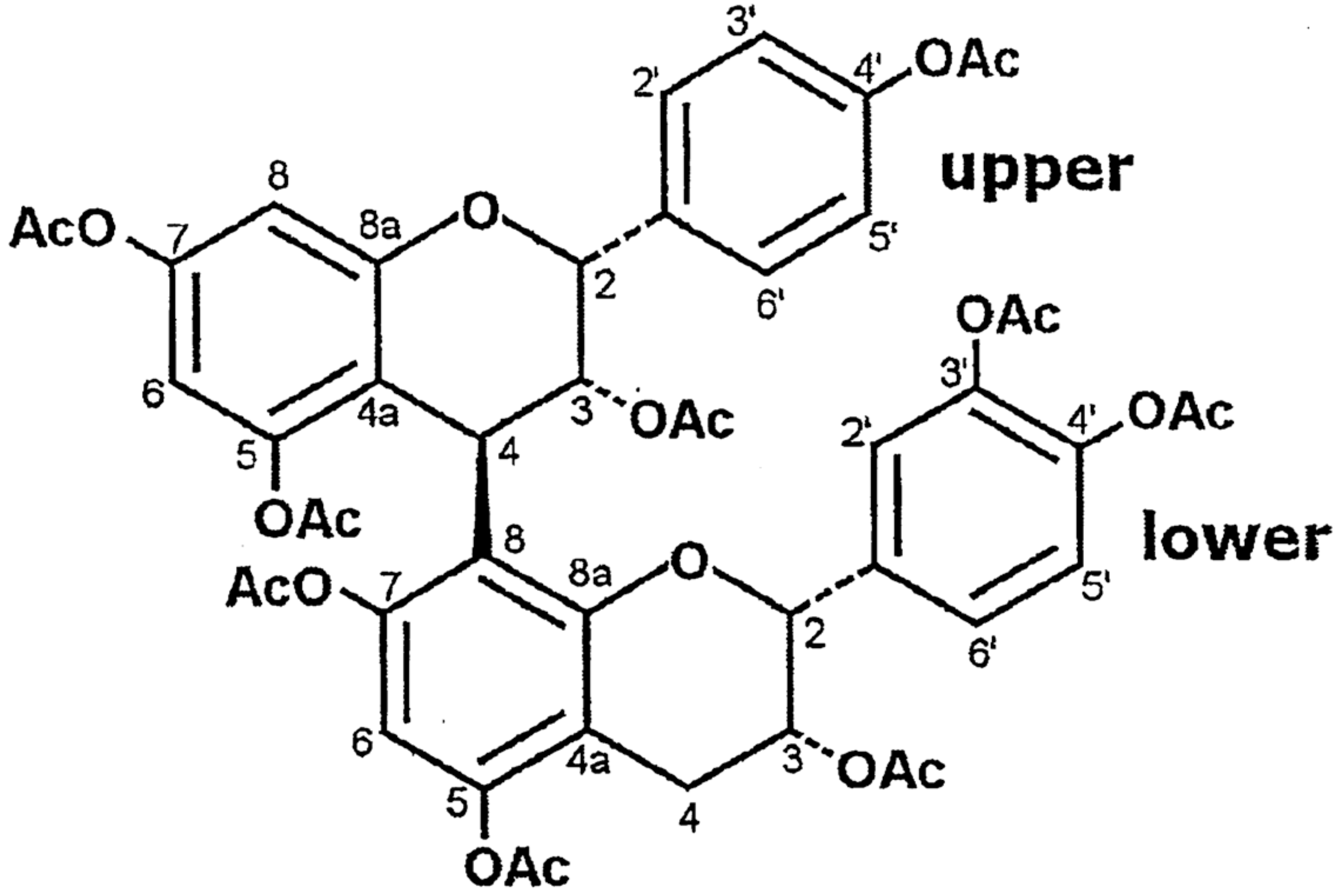

Fig. S6a. Peracetate structure of PTI-777 compound k1.

Fig. S6b

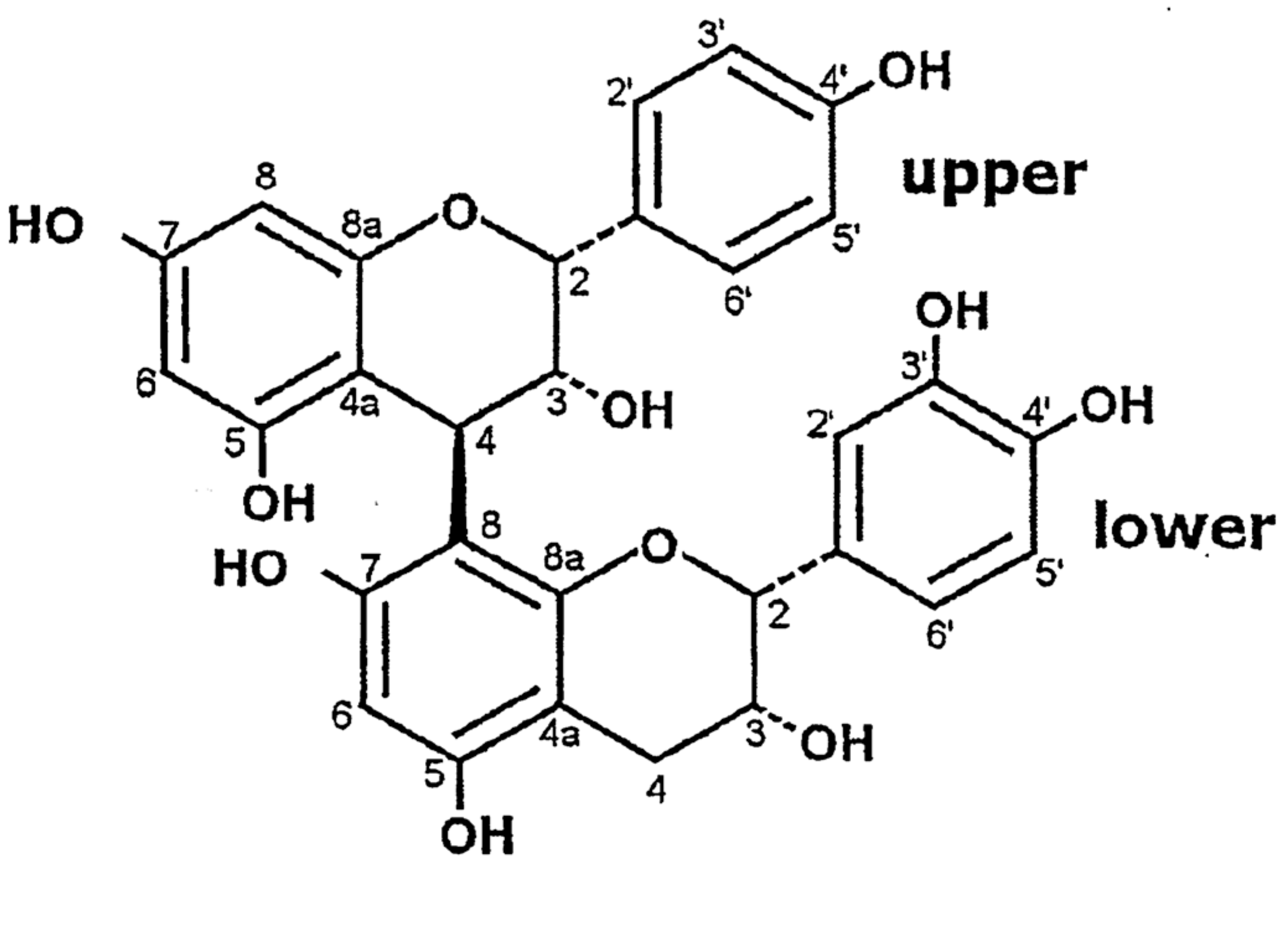

Fig. S6b. Structure of PTI-777 compound k1 determined to be epiafzelechin-4β-8-epicatechin.

Fig. S6c

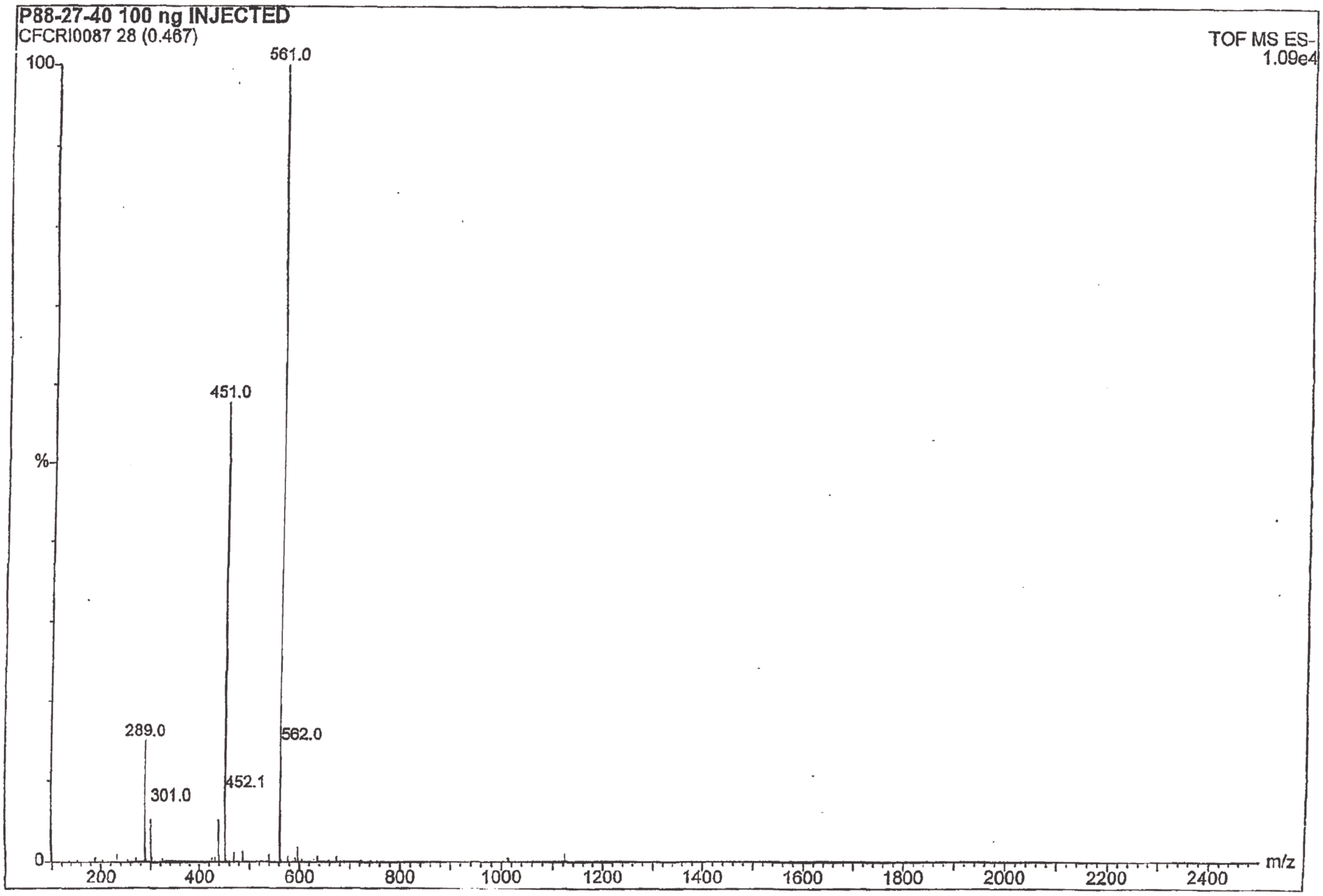

Fig. S6c. -ve ion electrospray mass spectrum of PTI-777 compound k1.

Fig. S6d

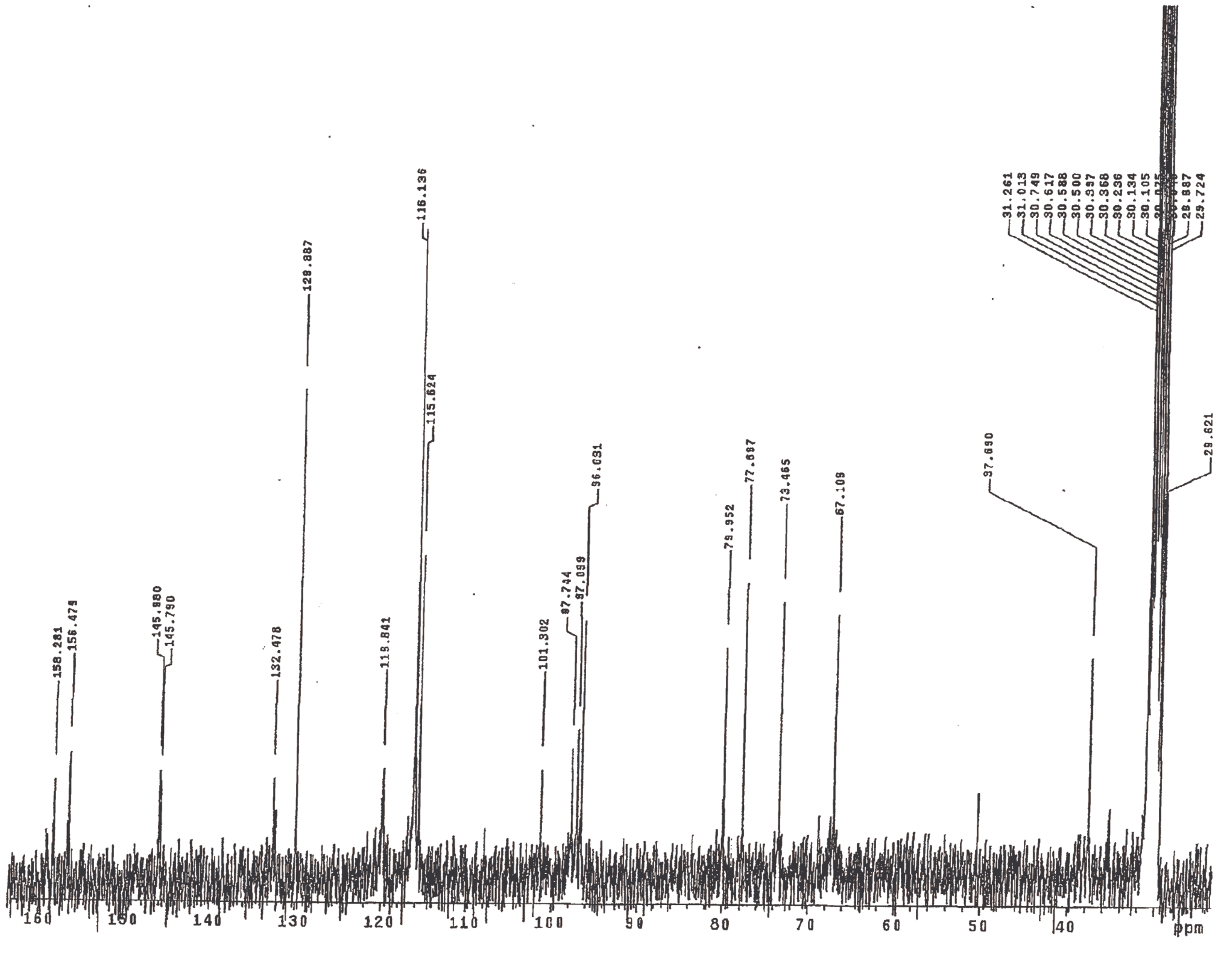

Fig. S6d. ^{13}C NMR spectra of PTI-777 compound k1 in deuteroacetone.

Fig. S6e

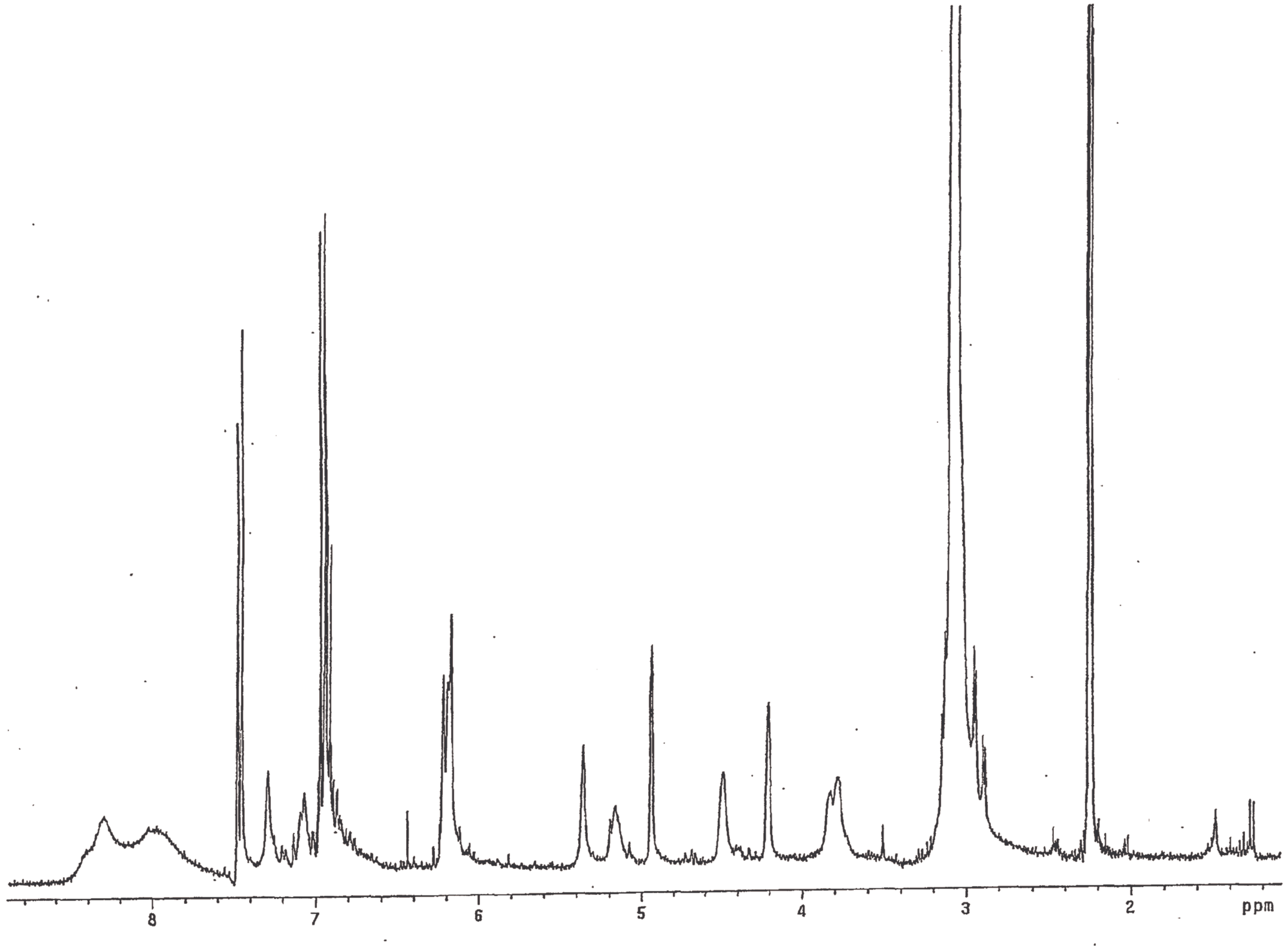

Fig. S6e. ^{1}H NMR spectra of PTI-777 compound k1 in deuteroacetone.

Fig. S6f

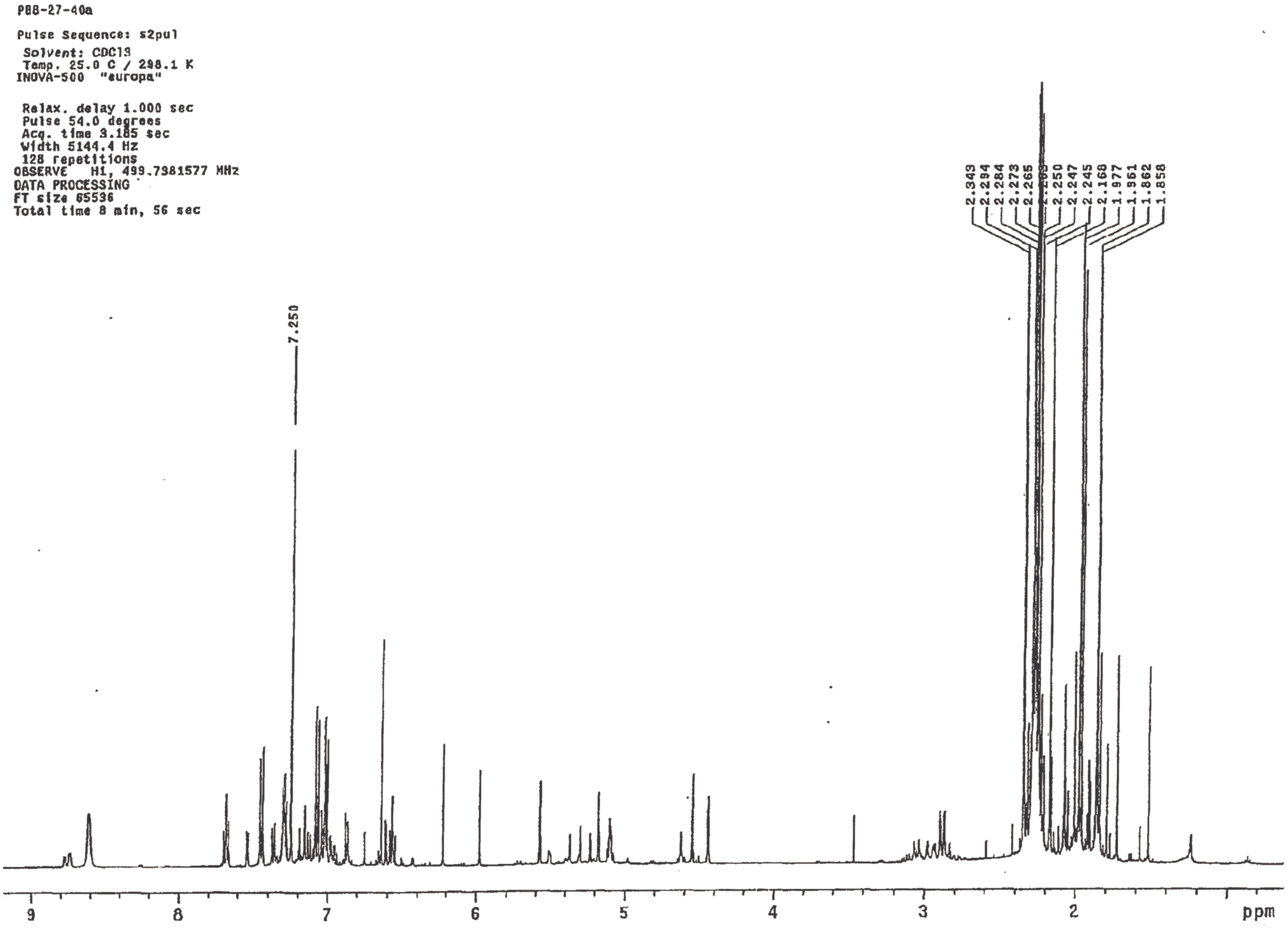

Fig. S6f. ^{1}H NMR spectra of PTI-777 compound k1 peracetate in $CDCl_3$.

Fig. S6g

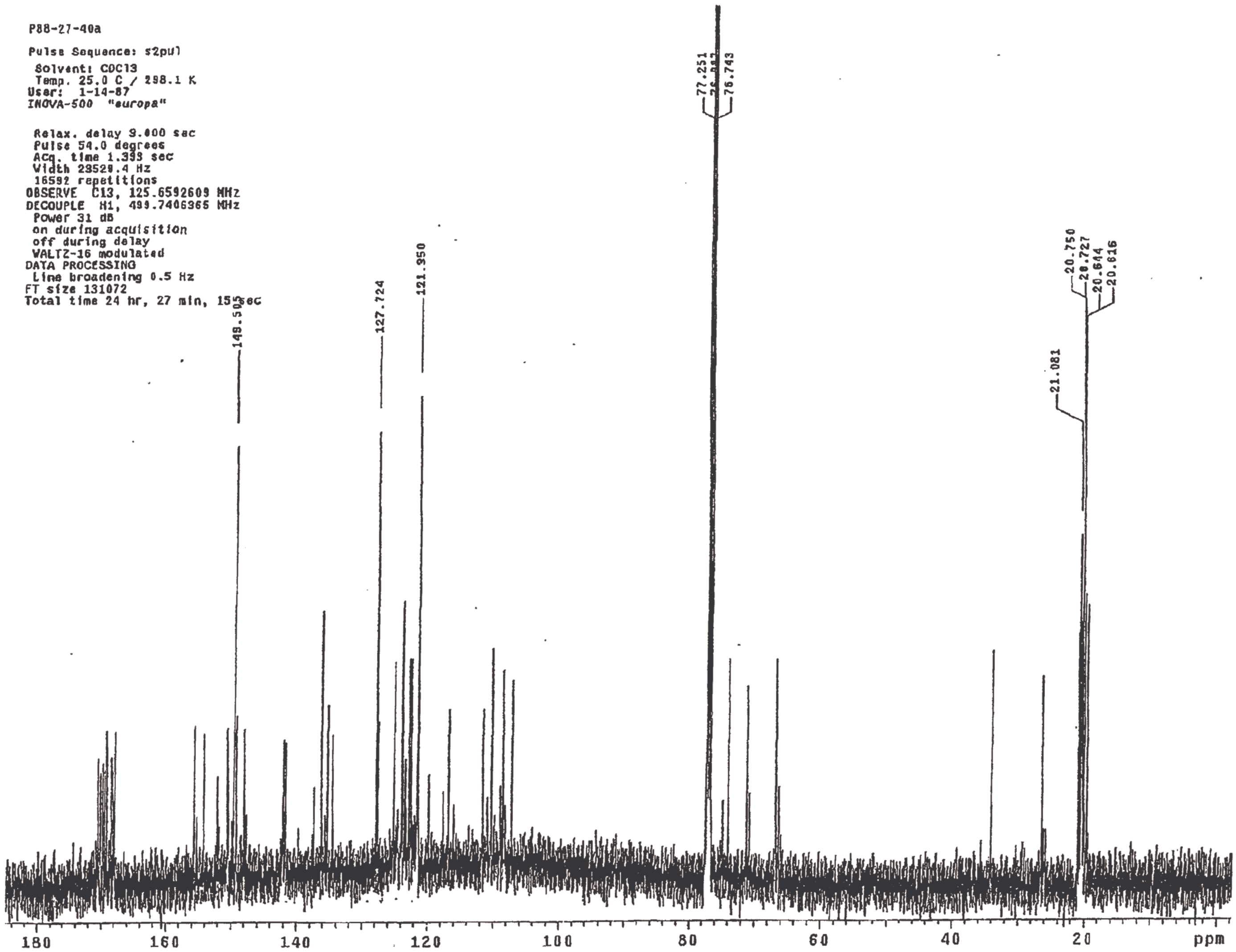

Fig. S6g. ^{13}C NMR spectra of PTI-777 compound k1 peracetate.

Fig. S6h

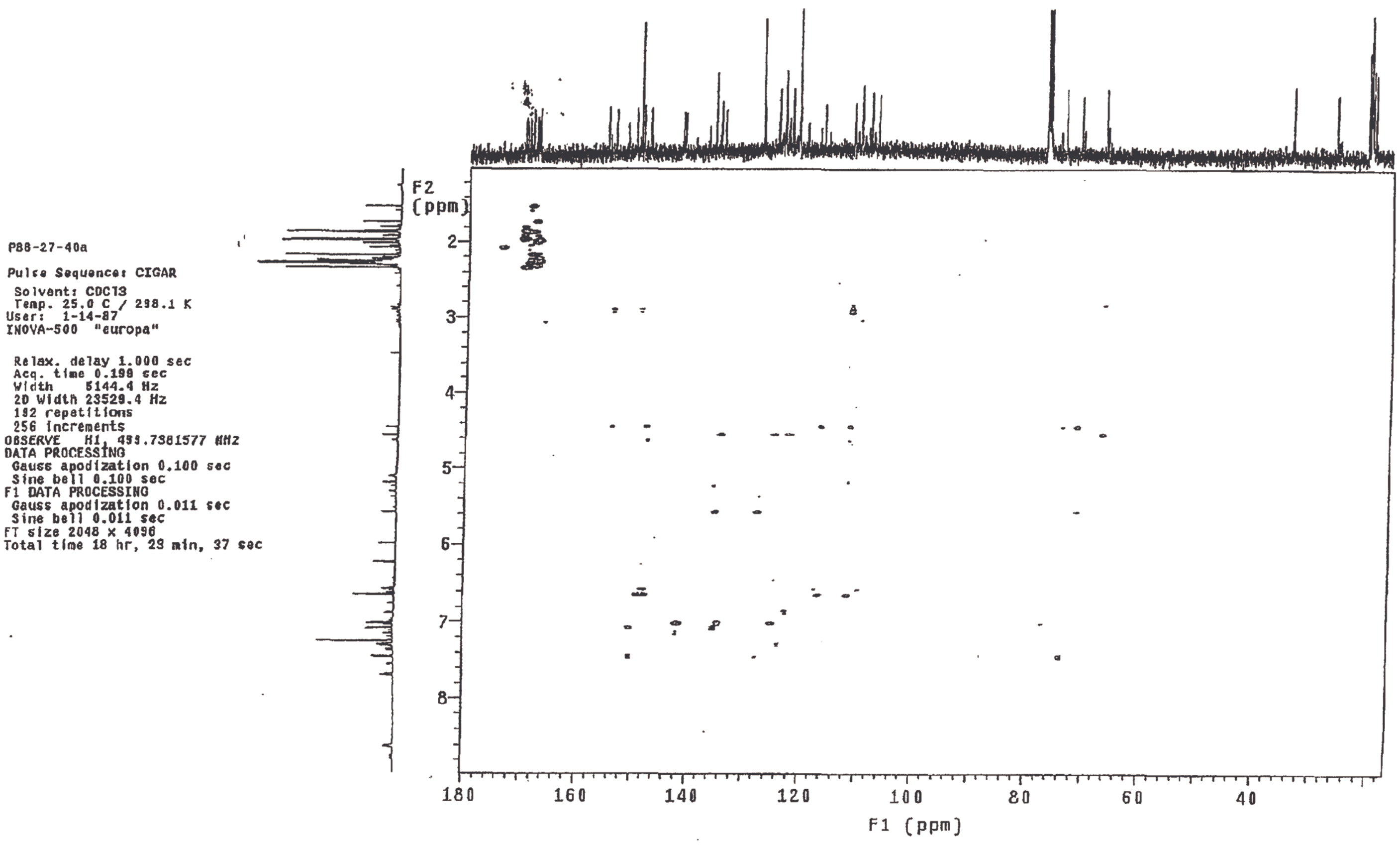

Fig. S6h. CIGAR ^{1}H-^{13}C correlation spectrum (low resolution) of PTI-777 compound k1 peracetate.

Fig. S6i

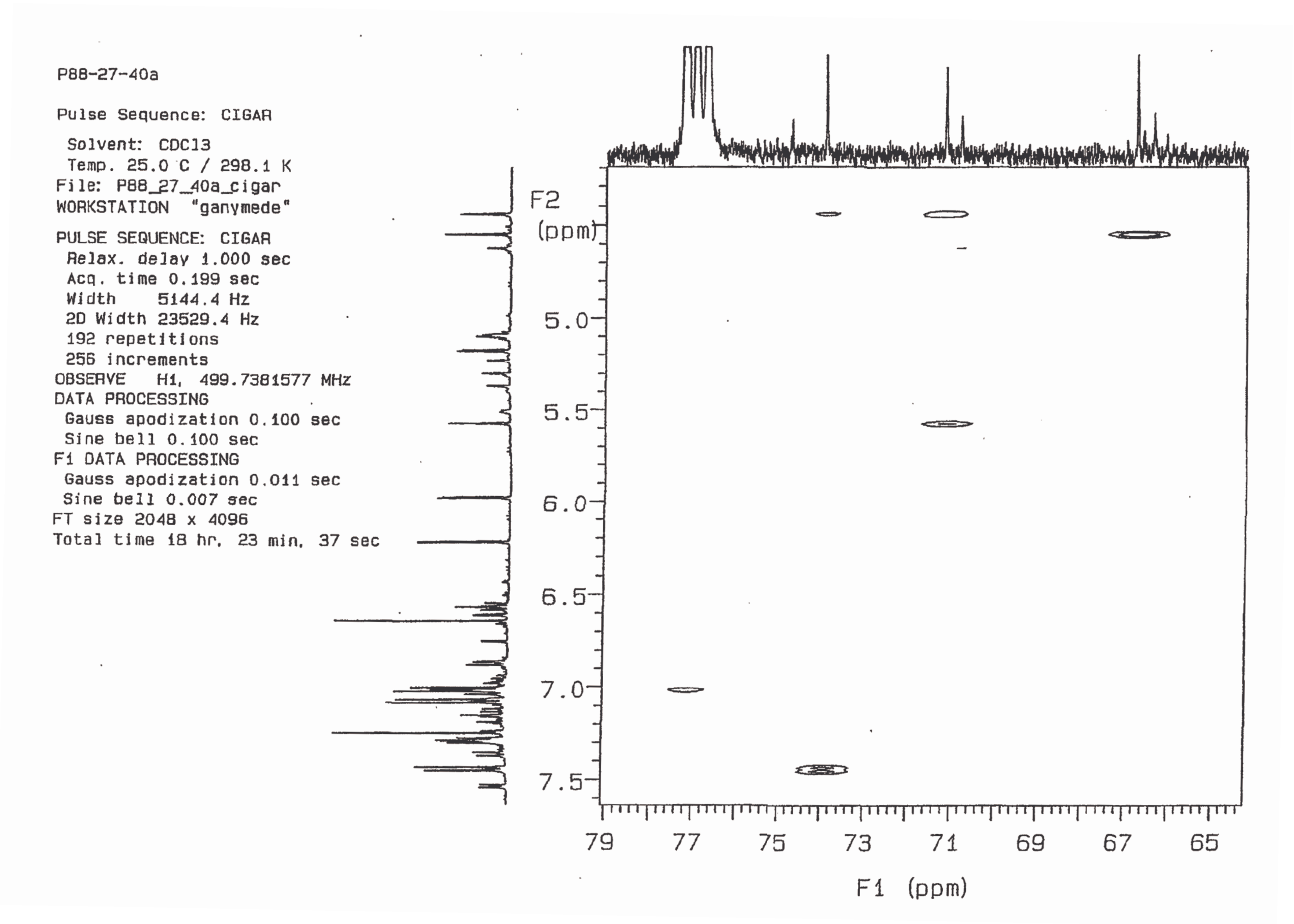

Fig. S6i. CIGAR ^{1}H-^{13}C correlation spectrum (medium resolution) of PTI-777 compound k1 peracetate.

Fig. S6j

P88-27-40a

Pulse Sequence: CIGAR

Solvent: CDCl3
Temp. 25.0 C / 298.1 K
File: P88_27_40a_cigar
WORKSTATION "ganymede"

PULSE SEQUENCE: CIGAR
Relax. delay 1.000 sec
Acq. time 0.199 sec
Width 5144.4 Hz
2D Width 23529.4 Hz
192 repetitions
256 increments
OBSERVE H1, 499.7381577 MHz
DATA PROCESSING
Gauss apodization 0.100 sec
Sine bell 0.100 sec
F1 DATA PROCESSING
Gauss apodization 0.011 sec
Sine bell 0.007 sec
FT size 2048 x 4096
Total time 18 hr, 23 min, 37 sec

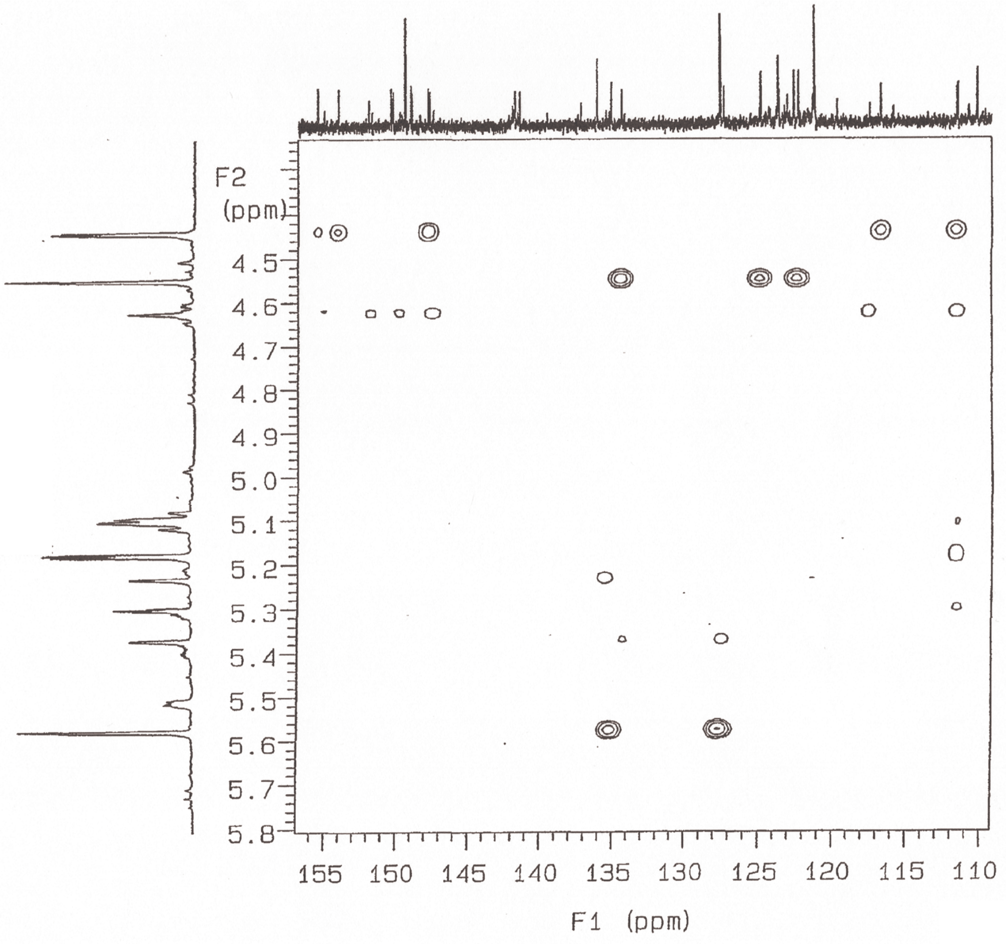

Fig. S6j. CIGAR ^1H-^{13}C correlation spectrum (high resolution) of PTI-777 compound k1 peracetate.

Fig. S7

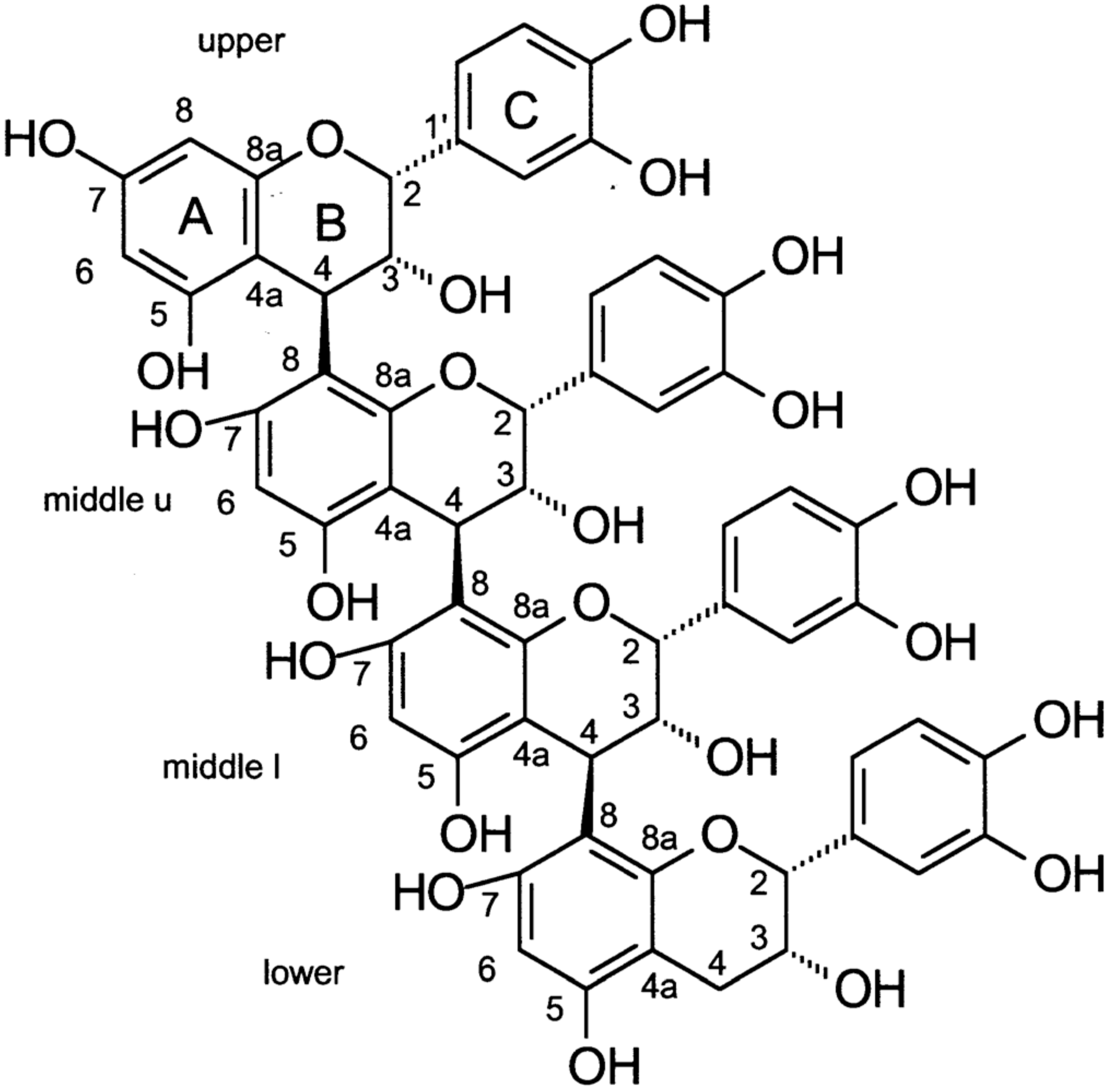

Fig. S7a. The structure of PTI-777 fraction I which is an epicatechin tetramer.
